# MeSH ORA framework: R/Bioconductor packages to support MeSH over-representation analysis

**DOI:** 10.1186/s12859-015-0453-z

**Published:** 2015-02-15

**Authors:** Koki Tsuyuzaki, Gota Morota, Manabu Ishii, Takeru Nakazato, Satoru Miyazaki, Itoshi Nikaido

**Affiliations:** 1 Department of Medical and Life Science, Faculty of Pharmaceutical Science, Tokyo University of Science, 2641 Yamazaki, Noda, 278-8510 Chiba Japan; 2 Department of Animal Science, University of Nebraska-Lincoln, Lincoln, NE USA; 3 Department of Animal Sciences, University of Wisconsin-Madison, Madison, WI USA; 4 Database Center for Life Science (DBCLS), Research Organization of Information and Systems (ROIS), Faculty of Engineering Building 12, The University of Tokyo, 2-11-16 Yayoi, Bunkyo-ku, 113-0032 Tokyo Japan; 5 Bioinformatics Research Unit, Advanced Center for Computing and Communication, RIKEN, 2-1 Hirosawa, Wako, 351-0198 Saitama Japan

**Keywords:** MeSH, Over-representation analysis, Enrichment analysis, Annotation

## Abstract

**Background:**

In genome-wide studies, over-representation analysis (ORA) against a set of genes is an essential step for biological interpretation. Many gene annotation resources and software platforms for ORA have been proposed. Recently, Medical Subject Headings (MeSH) terms, which are annotations of PubMed documents, have been used for ORA. MeSH enables the extraction of broader meaning from the gene lists and is expected to become an exhaustive annotation resource for ORA. However, the existing MeSH ORA software platforms are still not sufficient for several reasons.

**Results:**

In this work, we developed an original MeSH ORA framework composed of six types of R packages, including *MeSH.db*, *MeSH.AOR.db*, *MeSH.PCR.db*, the *org.MeSH.XXX.db*-type packages, *MeSHDbi*, and *meshr*.

**Conclusions:**

Using our framework, users can easily conduct MeSH ORA. By utilizing the enriched MeSH terms, related PubMed documents can be retrieved and saved on local machines within this framework.

**Electronic supplementary material:**

The online version of this article (doi:10.1186/s12859-015-0453-z) contains supplementary material, which is available to authorized users.

## Background

Due to the rapid development of “-omics” technology, such as DNA microarrays [1] and next-generation sequencing (NGS) [2,3], scientists are now able to quantify large numbers of transcripts from organisms simultaneously. The data output of such high-throughput experiments typically becomes overwhelmingly large, and some statistical analyses must be performed to focus on the genes that are related to the experiment being conducted. For example, *p*-values indicating significance [4-14], fold changes [15,16], ranks [17,18], factor loadings in principal component analysis [19] and other scores [20] are calculated and considered. Genes of interest can be selected based on these criteria and are listed such as differentially expressed genes (DEGs), single nucleotide polymorphism (SNPs), insertion/deletion (INDEL) mutations, or copy number variations (CNVs), and so on, depending on the experimental paradigm used.

To extract biological meaning from these lists, over-representation analysis (ORA, or enrichment analysis) is widely employed [21,22]. ORA determines which types of biological terms are significantly enriched among the genes on a given list. The degree of enrichment is calculated as a probability that indicates that particular terms are detected in the gene lists more often than expected by chance. The hypergeometric test (or *Fisher*’s exact test) is widely used to calculate such probabilities. Several thousand statistical tests are conducted against the terms assigned to the list, and only significant terms are extracted. Such terms will help us to gain insight into the biological mechanisms behind the phenomena being investigated. Many annotation resources for ORA have been used, such as Gene Ontology (GO) [23], KEGG [24], Reactome [25], BioCyc [26], BioCarta [27], Disease Ontology [28], and MSigDB [29]. In addition, a wide variety of ORA software has also been developed, including programs such as DAVID/EASEonline [30], FatiGO [31], GOstats [32], topGO, GenMAPP [33], GOMiner [34], GOSurfer [35], FIDEA [36], GOseq [37], EnrichNet [38], OntoTools [39], IPA http://www.ingenuity.com/products/ipa, and BiNGO [40] (comprehensive review of ORA analyses and resources are available in [41-45]).

Recently, Medical Subject Headings (MeSH) [46] terms have also been used for ORA. MeSH is the annotation used for PubMed documents and is manually curated by the U. S. National Library of Medicine (NLM). MeSH has 16 categories and the size of its vocabulary is approximately twice as large as that of GO [47]. MeSH includes biological categories such as “Phenomena and Processes”, “Chemicals and Drugs” and “Anatomy”, as well as the other annotation resources such as GO. MeSH also has a “Diseases” category, which contains many disease-related terms (e.g., “Hypertension” and “Neoplasms”) that facilitate a medical interpretation of data.

Moreover, some MeSH terms are categorized as unique concepts such as “Therapeutic Equipment”, “Anthropology”, “Humanity”, “Psychology”, or even “Information Science”. Therefore, MeSH enables the extraction of broad meaning from the gene lists and is expected to become an exhaustive annotation resource for ORA. In fact, some tools for MeSH ORA are already available, such as Biocompass [47], Gendoo [48], Gene2mesh http://gene2mesh.ncibi.org/, Metab2mesh [49] and Genemesh [50]. However, in certain cases, the implementation of these tools is still not sufficient, for the reasons described below. **1) Few CUI environments**

- With the exception of the application programmable interfaces (API) of gene2mesh and metab2mesh, the available character user interface (CUI) environments are still insufficient. Many bioinformaticians often combine multiple CUI tools and construct an original analytic pipeline on a local machine. Because R developers (notably Bioconductor) provide thousands of R packages to support many types of data analysis and because R is widely used by many “-omics” data analyses, Bioconductor packages are ideal for MeSH ORA. All Bioconductor packages are freely downloadable and accessible for any user. With Bioconductor packages, the output of upstream analyses can be seamlessly input into the function of downstream analysis.

**2) Multiplicity of tests**

- As described above, in ORA, several thousand statistical tests are conducted simultaneously. Such an approach is categorized as a multiple testing problem. Existing MeSH ORA software does not account for this multiplicity. Therefore, type 1 errors accumulate, and the significance level (*p*-value) may cause a high incidence false-positives. Many bioinformatics tools have employed the false discovery rate (FDR) statistic [51] instead of *p*-value, which is appropriate for MeSH ORA.

**3) Time-consuming PubMed searches**

- Many -omics studies focus on the functions of individual genes. To do so, the researcher must retrieve many references from the literature about each gene and investigate the details. This step is very time-consuming, and there is no existing informatics tool to support this step. Because MeSH itself is an annotation of the information contained in PubMed documents, enriched pairs of MeSH terms and Gene IDs can be used to retrieve relevant literature from PubMed based on the co-occurrence of these pairs. No other annotation resouce (such as GO) can perform this step because such terms in other resources correspond only to Gene IDs and not to PubMed IDs. This is the biggest advantage when using MeSH ORA; MeSH terms help users to retrieve relevant literature without manual web browsing. However, the existing MeSH ORA software does not implement such a function.

For these reasons, we designed an original MeSH ORA framework to meet the requirements stated above.

## Implementation

### Framework and implementation

**Figure 1:**
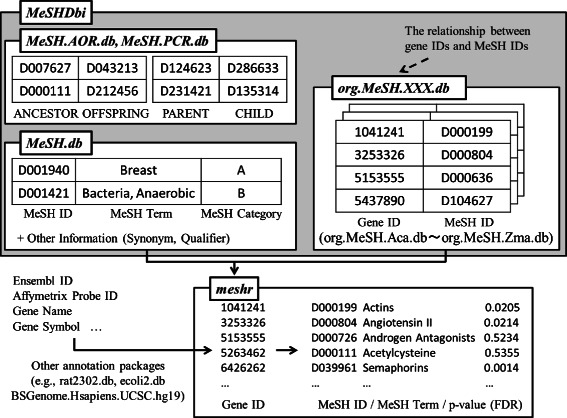
**MeSH ORA framework and dependency of the packages.** Our MeSH framework consists of six types of R packages: the *MeSH.db*, *MeSH.AOR.db*-type packages, *MeSH.PCR.db*, *org.MeSH.XXX.db*, *MeSHDbi*, and *meshr*. *MeSHDbi* defines the class used in the *MeSH.db*, *MeSH.AOR.db*, *MeSH.PCR.db*, and *org.MeSH.XXX.db*-type packages and then unifies the behavior of these packages. *meshr* imports the data from *MeSH.db* and *org.MeSH.XXX.db* and performs ORA.

Our framework consists of six types of R packages: *MeSH.db*, *MeSH.AOR.db*, *MeSH.PCR.db*, the *org.MeSH.XXX.db*-type packages (where XXX represents the abbreviation for an organism such as “Hsa” for *Homo sapiens*), *MeSHDbi* and *meshr* (Figure 1). These packages are freely available from Bioconductor [52] 2.14 under the Artistic-2.0 public license.

### *MeSH.db*

*MeSH.db* outputs general information from MeSH. The MeSH data in the NLM database (http://www.nlm.nih.gov/mesh/filelist.html) are stored internally in *MeSH.db*. NLM MeSH database is updated annually, and version 1.0.0 of *MeSH.db* supplies the MeSH data from 2014. MeSH has 16 categories and each category is expressed as a single capital letter, as defined by NLM (Table 1).

**Table 1:** **Categories of MeSH:** ***MeSH.db*** **provides 16 categories of MeSH terms in 2013**

**Abbreviations**	**Categories**	**No. of terms**	**Examples**
A	Anatomy	2882	Muscles, Skeleton
B	Organisms	5169	Gram-negative Bacteria
C	Diseases	111257	Leukemia, Burns
D	Chemicals and Drugs	20633	Fatty Acids, Ligases
E	Analytical Diagnostic and Therapeutic	4720	Dental Care
Techniques and Equipment			
F	Psychiatry and Psychology	1127	Behavior, Motivation
G	Phenomena and Processes	3352	Antibody Formation
H	Disciplines and Occupations	495	Biology, Clinical Medicine
I	Anthropology, Education, Sociology and	622	Economics, Culture
Social Phenomena			
J	Technology and Food and Beverages	597	Bread, Coffee, Tea
K	Humanities	216	Music, Religion
L	Information Science	505	Communications Media
M	Persons	245	Adult Children, Drug Users
N	Health Care	2297	Oral Health, Women’s Health
V	Publication Type	180	Book Illustrations, Letter
Z	Geographical Locations	546	Japan, China, Taiwan

Each category is abbreviated as a single capital letter defined by NLM.

*MeSH.db* also provides qualifier terms, which are broader categories (e.g., pathology, history, and genetics) and synonyms, which are more colloquial words with that have same meanings as the MeSH terms.

### *MeSH.AOR.db* and *MeSH.PCR.db*

*MeSH.AOR.db* and *MeSH.PCR.db* provide information regarding to the MeSH database structure. The MeSH database has a tree structure similar to that of the GO database; i.e., its structure is hierarchical, and the higher-order terms (e.g., cancer) include the lower-order terms (e.g., breast cancer). *MeSH.AOR.db* and *MeSH.PCR.db* provide ancestor-offspring relationships (AOR) and parent-child relationships (PCR), respectively.

### *org.MeSH.XXX.db*-type packages

*org.MeSH.XXX.db*-type packages provide the correspondence between NCBI Entrez Gene IDs and NLM MeSH IDs for each organism. Only the data that have sufficiently high correspondence between the Entrez Gene and MeSH IDs were selected.

**Figure 2:**
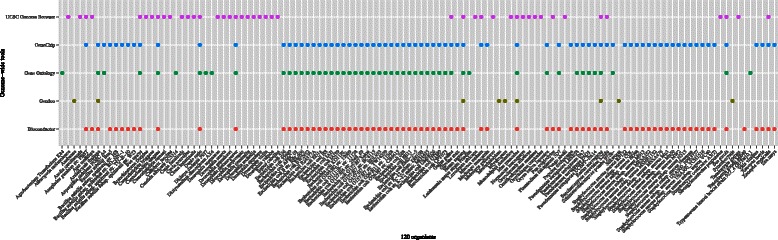
**Genome-wide tools.** We focused on organisms that are used in genome-wide tools such as the UCSC Genome Browser, GeneChip, Gene Ontology, Gendoo, and Bioconductor.

**Figure 3:**
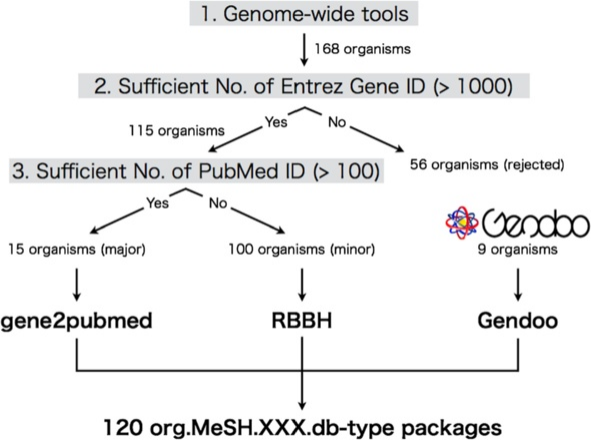
**120 orgnisms.** To construct *org.MeSH.XXX.db*-type packages, we focused on organisms satisfying three requirements: 1) use in at least one of five genome-wide tools; 2) possession of an Entrez Gene ID, rather than an Ensembl Gene ID; and 3) published data abailable in at least 100 papers. Finally, 120 organisms were selected for the framework.

First, we focused on the organisms used in at least one of five available genome-wide tools: Affymetrix GeneChip [1], Gene Ontology [23], Bioconductor [52], UCSC Genome Browser [53] and Gendoo [48] (Figure 2). Overall, 168 organisms were selected by this criterion (Figure 3).

Next, we focused on the organisms that had at least 1000 of Entrez Gene IDs. Some organisms have only Ensembl Gene IDs but no Entrez Gene IDs, and because our framework uses only Entrez Gene IDs, such organisms were rejected. After this step, 115 organisms remained.

Finally, we classified the remaining organisms as major or minor. Here we defined major and minor organisms by whether the organisms have at least 100 PubMed IDs. We defined 15 well-annotated organisms as “major organisms”: *Arabidopsis thaliana*, *Bacillus subtilis subsp. spizizenii str. 168*, *Bos taurus*, *Caenorhabditis elegans*, *Drosophila melanogaster*, *Danio rerio*, *Escherichia coli str. K-12 substr. MG1655*, *Gallus gallus*, *Homo sapiens*, *Mus musculus*, *Rattus norvegicus*, *Saccharomyces cerevisiae S288c*, *Shizosaccharomyces pombe 972h-*, *Sus scrofa*, and *Xenopus laevis*. In some cases, thousands of Entrez Gene IDs are assigned in a single publication. Most such papers describe the determination of genome sequences or genome projects/databases rather than specific gene functions. Therefore, we omitted those papers that were assigned more than 1000 Entrez Gene IDs.

**Figure 4:**
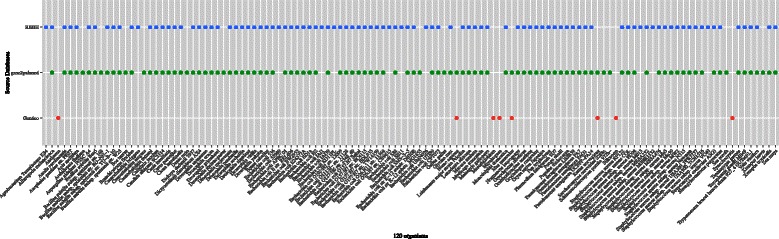
**Data Sources.** The data source of the 120 organisms in our framework. Three data sources - RBBH, gene2pubmed, Gendoo - were choosen.

Three ways of corresponding the Entrez Gene and MeSH IDs was used in these organisms: Gendoo (http://gendoo.dbcls.jp/data/), gene2pubmed (ftp://ftp.ncbi.nih.gov/gene/DATA/), and RBBH (reciprocal BLAST best hit). Gene2pubmed is used against 15 major and 100 minor organsisms, and RBBH is used against only the 100 minor organisms. Gendoo is used against only 9 organisms included in the Gendoo website (http://gendoo.dbcls.jp/). Figure 4 shows the 120 organisms and their data sources.

**1) gene2pubmed**

- gene2pubmed provides the correspondence between the Entrez Gene and PubMed IDs assigned by NCBI manual curation. We converted these PubMed IDs to their corresponding MeSH terms using data licenced by PubMed (http://www.nlm.nih.gov/databases/license/license.html), and then converted these MeSH terms to MeSH IDs using the data from the NLM MeSH (Figure 5).
  **Figure 5:**
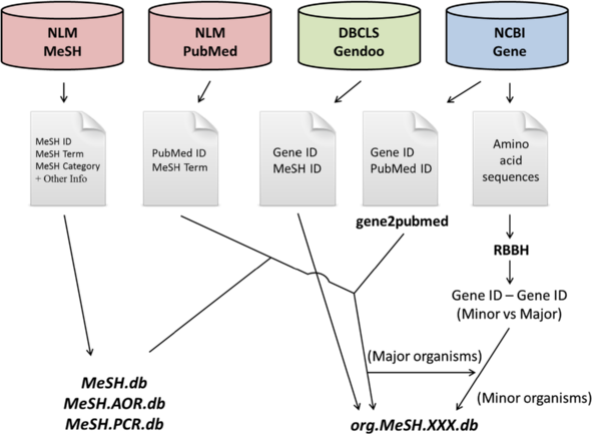
**Data retrieval schema for the construction of** ***MeSH.db*** **and** ***org.MeSH.XXX.db*** **.** *MeSH.db* uses the data for MeSH terms from NLM. *org.MeSH.XXX.db* uses the data from Gendoo, gene2pubmed and RBBH.

***2) RBBH***

**Figure 6:**
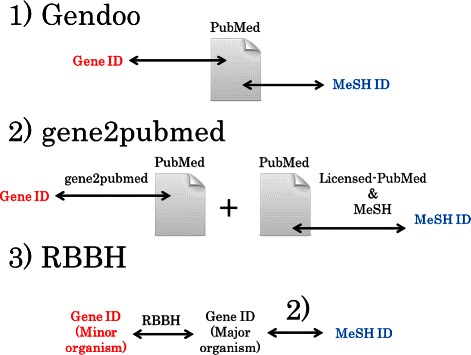
**Three types of correspondence between Entrez Gene ID and MeSH ID.** *org.MeSH.XXX.db*-type packages provide three types of correspondence between Entrez Gene ID and MeSH ID: 1) Gendoo data, in which the correspondence is assigned by a text-mining technique; 2) gene2pubmed data, in which the correspondence is assigned by manual curation of NCBI; and 3) RBBH data, in which the correspondence is assigned by using reciprocal BLASTP best hits among all possible combinations of minor organisms and major organisms.

**Figure 7:**
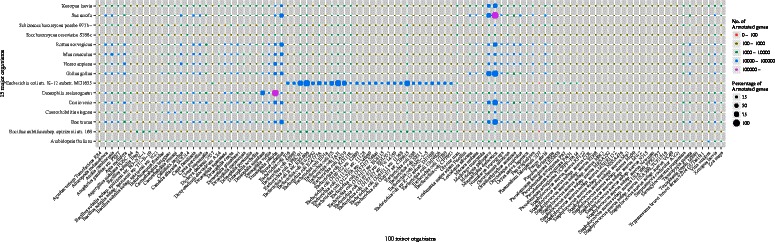
**RBBH.** A comparison of 100 minor organisms with 15 major organisms by RBBH was conducted.

- Due to the lack of relevant literature, minor organisms were poorly annotated by 1) gene2pubmed approarch. Because many gene and protein annotations are based on sequence similarity against other organisms such as FANTOM project [54,55], GO [23], blast2go [56], InterProScan [57], and RAPSearch [58], we conducted a sequence homology search of these minor organisms against the major organisms and then applied the MeSH IDs of the major organisms to these minor organisms (Figure 6). We performed a reciprocal BLAST [59] best-hit search among all possible combinations of the 15 major organisms and 100 minor organisms (Figure 7). A total of 3000 (100 minor organisms × 15 major organisms × 2-direction) blastp programs were performed (E-values are 50).

**3) Gendoo**

- The Entrez GeneID - MeSH ID relationship in Gendoo was assigned using a text-mining technique. The degree of relevance between the Entrez Gene and MeSH IDs was assessed by the *Kullback-Leibler* divergence [60].

A summary of the 120 organisms in each MeSH category is given in Additional file 1.

### *MeSHDbi*

The *MeSHDbi* package has two roles; class definition and aiding in the construction of custom *org.MeSH.XXX.db*-type packages by the user. **1) Class definition**

**Figure 8:**
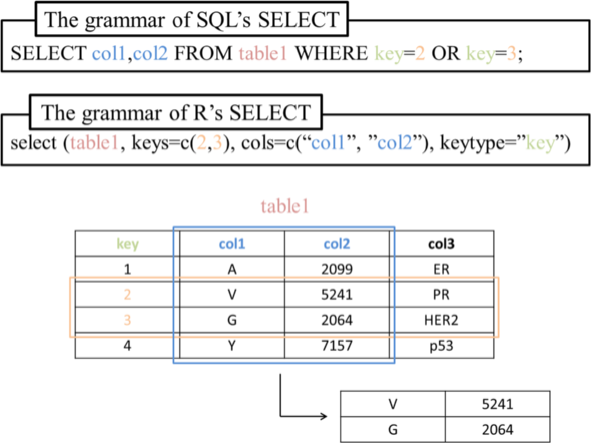
**SELECT function in R.** *AnnotationDbi* package declares that data are designed to be retrieved by the SELECT function. Its grammar is similar to that of SQL’s SELECT method and very simple.

- *MeSH.db*, *MeSH.AOR.db*, *MeSH.PCR.db* and the *org.MeSH.XXX.db*-type packages follow the classes and methods of S4, which is the R OOP (Object-Oriented Programming) system. The *MeSHDbi* package defines an S4 class named “MeSHDb” and then unifies the grammar of the functions of these packages. Thus, the behavior of these packages is unified by *MeSHDbi* such that the user can input the same commands for all packages. This implementation is not only for the users but also for the developers, because it reduces the amount of source code.
  The “MeSHDb” class has a “SELECT” function, which is used for data retrieval (Figure 8). The user specifies three optional parameters in SELECT: keys, cols and keytype. This grammar is very similar to that of SQL languages, which have been used for the management of relational databases.

**2) Custom** ***org.MeSH.XXX.db*** **-type package construction**

- Although we implemented 120 *org.MeSH.XXX.db*-type packages, more genomes are being sequenced thanks to the rapid spread of NGS technology. Additionally, some users may want to utilize the correspondence between Gene IDs and MeSH IDs designed in other databases, which we currently do not support [47,49,50]. Therefore, we implemented the *makeGeneMeSHPackage* function, which enables the users to construct an original *org.MeSH.XXX.db*-type package.

### *meshr*

*meshr* imports data from *MeSH.db* and *org.MeSH.XXX.db*-type packages and then performs MeSH ORA against a gene list of the user’s choice. The hypergeometric test evaluates the types of MeSH terms that are enriched in the gene lists. The *p*-value is defined by the following equation:

(1) $$ p = \frac{{~}_{M}C_{x} \times {~}_{N - M}C_{k - x}}{{~}_{N}C_{k}}  $$

where N is the total number of all genes, k is number of interesting genes (e.g., DEGs), M is the number of genes assigned to a MeSH term and x is the number of interesting genes (e.g., DEGs) assigned to a MeSH term.

In the *meshr* package, the following three FDR control methods are implemented for multiple testing. **1)** ***Benjamini-Hochberg*** **(BH) method**

- The BH method [51] assumes a uniform distribution of *p*-values when all null-hypotheses (e.g., non-DEGs) are true. The BH method defines the threshold of the *q*-value instead of the *p*-value. The *q*-value is an expectation value of FDR. The procedures of the BH method is as follows.
  - Set an *α* threshold, where 0<*α*<1.
  - Sort the observed *p*-values in ascending order, such as *p*_1_≤*p*_2_≤..≤*p*_*m*_, where m is the number of hypothesis tests.
  - For each *i*th *p*-value, calculate $q_{i} = \frac {p_{i} \times N}{i}$, where N is the number of *p*-values.
  - If the *k*th *q*-value is less than *α*, then reject the null-hypotheses corresponding to *p*_1_≤*p*_2_≤...≤*p*_*k*_. Otherwise, reject nothing.

**2)** ***Q*** **-value and local FDR**

- In contrast to the BH method, the *Q*-value [61,62] and the local FDR [63,64] methods hypothesize that *p*-values are from mixture distribution of null-hypotheses and alternative hypotheses (e.g., DEGs), where the mixture ratio is *π*_0_ : 1 - *π*_0_.
  In *Q*-value, *π*_0_ is estimated from a *p*-value histogram using a natural cubic spline curve [62]. The estimated *π*_0_ is multiplied to *q*-value in BH method:
  (2) $$ Q_{i} = \frac{\pi_{0} \times p_{i} \times N}{i},  $$
  whereas, local FDR uses a Bayesian approach. *π*_0_ is assumed to be a prior distribution in Bayes’ theorem, and FDR is estimated using the following equation:
  (3) $$ LocalFDR = \frac{f_{0}(p) \times \pi_{0}}{f(p)},  $$
  where, *f*_0_(*p*) is the null-hypothesis density distribution function and *f*(*p*) is the density distribution function of the observed *p*-values.

These methods are expected to work appropriately even if the *p*-values are not uniformly distributed. Therefore, the users should first observe the distribution of *p*-values and then choose the appropriate method.

*meshr* also retrieves PubMed documents related to enriched MeSH terms, which saves the researcher some of the time that would have been spent searching the related literature. The documents can be saved in PDF or HTML format on a local machine, and a directory can be organized by Gene ID, MeSH ID or PubMed ID. Therefore, a researcher can directly call the publications in which a gene of interest was studied.

## Results and discussion

### Summary of MeSH assignment to Entrez gene ID

MeSH IDs and Gene IDs are linked from the data sources gendoo, gene2pubmed, and RBBH. Here, we demonstrate how many genes are assigned to MeSH, how much MeSH and existing annotation are duplicated and how many poorly annotated genes are newly annotated by MeSH, for each organism meeting our criteria.

**Figure 9:**
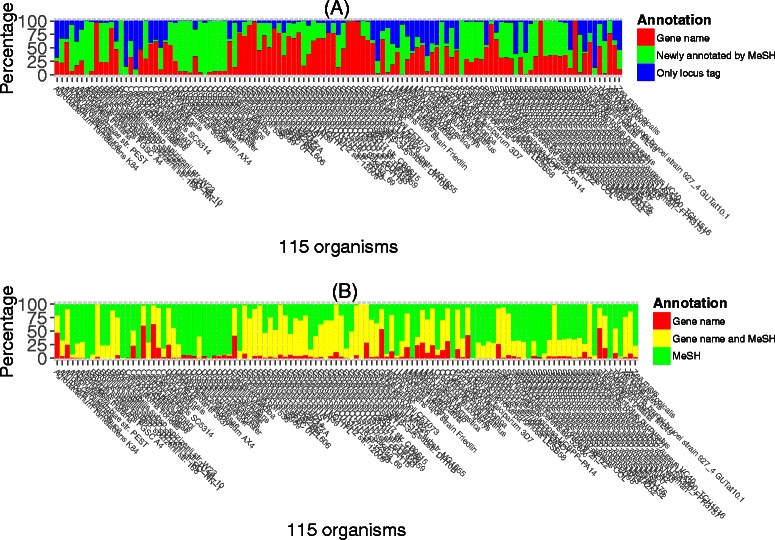
**Summary of MeSH Assignment to Entrez Gene ID.** Detailed coverage of MeSH against all genes of 115 organisms without Synechocystis, Miyakogusa, Mesorhizobium, Anabaena, and Silkworm. We hypothesized that genes assigned to gene name is well-annotated genes and genes assigned to only locus tag is not-annotated genes. **(A)** The genes assigned to gene name (red), the genes assigned to only locus tag (blue), and the genes newly annotated by MeSH in this work (green). **(B)** The genes assigned to only gene name (red), the genes annotated by gene name and MeSH (green), and the genes newly annotated by MeSH in this work (blue).

A summary of the MeSH assignment of all genes of 115 organisms is shown in Figure 9 (the Entrez Gene IDs of *Synechocystis*, *Miyakogusa*, *Mesorhizobium*, *Anabaena*, and *Silkworm* are not assigned to a specific species). We hypothesized that the genes assigned a name were annotated and the genes assigned only a locus tag were unannotated. Figure 9 (A) shows that the genes assigned a name (red, the average value is 39.61), a MeSH (green, the average value is 10.04), and a locus tag (blue, the average value is 50.34) in this work. Figure 9 (B) shows that the genes assigned gene only (red, the average value is 33.97), a gene name and a MeSH (yellow, the average value is 34.25), and MeSH only (green, the average value is 31.77). In the case of *Aspergillus nidulans FGSC A4*, most genes were not previously annotated (B, red) but were newly annotated by MeSH in this work (A, green). In contrast, the percentage of genes newly annotated by MeSH was low in some strains of *Escherichia coli*. It may be because the genes of the wild type strain *Escherichia coli K-12 substr. MG1655* are extremely well assigned (approximately 100%) and the gene annotation of the other *E. coli* strains are based on this wild-type strain. Precise number of the coverage of MeSH in each organisms is summarized in Additional file 2.

### USAGE

**Figure 10:**
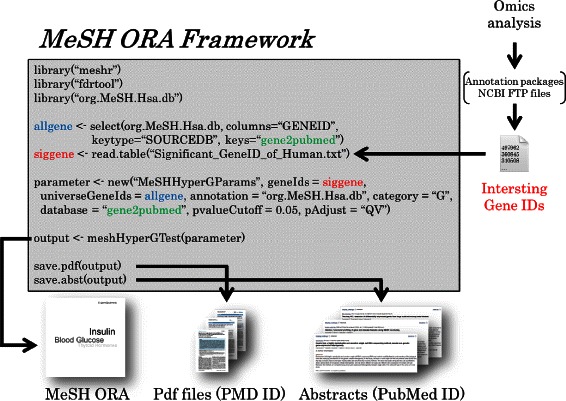
**Simple R code for MeSH ORA.** MeSH ORA for *Homo sapiens* can be performed by a simple R code.

We demonstrate the generation of a simple MeSH ORA by using the R script (Figure 10). The download process of our packages will finished in about several minutes (we calculated the time using an iMac, 8GB RAM, 64bit, Intel Core i7, and Mac OS X v-10.10 Yosemite). To perform MeSH ORA, the *meshr* package is loaded on the 1st line. The *fdrtool* package, which is used for the FDR adjustment, is loaded on the 2nd line. On the 3rd line, the correspondence between the Entrez Gene IDs and MeSH IDs is loaded. On the 4th and 5th lines, all Entrez Gene IDs and interesting Gene IDs are loaded. All Entrez Gene IDs are assumed to be retrieved from org.MeSH.XXX.db-type packages. Interesting Gene IDs are assumed to be generated by some omics-analyses. At present, *meshr* accepts only Entrez Gene IDs as input. Therefore, if a user starts with Ensembl Gene ID, Affymetrix probe ID, or another identifier, he or she has to convert these IDs to Entrez Gene IDs. Such a task can be easily performed by a pre-existing annotation packages (e.g., *Homo.sapiens*, *Mus.musculus*, *Rattus.norvegicus*, and *biomaRt* [65,66] packages). These packages will convert IDs from ID lists such as Ensembl Gene ID, RefSeq ID, Locus Tag, or gene name. The NCBI FTP site (ftp://ftp.ncbi.nih.gov/gene//DATA/) also provides some useful files, which may help to retrieve Entrez Gene IDs from different forms of ID.

- RefSeq ID to Entrez Gene ID : gene2refseq
- Taxonomy ID to Entrez Gene ID : gene2accession
- Locus tag to Entrez Gene ID : gene2accession
- Gene name to Entrez Gene ID : gene2accession
- Ensembl Gene ID to Entrez Gene ID : gene2ensembl
- GO ID to Entrez Gene ID : gene2go
- PubMed ID to Entrez Gene ID : gene2pubmed
- UniGene ID to Entrez Gene ID : gene2unigene
- STS ID to Entrez Gene ID : gene2sts

Each of the 120 organisms can be loaded in the same way, and the same analysis can be performed. The parameters for the MeSH ORA are defined on the 6th line. This procedure is the same as that used by other ORA packages such as *GOstats* [32]. Here, MeSH “D” (Chemicals and Drugs) category from the Gendoo dataset is set using a 0.05 threshold for QV (*Q*-value method) FDR adjustment. The function *meshHyperGTest* performes the MeSH ORA against the prepared parameters. The result is summarized in a table from which the user can retrieve additional information such as significant MeSH terms or corresponding Entrez Gene IDs.

We can also retrieve the documents of related studies seamlessly by using the save.pdf and save.abst functions. By these functions, PDF files can be downloaded onto a local machine, which saves the time spent on a PubMed search, allowing the researchers to spend more time on downstream data analysis involving biological interpretation (an abstract retrieval function will also be implemented by Bioconductor 3.1). The *meshr* package provides two types of documentation: manual pages to explain all functions and optional parameters and a vignette containing the R source-code, description, and figures. The user can refer to these documents and conduct further sophisticated analyses by combining this package with other Bioconductor packages.

### Case-study

Here, we demonstrate the usability of our framework by re-analyzing past genome-wide studies. We used a DNA-microarray dataset for a calorie-restricted rat as a representative study of a major organism and the NGS dataset of *Pseudomonas aeruginosa* - a known drug-resistant bacterium - as a minor organism.

#### Case-study 1: *Rattus norvegicus* (Rat) as a major organism

Caloric restriction (CR) has been suggested to be associated with longevity. *Chujo et al*. found that life-long CR is related to the remodeling of white adipose tissue (WAT) by analyzing the gene expression of rats under CR conditions [67].

The author used 4 CR rats and 4 control rats and compared them by Affymetrix Rat Genome 230 2.0 Array (GEO Accession: GSE30668). DEGs were detected by two-way ANOVA (*p* < 0.001) using superNORM [68] normalization, principal component analysis and further evaluation by quantitative real-time RT-PCR. They used the GO (Biological Process) database to annotate 199 upregulated DEGs and 226 downregulated DEGs. 36 GO terms showed that the DEGs were involved in “lipid biosynthesis” or “inflammation”.

**Figure 11:**
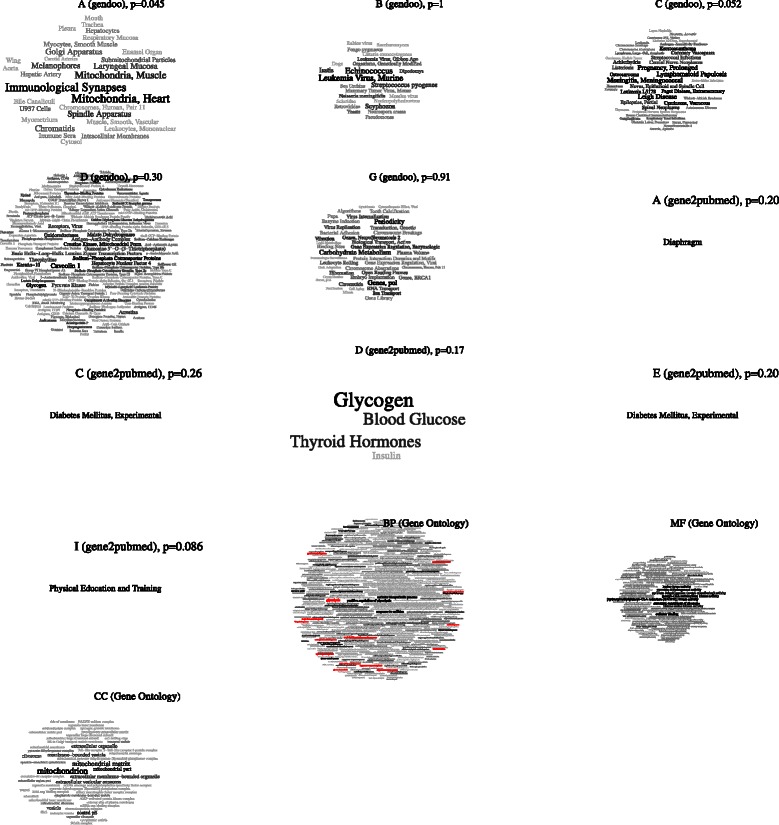
**ORA with calorie-restricted rats.** Enrichment analysis using Gendoo, gene2pubmed and GO. A (Anatomy), B (Organisms), C (Diseases), D (Chemicals and Drugs), G (Phenomena and Processes) of Gendoo, A, C, D, E (Analytical Diagnostic and Therapeutic Techniques and Equipment), I (Anthropology, Education, Sociology and Social Phenomena) of gene2pubmed, and BP (Biological Process), MF (Molecular Function), CC (Cellular Component) of GO. Only enriched terms are drawn by the *tagcloud* package (*p* < 0.05). The minus logarithm of *p*-values is used as weight and emphasized as the font size.

We re-analyzed the same data using by the *t*-test with the FDR method (*Q*-value) and detected 505 DEGs (*Q* < 0.2, see Additional file 3). We conducted ORA with these gene lists (Figure 11). 15 of the 36 previously identified GO terms were detected again (the red words in the tagcloud of Biological Process in Figure 11).

Well-known CR-related terms such as “Diabetes Mellitus, Experimental” (C of Gendoo and gene2pubmed, E of gene2pubmed), “Carbohydrate Metabolism” (G, Gendoo), “Cell Aging” (G, Gendoo), and “Blood Glucose” and “Insulin” (D, gene2pubmed) are detected. As reported by *Chujo et al.*, mitochondrial biogenesis is also enhanced by CR. For example, in A and D (Gendoo), and CC (Gene Ontology), “Submitochondrial Particles”, “Mitochondria, Muscle”, “Mitochondria, Heart”, “Mitochondrial matrix”, “Mitochondrion”, “Mitochondrial pyruvate dehydrogenase”, and “Mitochondrial part” are actually enriched.

Moreover, by using ORA within a MeSH framework, we can retrieve additional information, including immune-related terms such as “Immunological Synapses” and disease names such as “Meningitis, Meningococcal”, “Spinal Neoplasms” (C, Gendoo), and health-related scientific terms such as “Physical Education and Training”. These terms are expected to help researchers to consider other hypotheses that cannot be constructed using only GO.

#### Case-study 2: *Pseudomonas aeruginosa* as a minor organism

*Pseudomonas aeruginosa*, a clinically important bacterium, is known for its innate antibiotic resistance. *Gallagher et al.* developed the Tn-seq methodology based on NGS and the amplification of single-strand circles carrying transposon junction sequences that contribute to drug resistance [69]. The authors focused on 28 genes identified by Tn-seq as exhibiting strong mutant hypersensitivity to tobramycin.

They annotated these genes by gene description (NCBI). Known mutations related to drug resistance occurred in some genes, including those encoding the MexXY-OprM efflux pump (ABC-transporter) and a potassium uptake transporter.

Due to the usage of MeSH, the amount of annotation was considerably increased. Tables 2, 3 and 4 show a partial list of the results, and Additional file 3 contains the complete data for this analysis. Corresponding terms such as “Cell membrane” (BP of GO, A of MeSH), “ATP-binding cassette (ABC) transporter complex” (CC of GO, D of MeSH), and “Pseudomonas aeruginosa” were assigned by our framework and GO (Tables 2, 3 and 4). Furthermore, MeSH returned other bacterial names (e.g., *Escherichia coli*, *Salmonella phimurium*, and *Bacillus subtilis*), the names of biological experiments (“Gene Knockout”, “Cloning” and “Transfection”), some clinical descriptions (“Drug Resistance, Microbial” and “Biofilm”) and even the field of study (“Systems Biology” and “Computer Simulation”). Such terms are unique and only MeSH can be used for the their annotation because GO only provides a molecular biological vocabulary.

**Table 2:** **GO and MeSH annotations for antibiotic registance** ***Pseudomonas aeruginosa*** **: comparison of NCBI gene descriptions, GO, and MeSH**

**Locus**	**Symbol**	**ID**	**Description**	**Gene ontology**		
				**BP**	**MF**	**CC**
PA3303	-	882468	(control)	-	-	-
PA0392	-	878514	Conserved hypothetical	-	-	-
PA4077	-	878707	Transcriptional regulator	-	-	-
PA5199	*amgS*	880300	Two-component sensor	Phosphorelay signal transduction system	Phosphorelay sensor kinase activity	-
PA5366	*pstB*	881628	Phosphate transport	Transport	-	Membrane (+1)
PA5200	*amgR*	880301	Two-component response regulator	Phosphate-containing compound metabolic process (+2)	Phosphorelay response regulator activity	-
PA0016	*trkA*	879255	Potassium uptake	Transport (+1)	Glutamyl-tRNA reductase activity (+1)	-
PA4942	*hflK*	877755	Protease subunit	Cytokinesis by binary fission (+2)	-	-
PA3014	*faoA*	878680	Fatty acid oxidation	Cellular amino acid metabolic process (+11)	Benzoylformate decarboxylase activity	-
PA4398	-	881355	Two-component sensor	Phosphate ion transport	-	-
PA5528	-	877964	Hypothetical	-	-	-
PA1805	*ppiD*	878369	Peptidyl-prolyl isomerase	Protein folding (+1)	Chorismate synthase activity	-
PA3016	-	879098	Hypothetical	-	-	-
PA4223	-	880074	Transport	Lipid transport	-	-
PA4960	-	878558	Amino acid metabolism	L-serine biosynthetic process	-	-
PA0374	*ftsE*	883078	Cell division	Cytokinesis (+2)	-	-
PA3013	*foaB*	880523	Fatty acid oxidation	Cellular amino acid metabolic process (+7)	Thioredoxin-disulfide reductase activity	-
PA5471	-	877632	Hypothetical	Translational termination	-	-
PA0502	-	878654	Biotin biosynthesis	Biotin biosynthetic process	-	-
PA3194	*edd*	882909	Carbohydrate metabolism	Generation of precursor metabolites and energy (+3)	Phosphoenolpyruvate carboxylase activity	-
PA1775	*cmpX*	877590	Cytoplasmic membrane protein	-	-	-
PA0427	*oprM*	877851	Multidrug efflux	transport (+1)	Porin activity (+1)	Membrane
PA4222	-	880073	Transport	Transport	-	ATP-binding cassette (ABC) transporter complex
PA5369	*pstS*	880528	Phosphate transport	-	-	-
PA2018	*mexY*	878882	Multidrug efflux	Transport (+1)	-	Membrane (+1)
PA5285	-	878098	Hypothetical	-	-	-
PA2604	-	882310	Conserved hypothetical	-	-	-
PA2019	*mexX*	878839	Multidrug efflux	Transport (+1)	-	-
PA4050	*pgpA*	879074	Phospholipid biosynthesis	Cellular lipid metabolic process	-	-

**Table 3:** **GO and MeSH annotations for antibiotic registance** ***Pseudomonas aeruginosa*** **: comparison of NCBI gene descriptions, GO, and MeSH**

**Locus**	**MeSH**					
	**A, p=0.0064**	**B, p=0**	**C, p=0.12**	**D, p=0**	**E, 3.9E-9**	**G, p=0**
PA3303	-	-	-	-	-	-
PA0392	-	-	-	-	-	-
PA4077	-	-	-	-	-	-
PA5199	Periplasm (+7)	Bacillus subtilis (+9)	Chromosome Deletion	DNA, Bacteria (+88)	Gene Knockout (+39)	Alleles (+98)
PA5366	Cell Membrane (+1)	Escherichia coli (+5)	Chromosome Deletion	RNA, Bacterial (+33)	Cloning (+14)	Binding Sites (+31)
PA5200	Cell Membrane (+10)	Salmonella typhimurium (+11)	Urinary Tract Infections (+2)	5’ Untranslated Regions (+115)	Amino Acid Substitution (+50)	Biofilm (+110)
PA0016	Cell Membrane (+8)	Enterobacteriaceae (+11)	Salmonella Infections	Acetates (+89)	Cell Fractionation (+17)	Drug Resistance, Microbial (+66)
PA4942	Cytoplasm (+10)	Bacillus subtilis (+10)	-	Adenosine Triphosphatases (+65)	Cloning, Molecular (+28)	Amino Acid Motifs (+73)
PA3014	Liver	Escherichia coli (+7)	-	Proteome (+58)	Methods (+15)	Kinetics (+37)
PA4398	-	-	-	-	-	-
PA5528	-	-	-	-	-	-
PA1805	Capsid (+4)	Escherichia coli (+1)	-	Sigma Factor (+31)	Mutagenesis (+13)	Cell Division (+32)
PA3016	-	-	-	-	-	-
PA4223	-	Pseudomonas aeruginosa	-	ATP-Binding Cassette Transporters (+4)	Gene Expression Profiling (+2)	Gene Expression Regulation, Bacterial (+4)
PA4960	-	Bacteriophage lamda	-	Alcohol Oxidoreductases (+35)	Autoradiography (+14)	Carbohydrate Metabolism (+38)
PA0374	Chromosomes (+4)	Escherichia coli (+2)	Chromosome Deletion	Magnesium (+42)	Culture Media (+11)	Operon (+50)
PA3013	Chromosomes, Bacterial (+2)	Swine (+5)	-	Acetyl Coenzyme A (+33)	Genetic Complementation Test (+8)	Promoter Regions, Genetic (+24)
PA5471	Ribosomes	Pseudomonas aeruginosa	-	Anti-Bacterial Agents (+8)	Microbial Sensitivity Test (+4)	Drug Resistance, Multiple (+10)
PA0502	-	-	-	-	-	-
PA3194	Cell-Free System (+2)	Salmonella typhimurium (+3)	Chromosome Aberrations	DNA, Bacterial (+45)	Spectrophotometry (+8)	Sequence Homology, Nucleic Acid (+28)
PA1775	-	-	-	-	-	-
PA0427	Brain (+6)	Animals, Newborn	Meningitis, Bacteria (+1)	Copper (+76)	Absorptiometry (+39)	DNA Damage (+67)
PA4222	-	-	-	-	-	-
PA5369	-	-	-	-	-	-
PA2018	Ribosomes	Escherichia coli (+1)	(+9)	Peptides (+9)	Transfection (+3)	Plasmids (+12)
PA5285	-	-	-	-	-	-
PA2604	Cytosol (+9)	Spheroplasts (+6)	-	Chaperonins (+31)	Statistics as Topic (+6)	Apoptosis (+25)
PA2019	-	Pseudomonas aeruginosa	-	Anti-Bacterial Agents (+4)	Electrophoretic Mobility Shift Assay	Promoter Regions, Genetic (+4)
PA4050	-	-	-	-	-	-

**Table 4:** **GO and MeSH annotations for antibiotic registance** ***Pseudomonas aeruginosa*** **: comparison of NCBI gene descriptions, GO, and MeSH**

**Locus**	**MeSH**				
	**H, p=0.040**	**J, p=0.021**	**L, p=1.5E-7**	**M, p=0.36**	**N, p=0.061**
PA3303	-	-	-	-	-
PA0392	-	-	-	-	-
PA4077	-	-	-	-	-
PA5199	Systems Biology (+1)	-	Computer Simulation (+3)	-	Temperature (+2)
PA5366	Photochemistry	-	Phylogeny (+3)	-	-
PA5200	Proteomics (+3)	Polystyrene (+1)	Base Sequence (+5)	-	Biomass (+3)
PA0016	Genetics, Microbial (+1)	Membranes, Artificial	Amino Acid Sequence (+3)	-	Sensitivity and Specificity (+1)
PA4942	-	-	Databases, Protein (+4)	-	Ultraviolet Rays (+2)
PA3014	-	Polyesters	Software (+4)	-	Temperature
PA4398	-	-	-	-	-
PA5528	-	-	-	-	-
PA1805	Genomics	-	Base Sequence (+1)	-	Hot Temperature
PA3016	-	-	-	-	-
PA4223	-	-	-	-	-
PA4960	Research	-	Computers (+4)	-	Cluster Analysis (+1)
PA0374	Computational Biology	-	Phylogeny (+4)	-	Temperature
PA3013	-	-	Software (+4)	-	-
PA5471	-	-	-	-	-
PA0502	-	-	-	-	-
PA3194	Molecular Biology (+1)	-	Molecular Sequence Data (+2)	-	Biomass
PA1775	-	-	-	-	-
PA0427	Crystallography (+2)	Liposomes (+2)	Base Sequence (+2)	Infant, Newborn	Environment
PA4222	-	-	-	-	-
PA5369	-	-	-	-	-
PA2018	-	-	Molecular Sequence Data	-	-
PA5285	-	-	-	-	-
PA2604	Statistics as Topic	-	Terminology as Topic (+4)	-	Statistics as Topic
PA2019	-	-	-	-	-
PA4050	-	-	-	-	-

## Conclusions

As shown in the case-studies described above, our framework can be a powerful tool for genome-wide studies. Due to the large number of categories included in MeSH, different interpretations are possible without a restriction to molecular biological terms. The MeSH ORA framework detects enriched MeSH terms from gene lists. Such results can seamlessly be converted into related PubMed documents. This function may support those researchers who spend much time searching through related literature, allowing them to concentrate on the biological interpretation involeved in the downstream steps.

We will also implement GSEA (gene set enrichment analysis) [70], SEA (simpler enrichment analysis) [71], or GSA (gene-set analysis) [72] functions using MeSH. In addition, the plot function will be implemented to visualize the results as a tagcloud, tree, or network mapped from enriched MeSH terms. All data will be semi-annually updated in every Bioconductor version. The data from MeSH and PubMed will be annually downloaded and the analysis of RBBH will also be performed regularly.

All R scripts used in this paper are provided in as Additional file 4.

## Availability and requirements

- **Project name**: MeSH ORA Framework
- **URLs**:
  - http://www.bioconductor.org/packages/release/data/annotation/html/MeSH.db.html
  - http://www.bioconductor.org/packages/release/data/annotation/html/MeSH.AOR.db.html
  - http://www.bioconductor.org/packages/release/data/annotation/html/MeSH.PCR.db.html
  - http://www.bioconductor.org/packages/release/data/annotation/html/org.MeSH.Hsa.db.html
  - http://bioconductor.org/packages/release/bioc/html/meshr.html
  - http://bioconductor.org/packages/release/bioc/html/MeSHDbi.html
- **Operating system**: Platform independent
- **Programming language**: R v 3.1.0 or higher
- **Other requirements**: Bioconductor 2.14 or higher (save.abst function will be released by Bioconductor 3.1). Please also note that *org.MeSH.XXX.db*-type packages are renamed as *MeSH.XXX.eg.db*-type packages by Bioconductor 3.1.
- **License**: Artistic-2.0
- **Any restrictions to use by non-academics**: For non-profit use only

## Additional files

Additional file 1
**Summary of all organisms in each MeSH category.**

Additional file 2
**Precise coverage of MeSH against all genes of 115 organisms.**

Additional file 3
**Complete data for Tables** 2 **,** 3 **and** 4 **.**

Additional file 4
**All R scripts and packages used in this paper.**
