# Supplementary material for: MeSH ORA framework: R/Bioconductor packages to support MeSH over-representation analysis
Source: BMC Bioinformatics. 2015 Feb 15;16:45. doi: 10.1186/s12859-015-0453-z (PMC4343279; doi:10.1186/s12859-015-0453-z)

A category is "**Anatomy**", which contains Animal Structures, Bacterial Structures, Body Regions, Cardiovascular System, Cells,Digestive System, Embryonic Structures, Endocrine System, Fluids and Secretions, Fungal Structures, Hemic and Immune Systems, Integumentary System, Musculoskeletal System, Nervous System, Plant Structures, Respiratory System, Sense Organs, Stomatognathic System, Tissues, Peritoneal Stomata, Urogenital System, and Viral Structures [*Anatomy* (NCBI)].

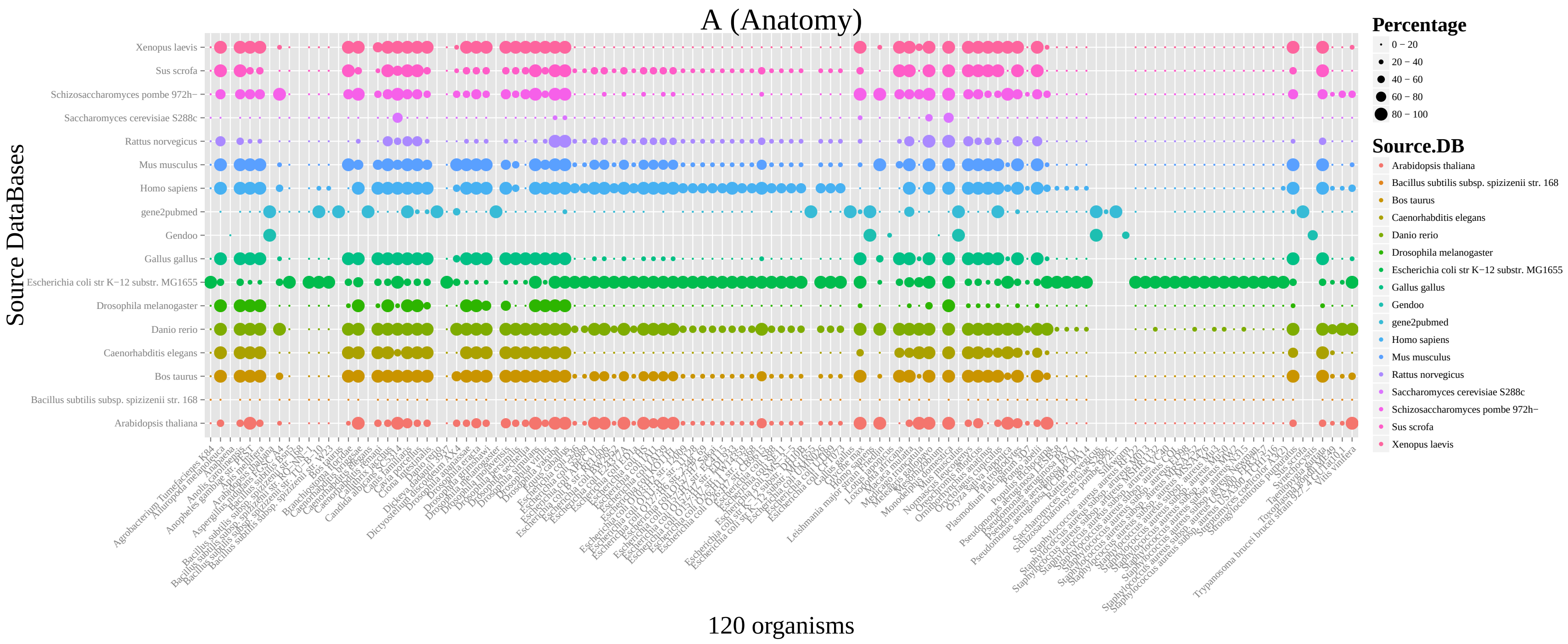

B category is "**Organisms**", which contains Archaea, Bacteria, Eukaryota, Organisms Form, and Viruses [*Organisms* (NCBI)].

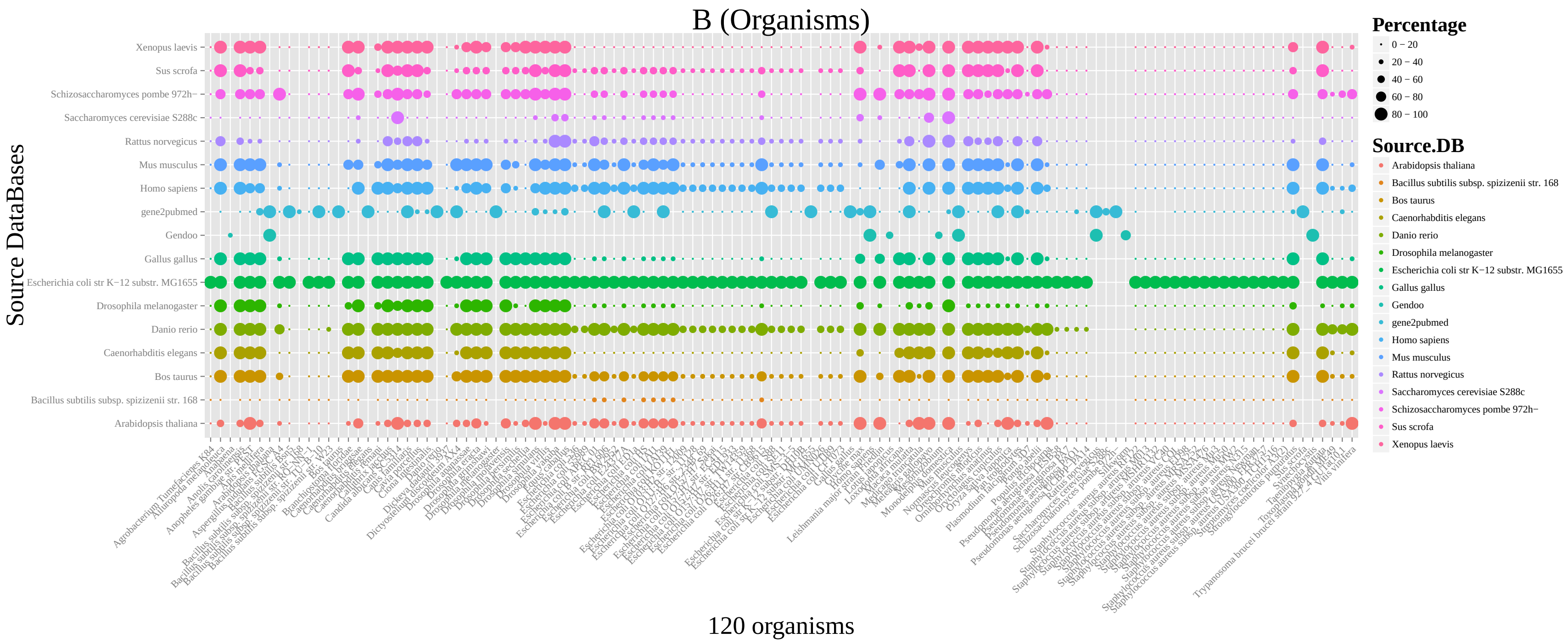

C category is "**Diseases**", which contains Animal Diseases, Bacterial Infections and Mycoses, Cardiovascular Diseases, Chemically-Induced Disorders, Congenital, Hereditary, Digestive System Diseases, Disorders of Environmental Origin, Endocrine System Diseases, Eye Diseases, Female Urogenital Diseases and Pregnancy Complications, Hemic and Lymphatic Diseases, Immune System Diseases, Male Urogenital Diseases, Musculoskeletal Diseases, Neoplasms, Nervous System Diseases, Nutritional and Metabolic Diseases, Occupational Diseases, Otorhinolaryngologic Diseases, Parasitic Diseases, Pathological Conditions, Signs and Symptoms, Respiratory Tract Diseases, Skin and Connective Tissue Diseases, Stomatognathic Diseases, Virus Diseases, and Wounds and Injuries [*Diseases* (NCBI)].

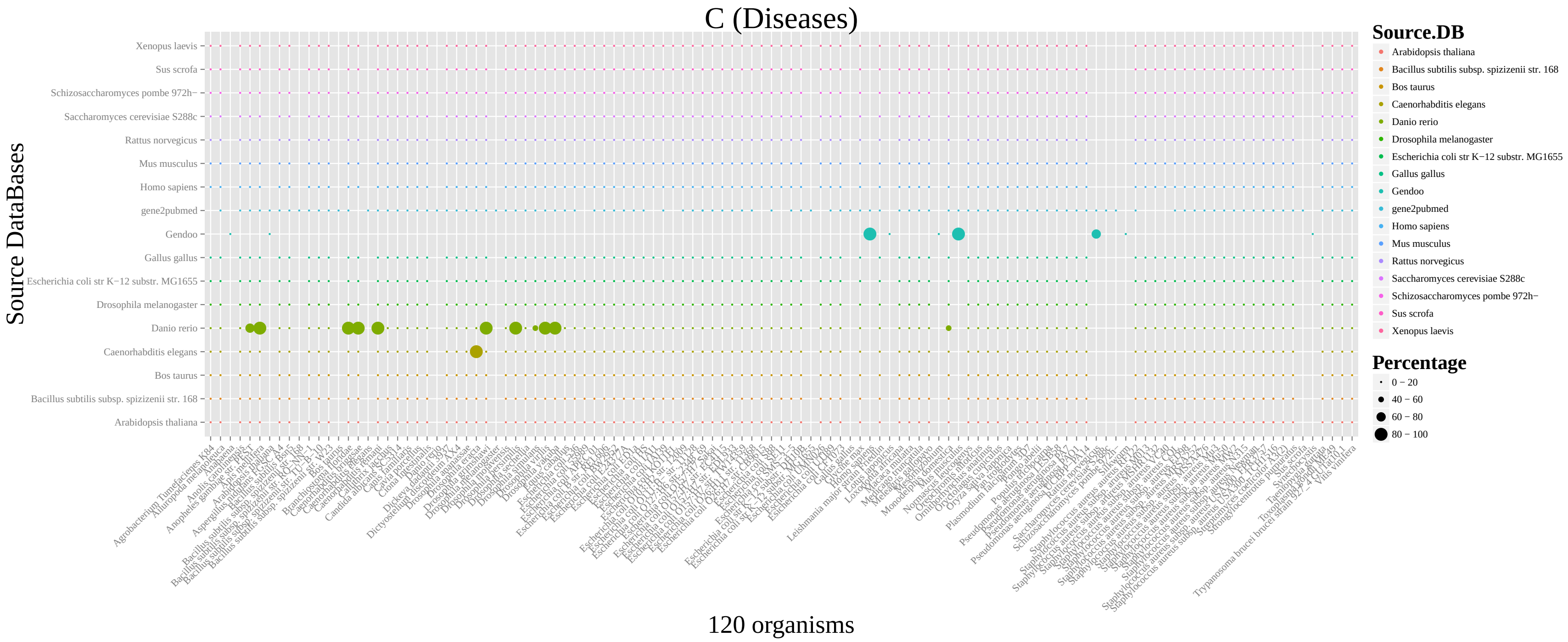

D category is “**Chemicals and Drugs**”, which contains Amino Acids, Peptides, and Proteins, Biological Factors, Biomedical and Dental Materials, Carbohydrates, Chemical Actions and Uses, Complex Mixtures, Enzymes and Coenzymes, Heterocyclic Compounds, Hormones, Hormone Substitutes, and Hormone Antagonists, Inorganic Chemicals, Lipids, Macromolecular Substances, Nucleic Acids, Nucleotides, and Nucleosides, Organic Chemicals, and Polycyclic Compounds [*Chemicals and Drugs* (NCBI)].

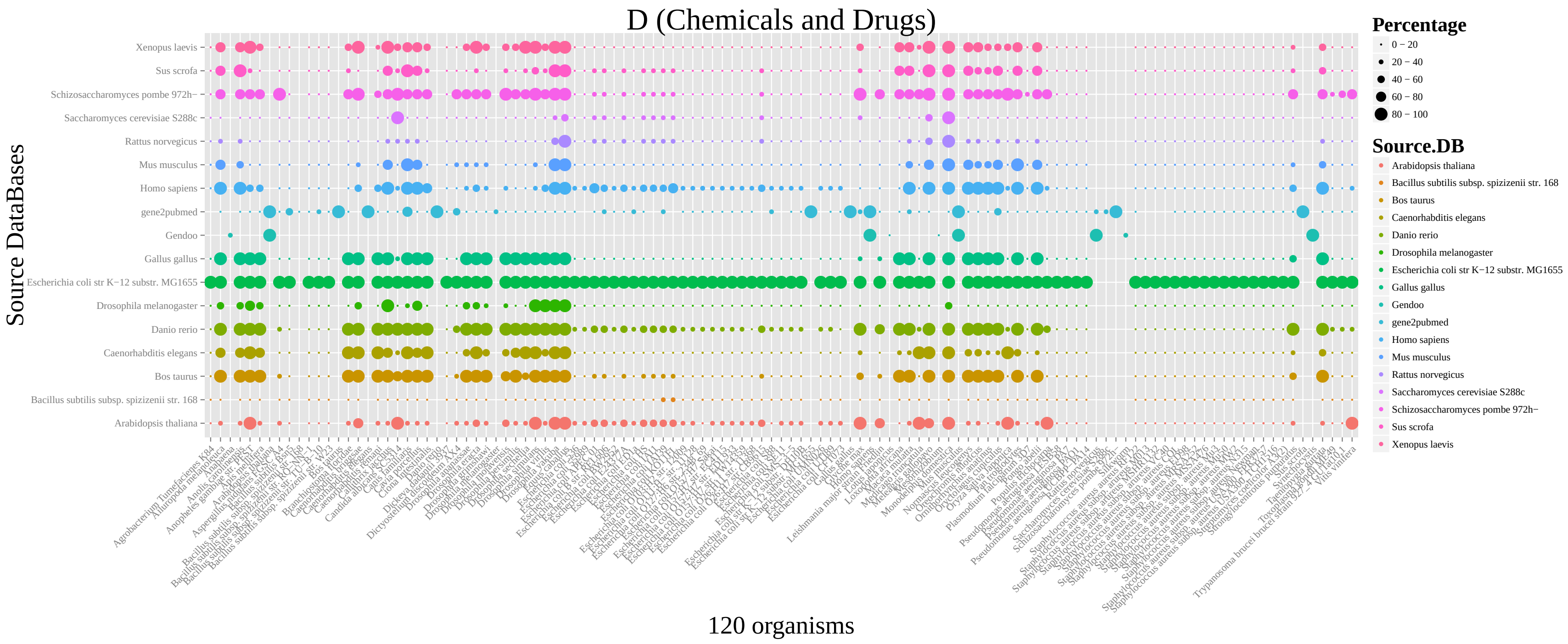

E category is “**Analytical Diagnostic and Therapeutic Techniques and Equipment**”, which contains Anesthesia and Analgesia, Dentistry, Diagnosis, Equipment and Supplies, Investigative Techniques, Surgical Procedures, Operative, and Therapeutics [*Analytical Diagnostic and Therapeutic Techniques and Equipment* (NCBI)].

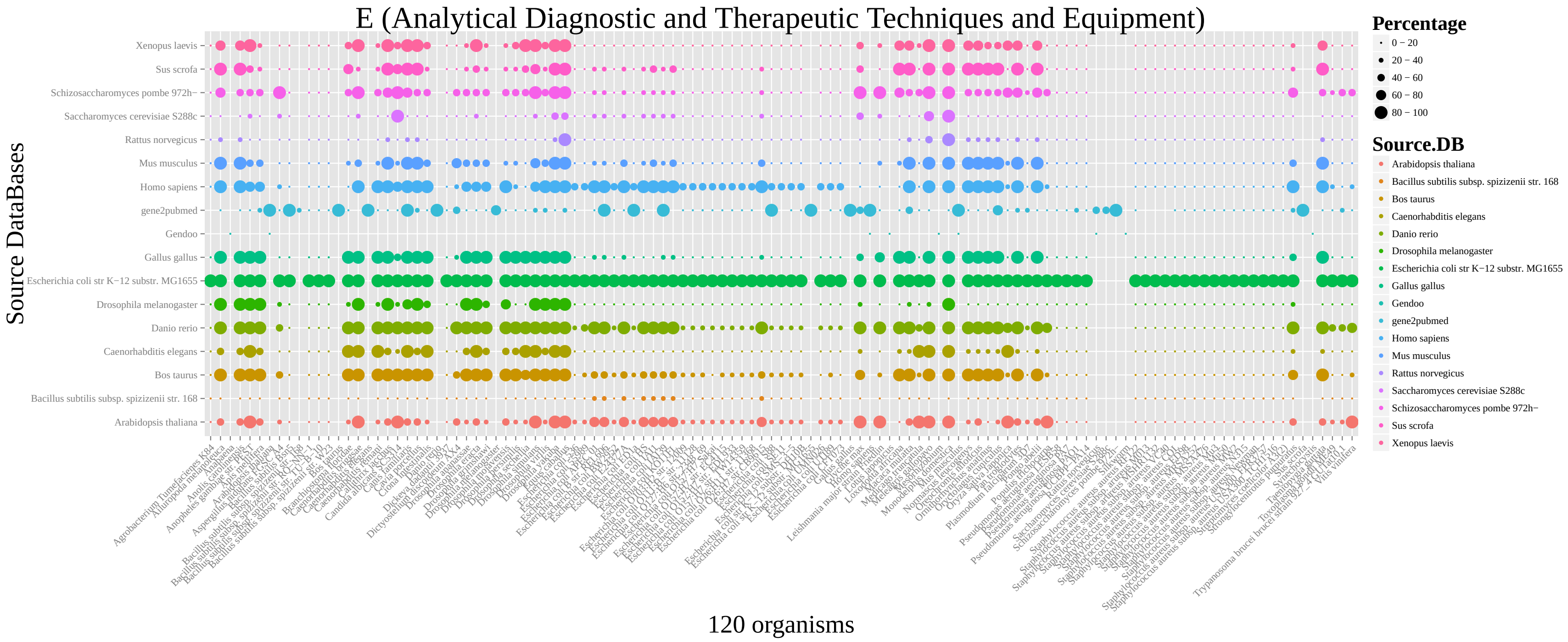

F category is “**Psychiatry and Psychology**”, which contains Behavior and Behavior Mechanisms, Behavioral Disciplines and Activities, Mental Disorders, and Psychological Phenomena and Processes [*Psychiatry and Psychology* (NCBI)].

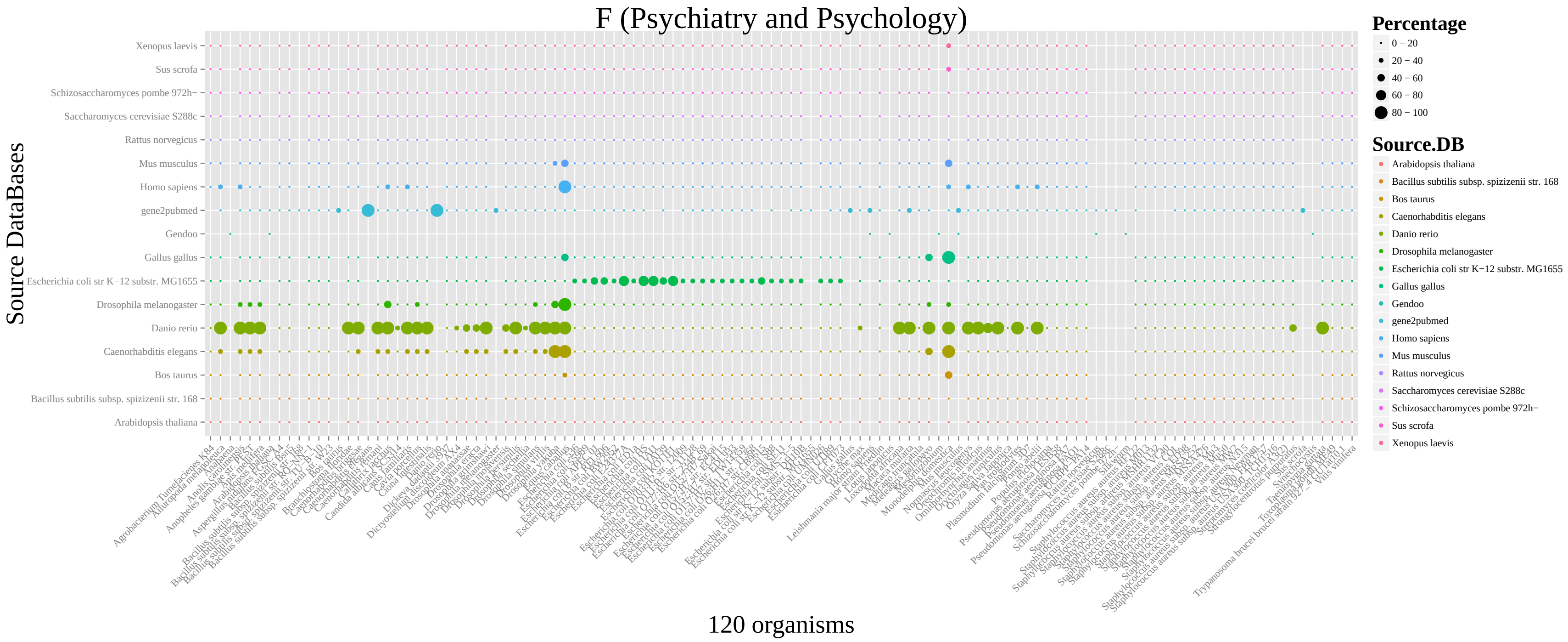

G category is "**Phenomena and Processes**", which contains Biological Phenomena, Cell Physiological Phenomena, Chemical Processes, Circulatory and Respiratory Physiological Phenomena, Digestive System and Oral Physiological Phenomena, Genetic Phenomena, Immune System Phenomena, Integumentary System Physiological Phenomena, Mathematical Concepts, Metabolic Phenomena, Microbiological Phenomena, Musculoskeletal and Neural Physiological Phenomena, Ocular Physiological Phenomena, Physical Phenomena, Physiological Phenomena, Plant Physiological Phenomena, and Reproductive and Urinary Physiological Phenomena [*Phenomena and Processes* (NCBI)].

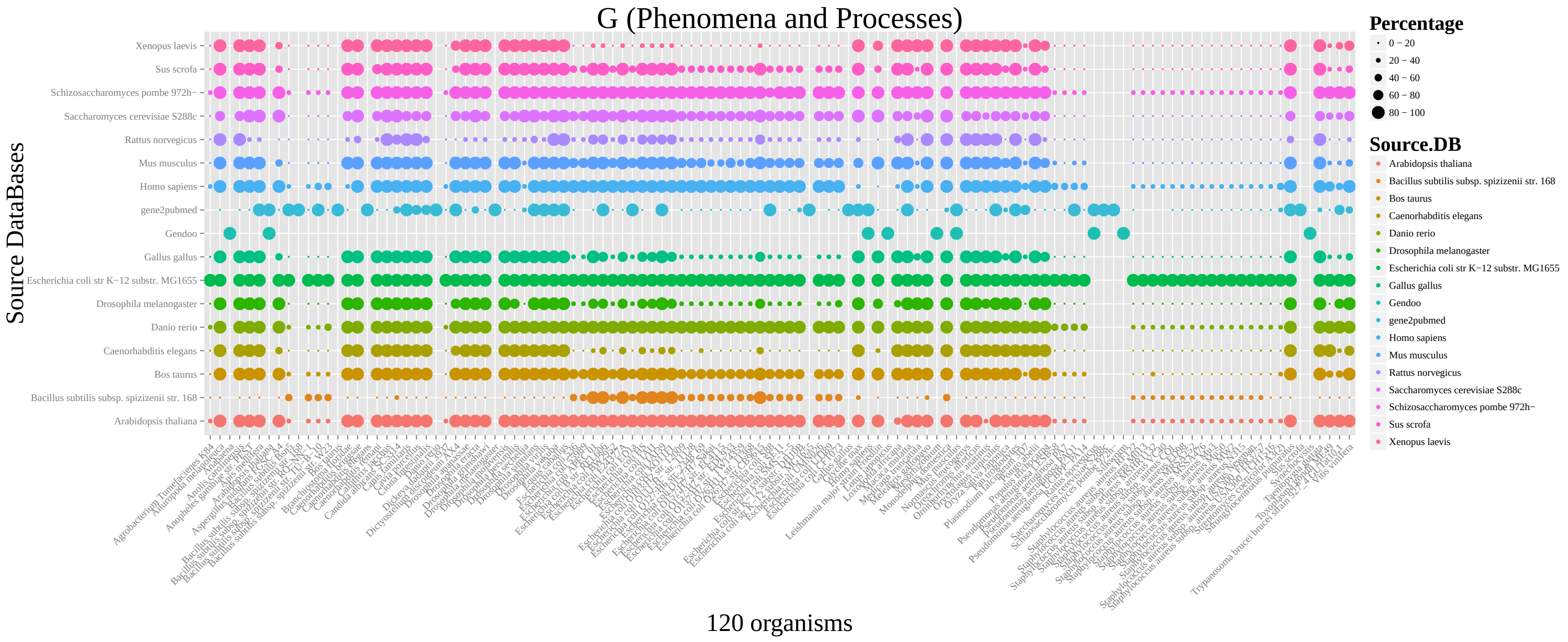

H category is "**Disciplines and Occupations**", which contains Health Occupations, and Natural Science Disciplines [*Disciplines and Occupations* (NCBI)].

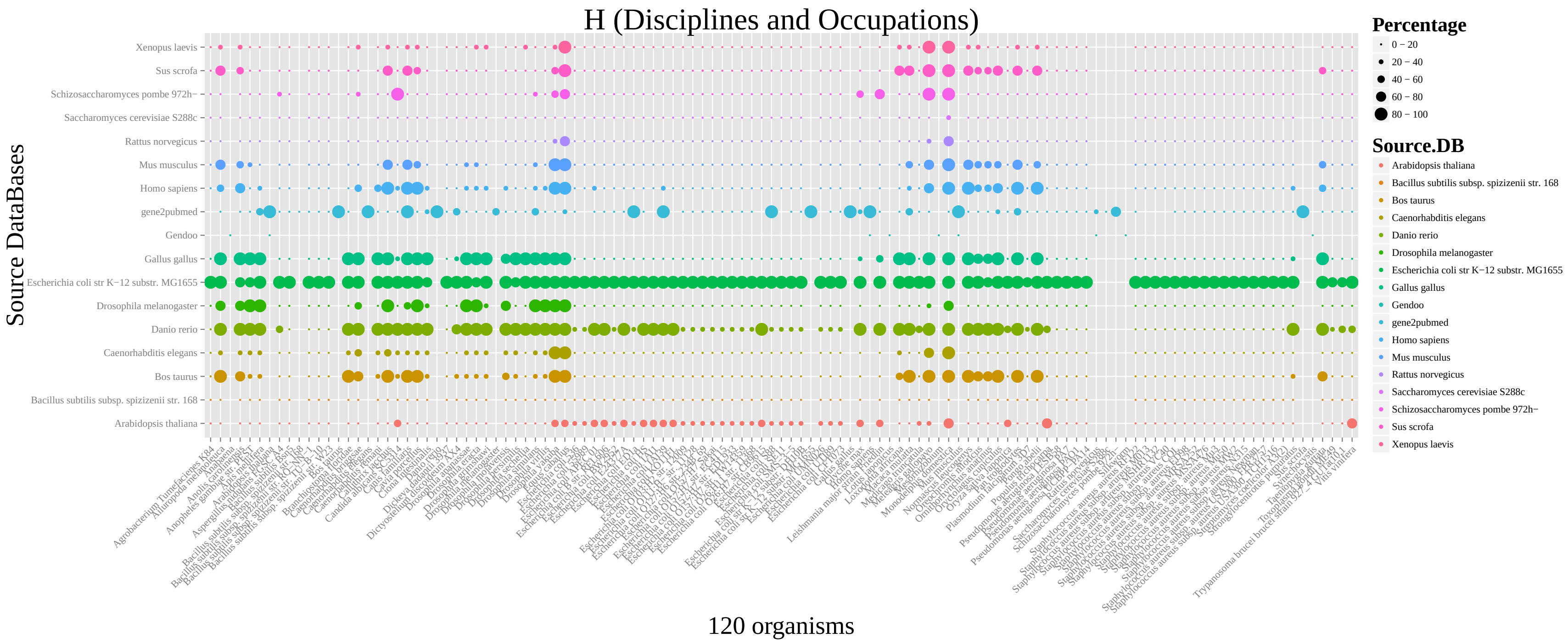

I category is "**Anthropology**, **Education**, **Sociology and Social Phenomena**", which contains Education, Human Activities, and Social Sciences [*Anthropology*, *Education*, *Sociology and Social Phenomena* (NCBI)].

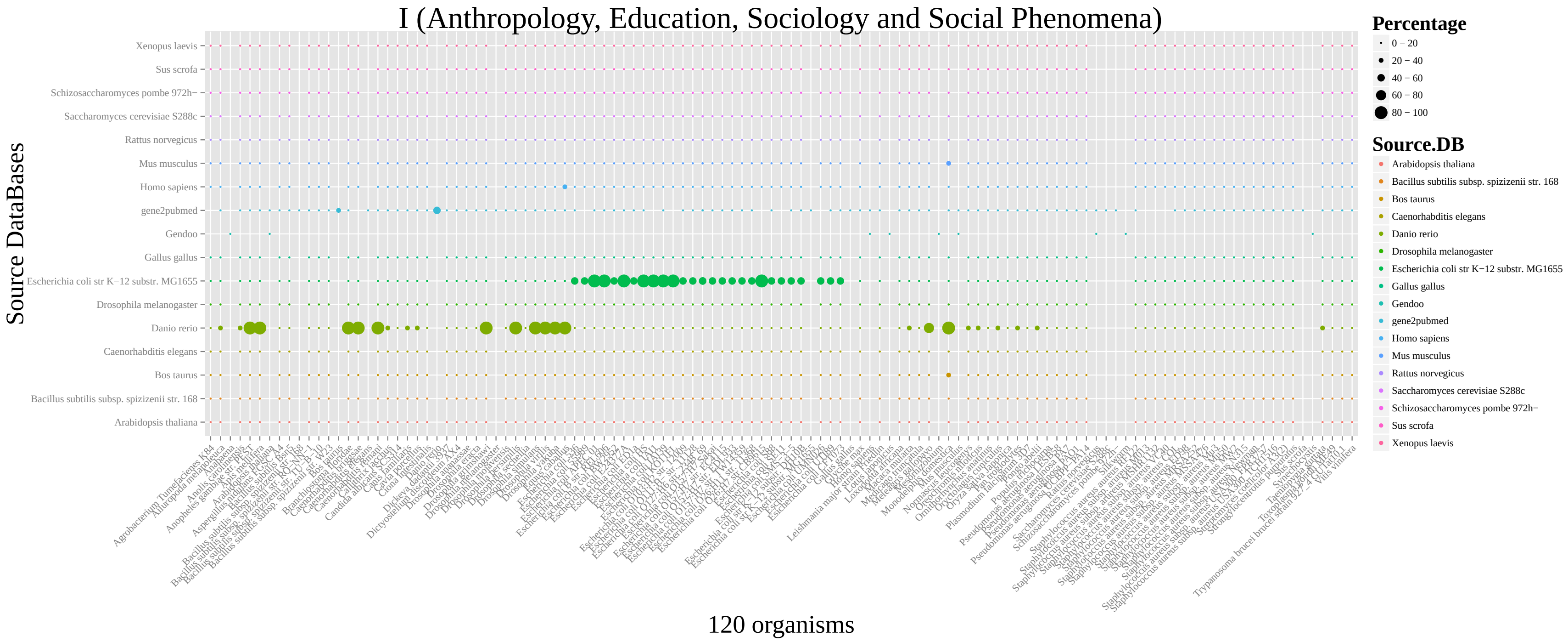

J category is "**Technology and Food and Beverages**", which contains Food and Beverages and Technology, Undustry and Agriculture [*Technology and Food and Beverages* (NCBI)].

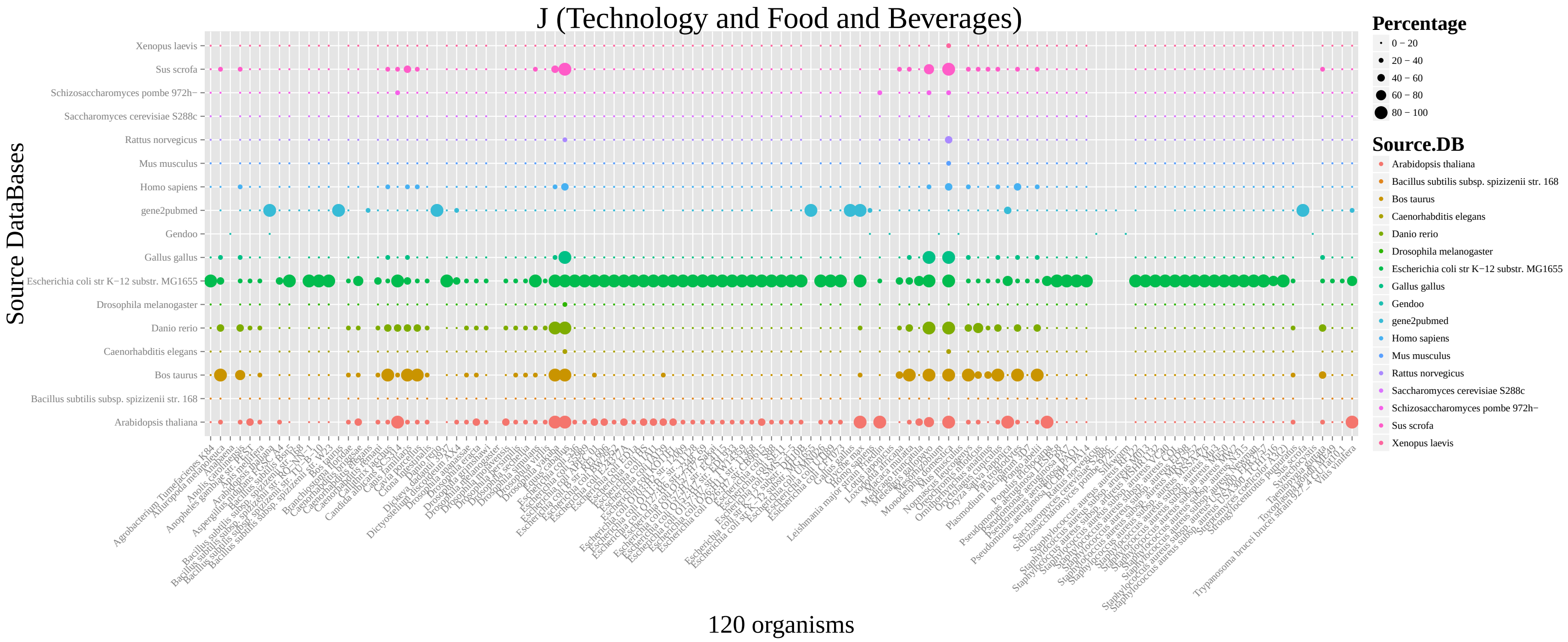

K category is “**Humanities**”, which contains Art, Awards and Prizes, History, Knowledge, Literature, Morals, Music, Nature, Occultism, Philosophy, Religion, and Secularism [*Humanities* (NCBI)].

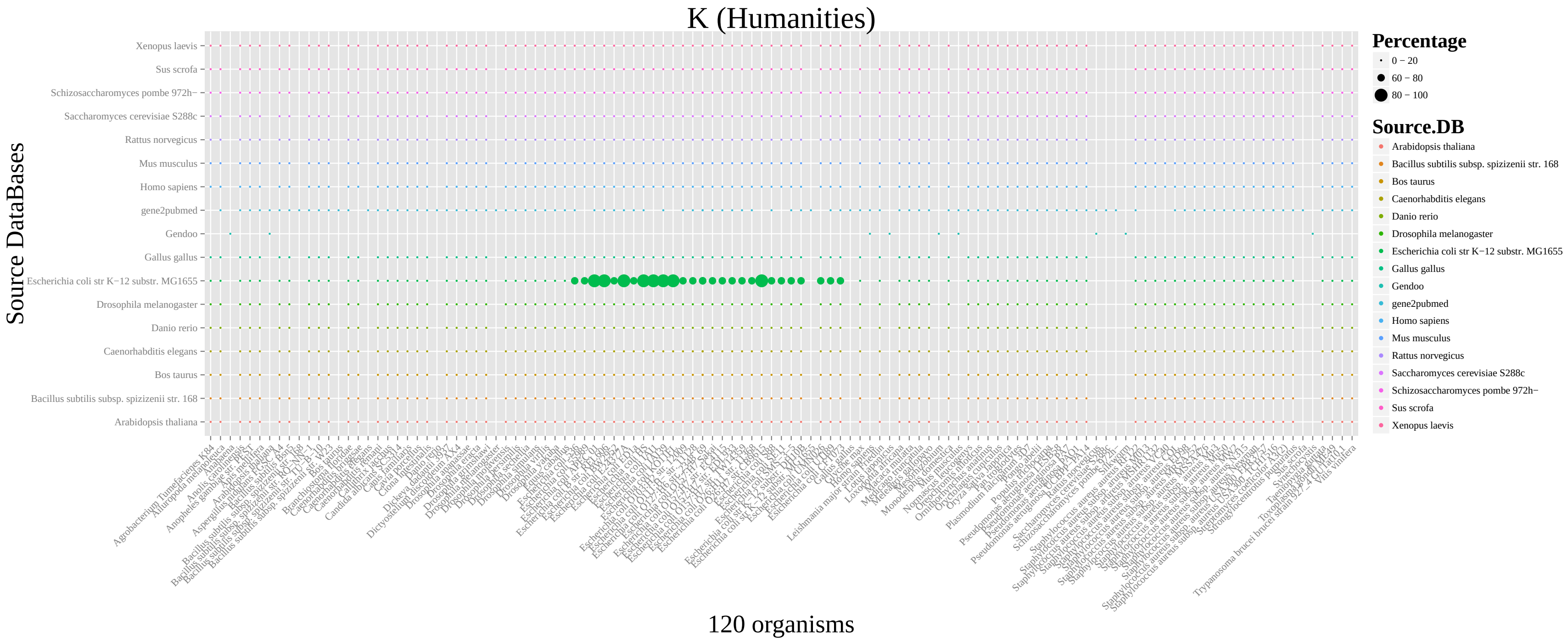

L category is "**Information Science**", which contains Book Collecting, Chronology as Topic, Classification, Communication, Communications Media, Computer Security, Computing Methodologies, Copying Processes, Data Collection, Data Display, Informatics, Information Centers, Information Management, Information Services, Information Storage and Retrieval, Information Theory, Knowledge, Library Science, Medical Informatics, Pattern Recognition, Automated, Publishing, Systems Analysis [$Information\ Science$ (NCBI)].

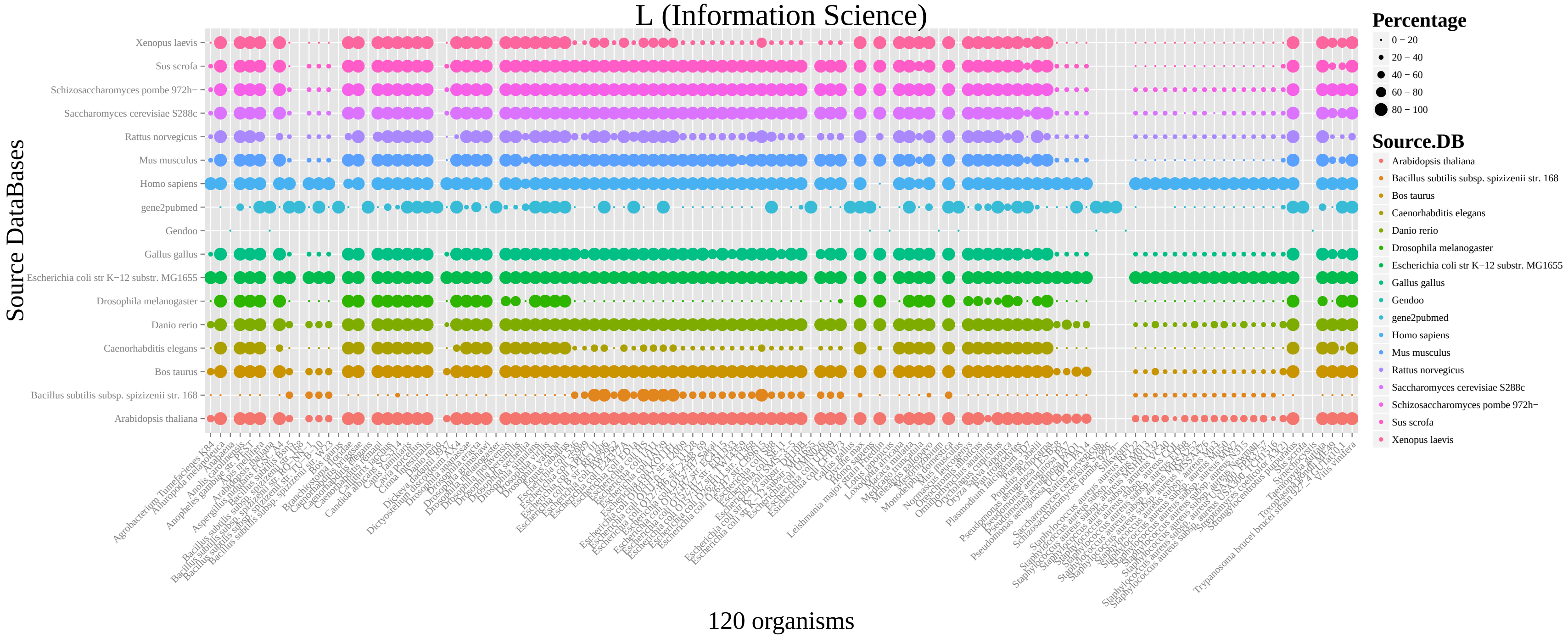

M category is "**Persons**", which contains Abortion Applicants, Adult Children, Age Groups, Alcoholics, Athletes, Caregivers, Child of Impaired Parents, Child, Abandoned, Child, Exceptional, Child, Orphaned, Child, Unwanted, Consultants, Crime Victims, Criminals, Disabled Persons, Disaster Victims, Drug Users, Emigrants and Immigrants, Famous Persons, Friends, Homebound Persons, Homeless Persons, Jehovah's Witnesses, Legal Guardians, Medically Uninsured, Men, Mentors, Minors, Multiple Birth Offspring, Occupational Groups, Parents, Patients, Population Groups, Prisoners, Refugees, Research Personnel, Research Subjects, Sex Workers, Sexual Partners, Siblings, Single Person, Slaves, Spouses, Students, Survivors, Terminally Ill, Tissue Donors, Transgendered Persons, Transients and Migrants, Veterans, Visitors to Patients, Volunteers, Vulnerable Populations, Women [*Persons* (NCBI)].

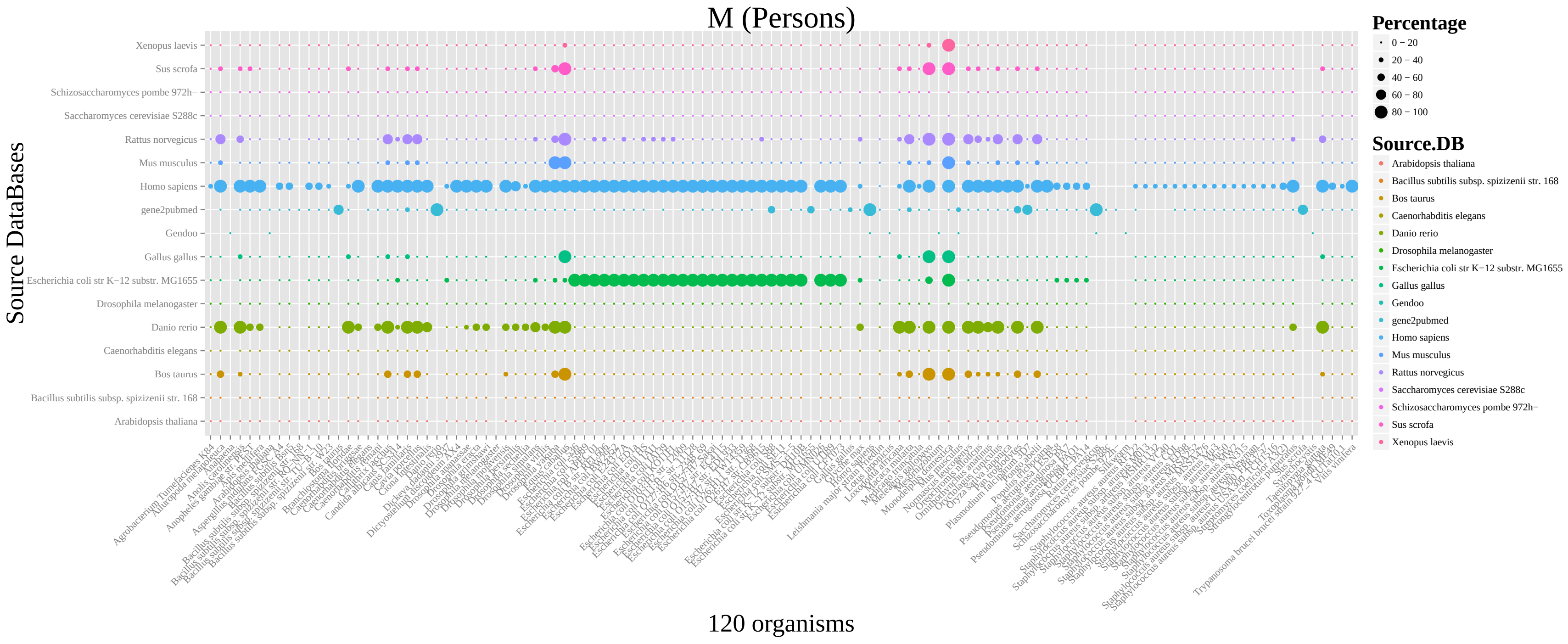

N category is "**Health Care**", which contains Environment and Public Health, Health Care Economics and Organizations, Health Care Facilities, Manpower, and Services, Health Care Quality, Access, and Evaluation, Health Services Administration, Population Characteristics [*Health Care* (NCBI)].

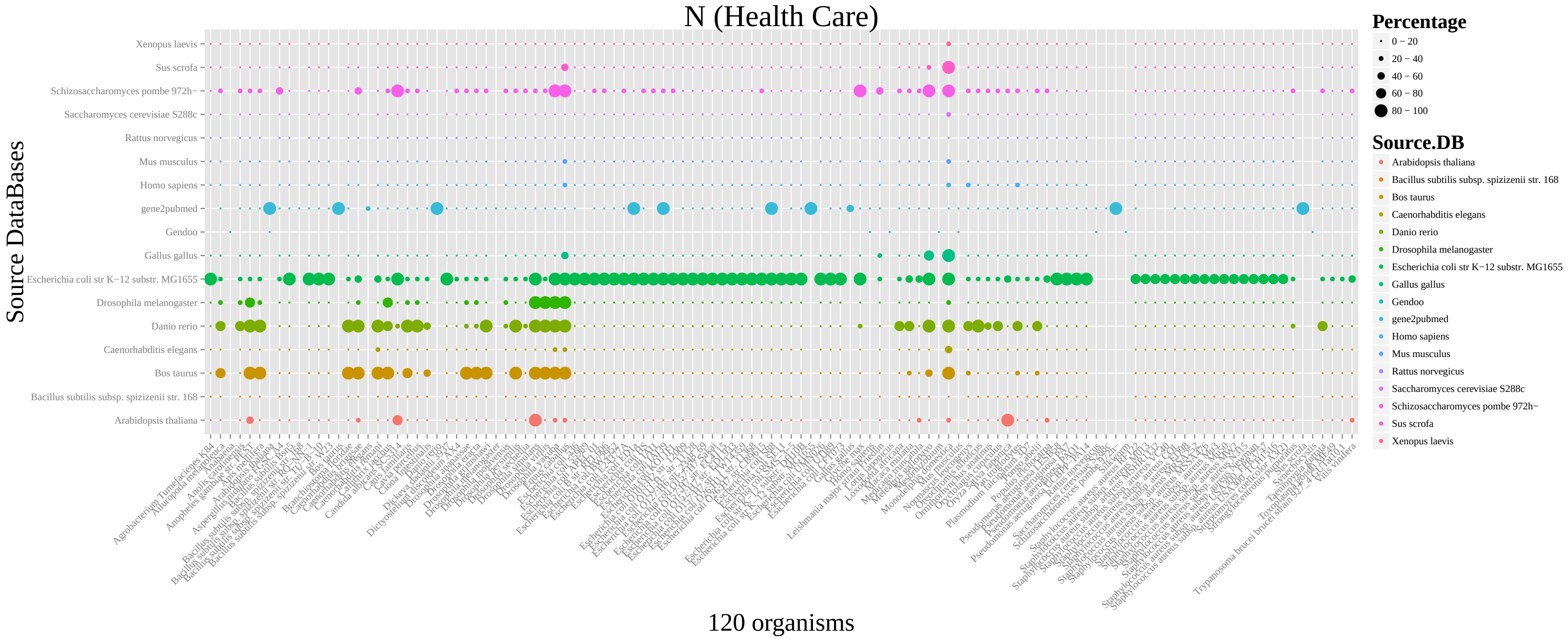

V category is "**Publication Type**", which contains Publication Components, Publication Formats, Study Characteristics, Support of Research [*Publication Type* (NCBI)].

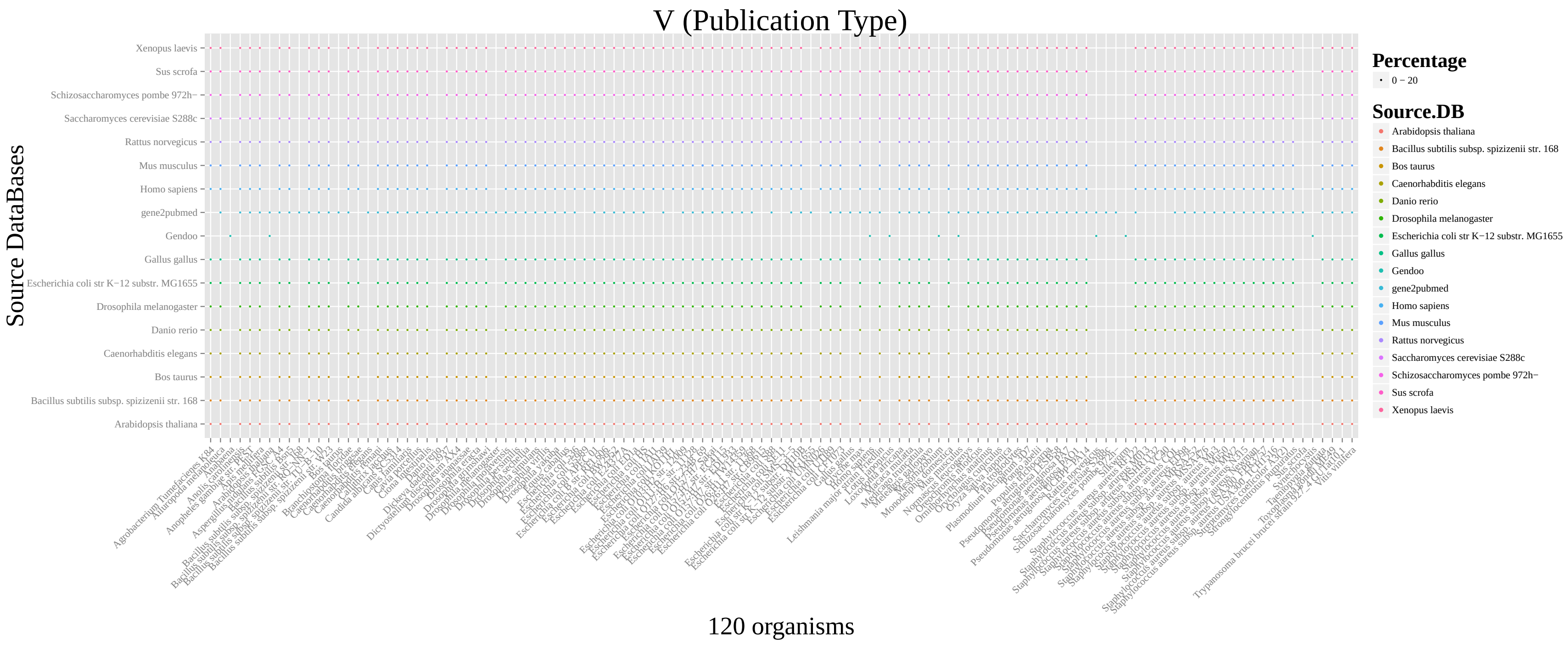

Z category is "**Geographical Locations**", which contains Africa, Americas, Antarctic Regions, Arctic Regions, Asia, Australia, Cities, Europe, Historical Geographic Locations, Islands, Oceania, Oceans and Seas [*Geographical Locations* (NCBI)].

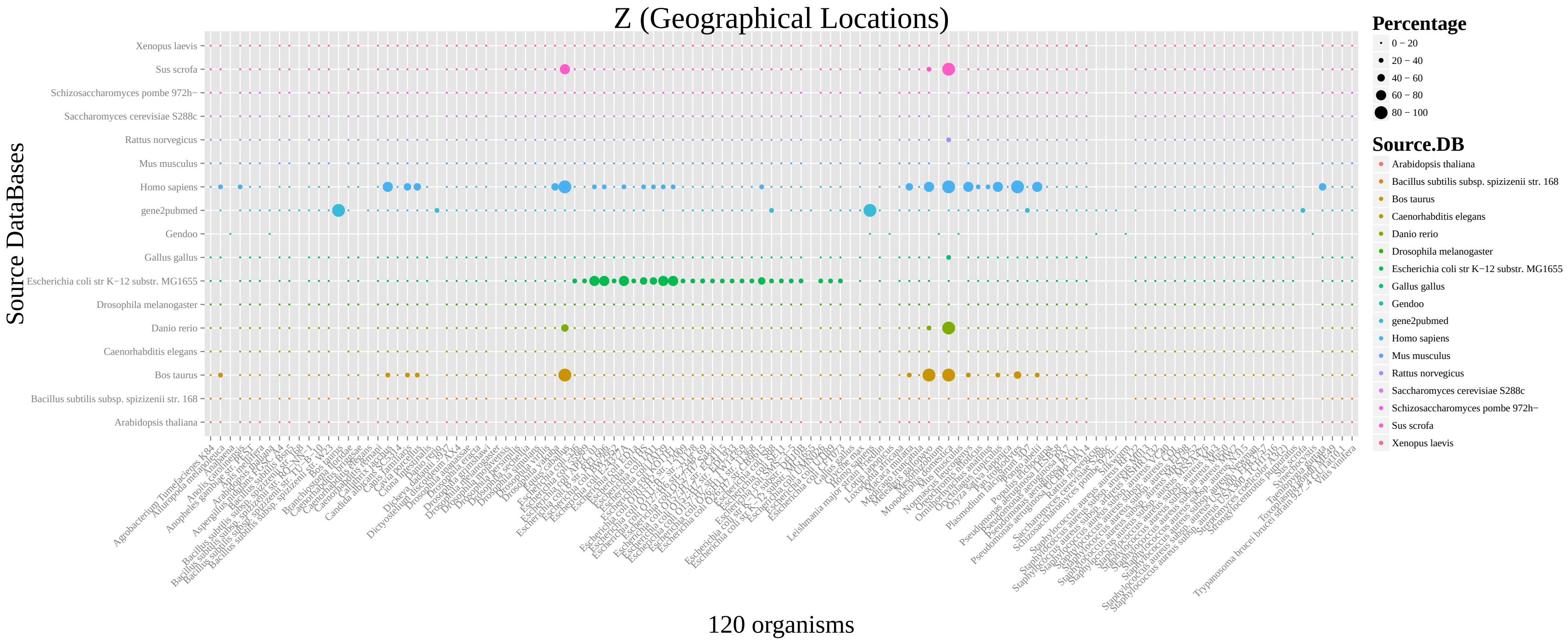

Supplement: Additional file 1 — Summary of all organisms in each MeSH category. [file 12859_2015_453_MOESM1_ESM.pdf]
