# Supplementary material for: MeSH ORA framework: R/Bioconductor packages to support MeSH over-representation analysis
Source: BMC Bioinformatics. 2015 Feb 15;16:45. doi: 10.1186/s12859-015-0453-z (PMC4343279; doi:10.1186/s12859-015-0453-z)
Supplement: Additional file 2 — Precise coverage of MeSH against all genes of 115 organisms. [file 12859_2015_453_MOESM2_ESM.pdf]

Coverage of MeSH against gene of 115 organisms

November 11, 2014

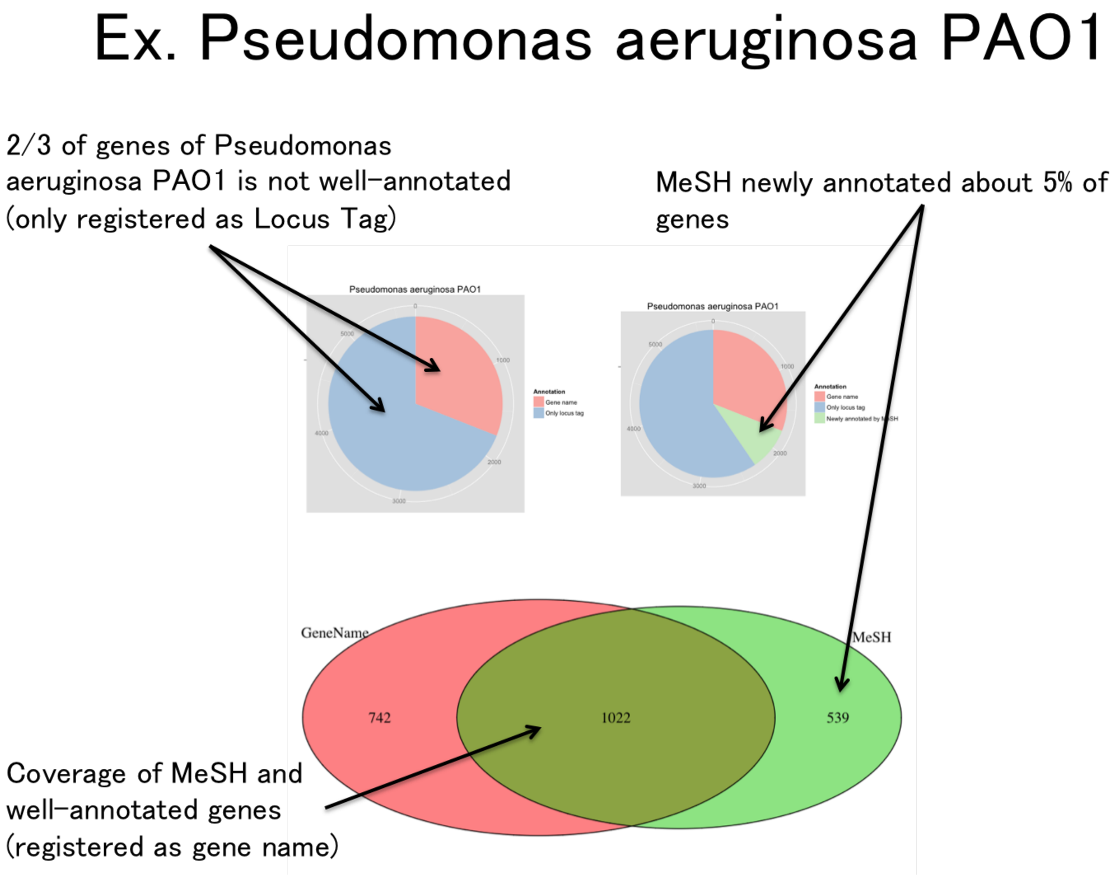

Figure 1: How to interpret these figures

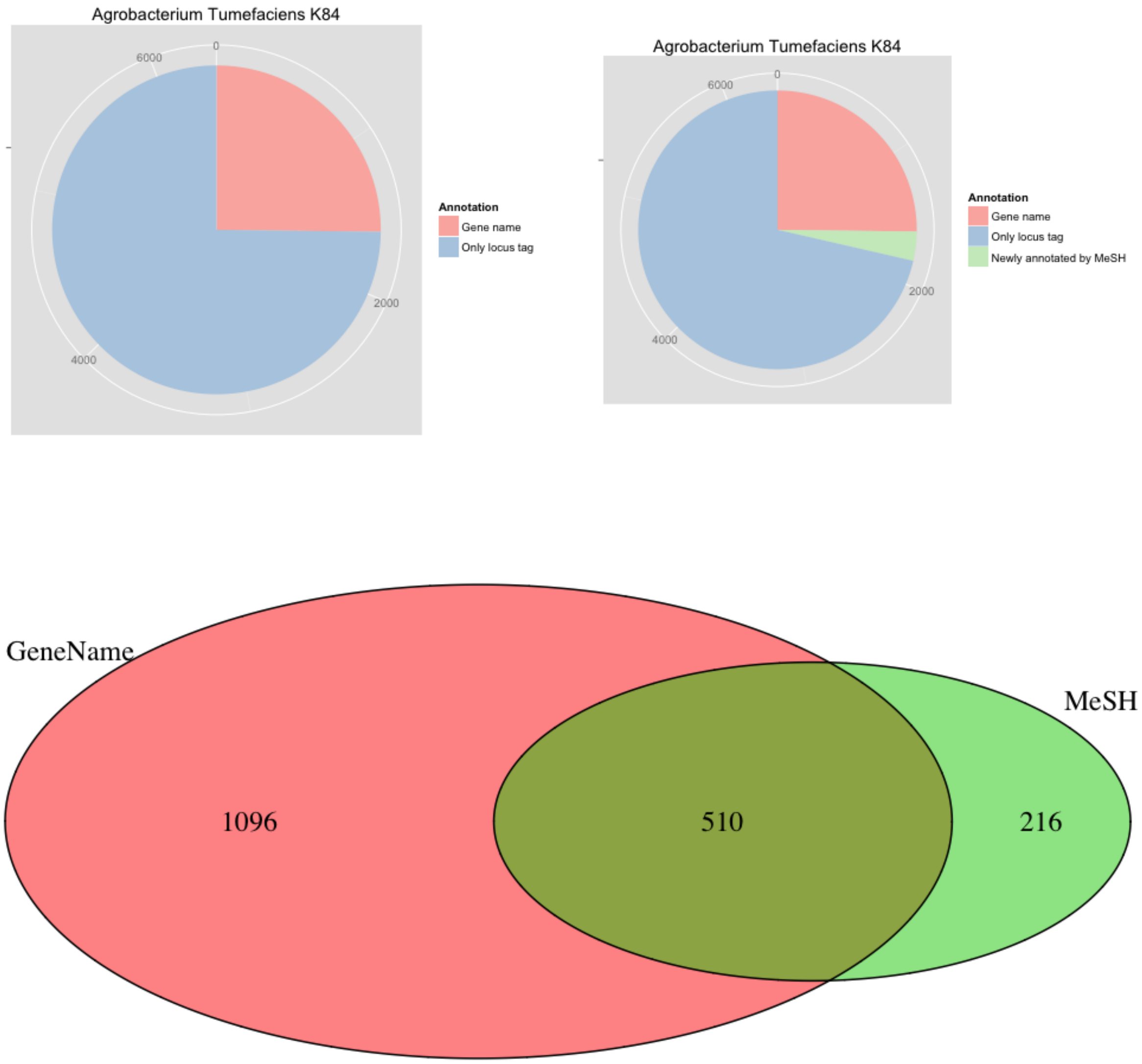

Figure 2: org.MeSH.Atu.K84.db

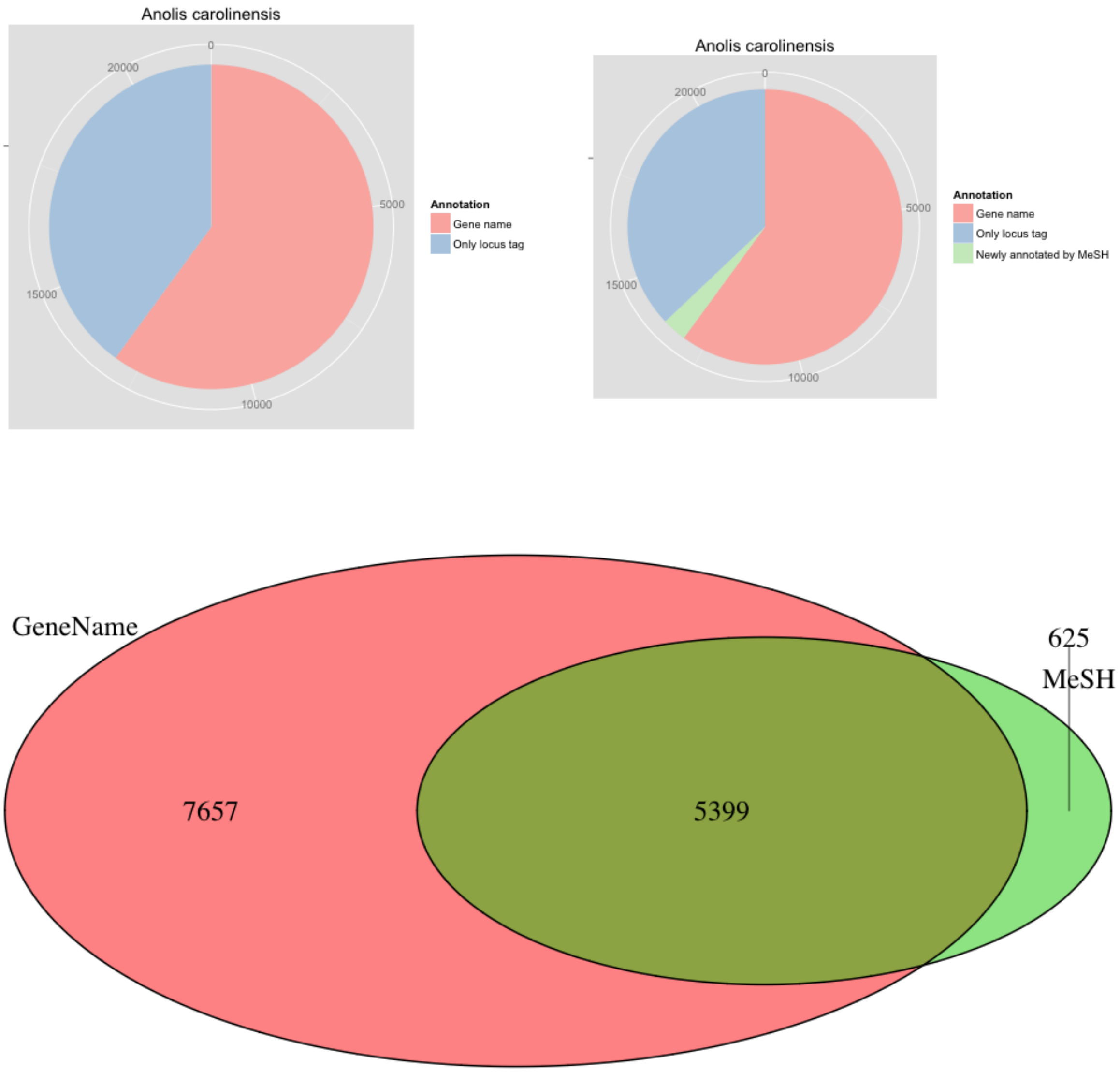

Figure 3: org.MeSH.Aca.db

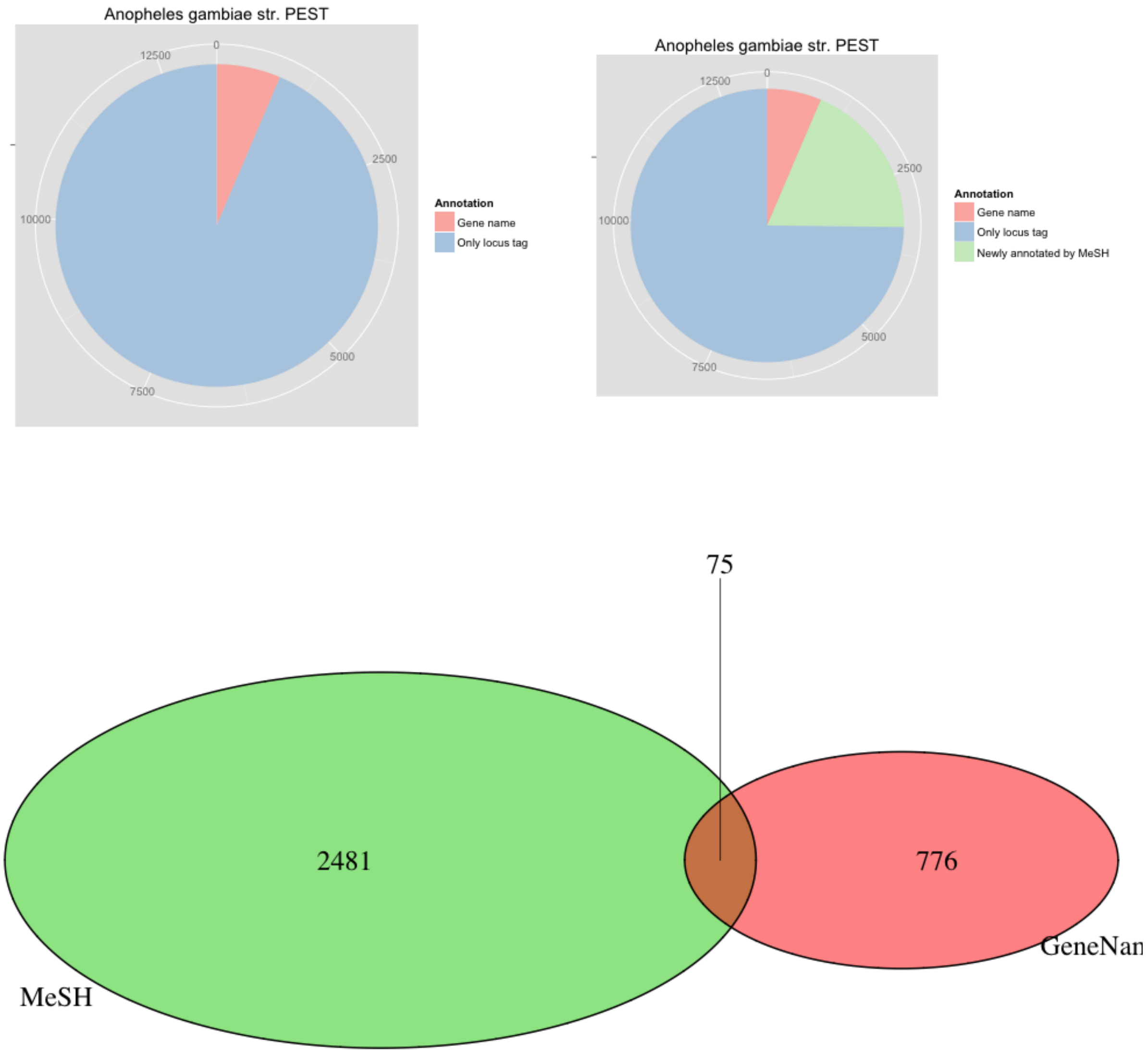

Figure 4: org.MeSH.Aga.PEST.db

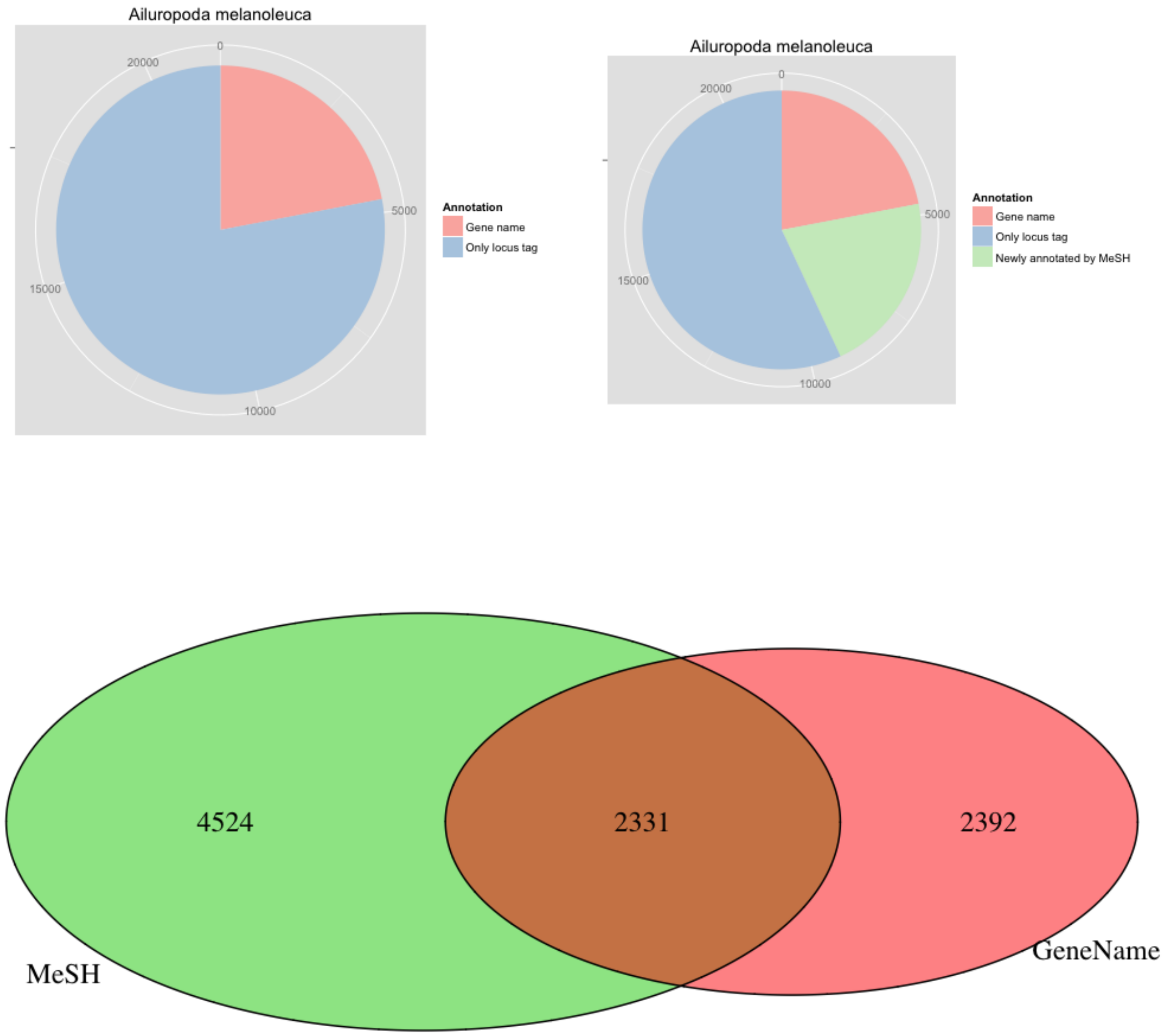

Figure 5: org.MeSH.Ame.db

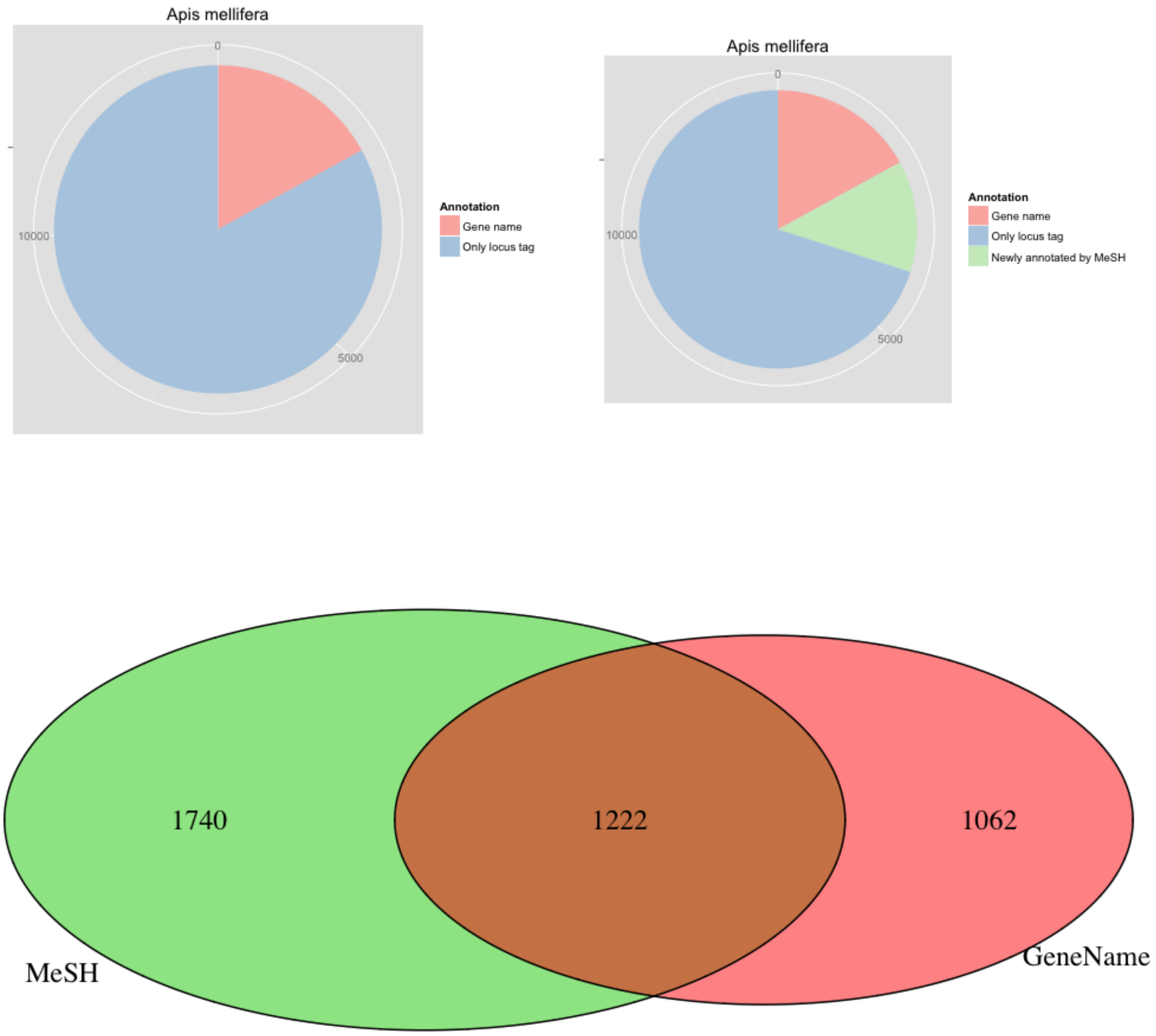

Figure 6: org.MeSH.Aml.db

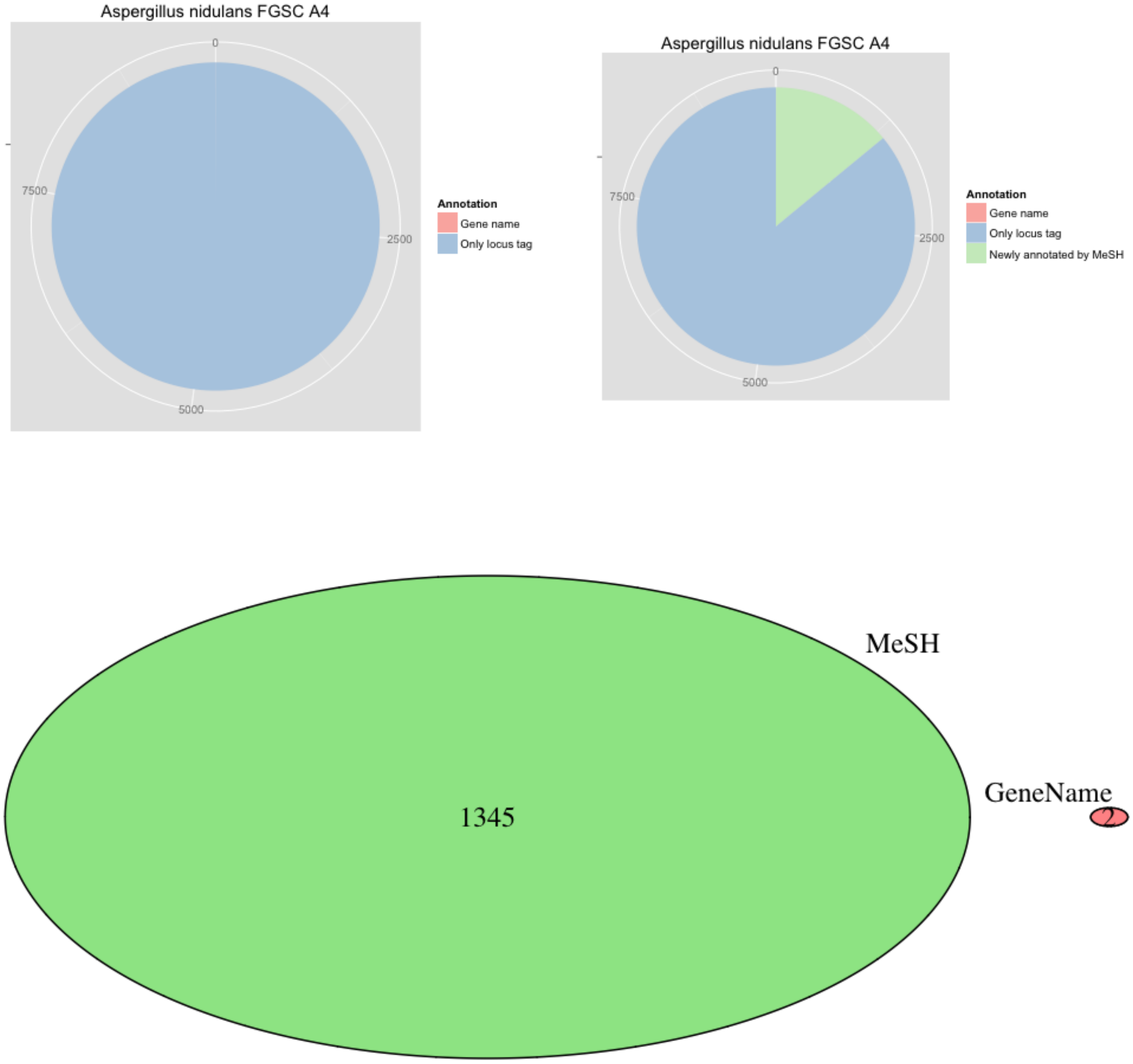

Figure 7: org.MeSH.Ani.FGSC.db

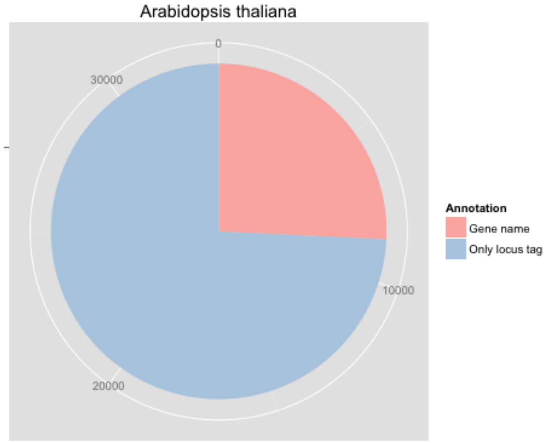

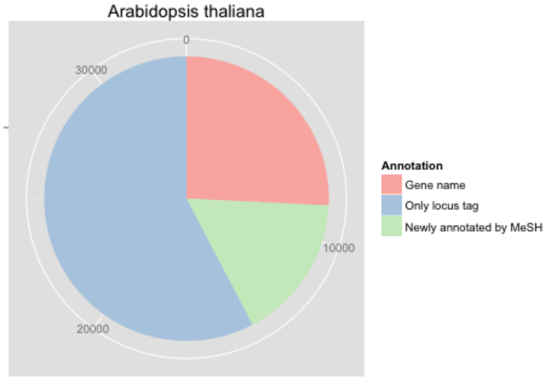

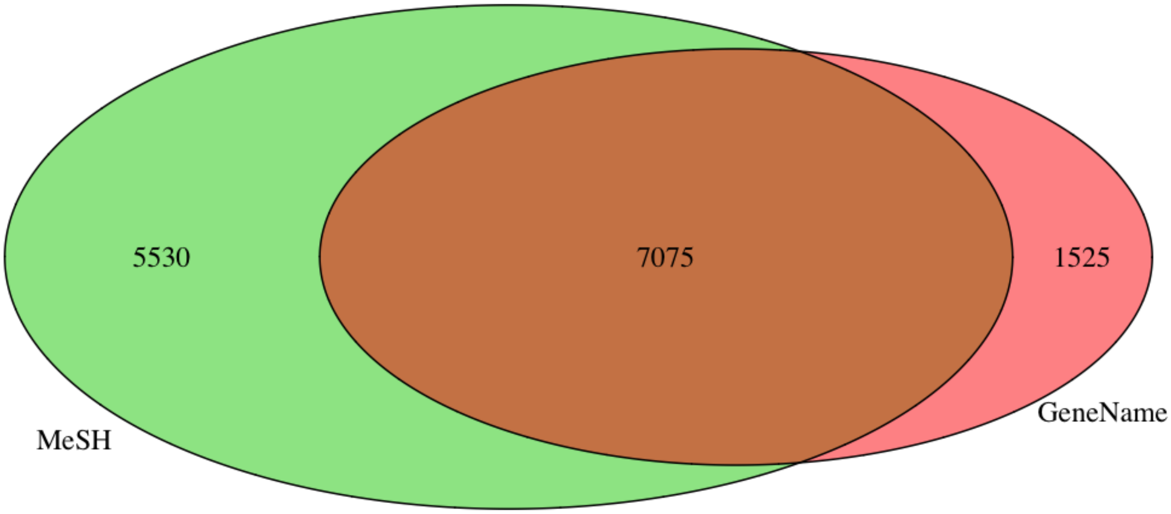

Figure 8: org.MeSH.Ath.db

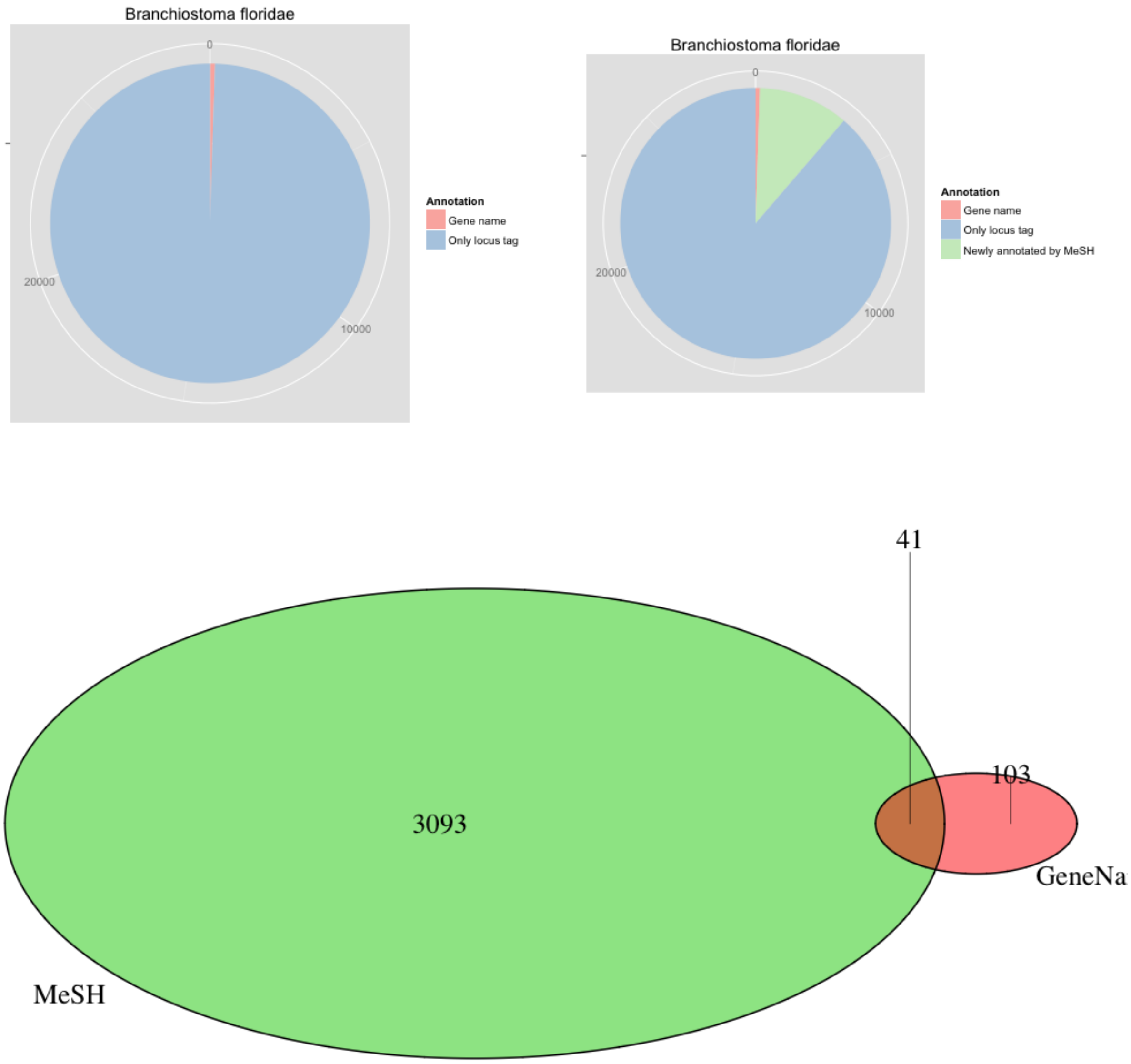

Figure 9: org.MeSH.Bfl.db

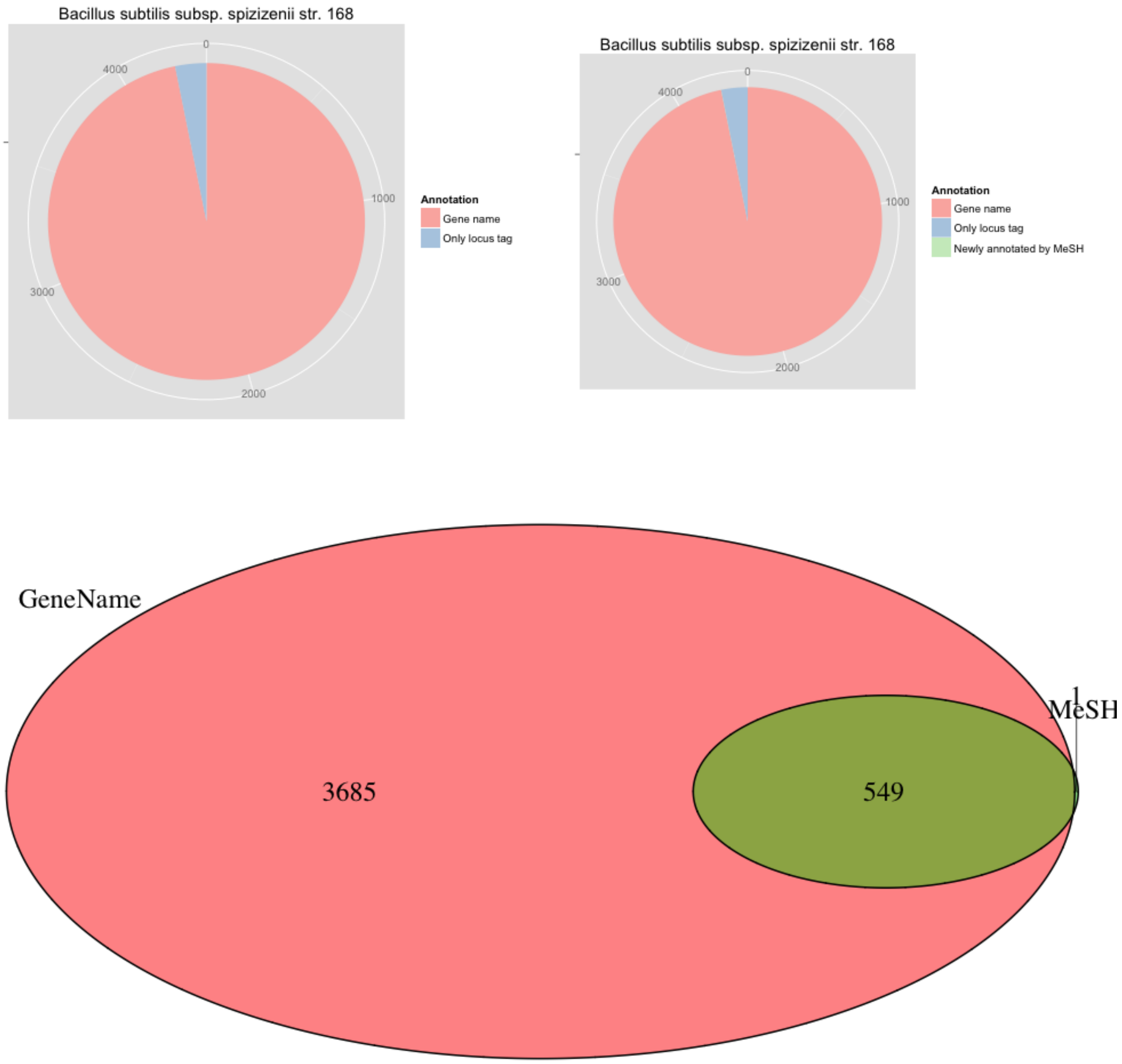

Figure 10: org.MeSH.Bsu.168.db

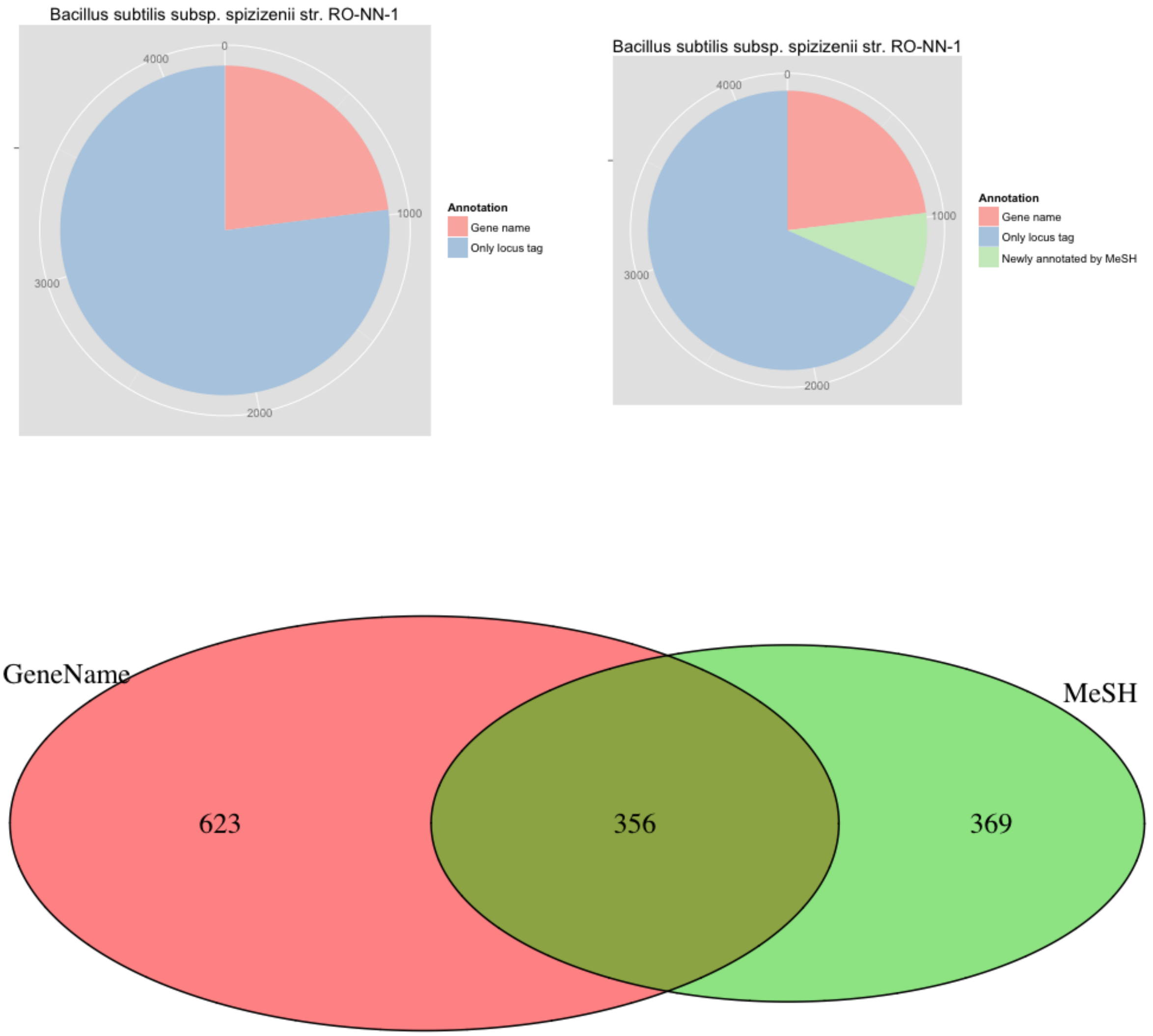

Figure 11: org.MeSH.Bsu.RONN1.db

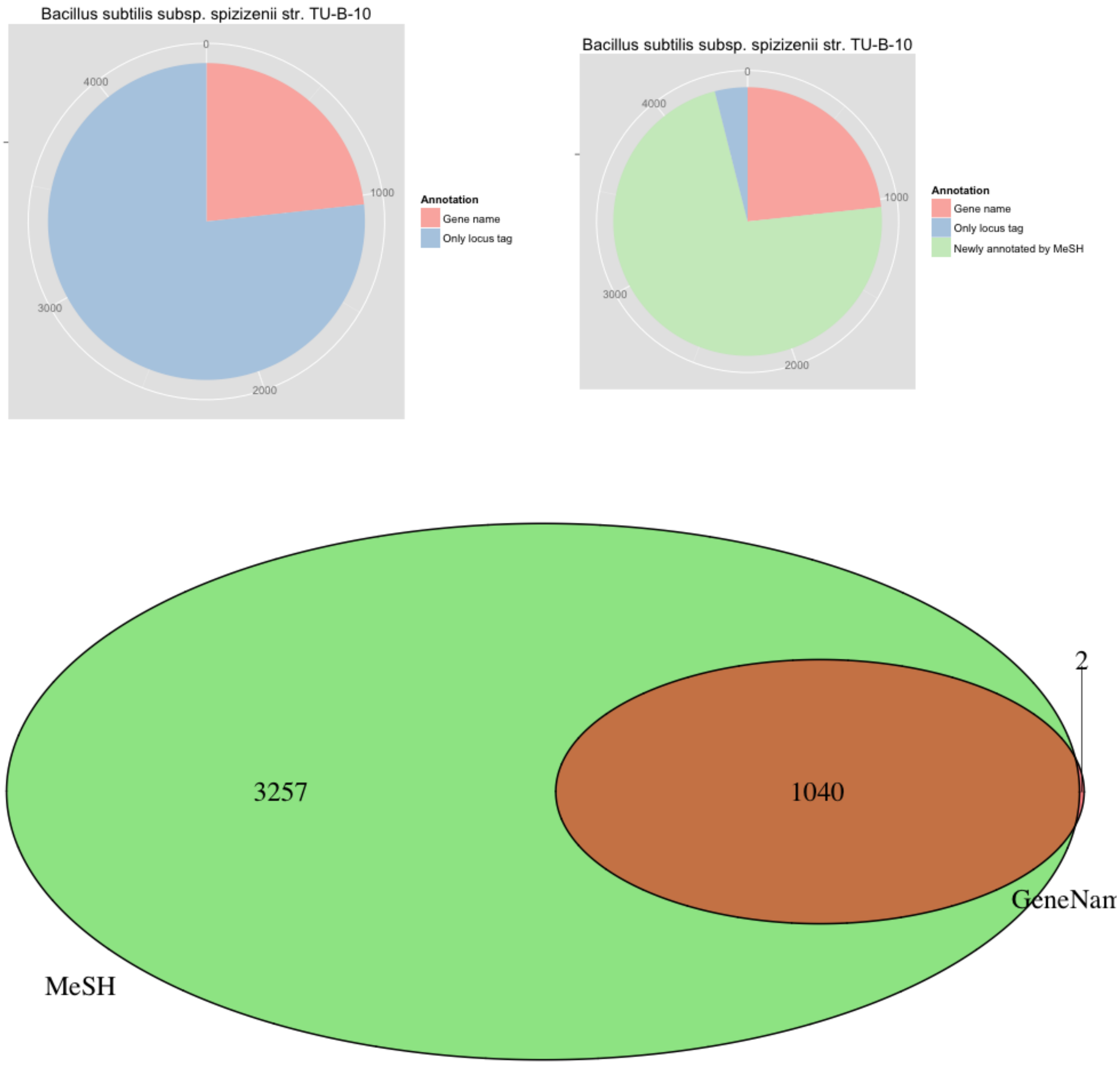

Figure 12: org.MeSH.Bsu.TUB10.db

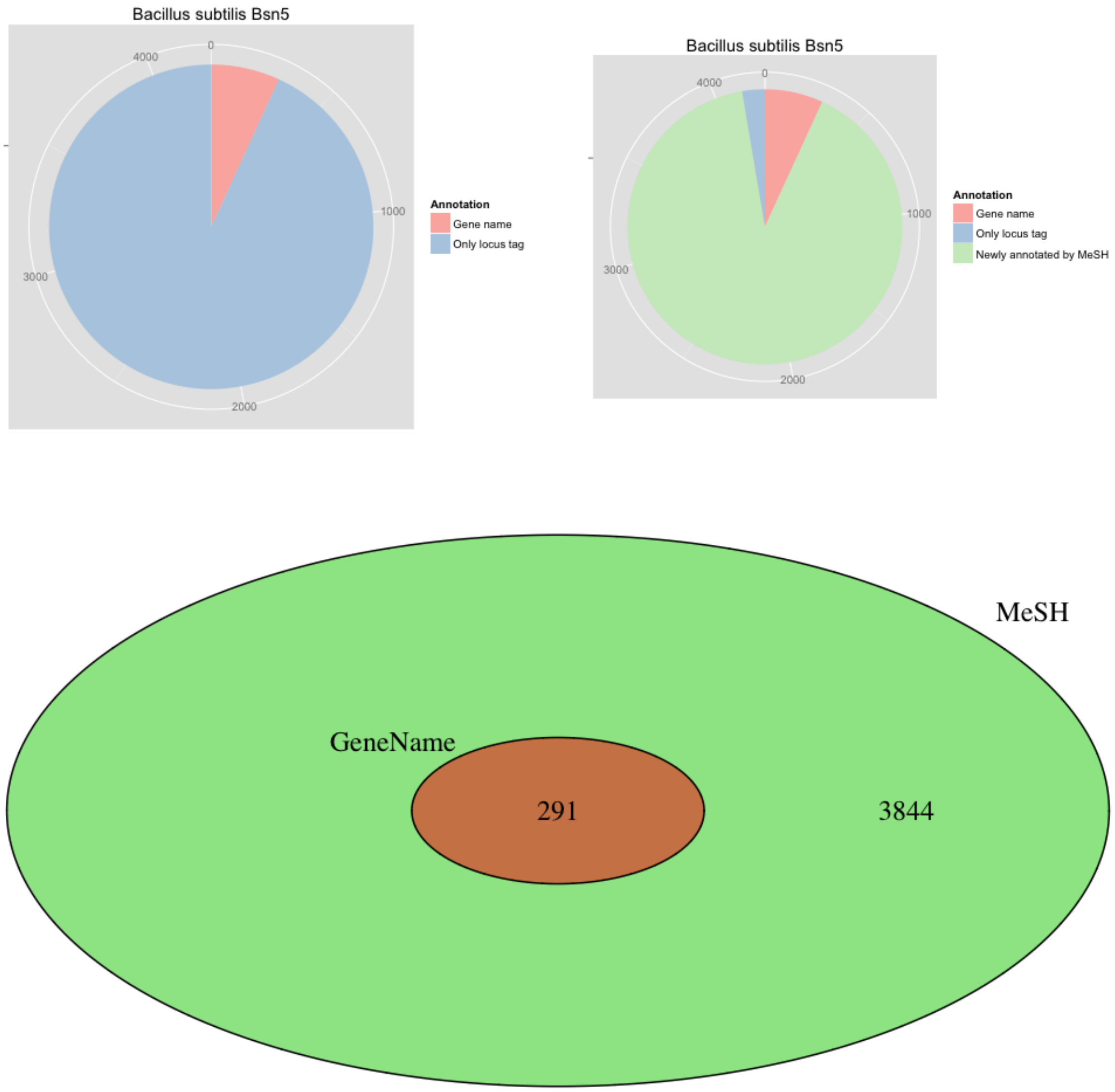

Figure 13: org.MeSH.Bsu.Bsn5.db

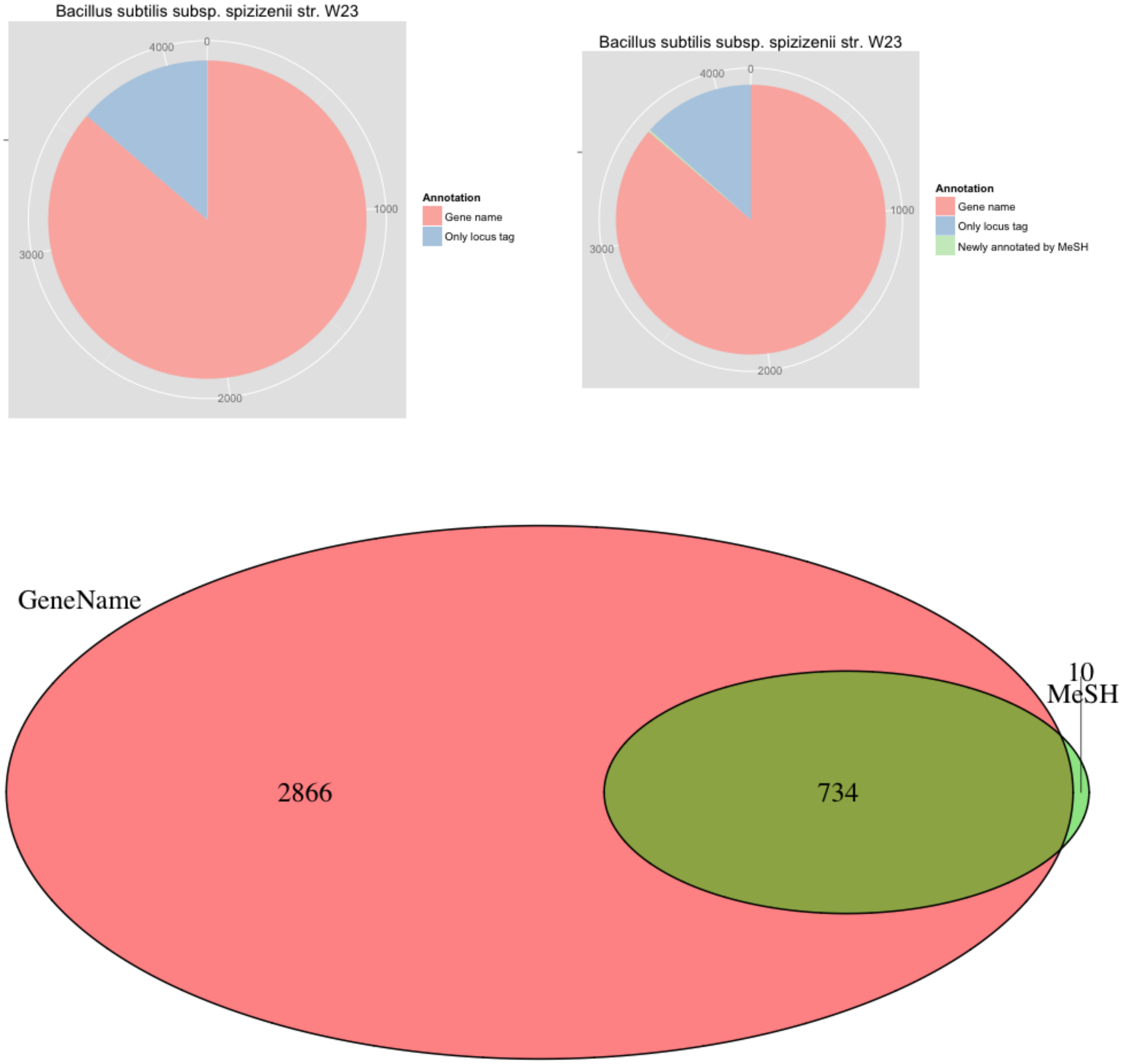

Figure 14: org.MeSH.Bsu.W23.db

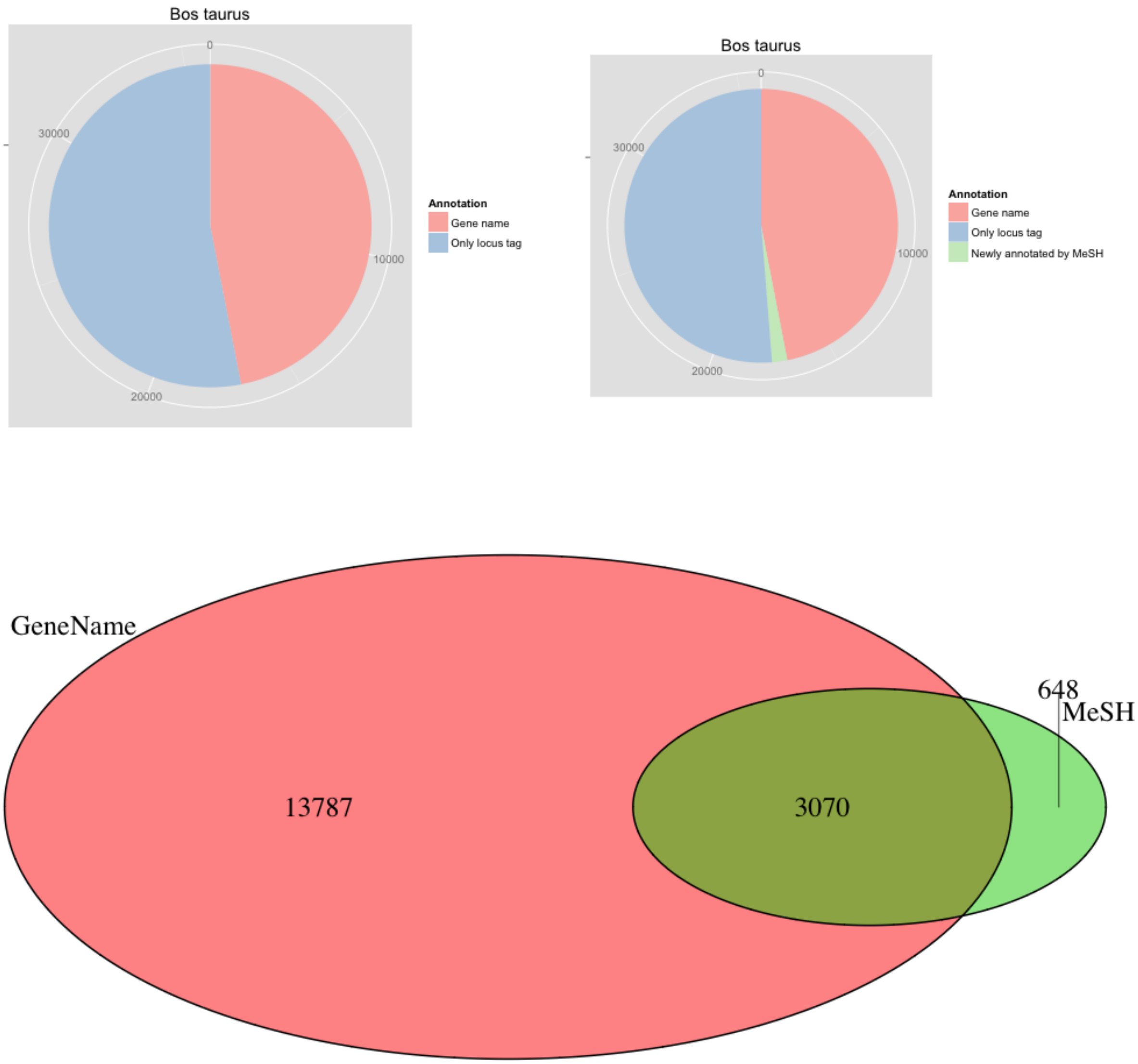

Figure 15: org.MeSH.Bta.db

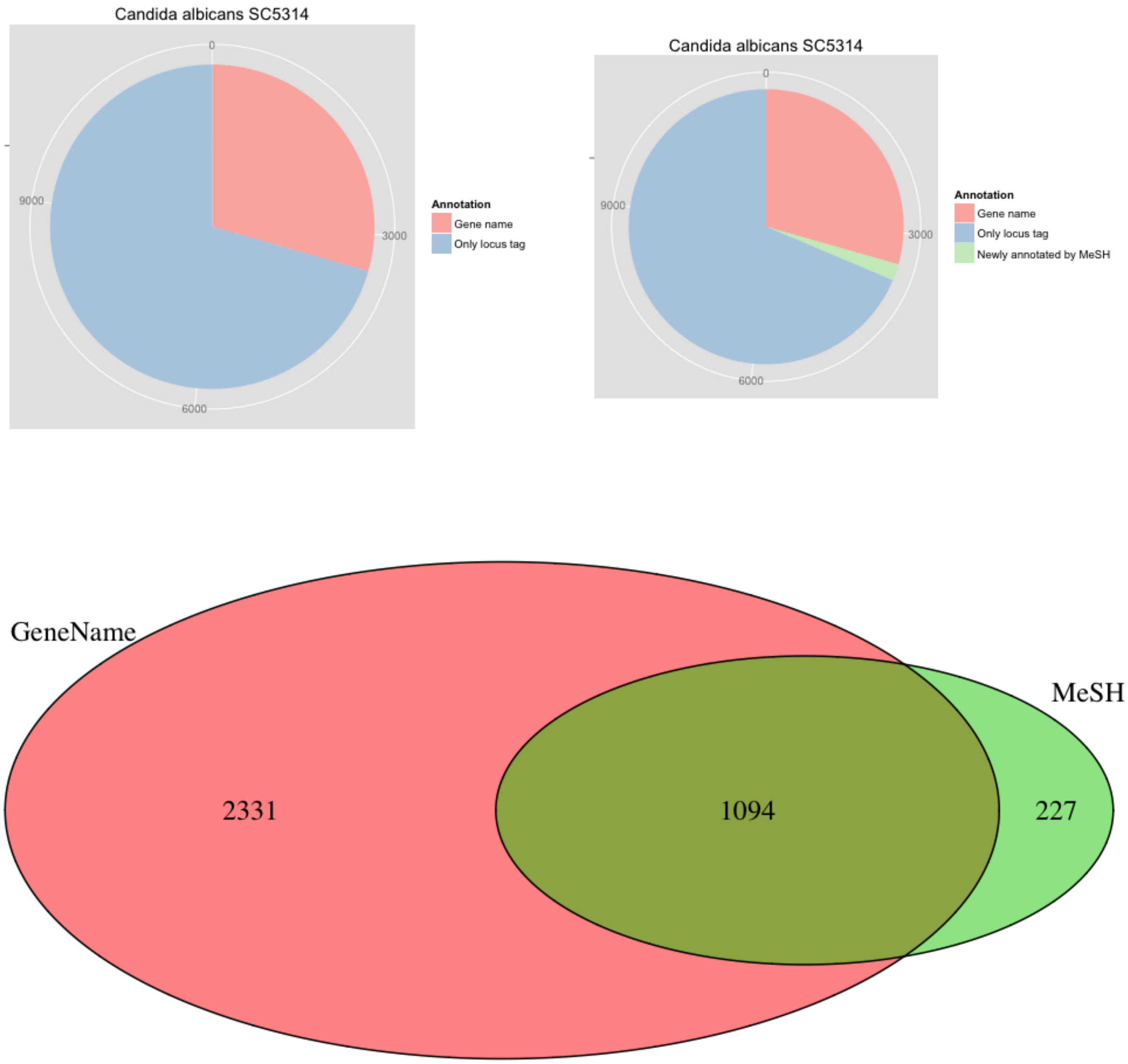

Figure 16: org.MeSH.Cal.SC5314.db

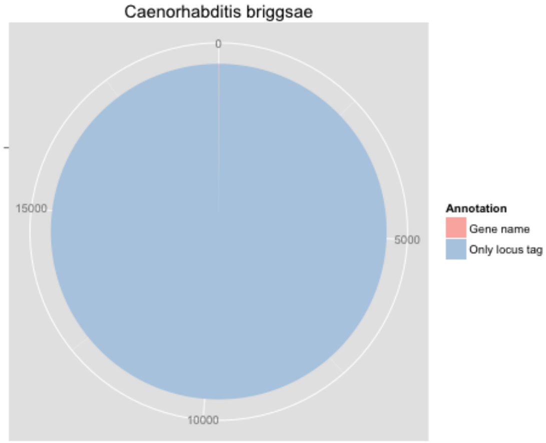

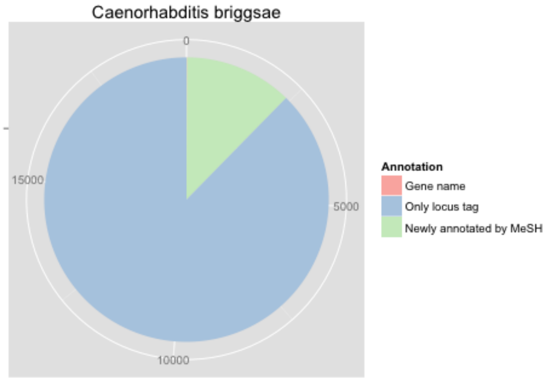

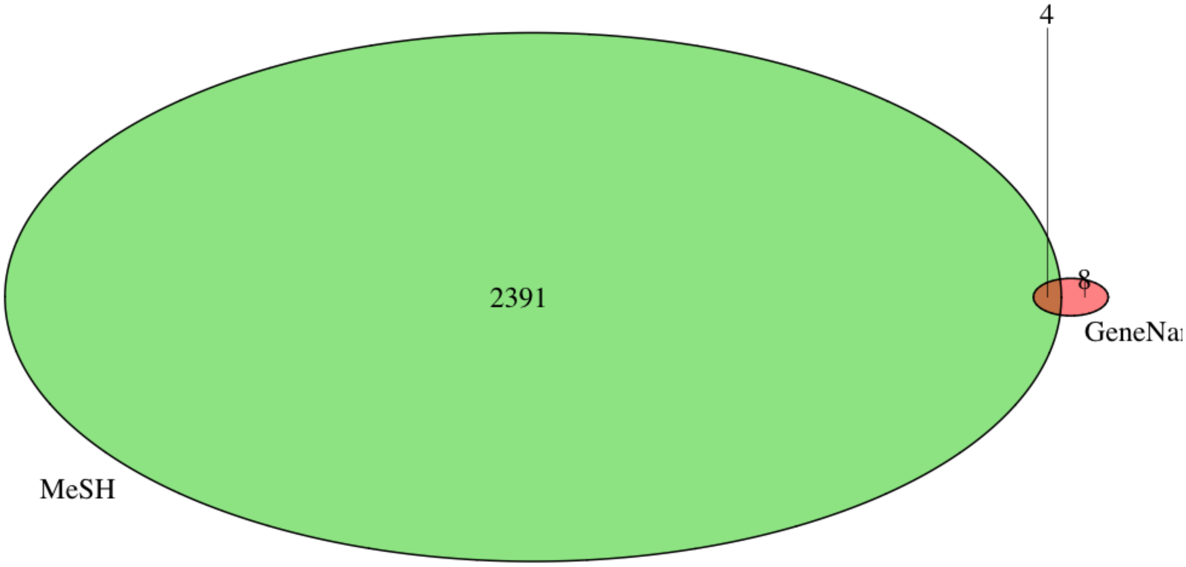

Figure 17: org.MeSH.Cbr.db

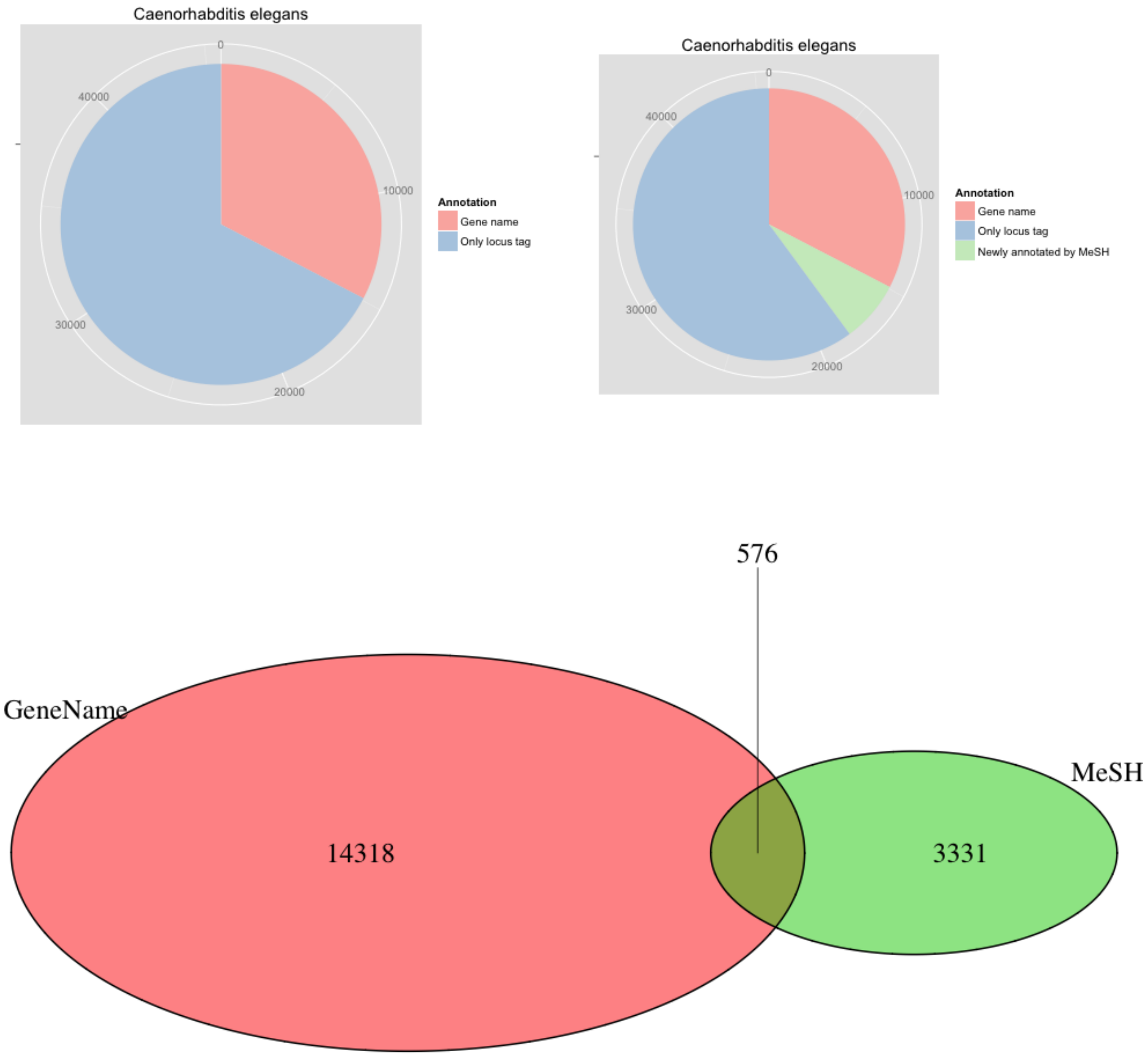

Figure 18: org.MeSH.Cel.db

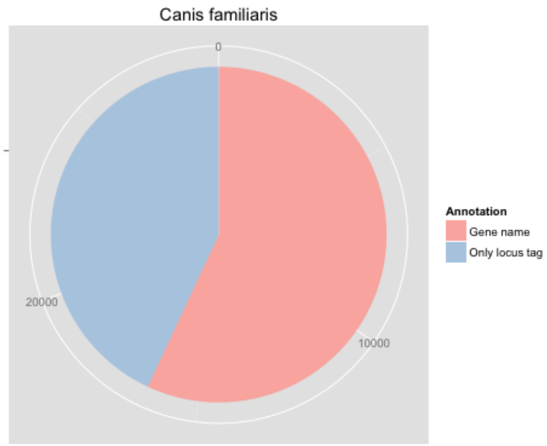

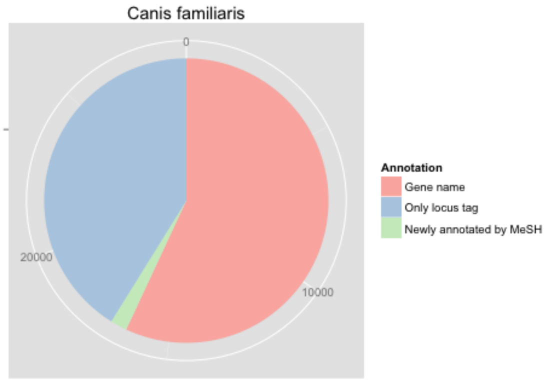

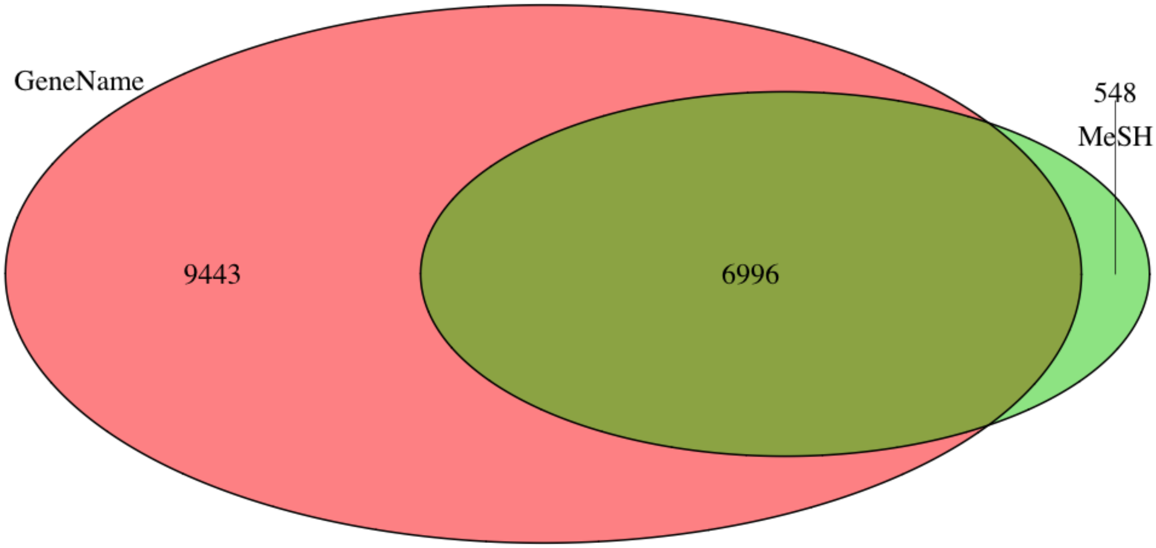

Figure 19: org.MeSH.Cfa.db

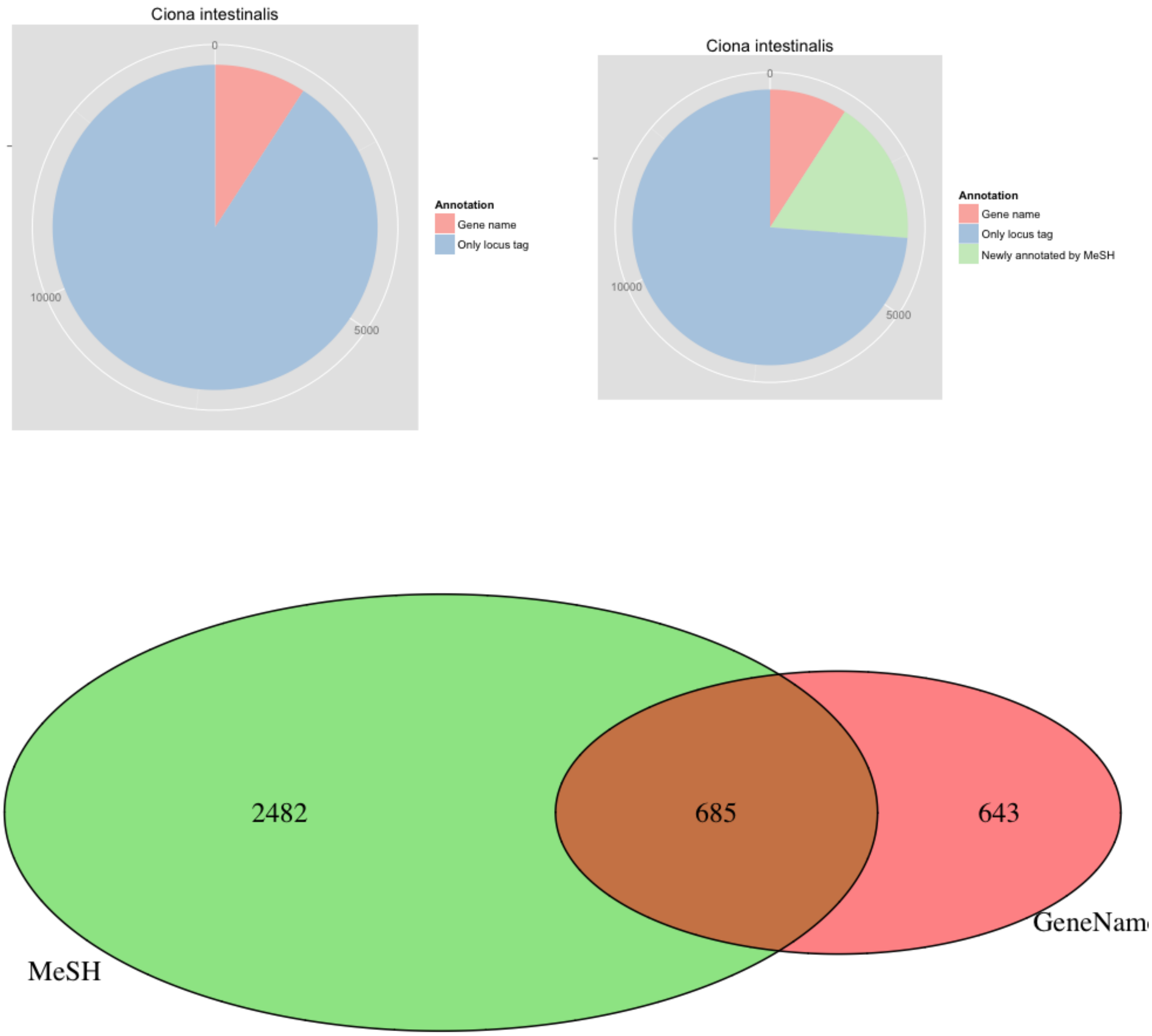

Figure 20: org.MeSH.Cin.db

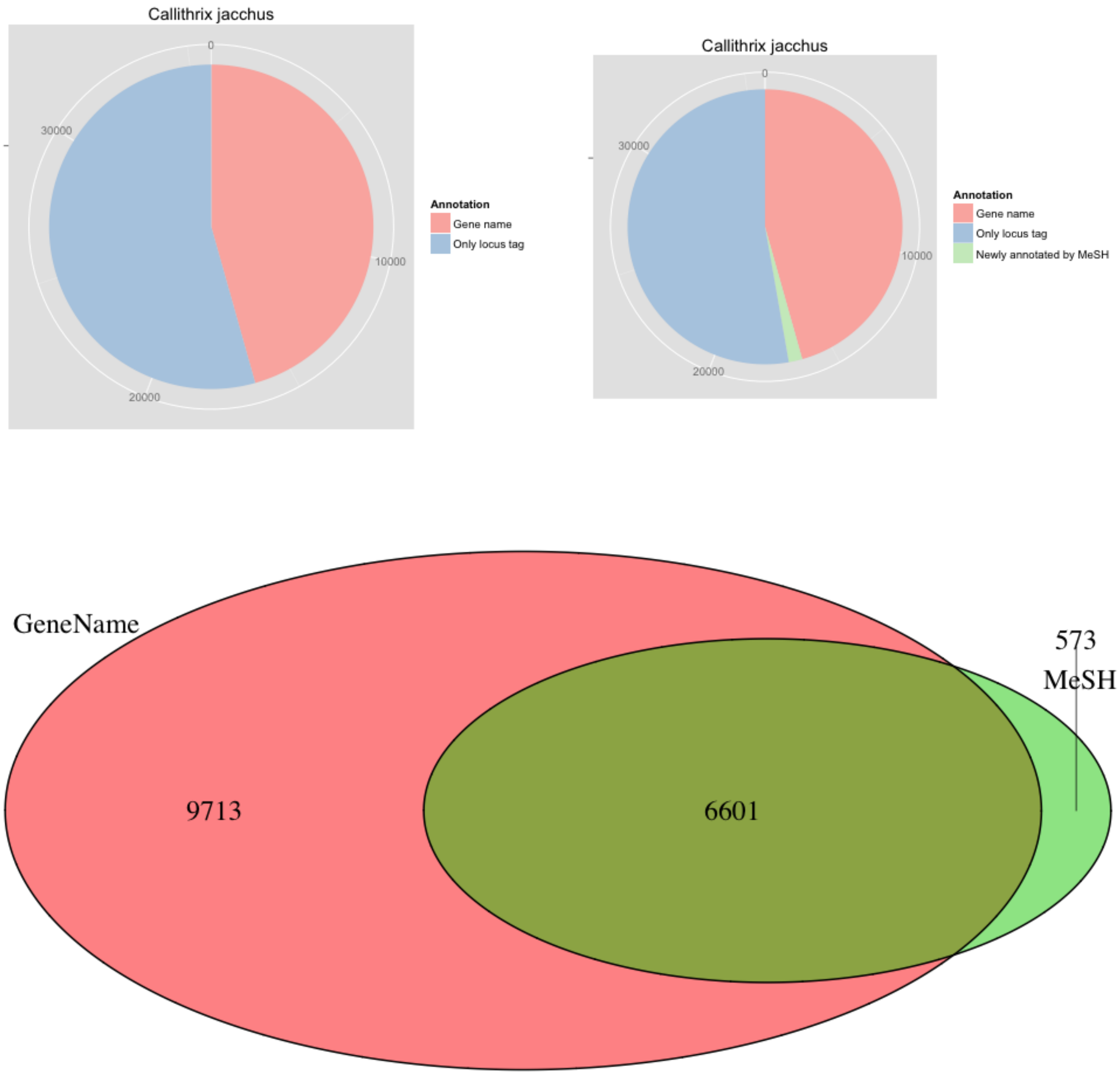

Figure 21: org.MeSH.Cja.db

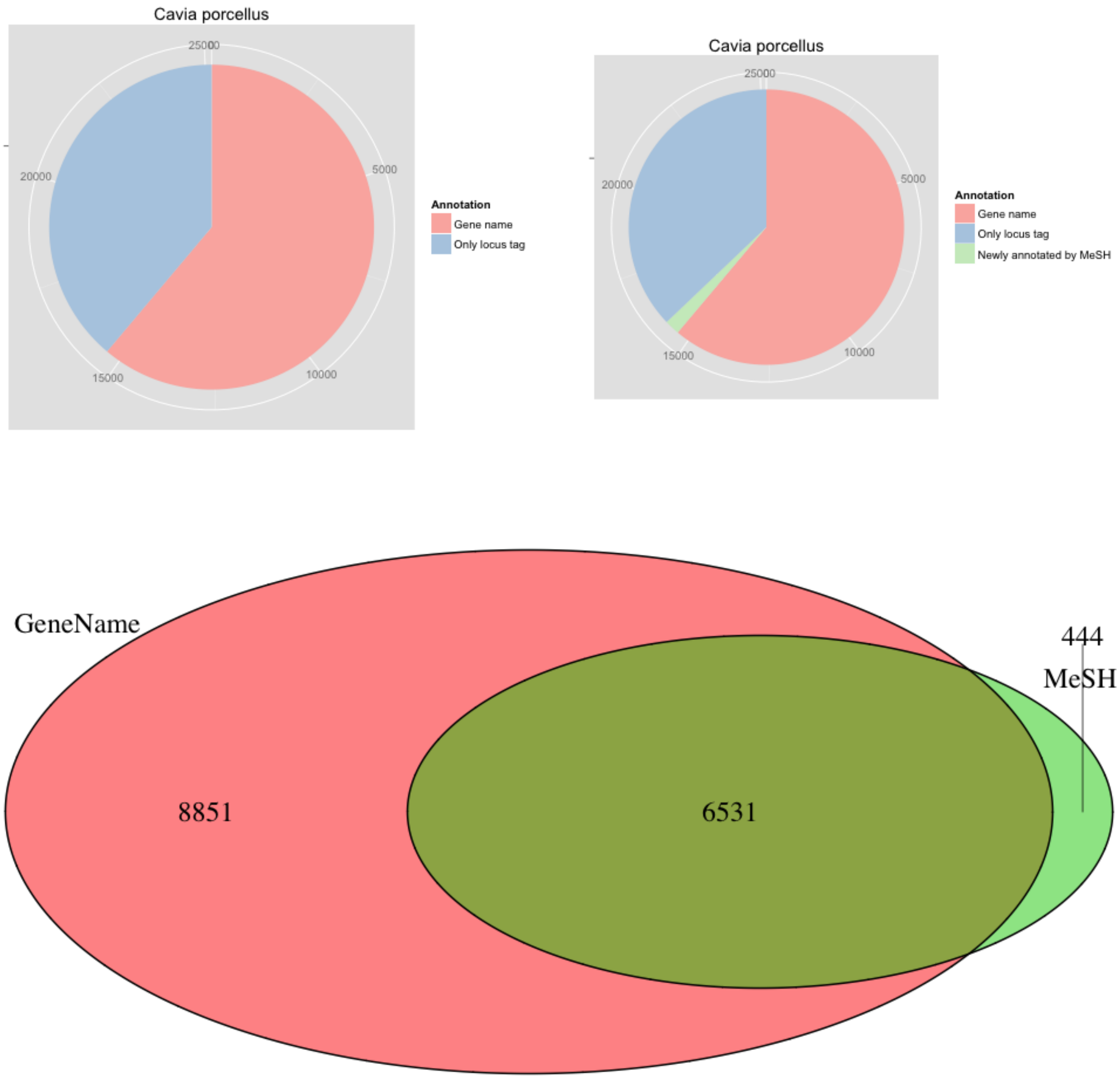

Figure 22: org.MeSH.Cpo.db

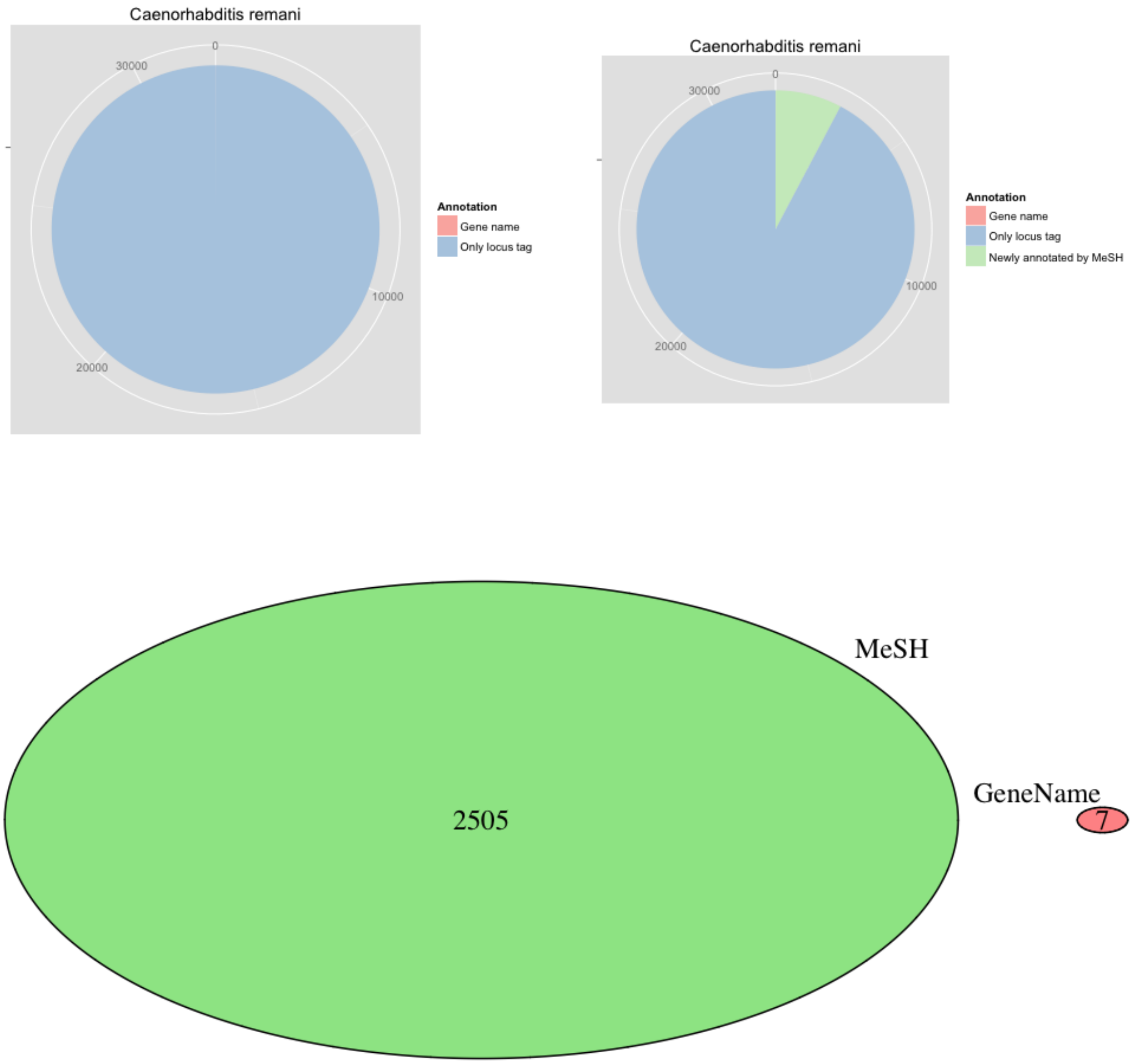

Figure 23: org.MeSH.Cre.db

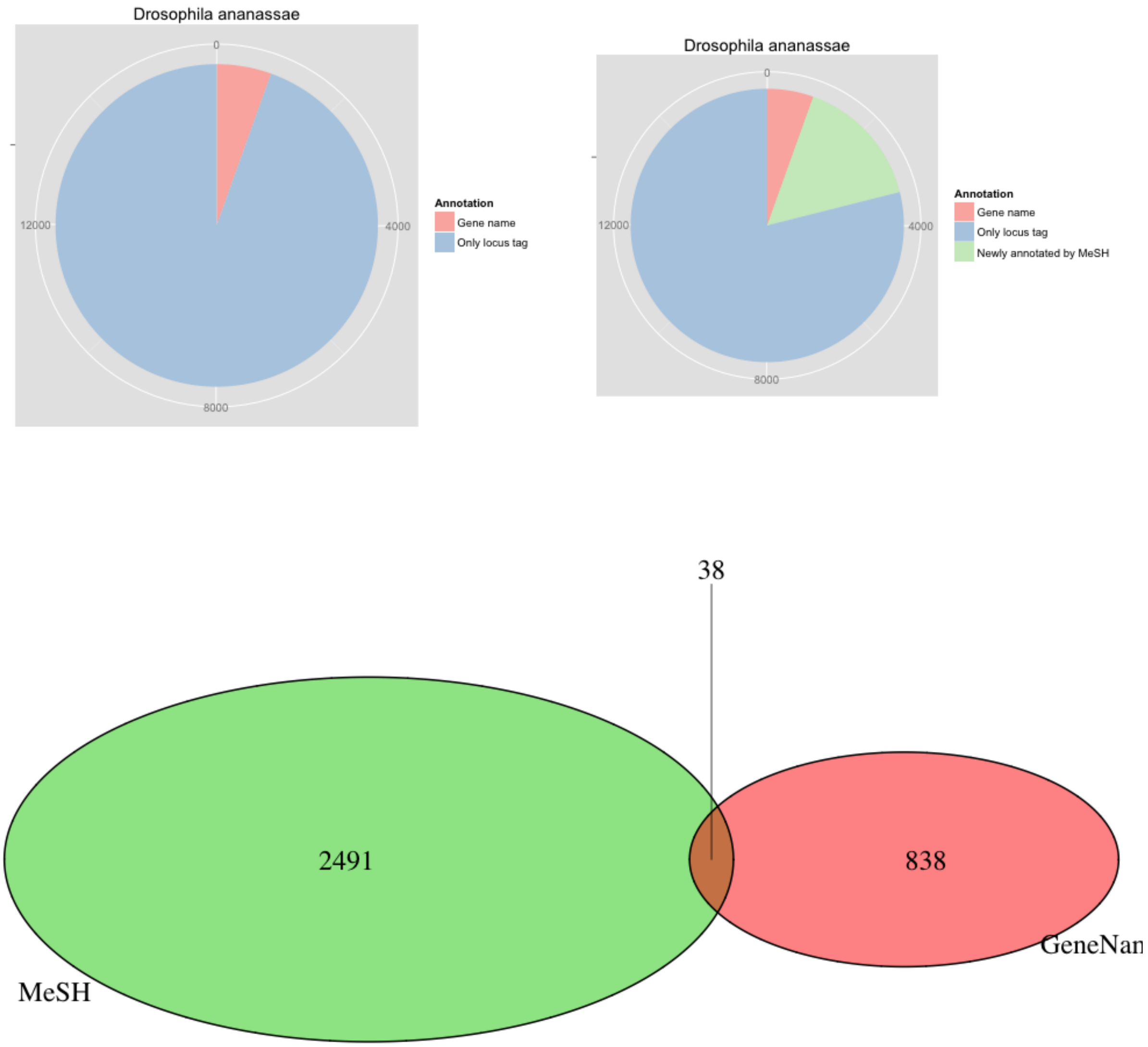

Figure 24: org.MeSH.Dan.db

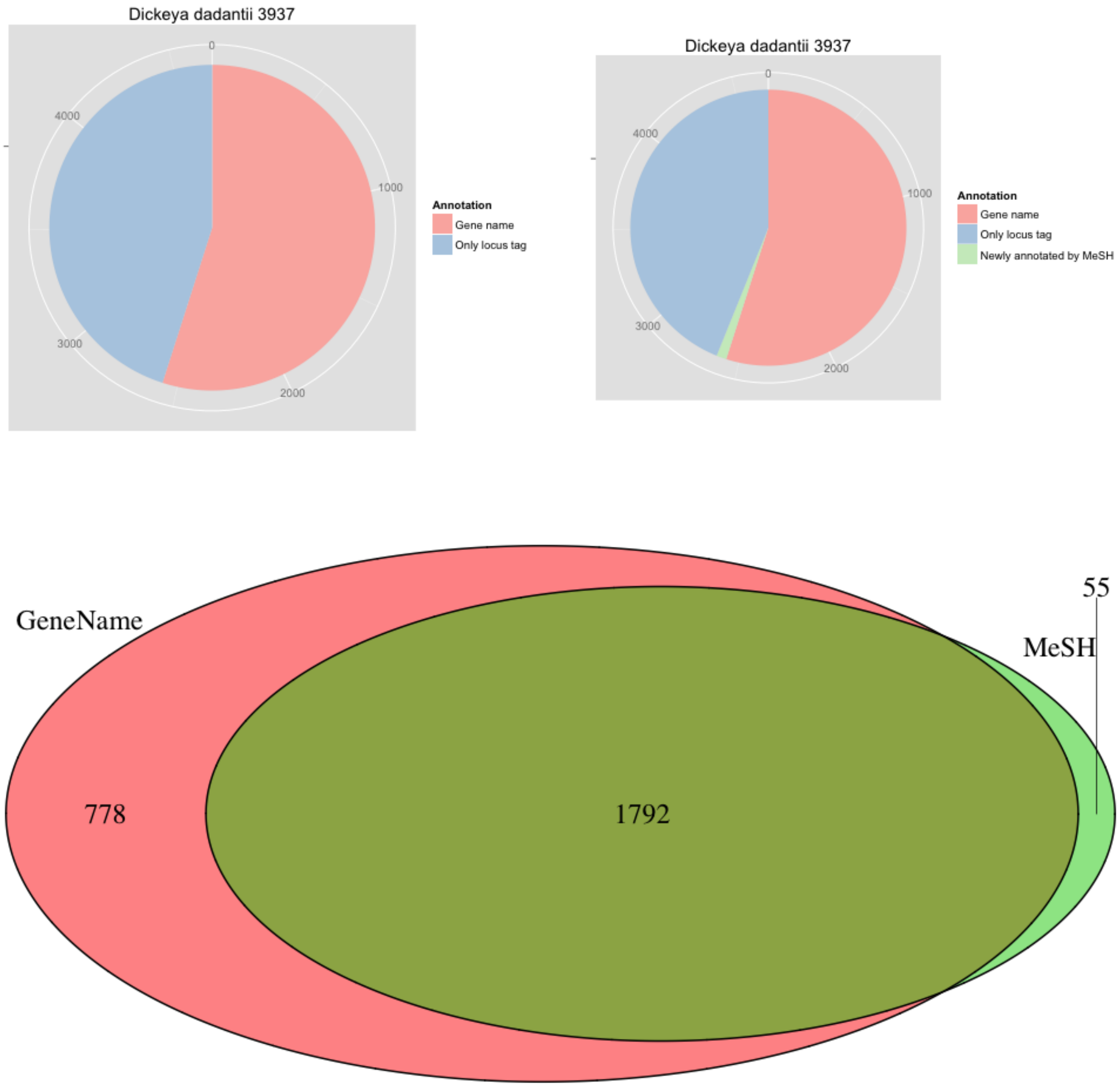

Figure 25: org.MeSH.Dda.3937.db

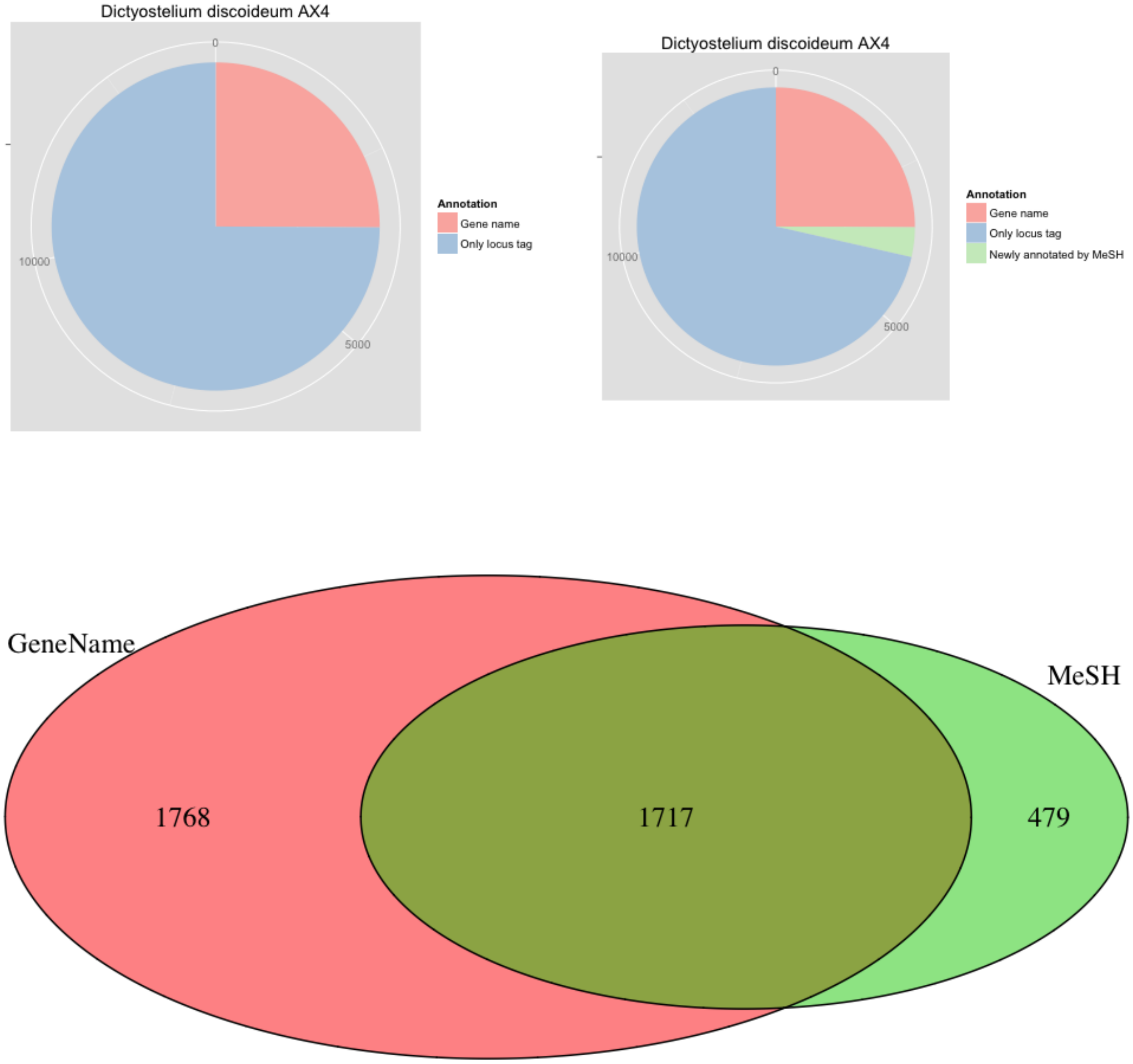

Figure 26: org.MeSH.Ddi.AX4.db

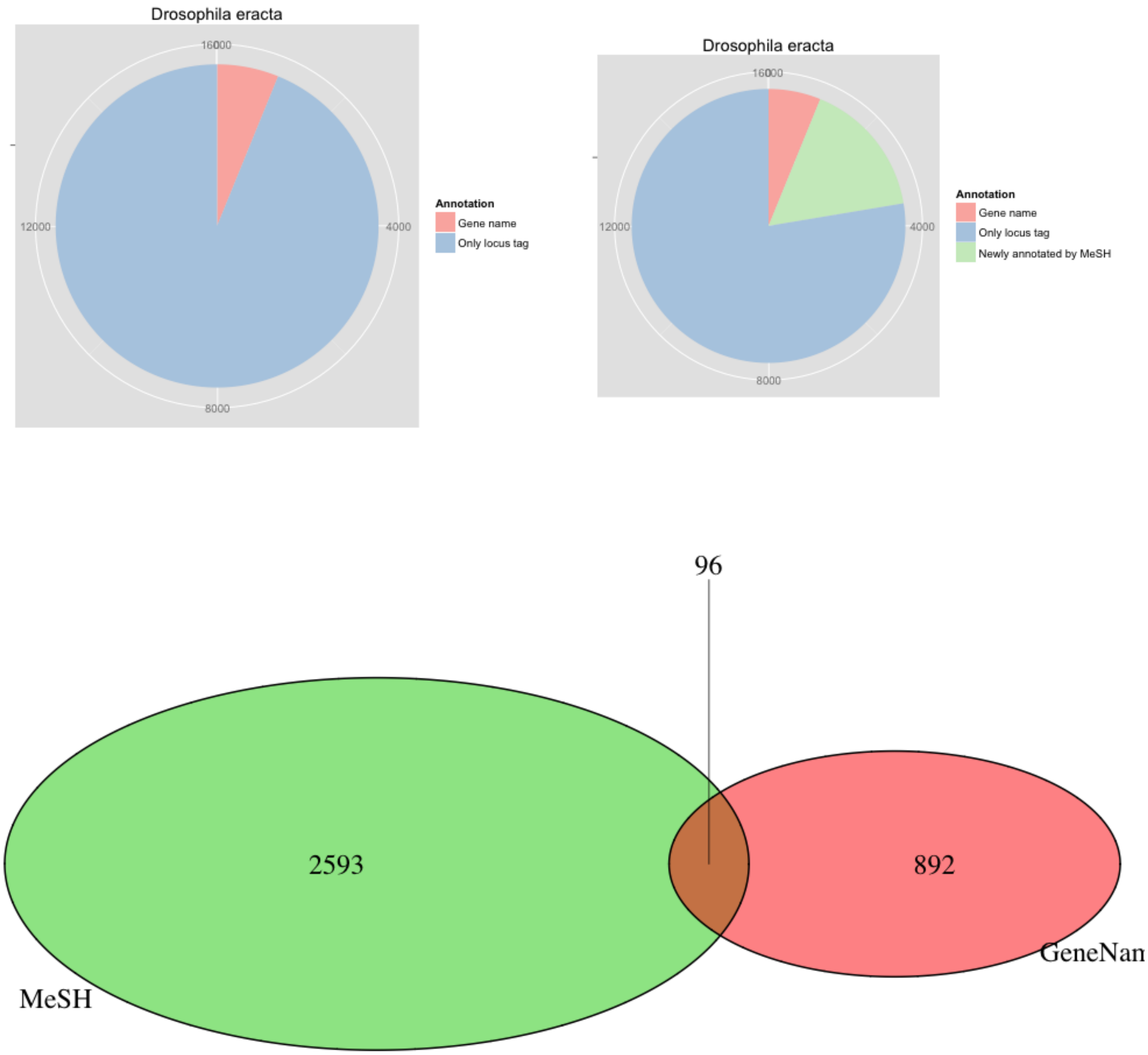

Figure 27: org.MeSH.Der.db

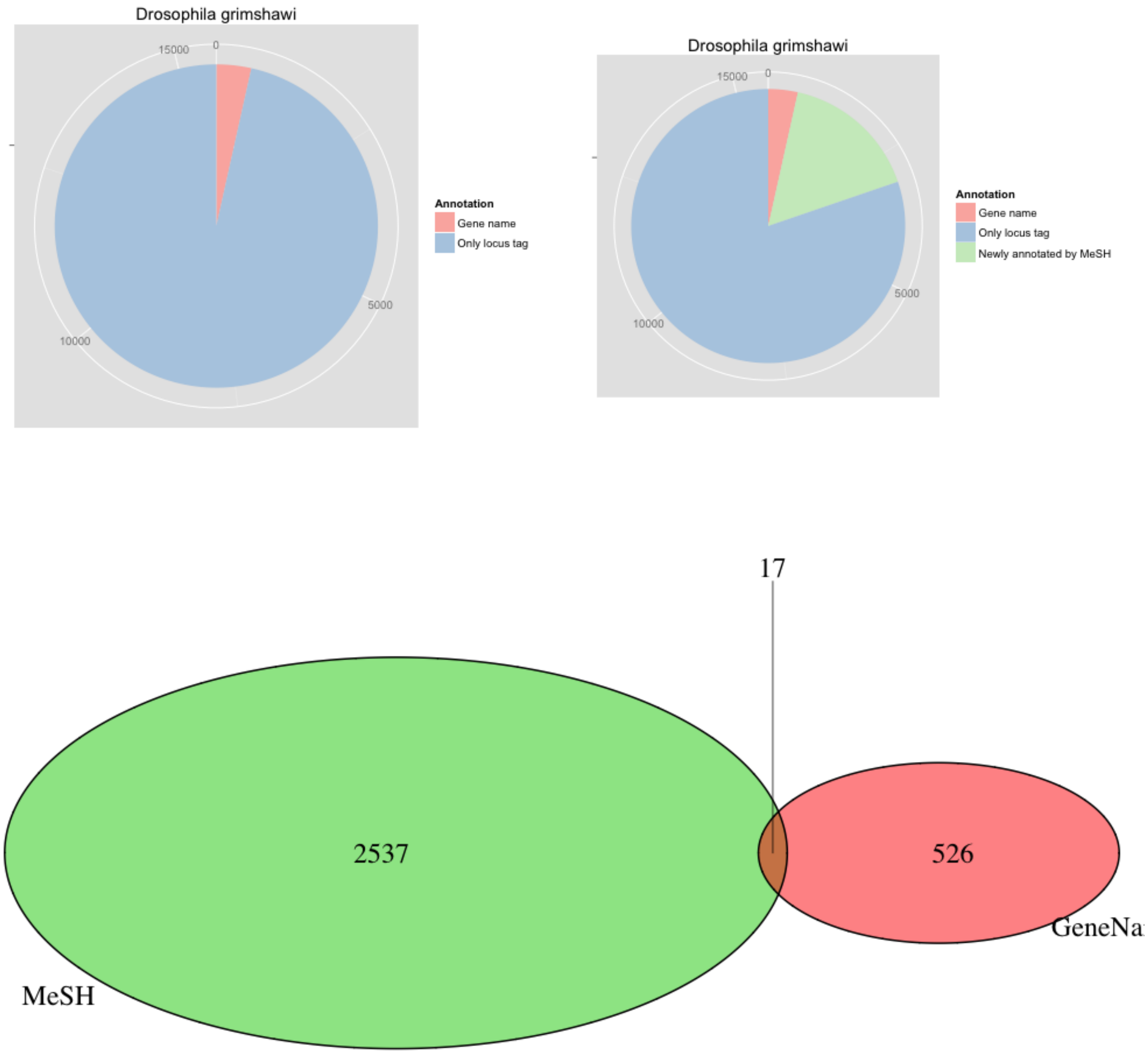

Figure 28: org.MeSH.Dgr.db

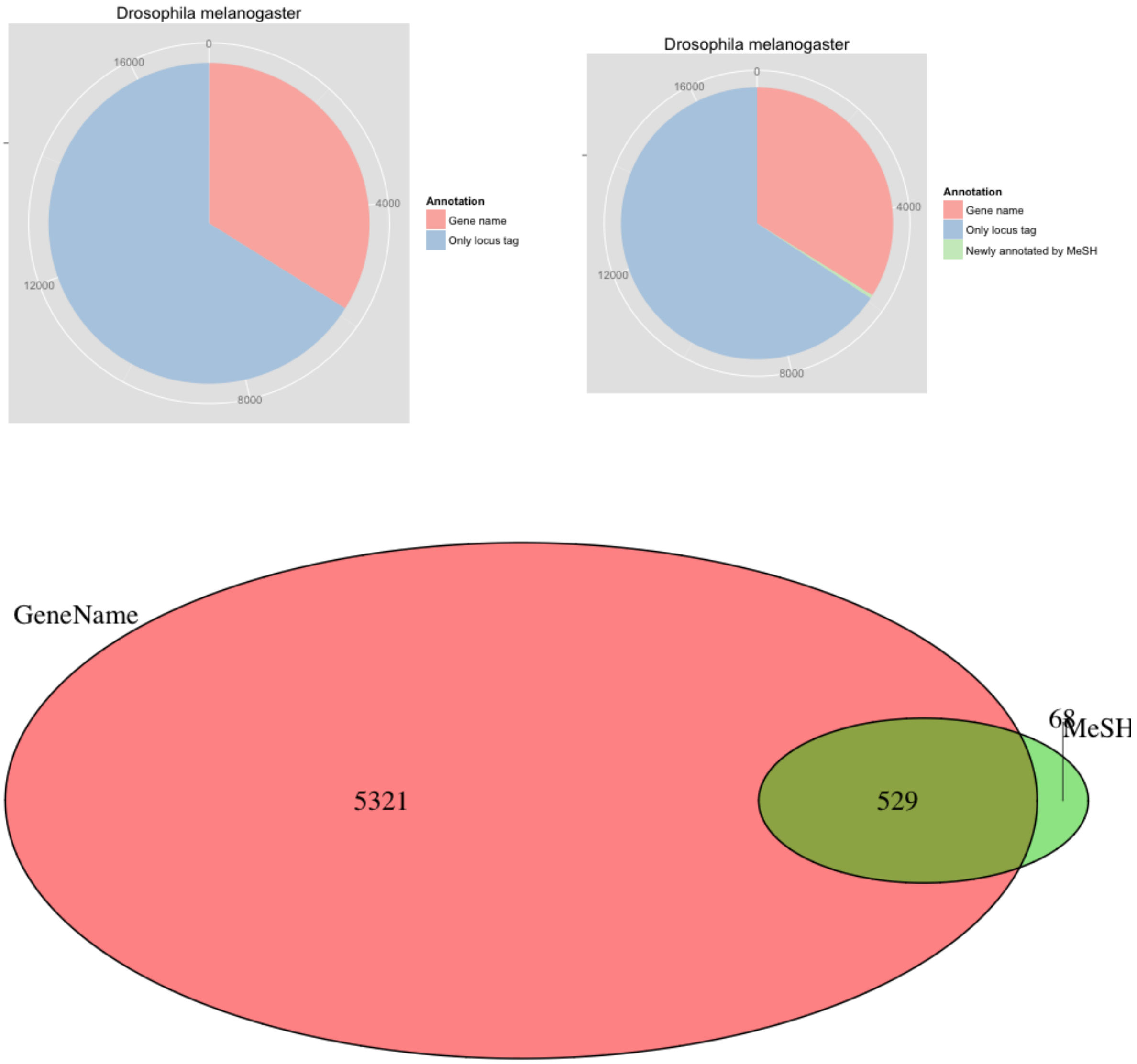

Figure 29: org.MeSH.Dme.db

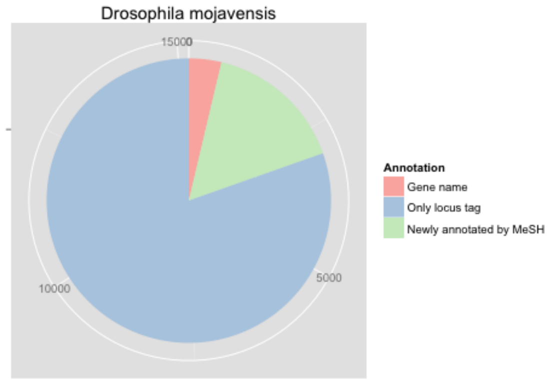

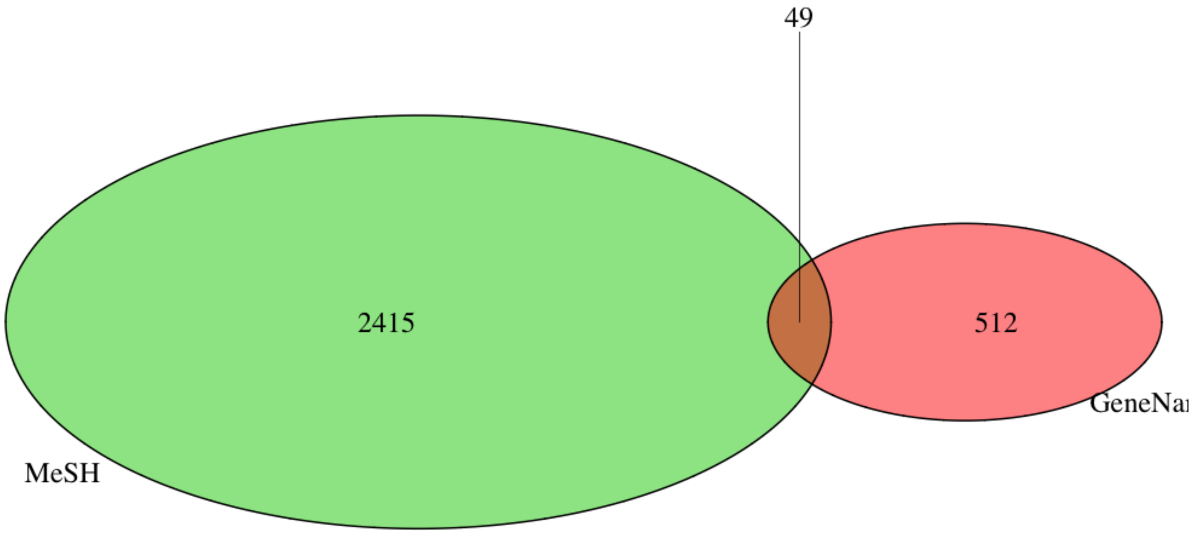

Figure 30: org.MeSH.Dmo.db

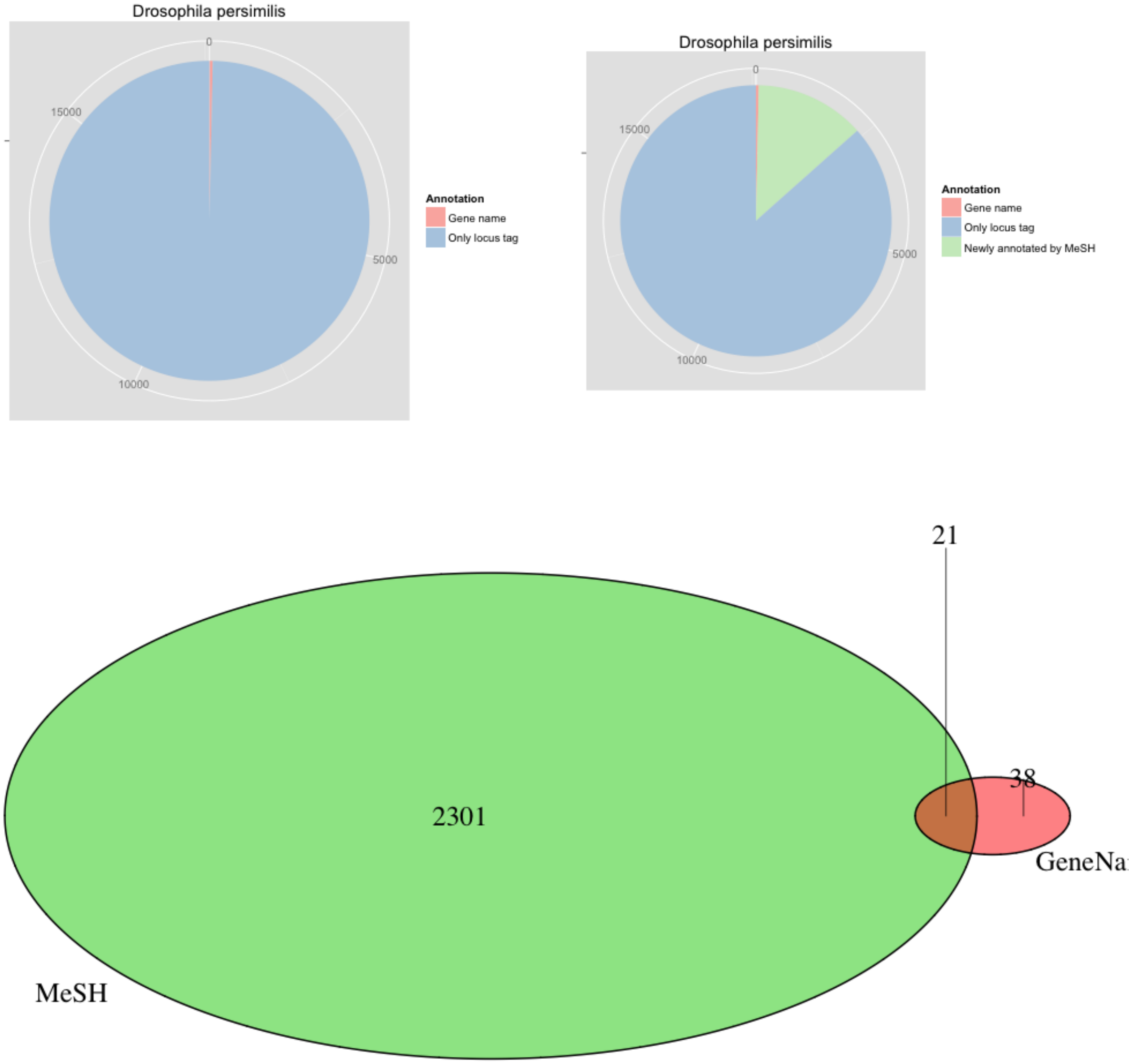

Figure 31: org.MeSH.Dpe.db

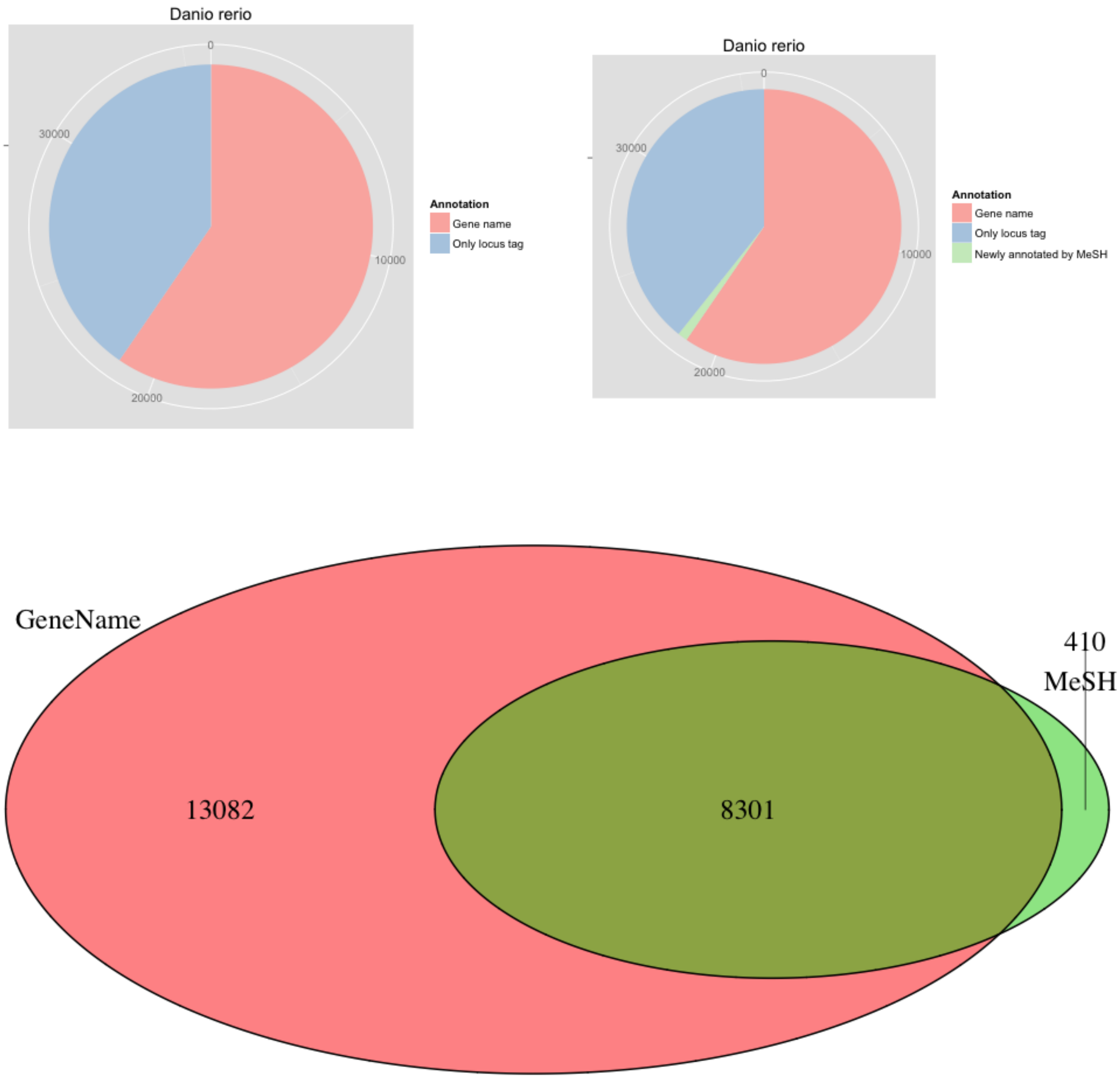

Figure 32: org.MeSH.Dre.db

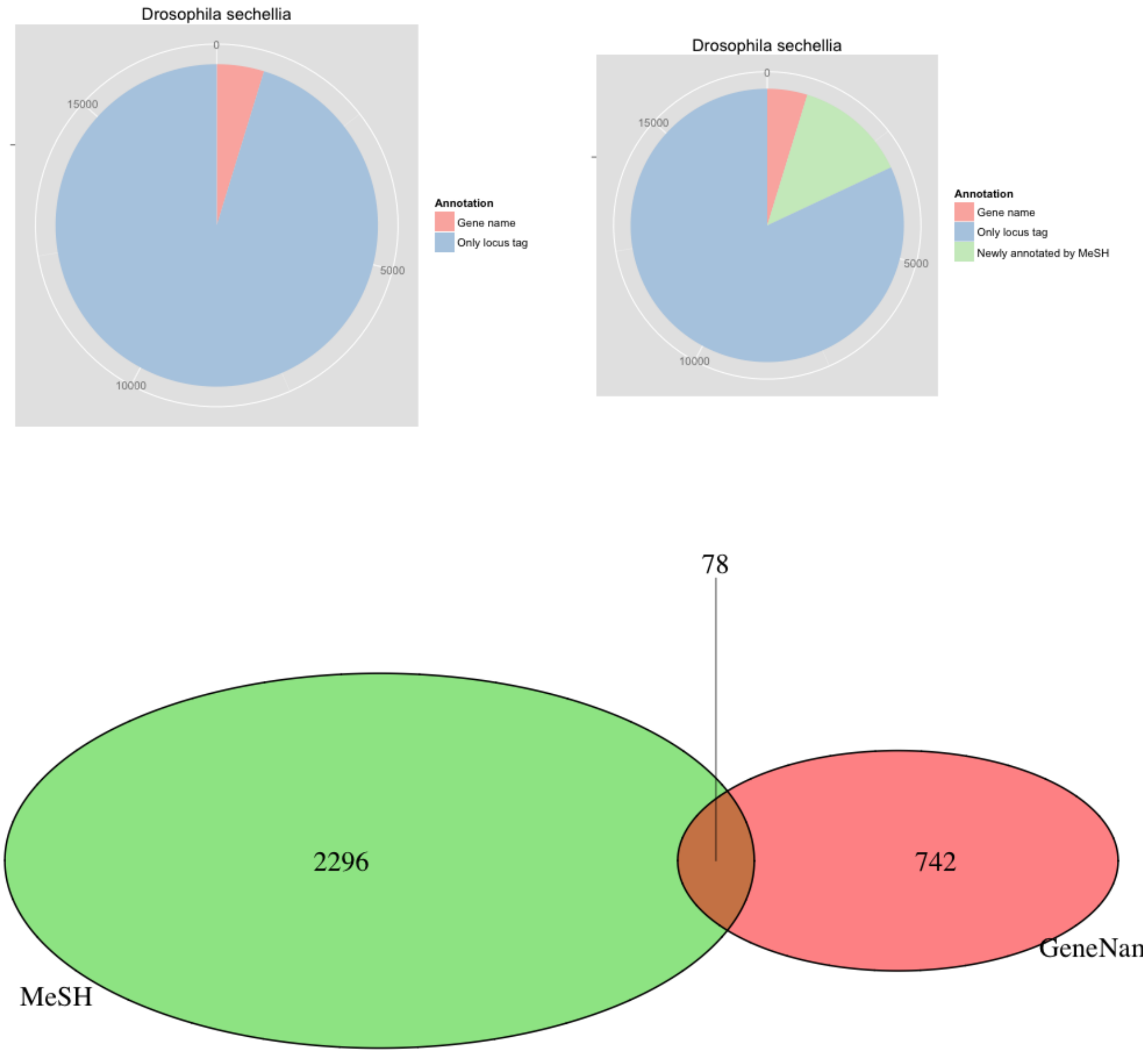

Figure 33: org.MeSH.Dse.db

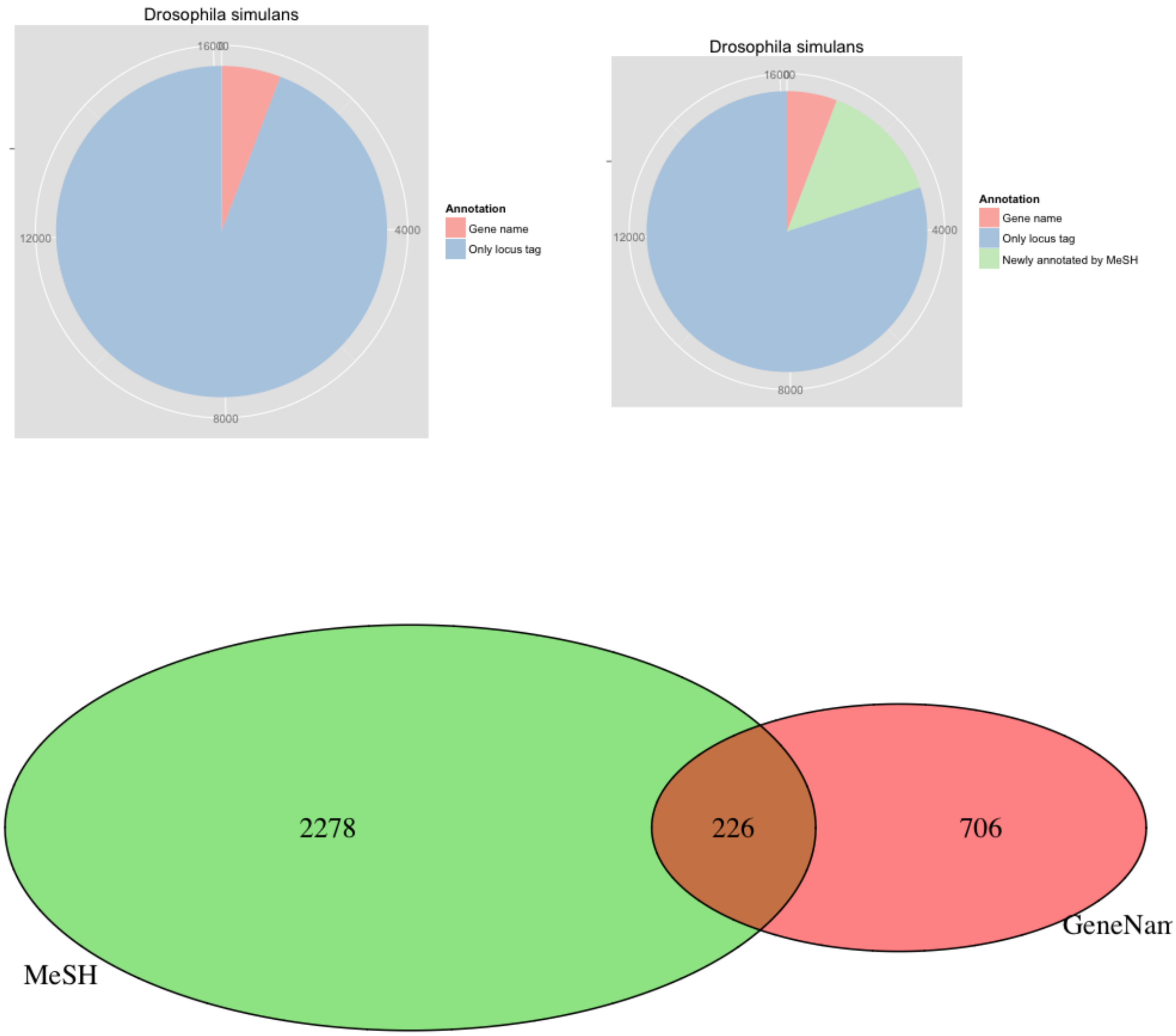

Figure 34: org.MeSH.Dsi.db

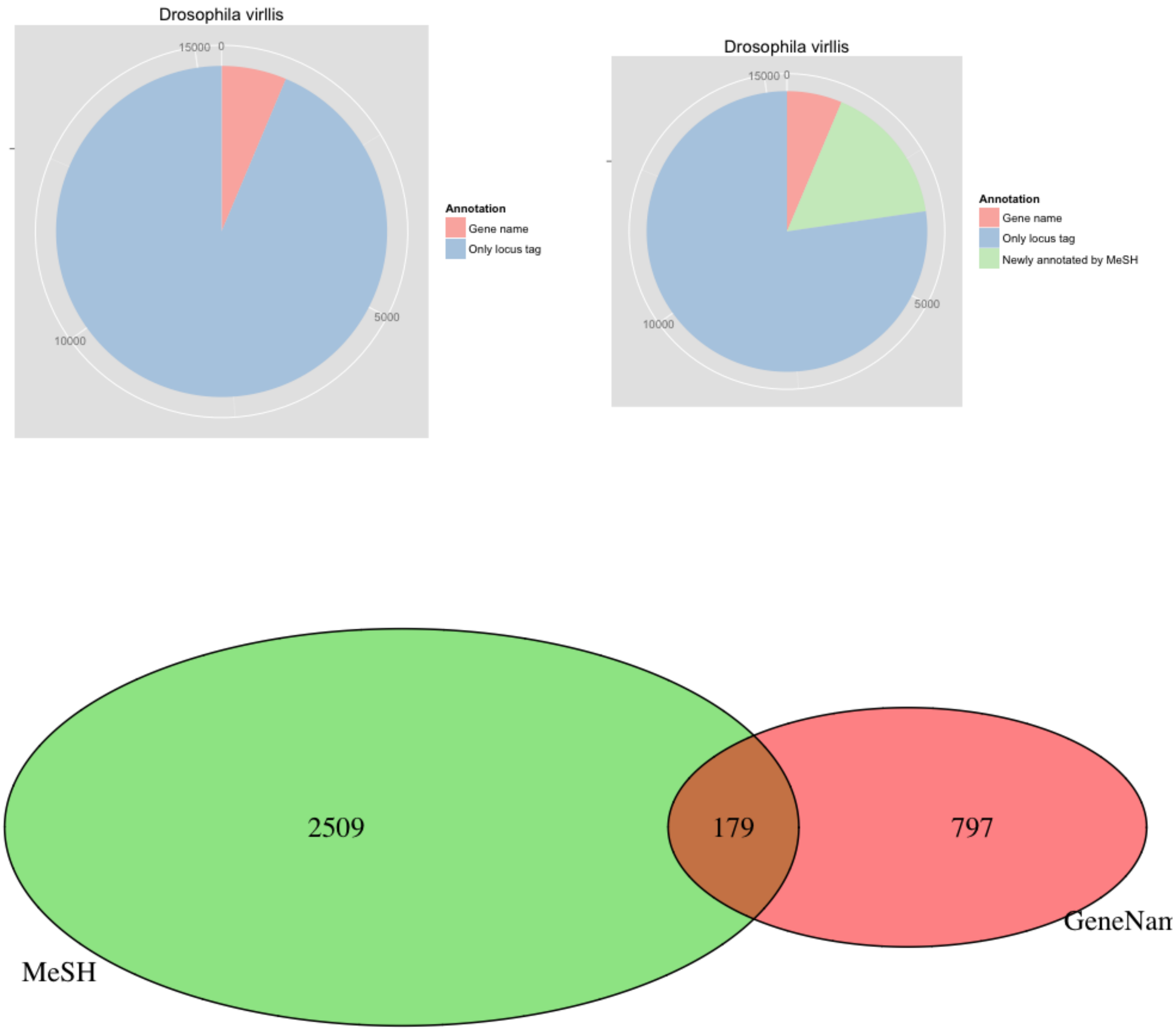

Figure 35: org.MeSH.Dvi.db

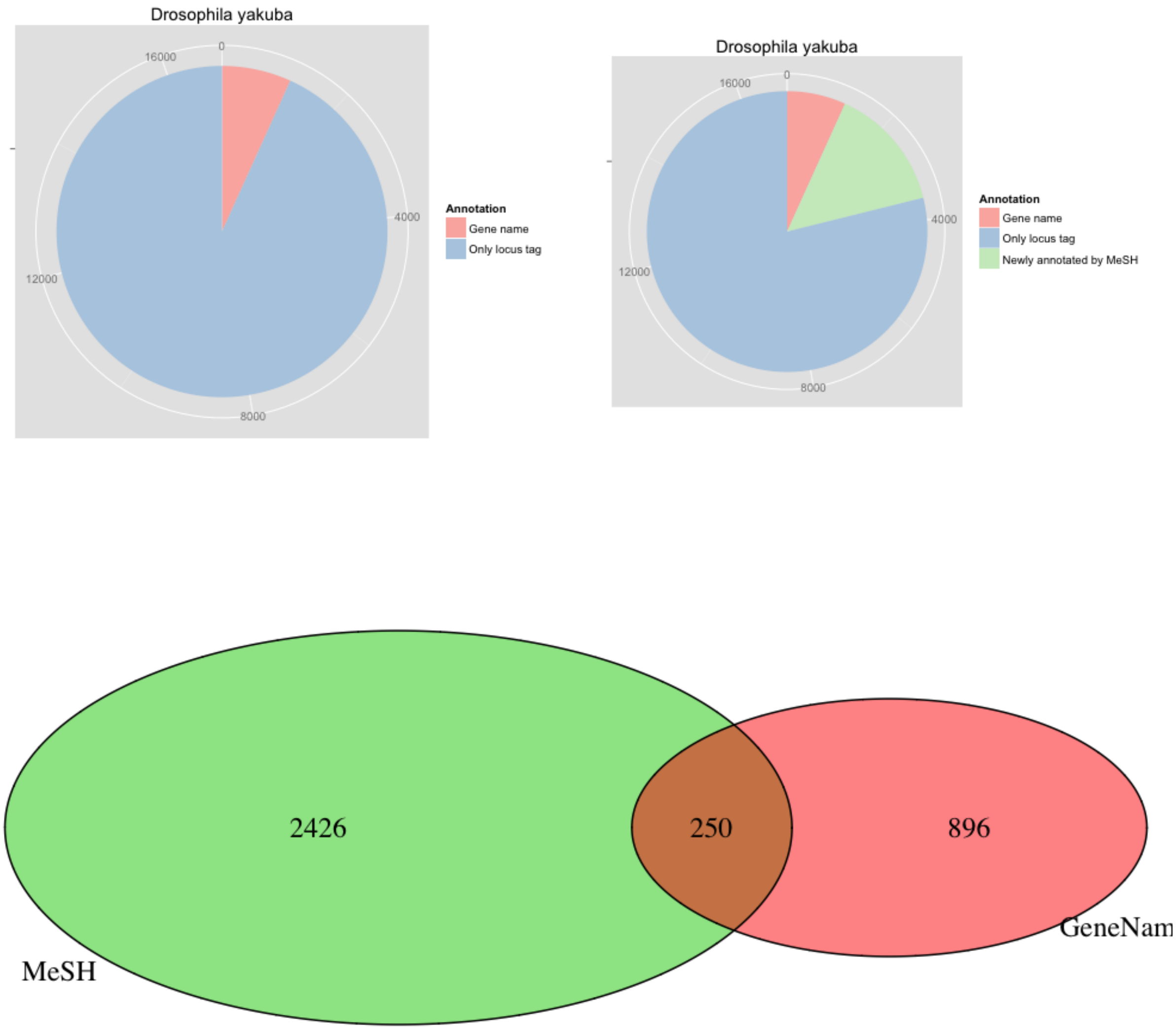

Figure 36: org.MeSH.Dya.db

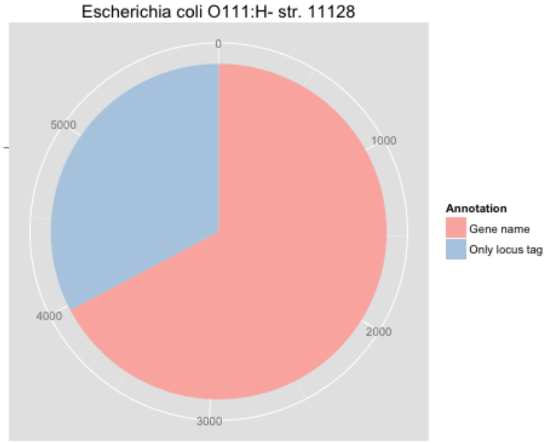

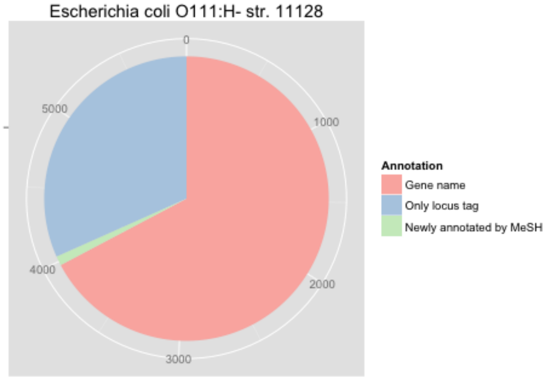

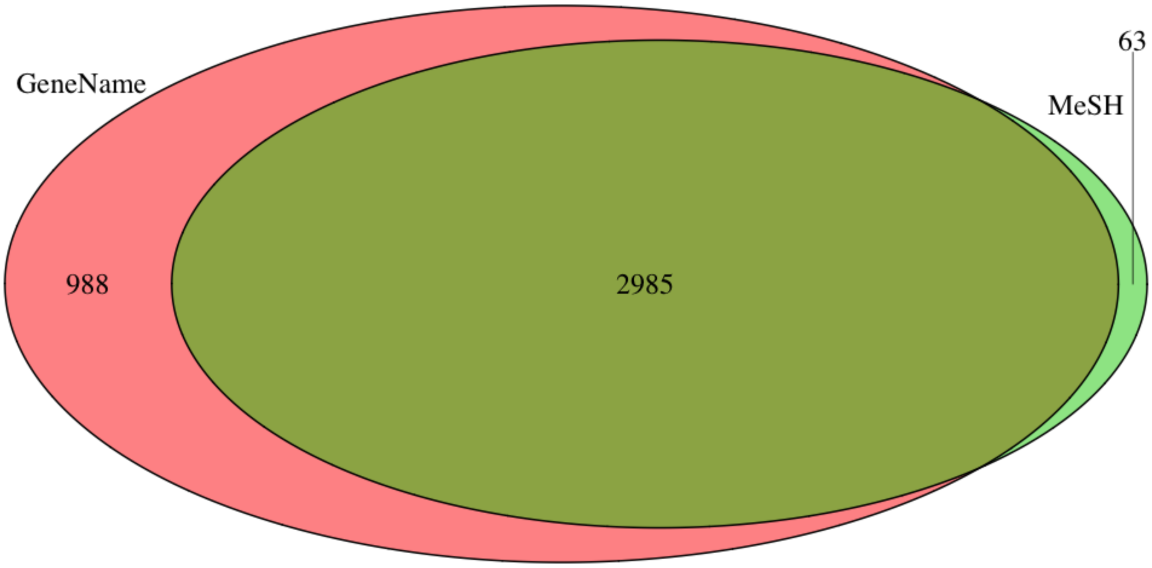

Figure 37: org.MeSH.Eco.O111.H.11128.db

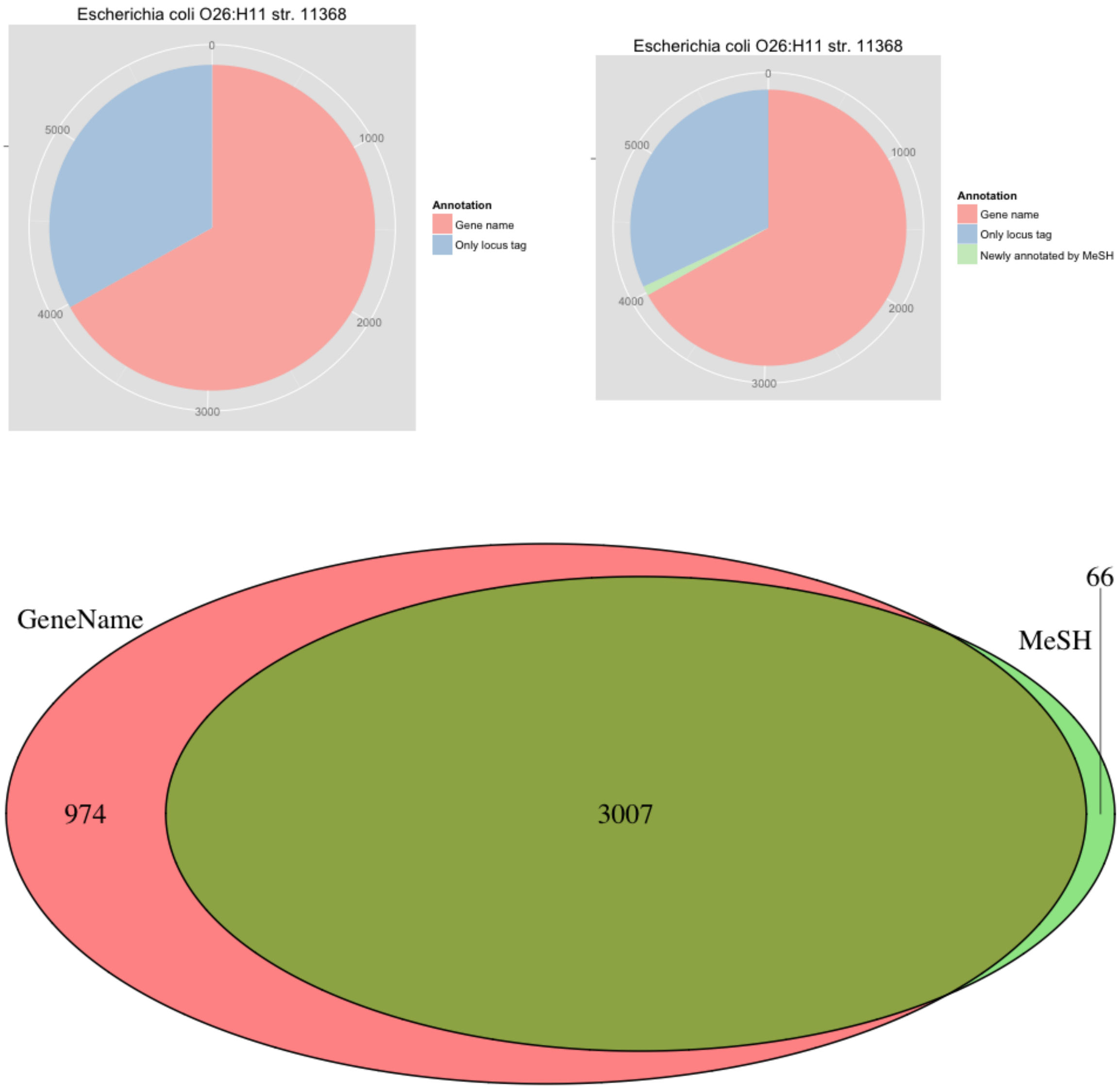

Figure 38: org.MeSH.Eco.O26.H11.11368.db

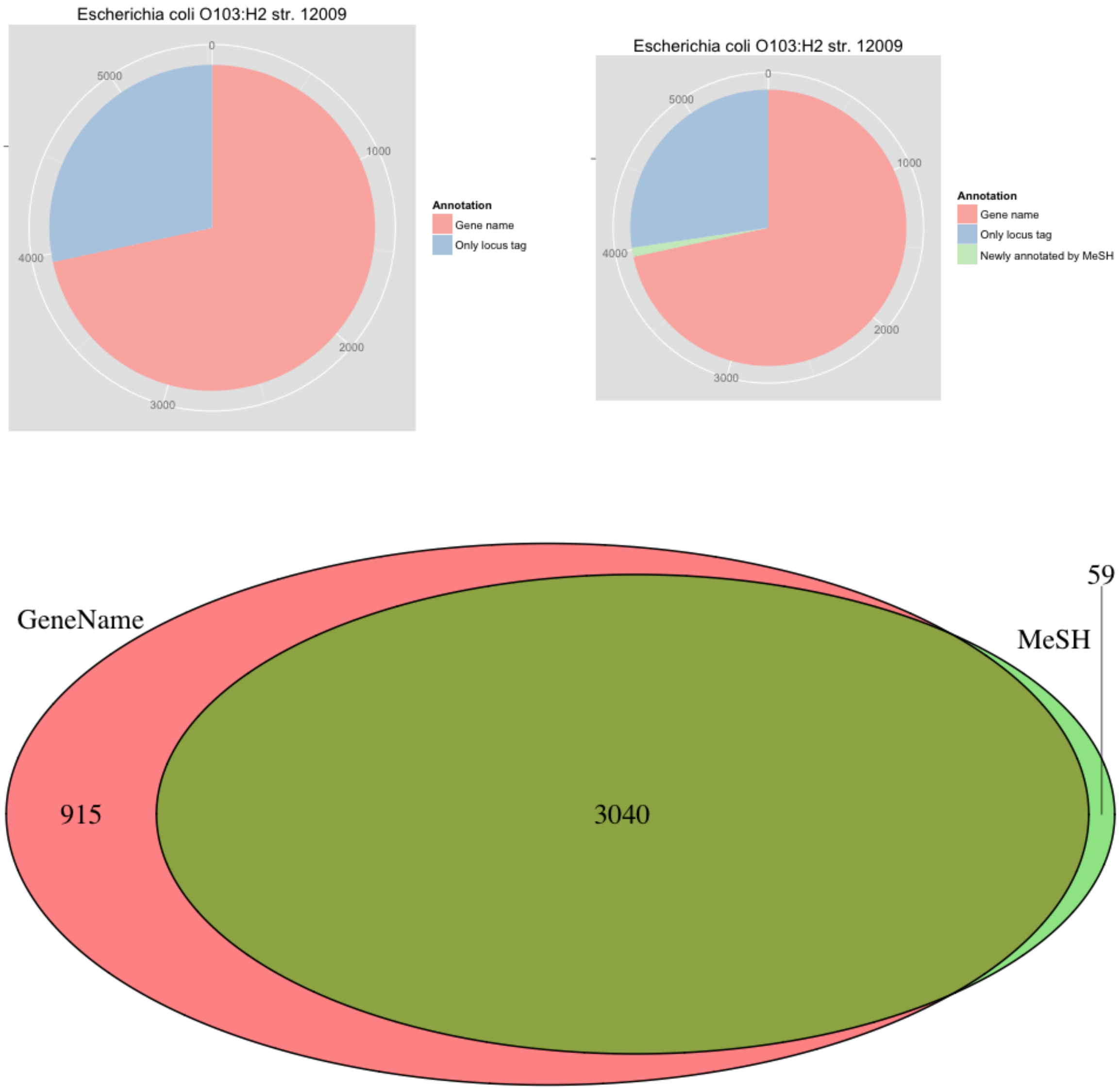

Figure 39: org.MeSH.Eco.O103.H2.12009.db

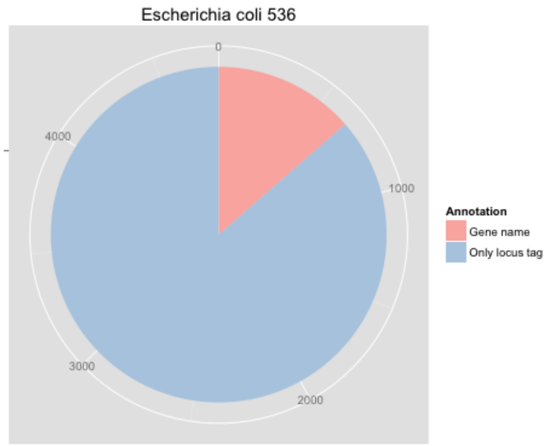

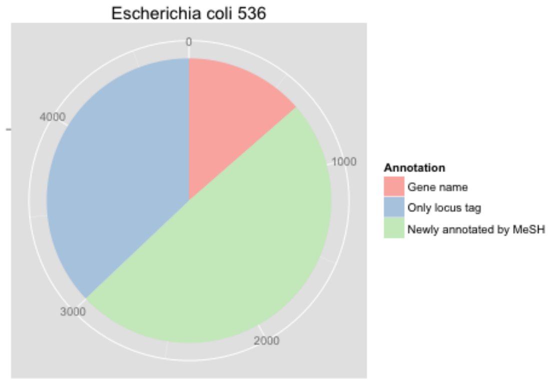

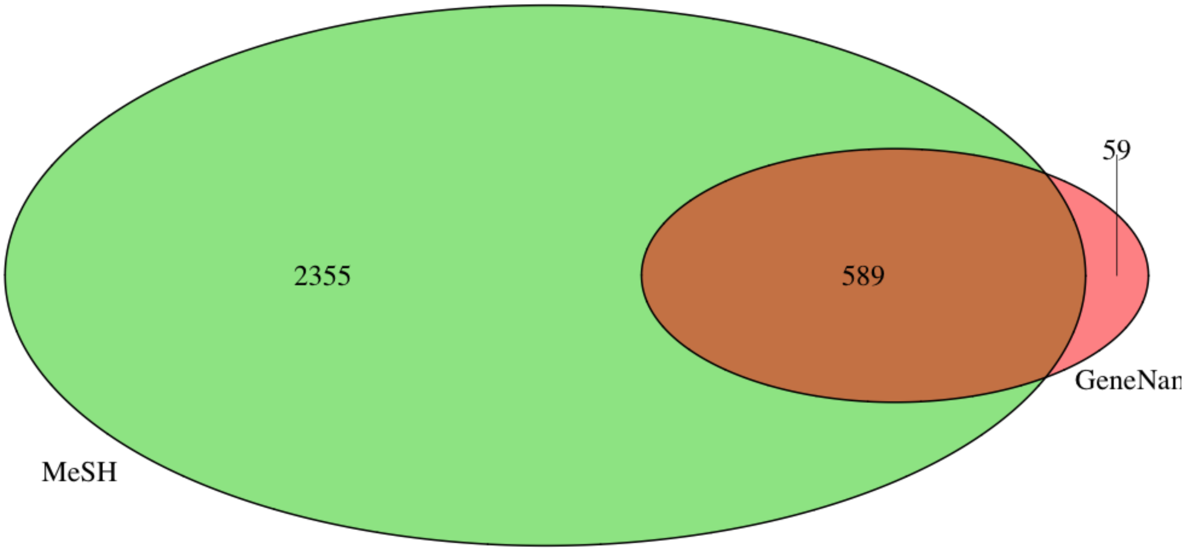

Figure 40: org.MeSH.Eco.536.db

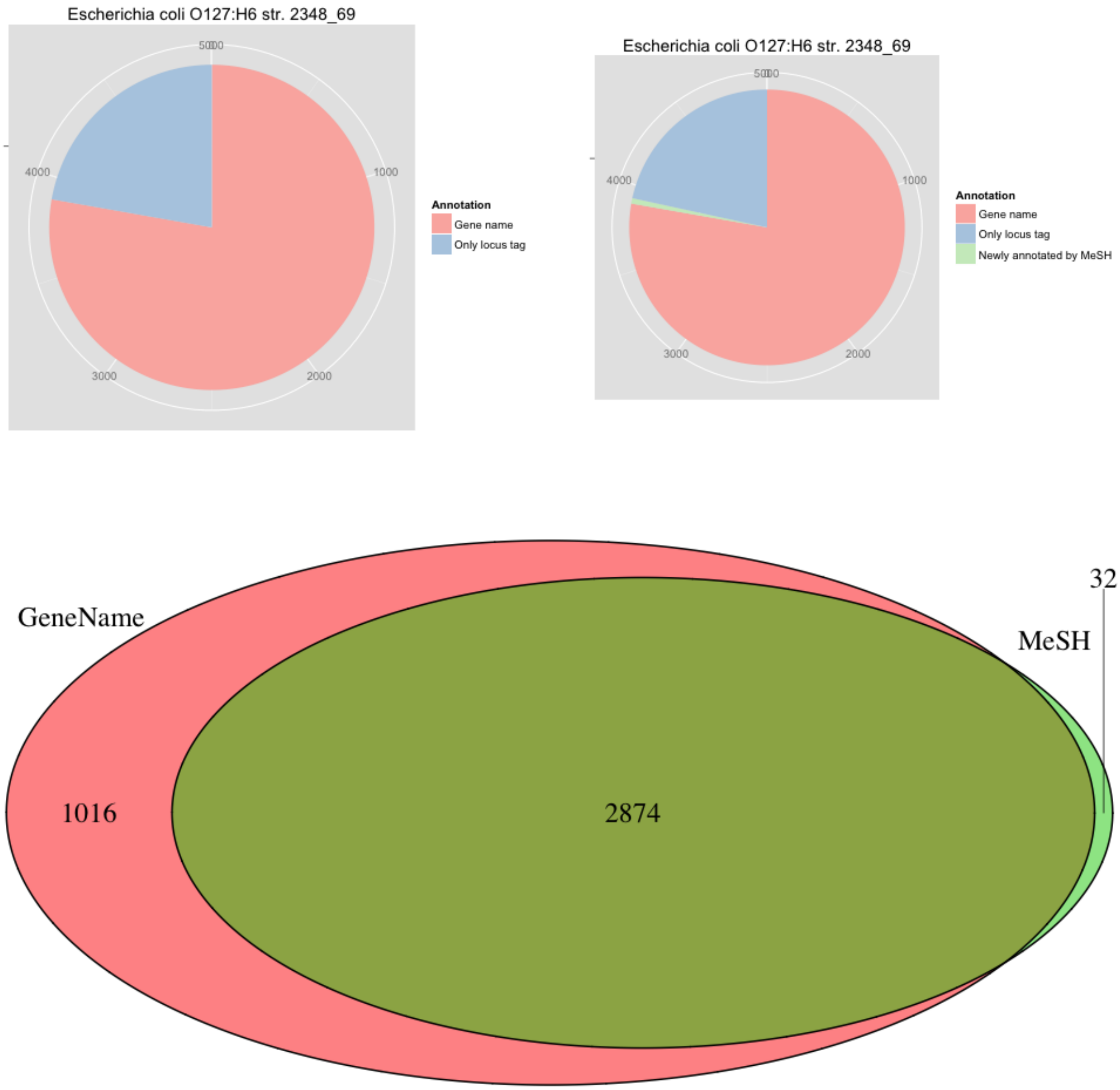

Figure 41: org.MeSH.Eco.O127.H6.E2348.69.db

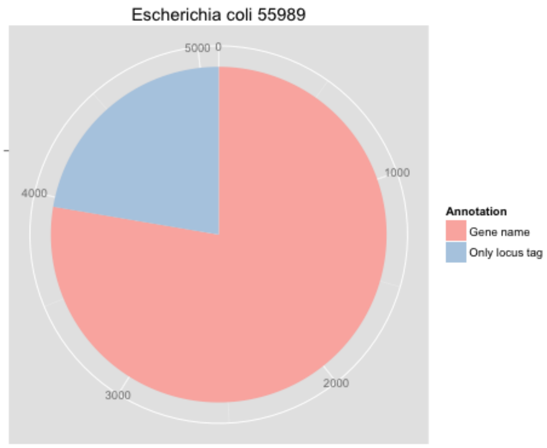

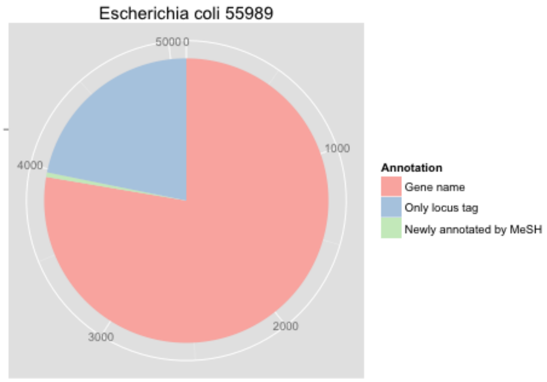

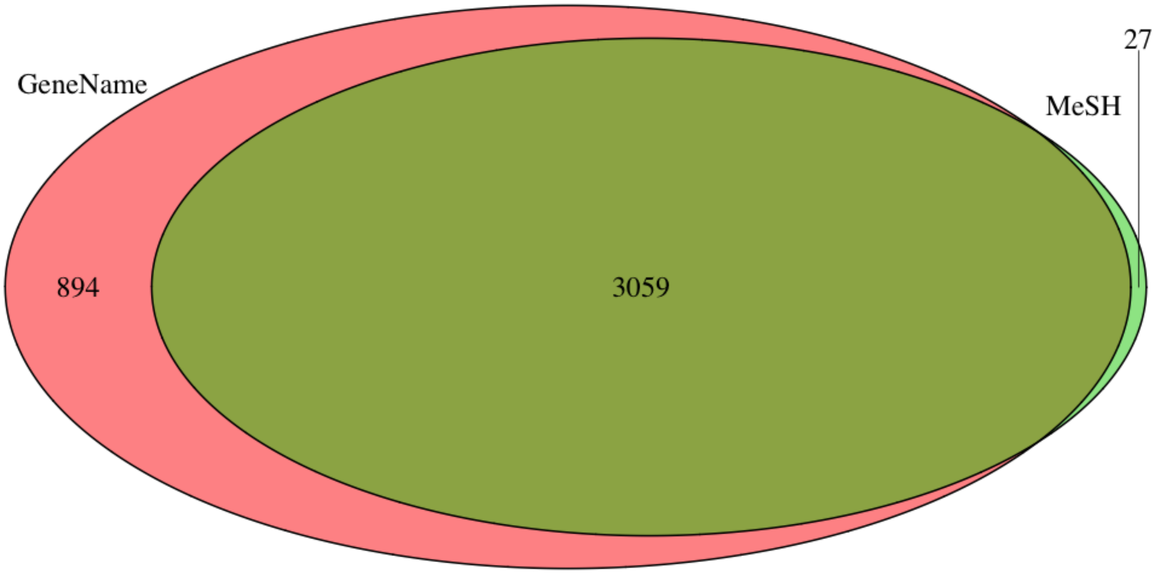

Figure 42: org.MeSH.Eco.55989.db

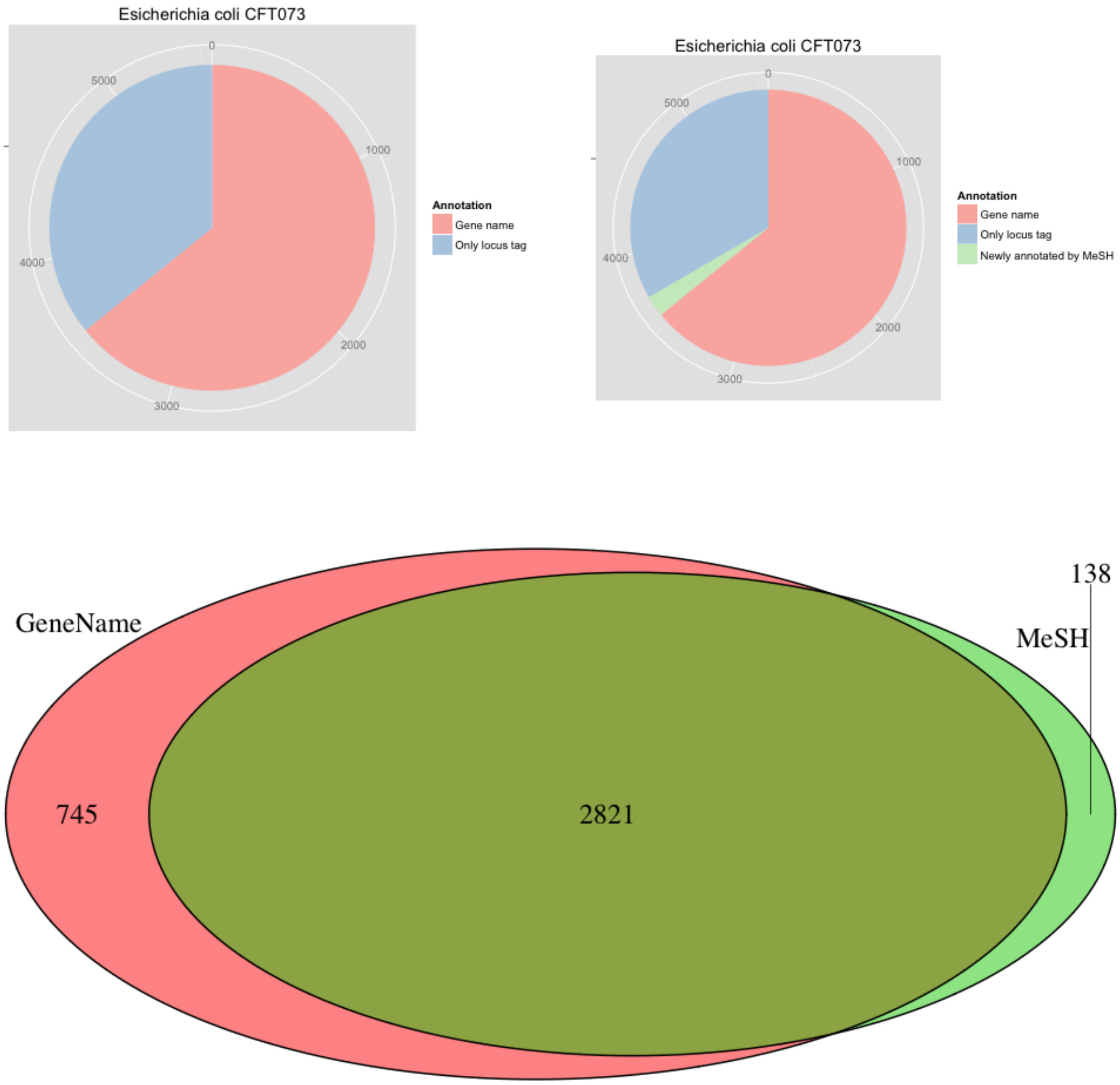

Figure 43: org.MeSH.Eco.CFT073.db

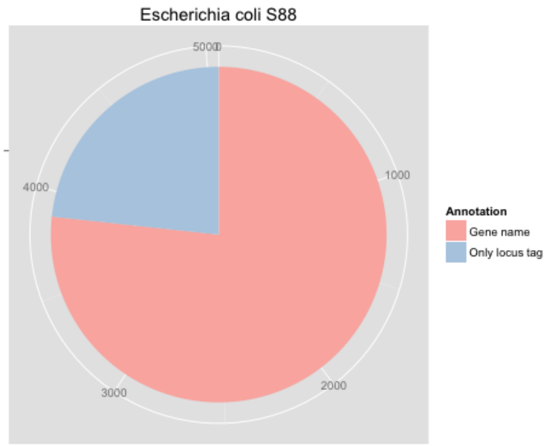

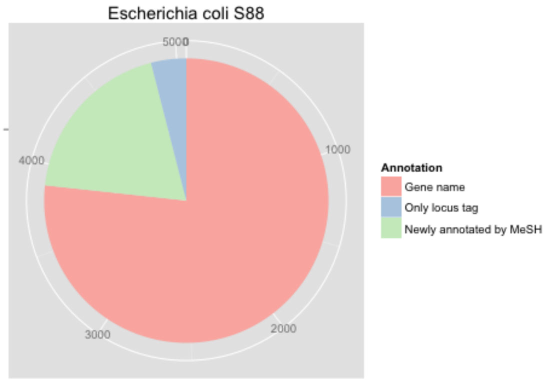

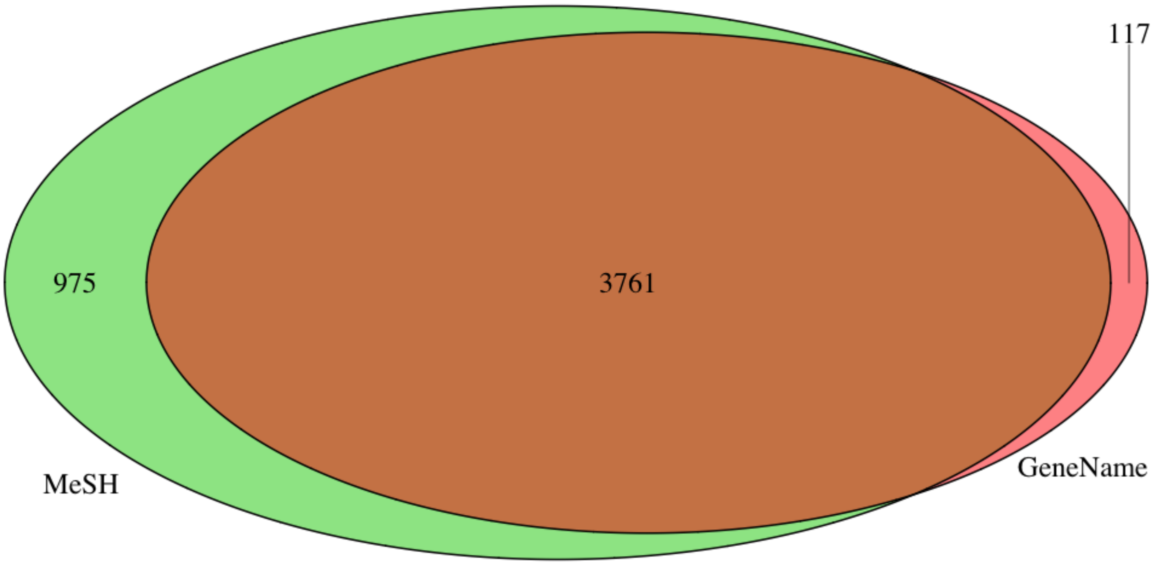

Figure 44: org.MeSH.Eco.S88.db

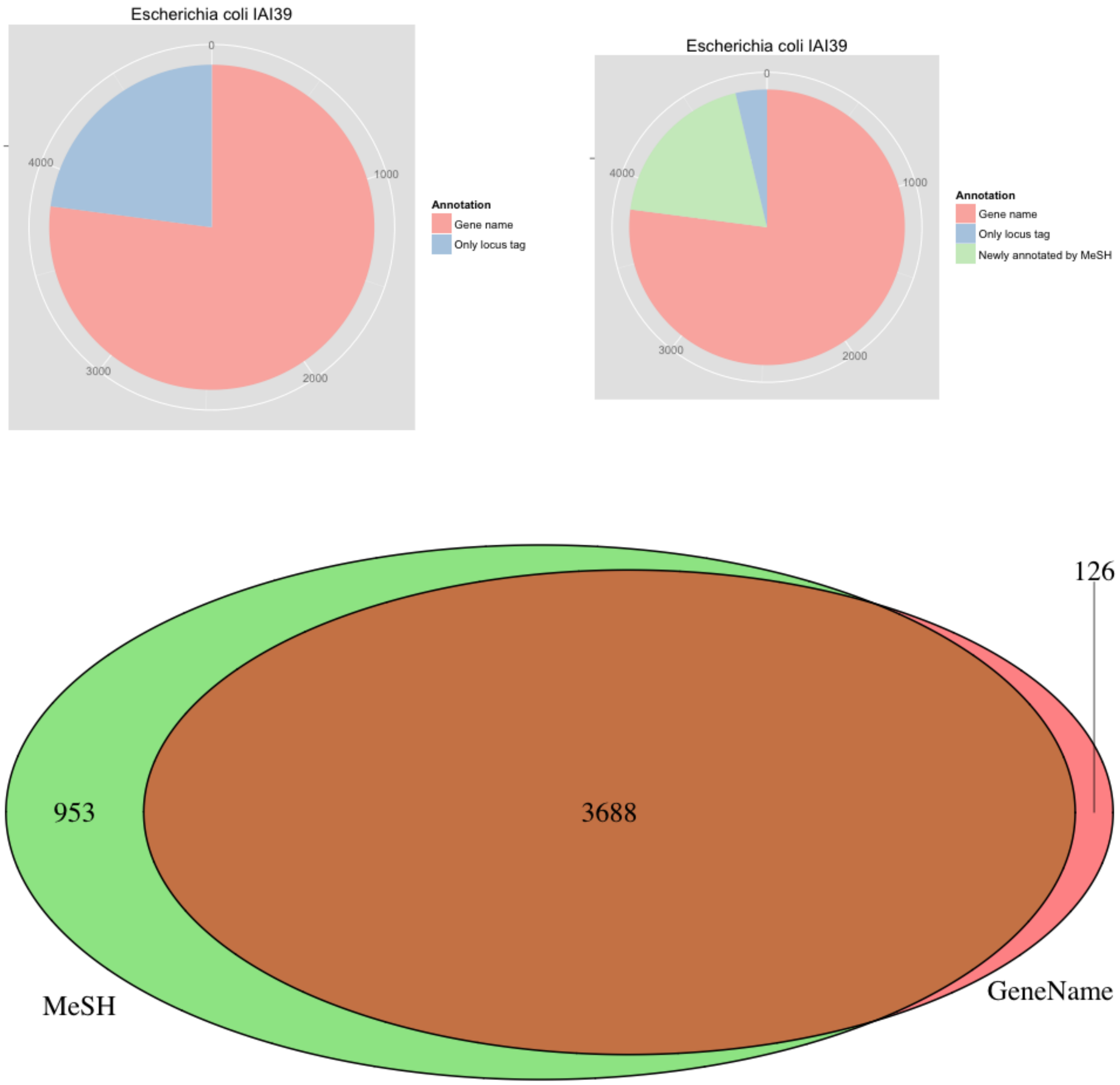

Figure 45: org.MeSH.Eco.IAI39.db

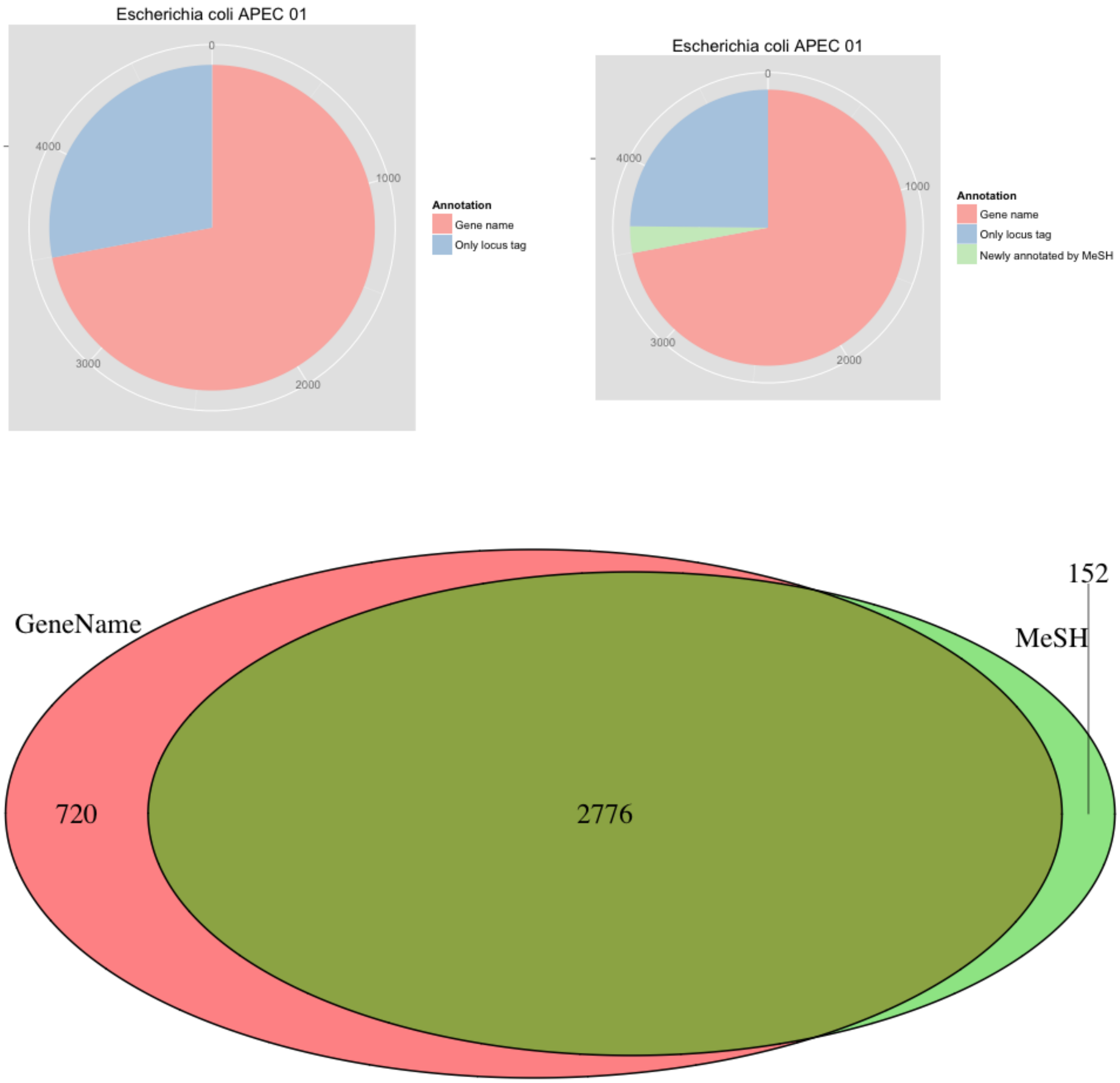

Figure 46: org.MeSH.Eco.APEC01.db

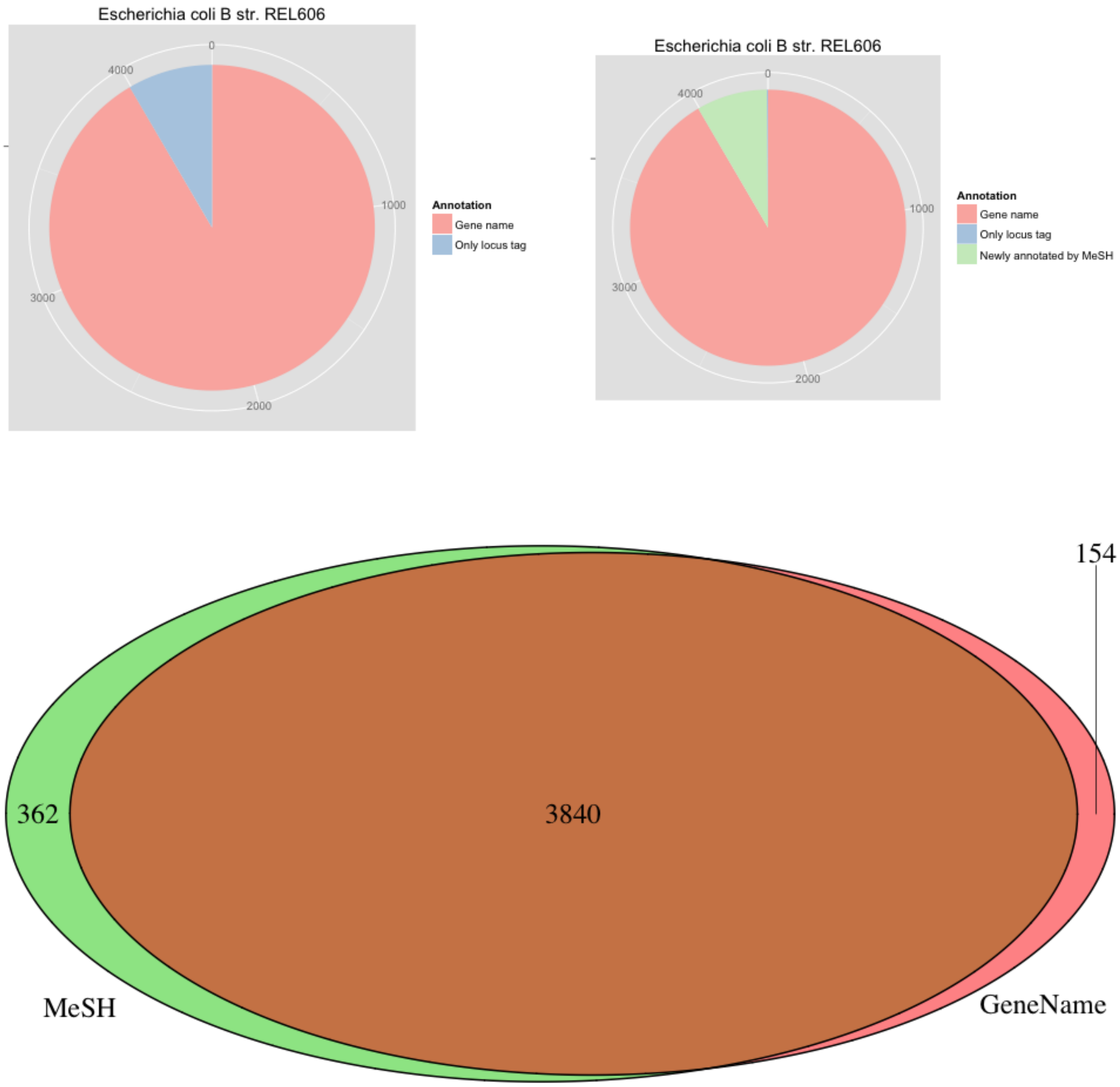

Figure 47: org.MeSH.Eco.B.REL606.db

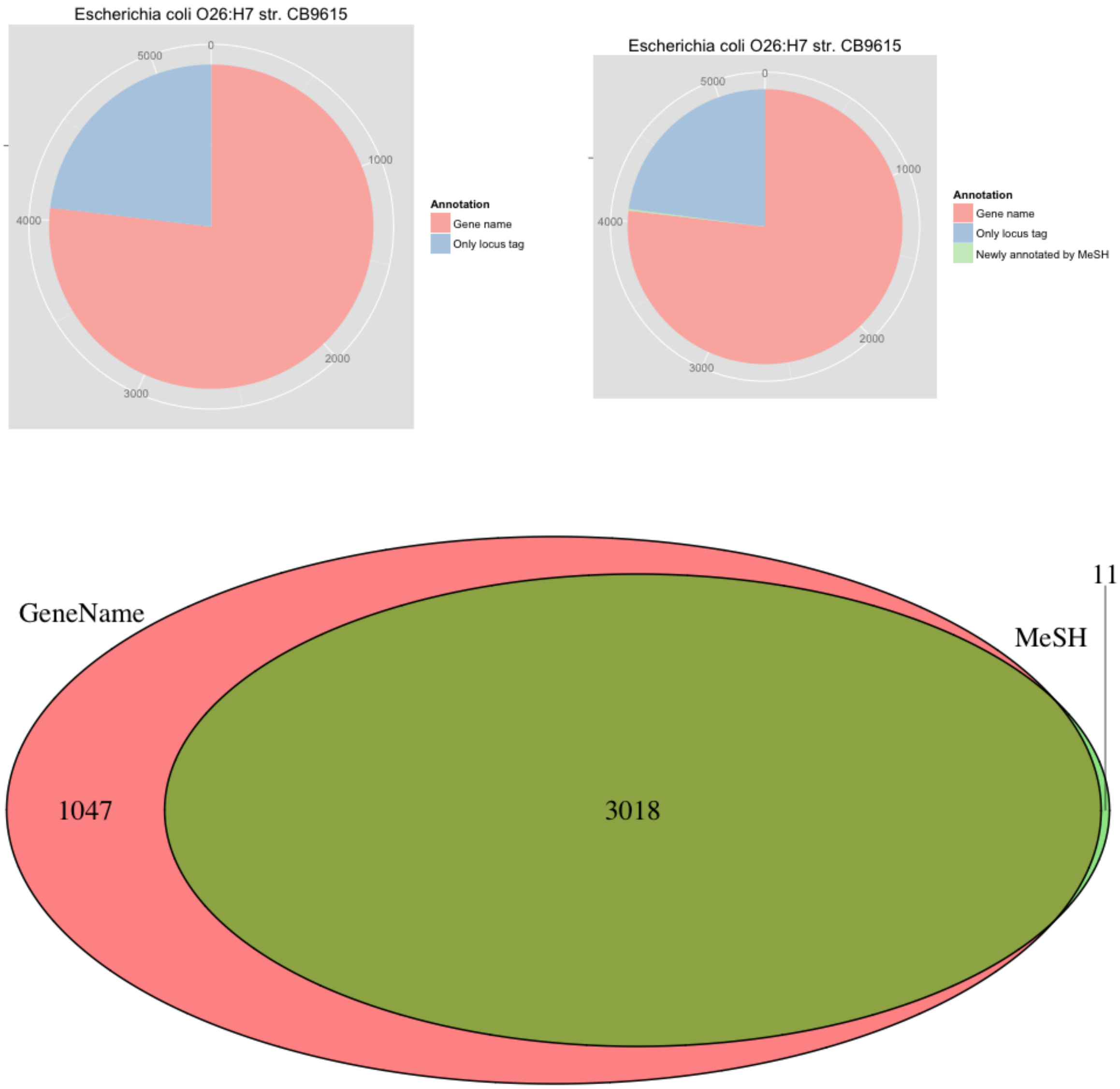

Figure 48: org.MeSH.Eco.O26.H7.CB9615.db

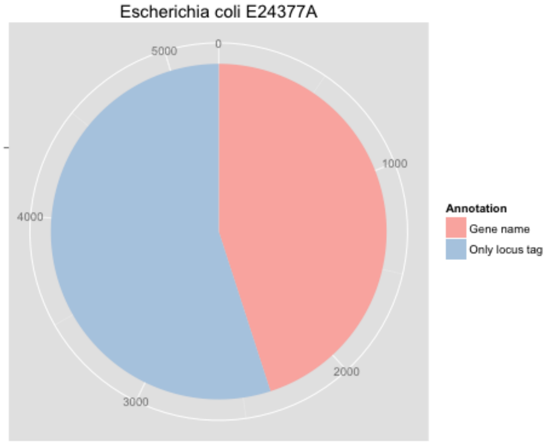

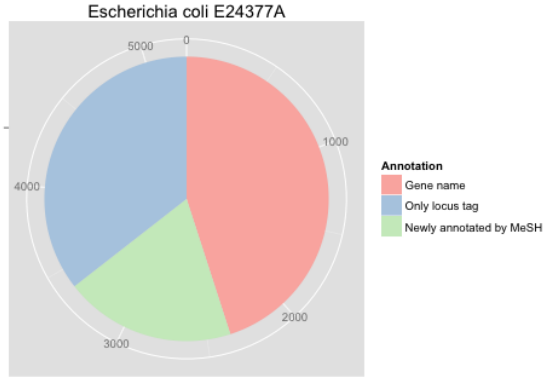

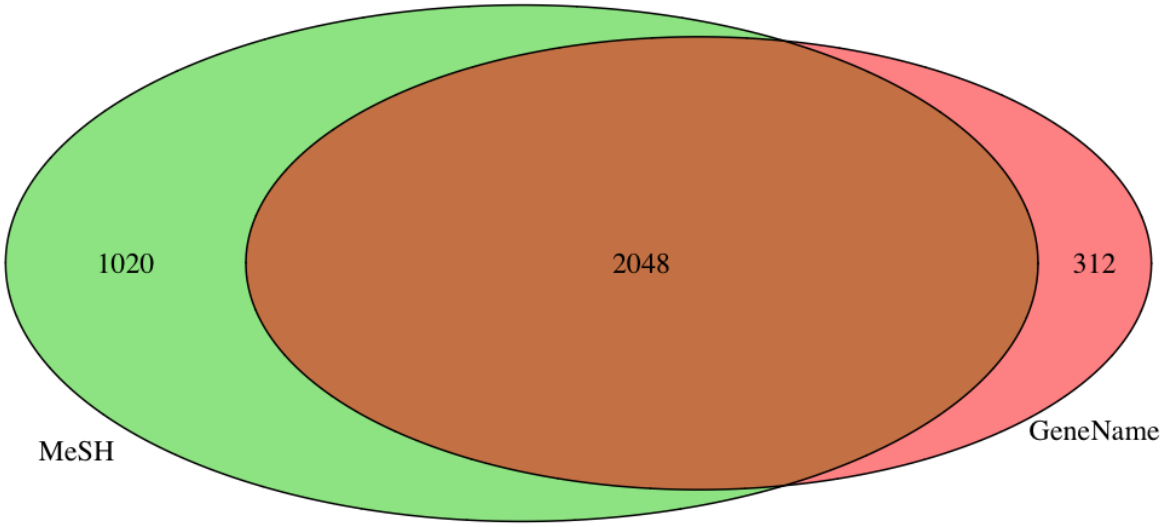

Figure 49: org.MeSH.Eco.E24377A.db

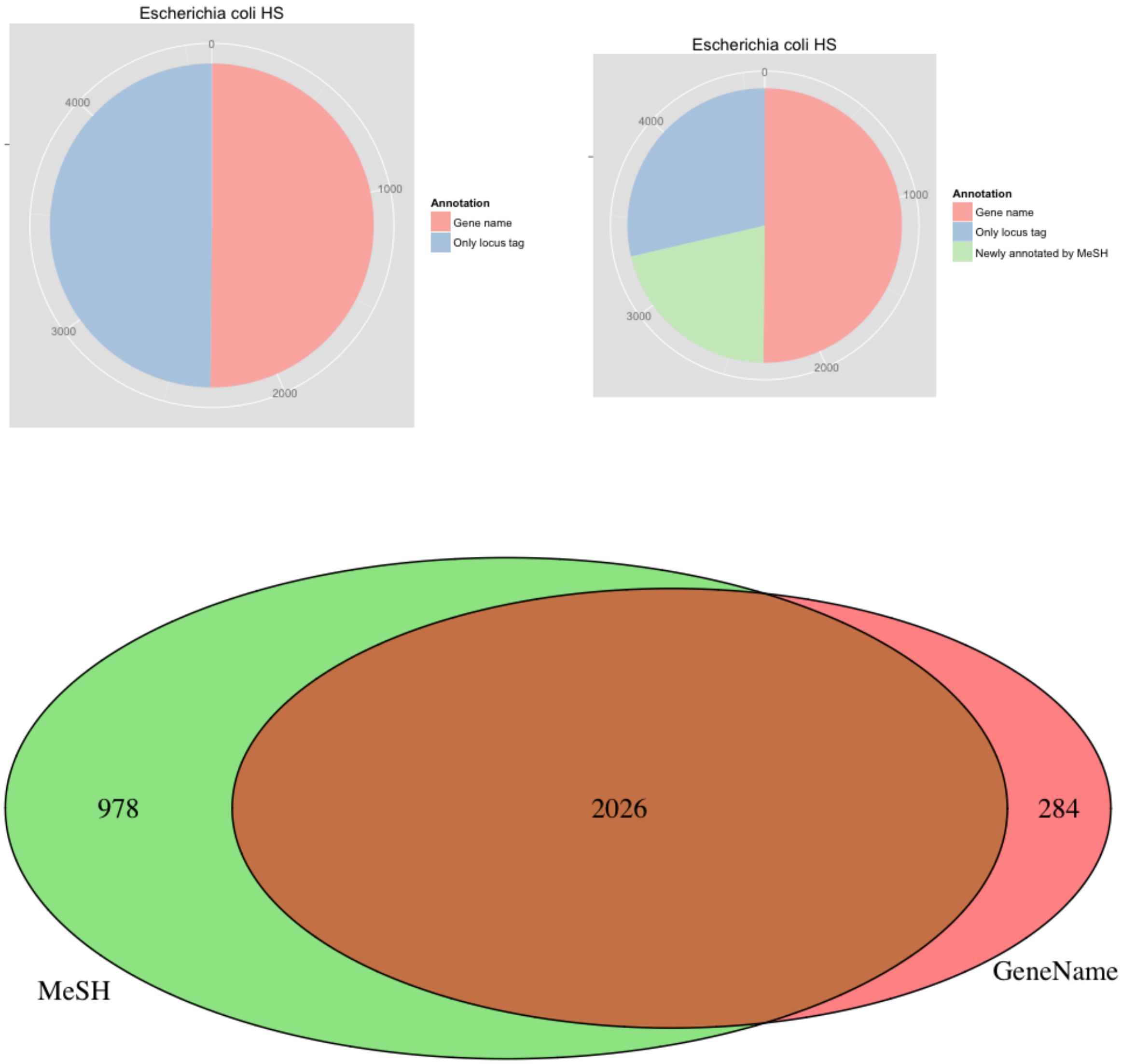

Figure 50: org.MeSH.Eco.HS.db

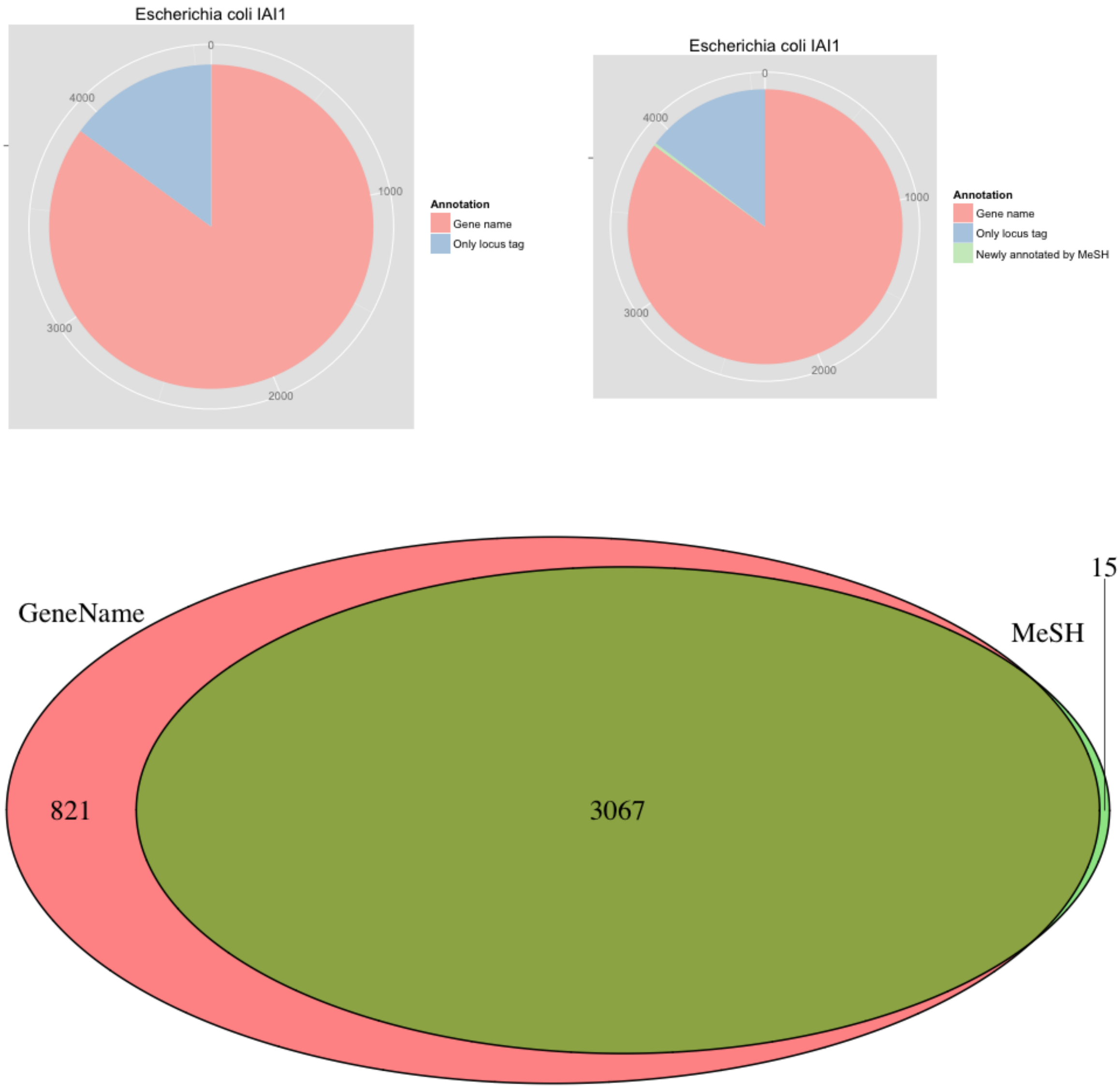

Figure 51: org.MeSH.Eco.IAI1.db

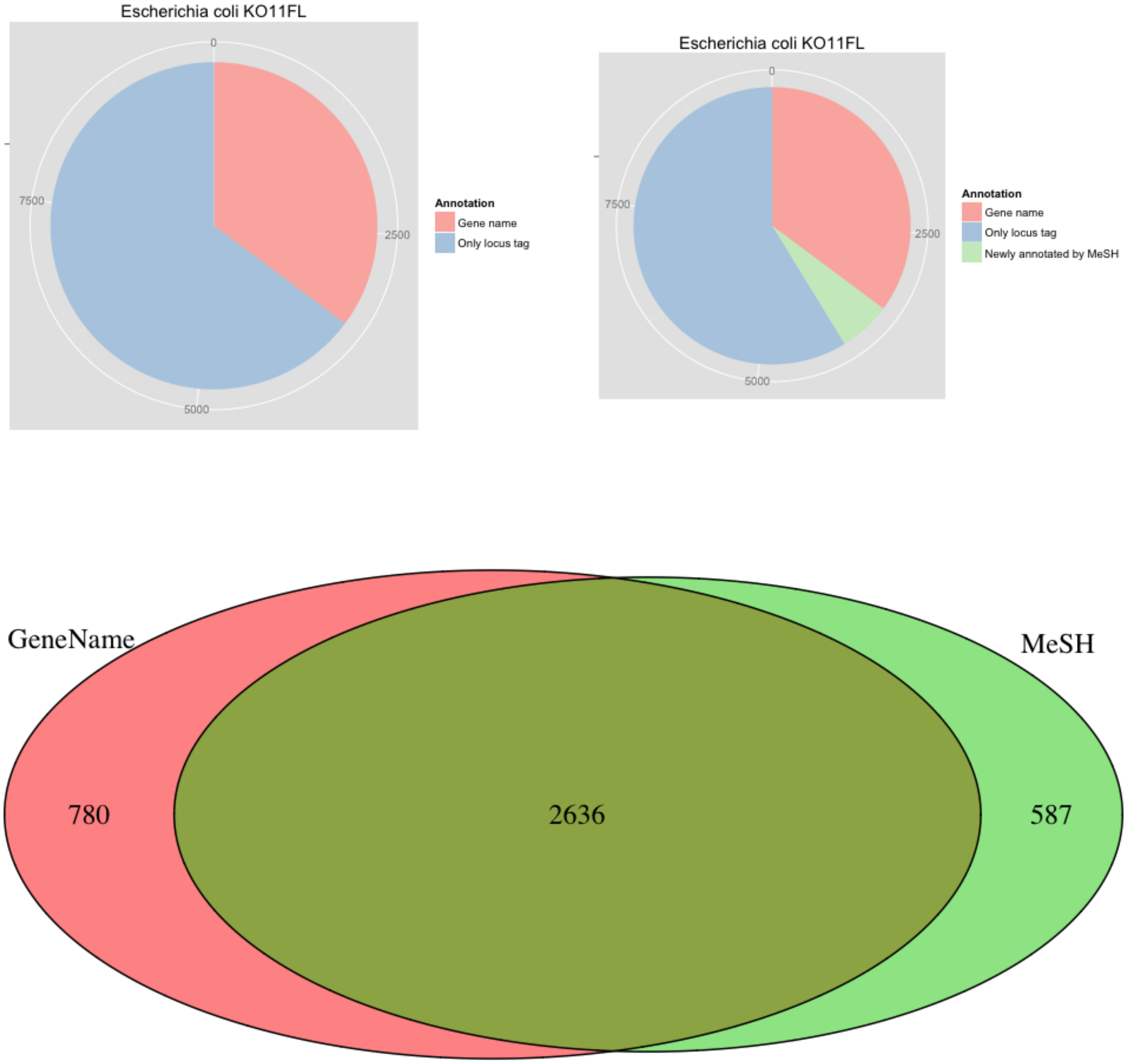

Figure 52: org.MeSH.Eco.KO11FL.db

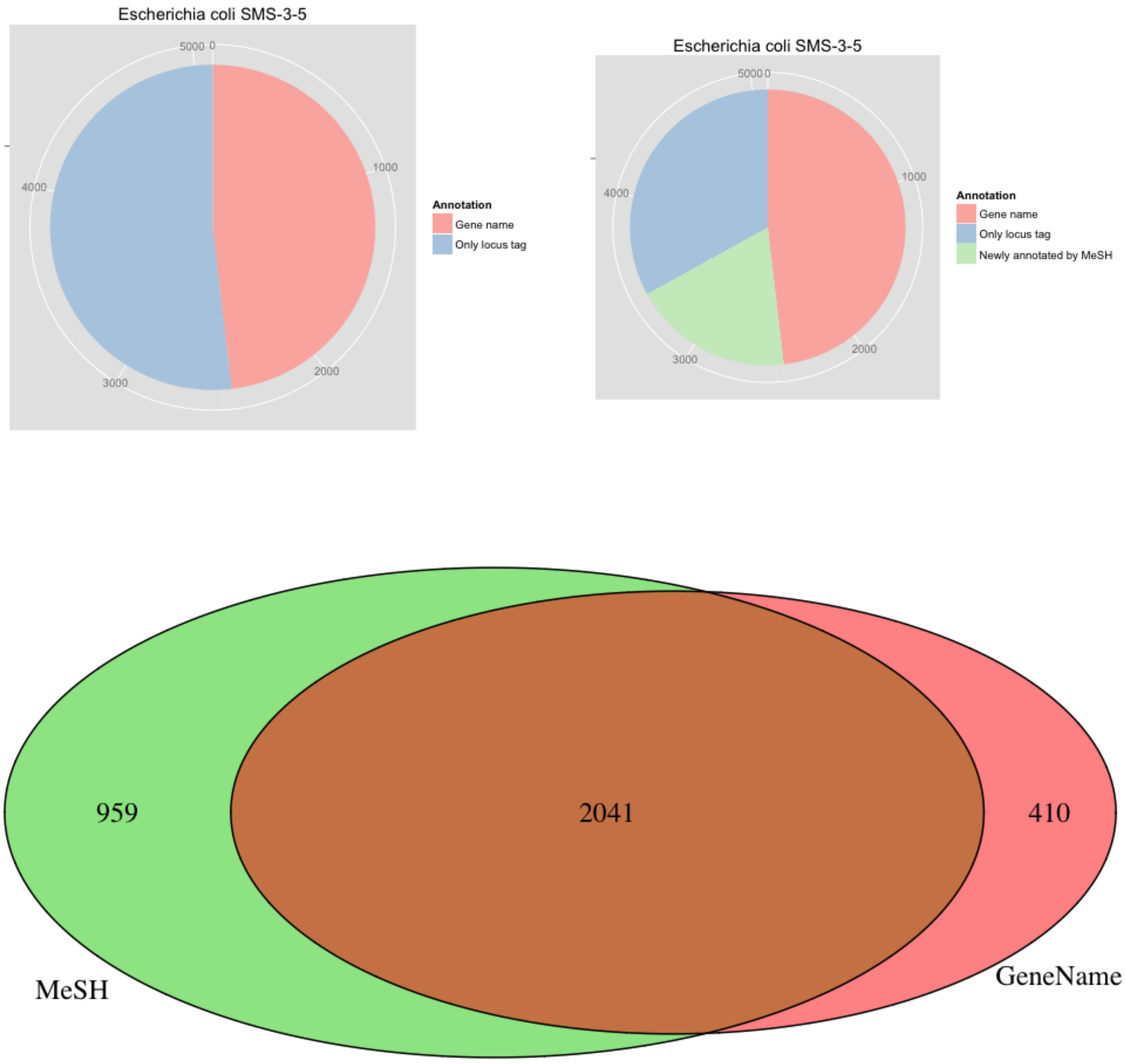

Figure 53: org.MeSH.Eco.SMS35.db

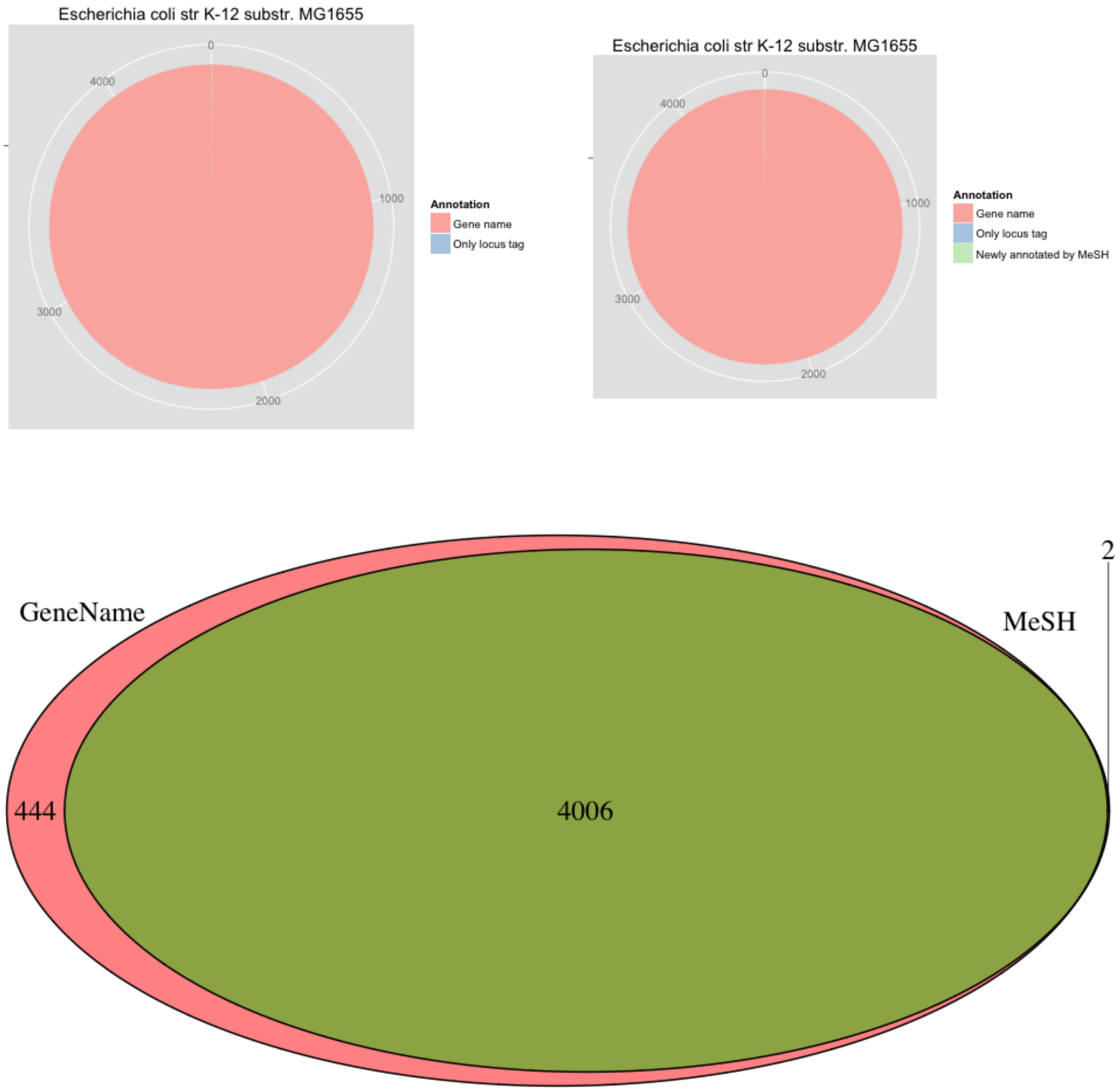

Figure 54: org.MeSH.Eco.K12.MG1655.db

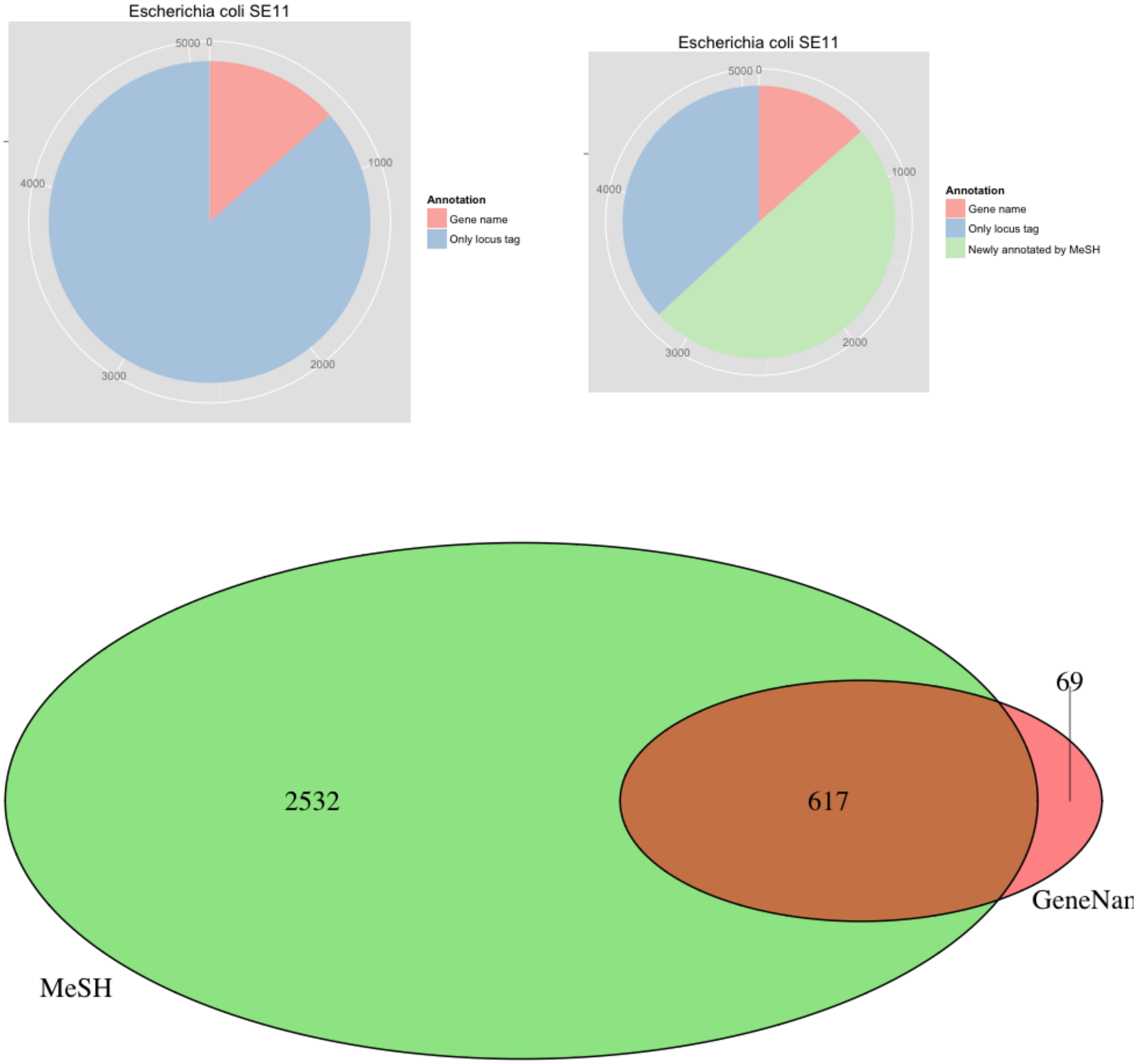

Figure 55: org.MeSH.Eco.SE11.db

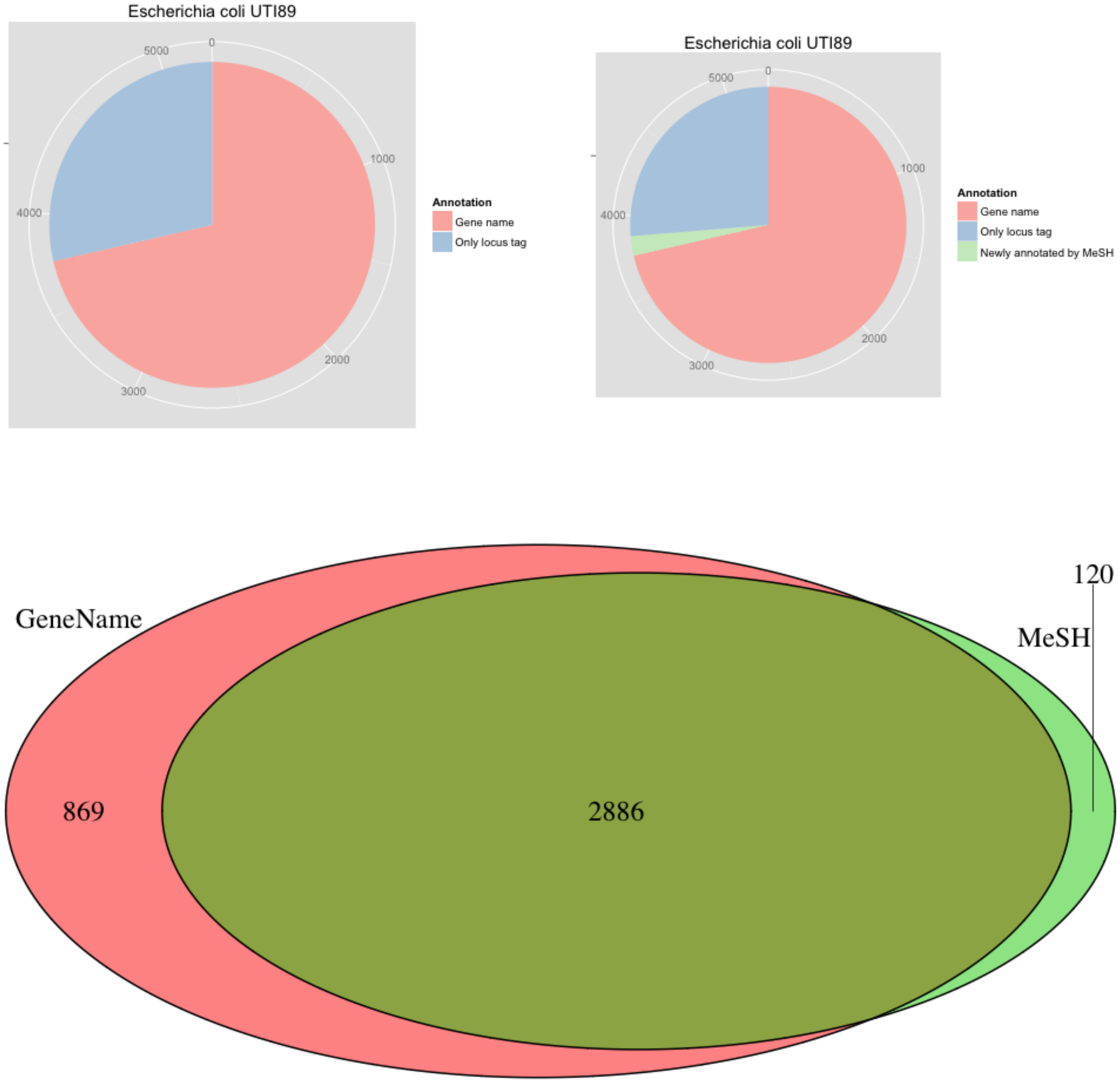

Figure 56: org.MeSH.Eco.UTI89.db

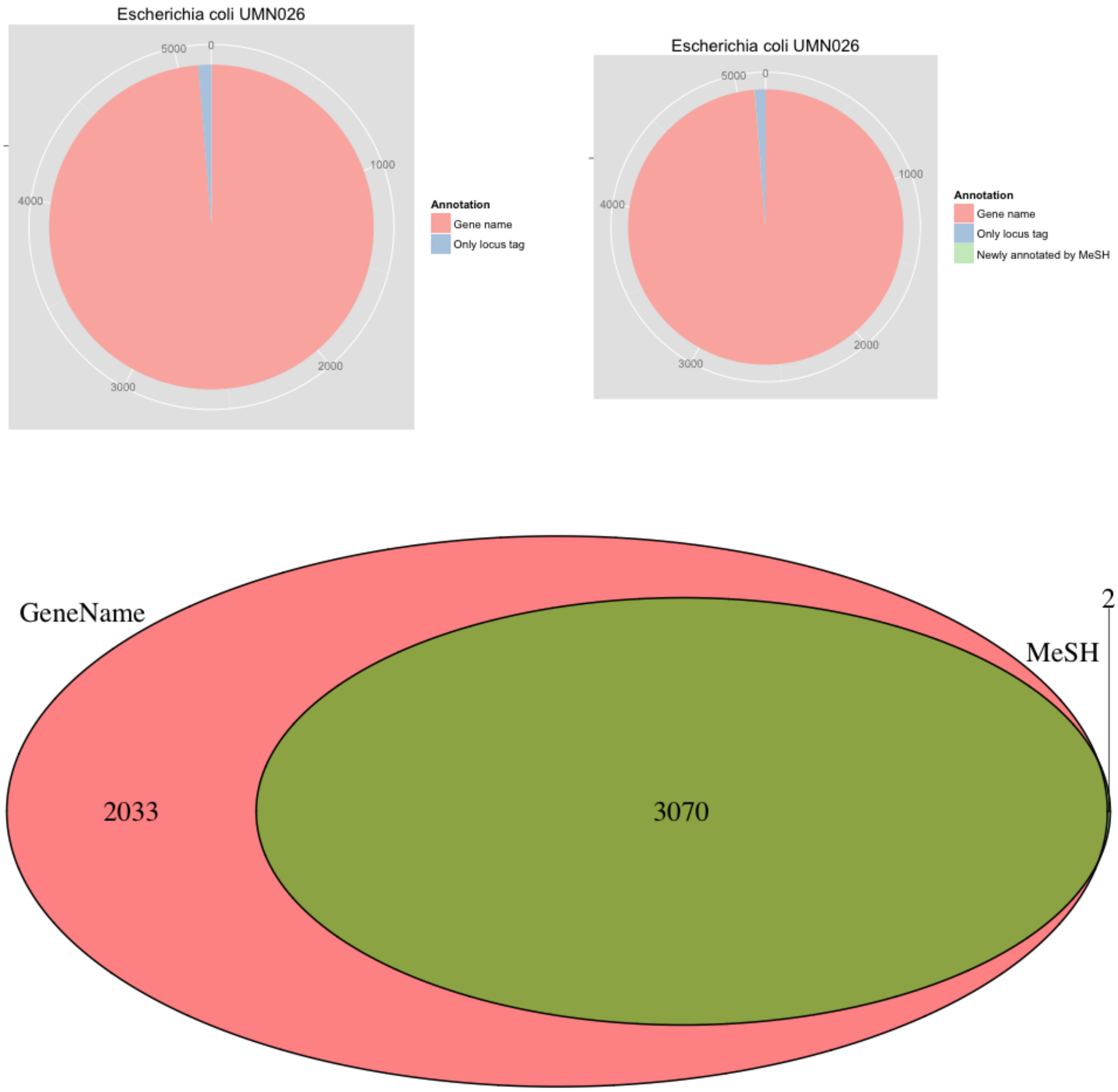

Figure 57: org.MeSH.Eco.UMN026.db

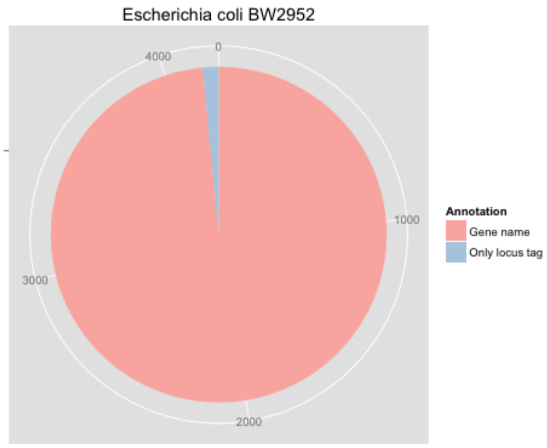

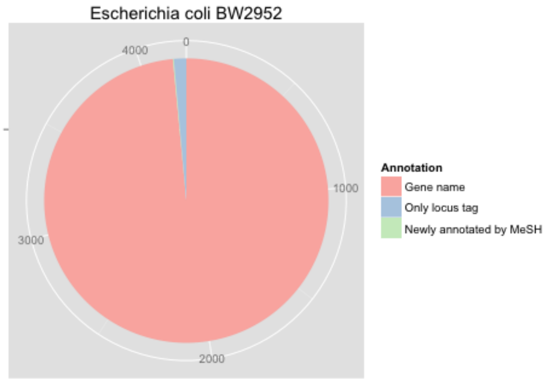

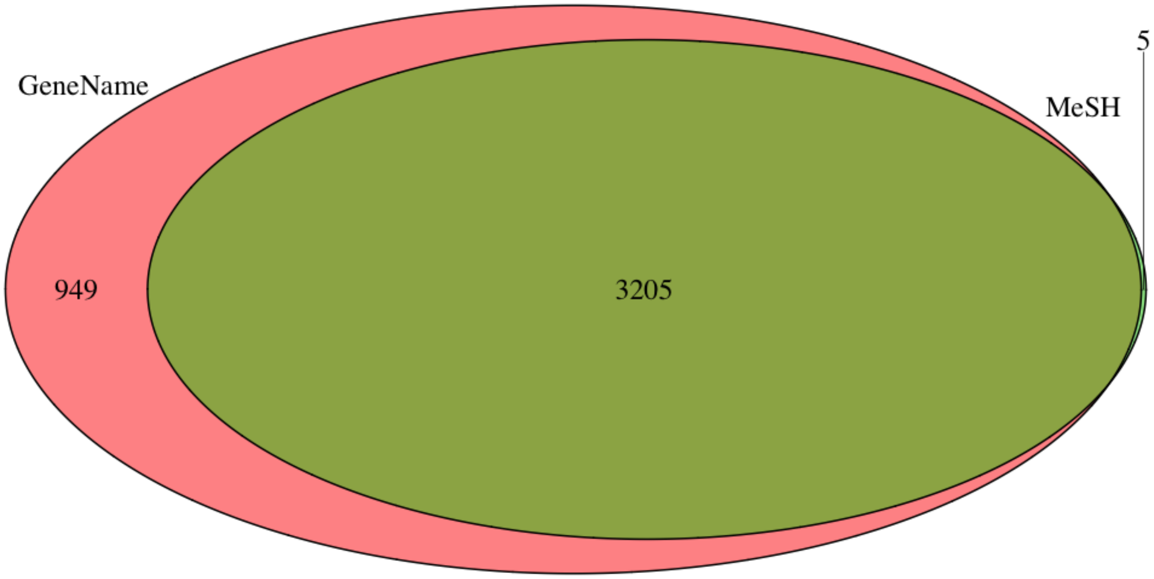

Figure 58: org.MeSH.Eco.BW2952.db

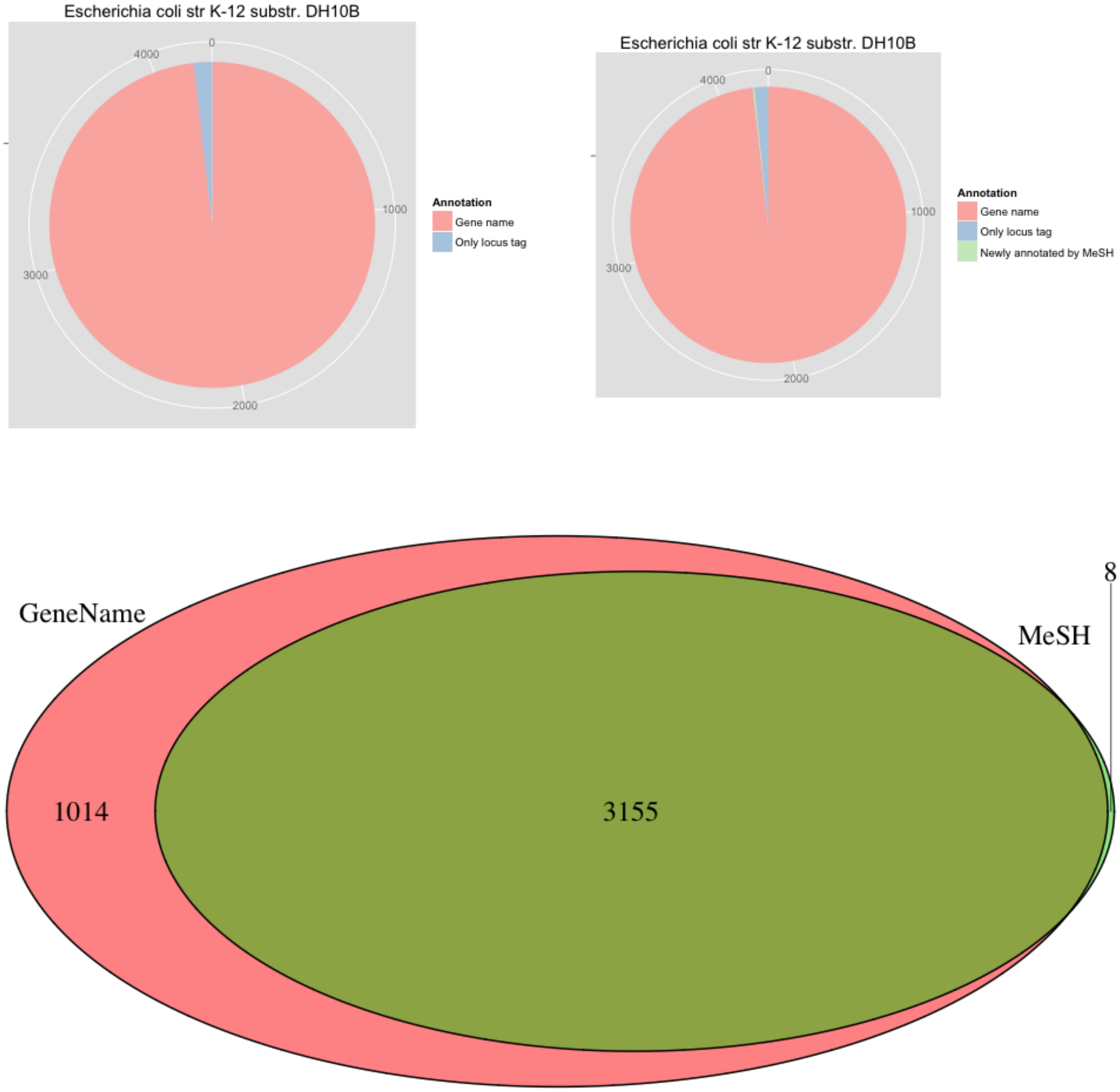

Figure 59: org.MeSH.Eco.K12.DH10B.db

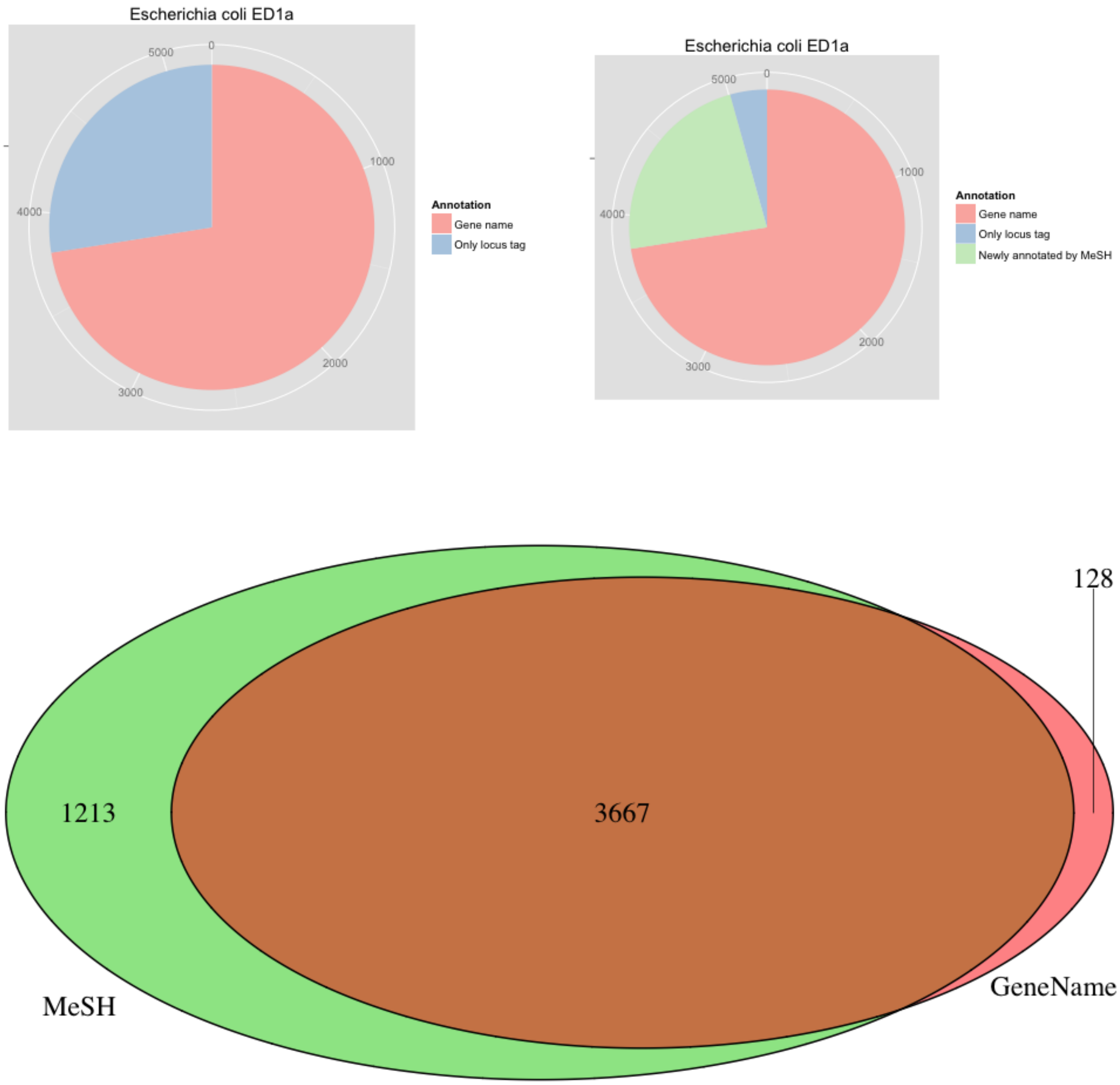

Figure 60: org.MeSH.Eco.ED1a.db

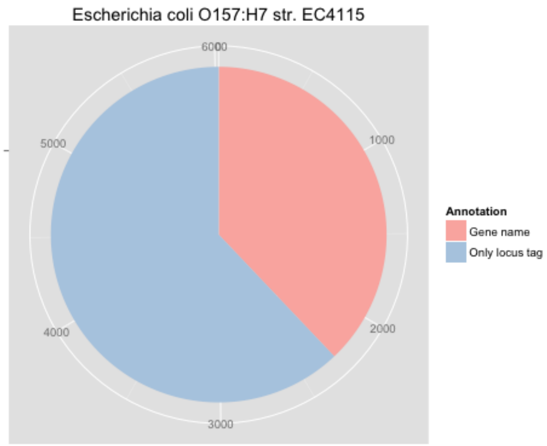

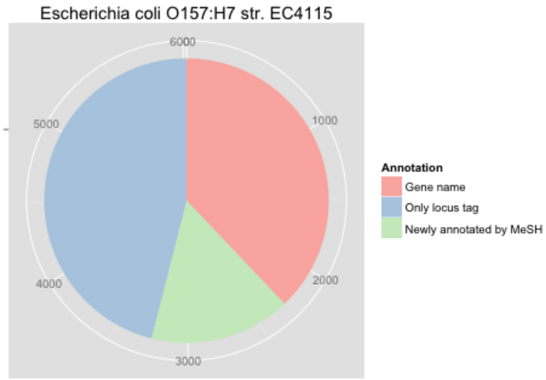

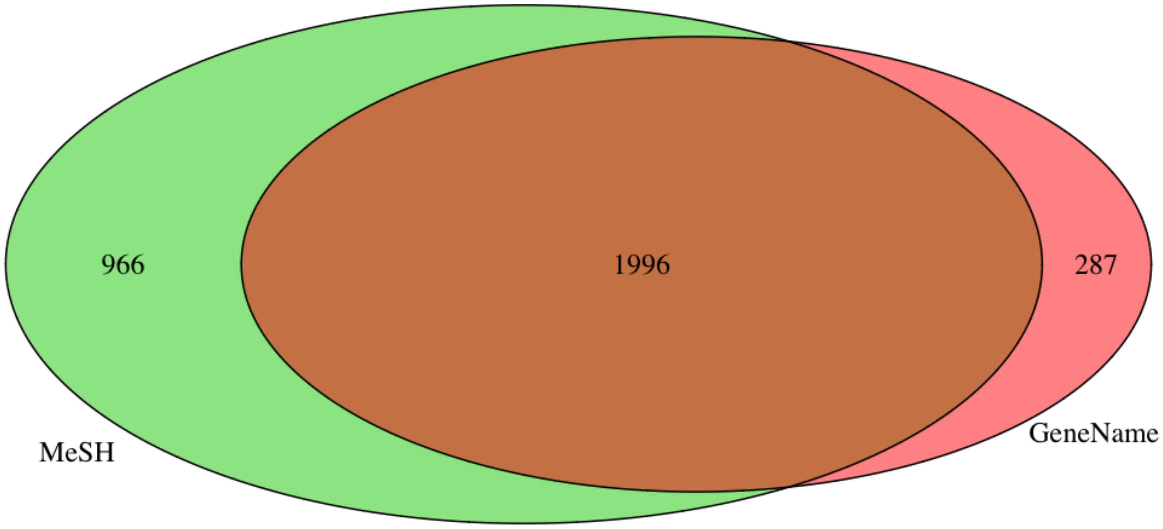

Figure 61: org.MeSH.Eco.O157.H7.EC4115.db

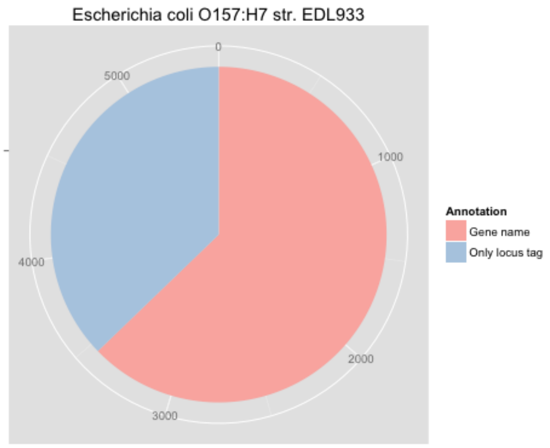

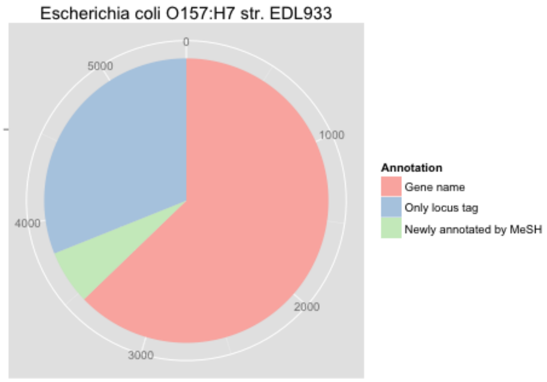

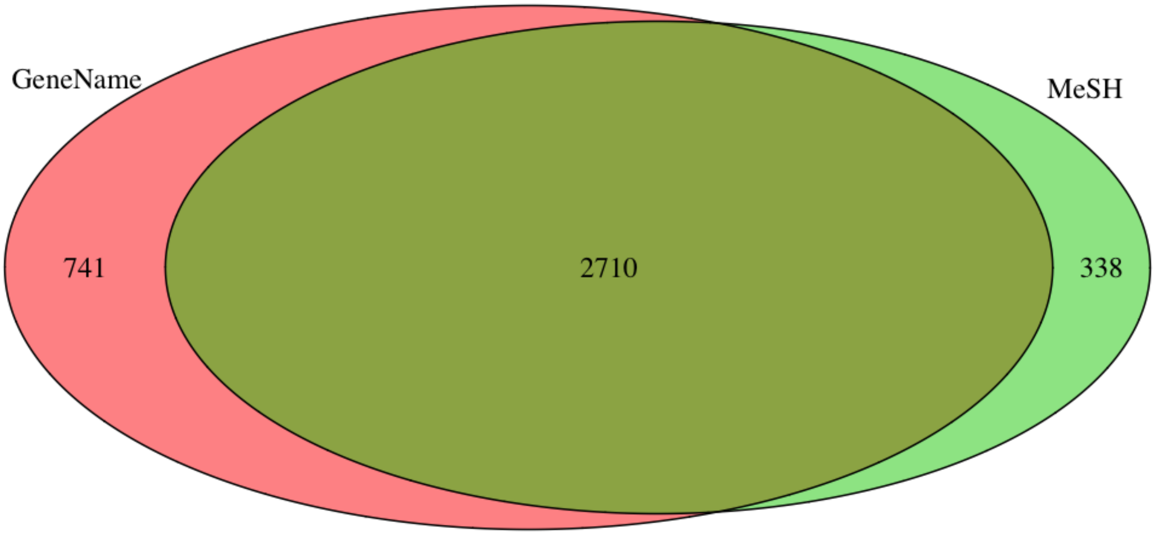

Figure 62: org.MeSH.Eco.O157.H7.EDL933.db

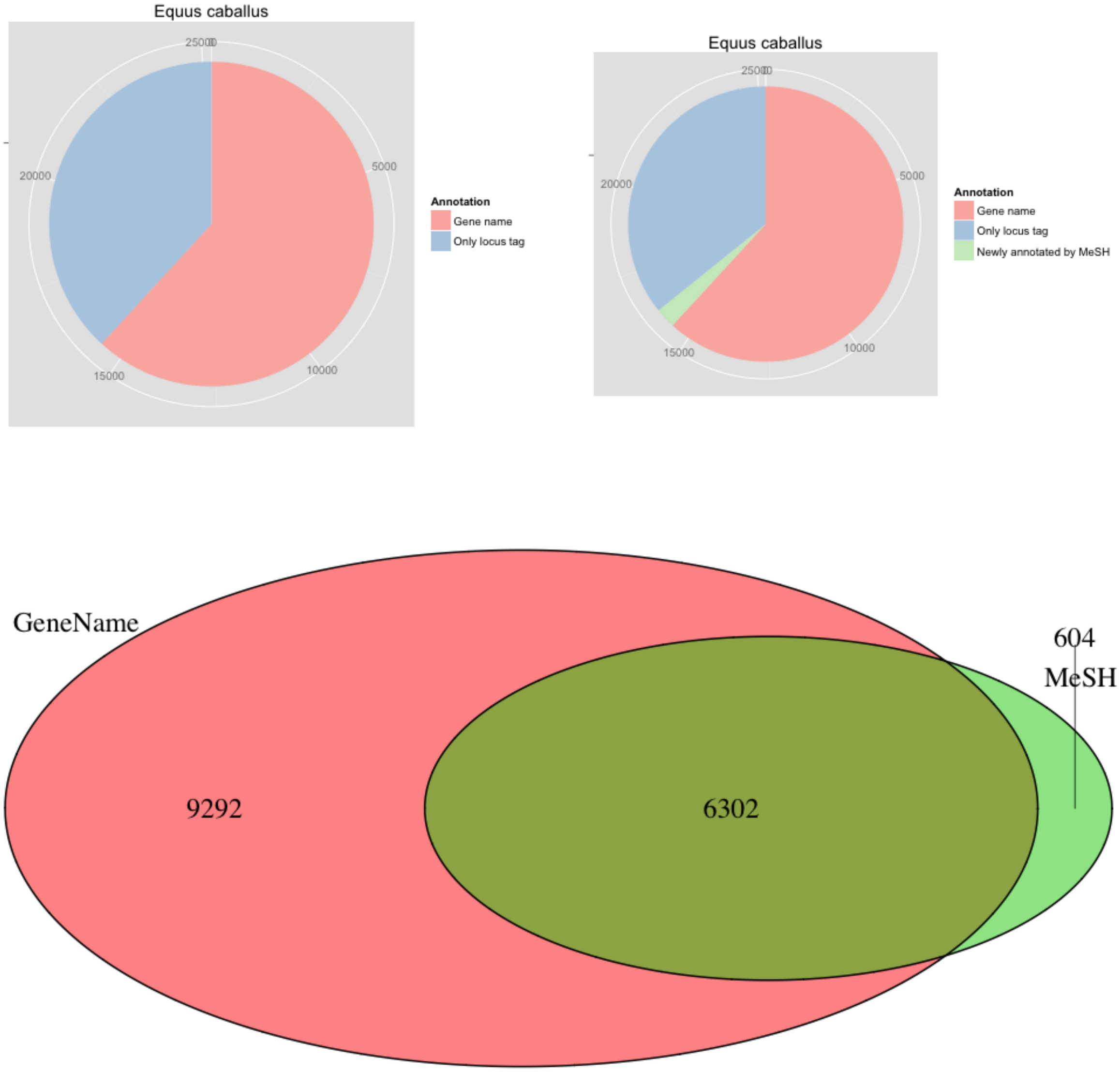

Figure 63: org.MeSH.Eqc.db

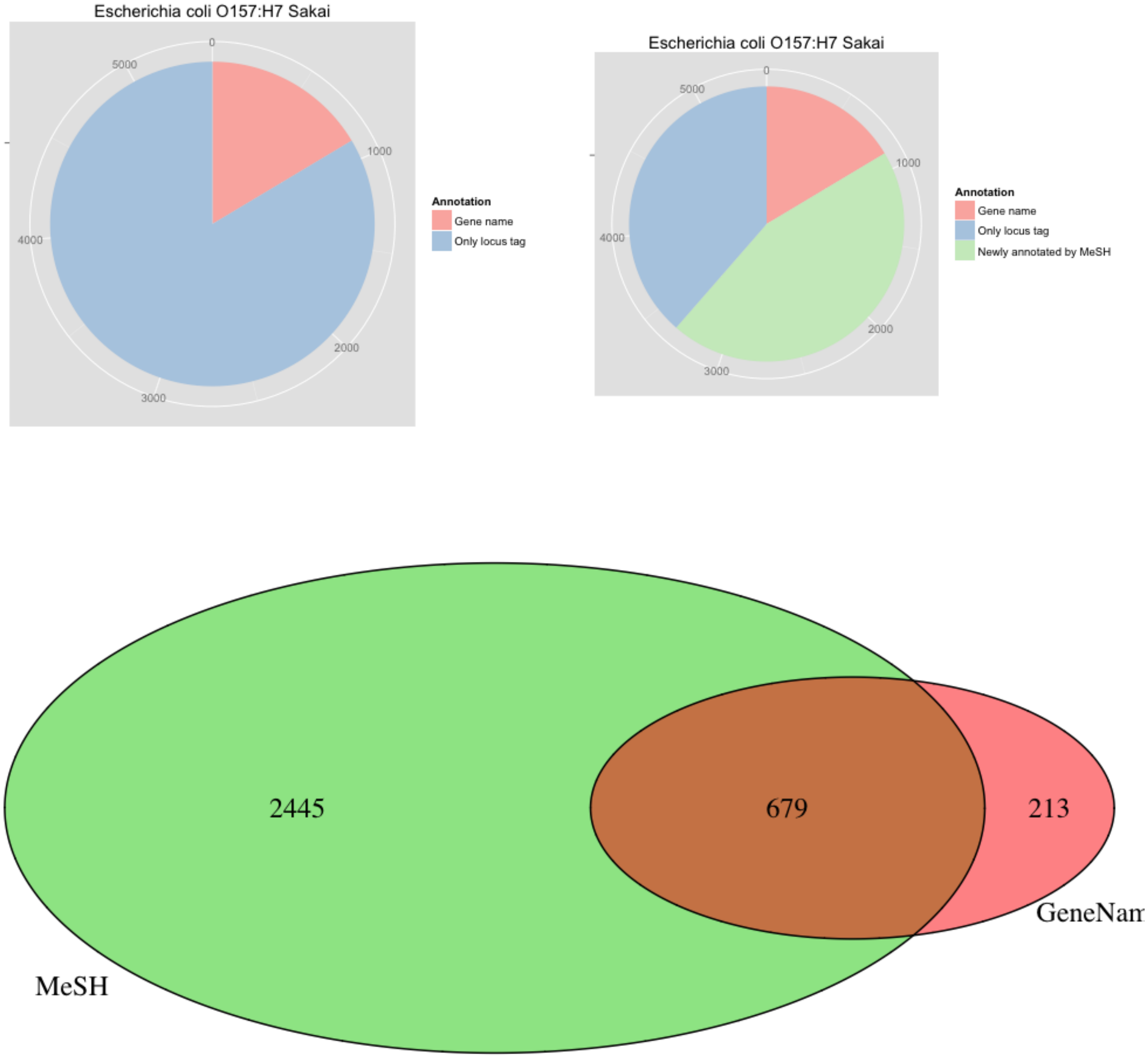

Figure 64: org.MeSH.Eco.O157.H7.Sakai.db

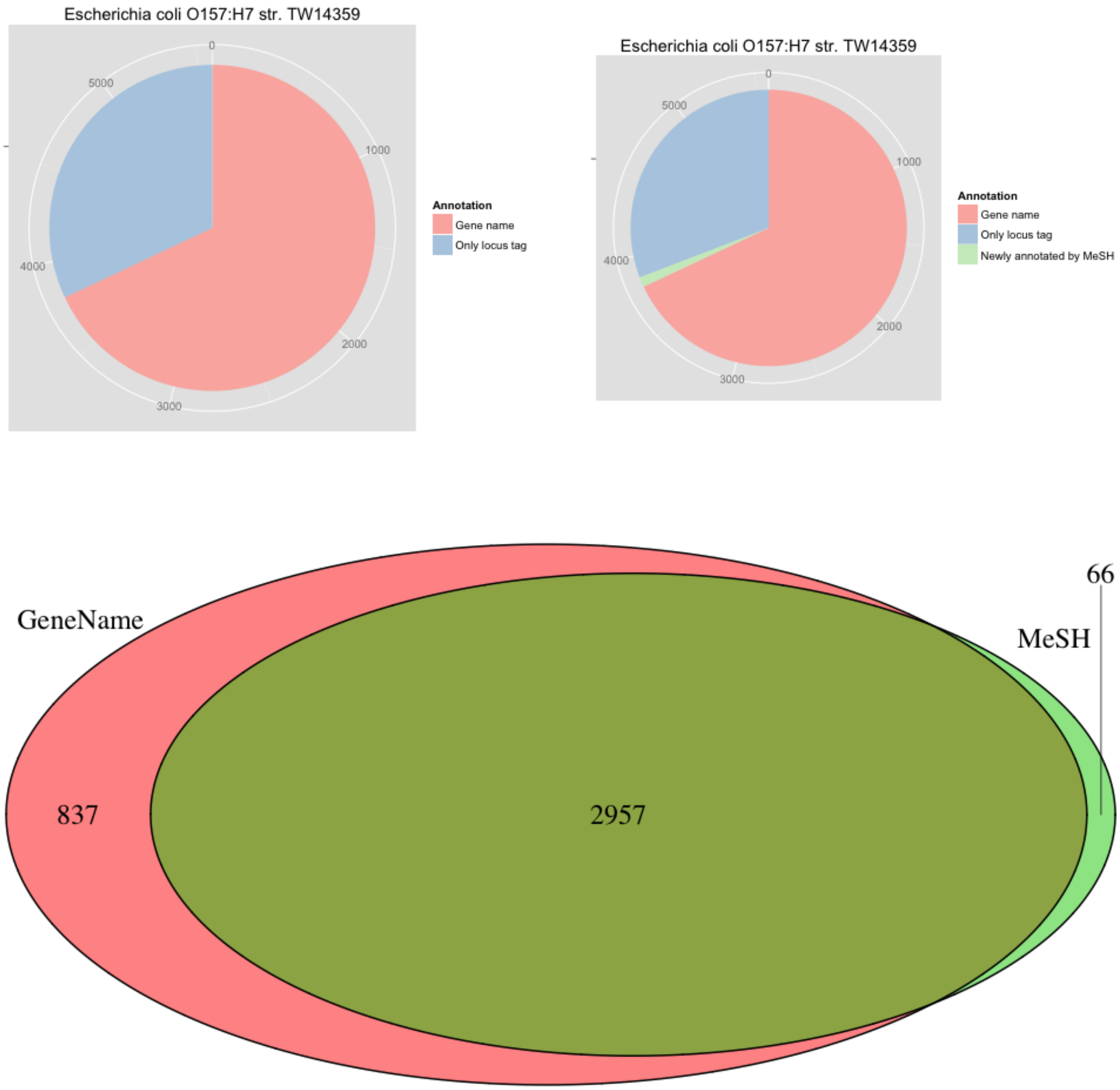

Figure 65: org.MeSH.Eco.O157.H7.TW14359.db

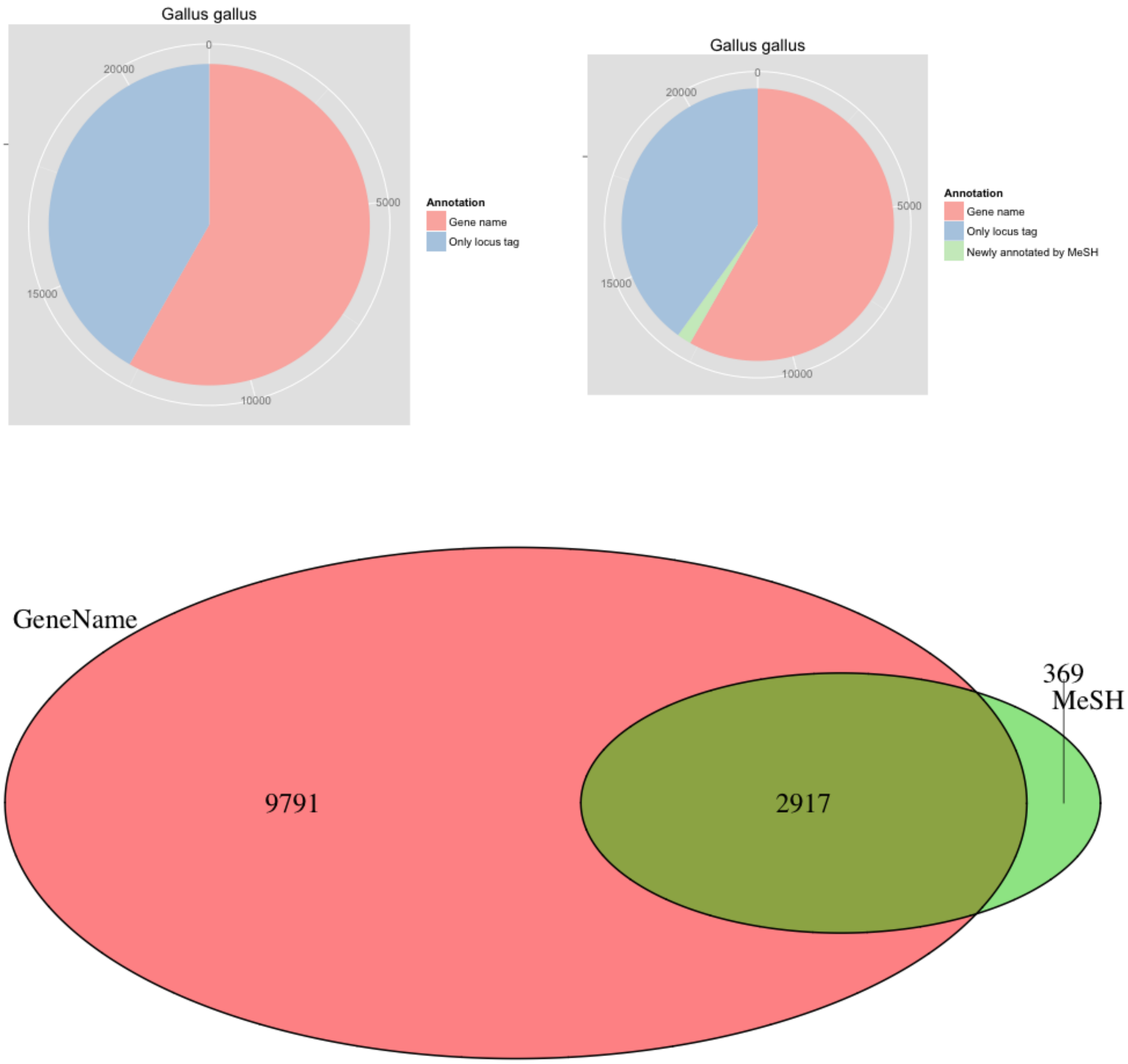

Figure 66: org.MeSH.Gga.db

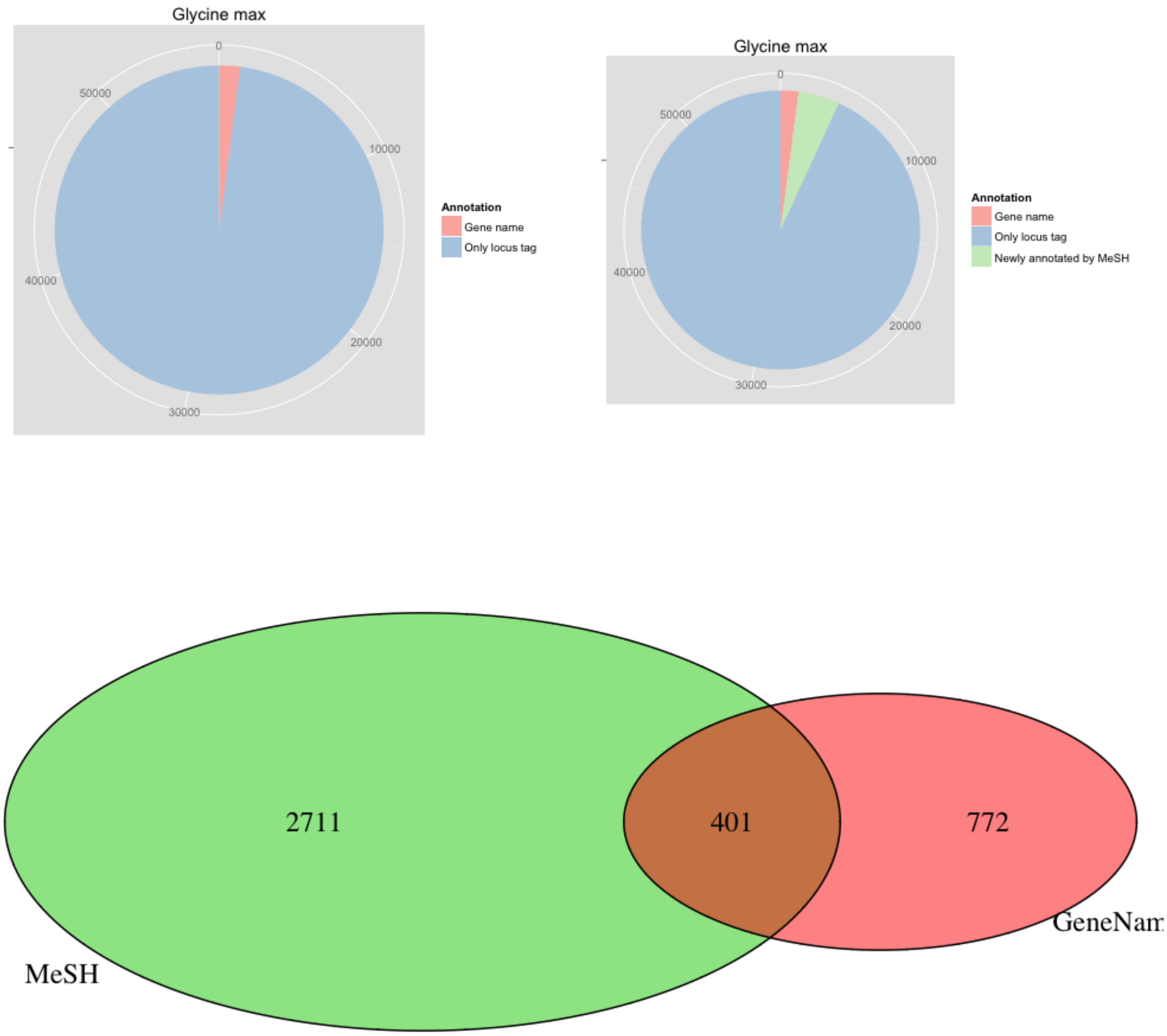

Figure 67: org.MeSH.Gma.db

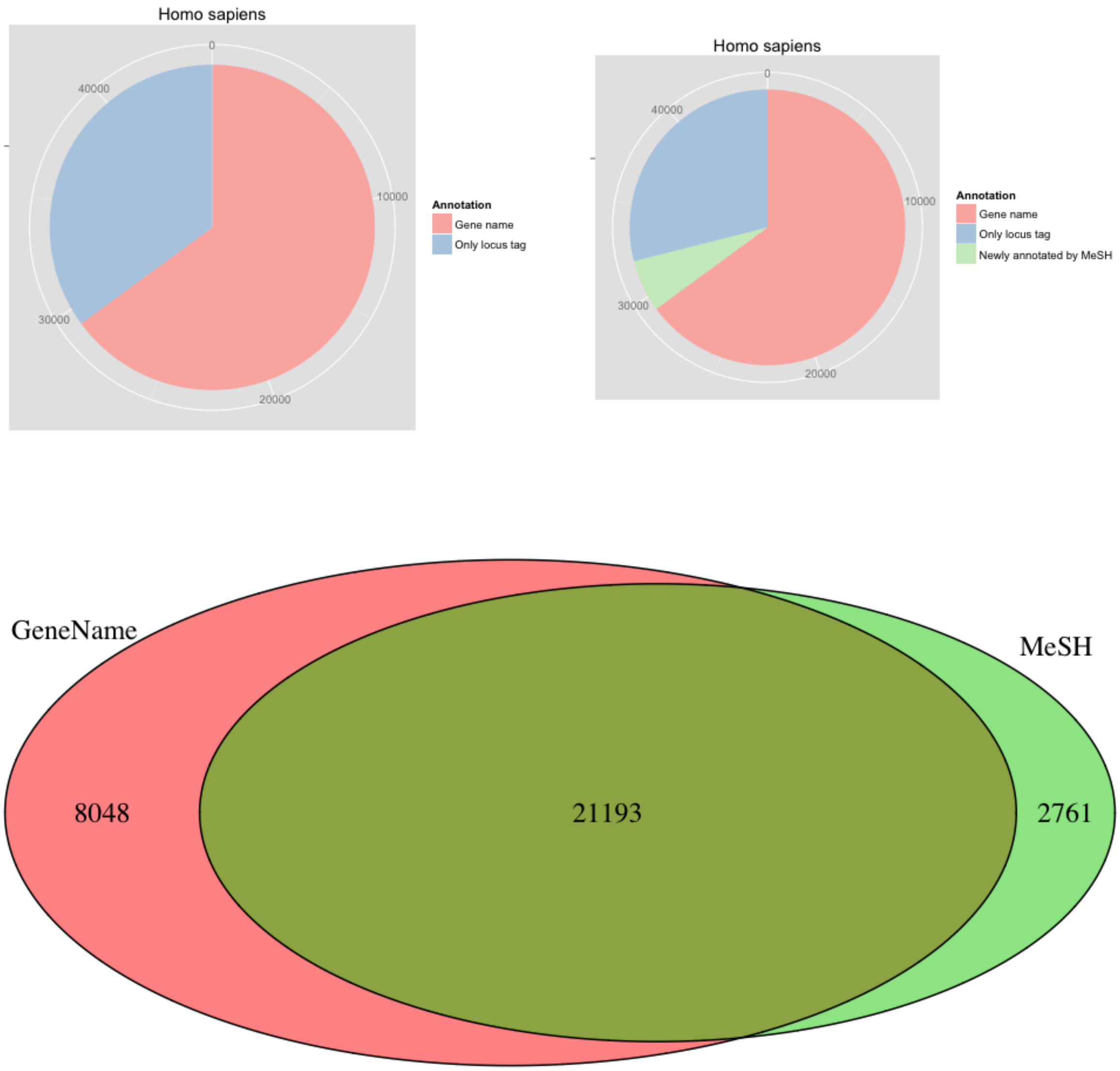

Figure 68: org.MeSH.Hsa.db

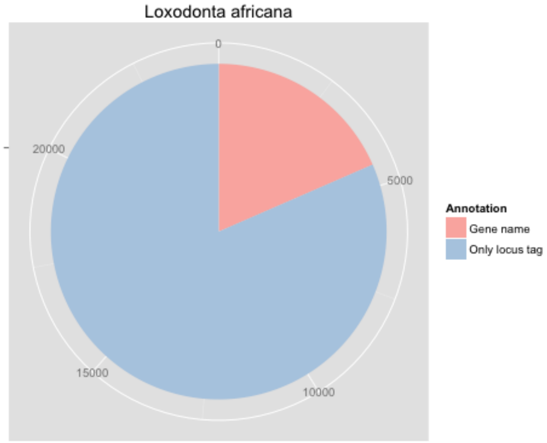

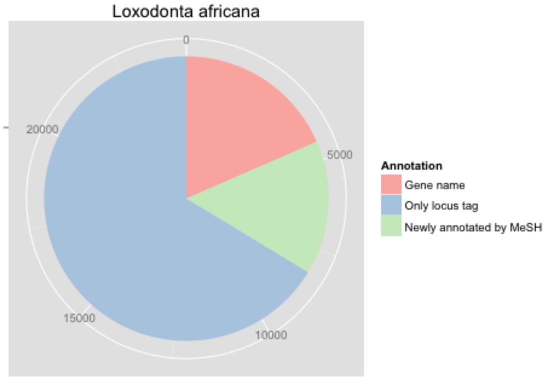

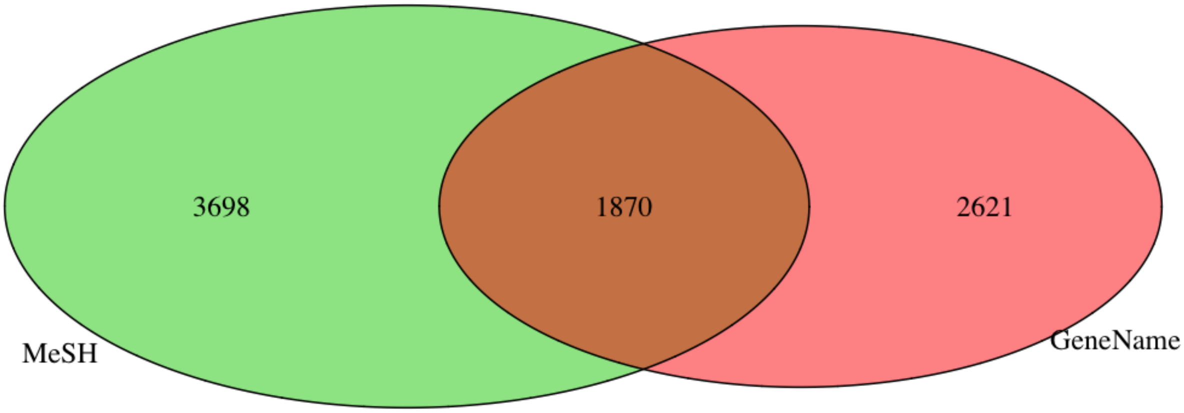

Figure 69: org.MeSH.Laf.db

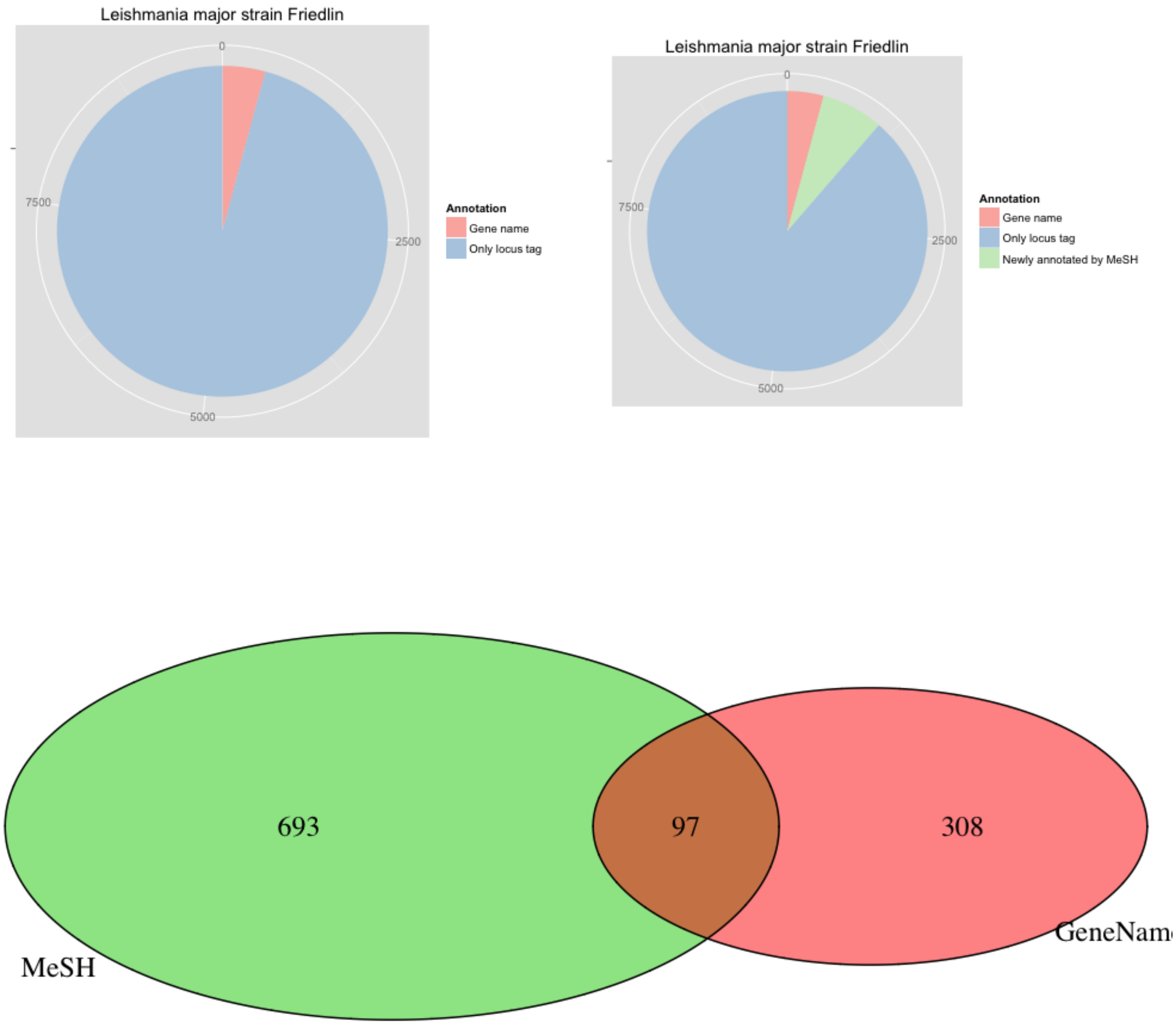

Figure 70: org.MeSH.Lma.db

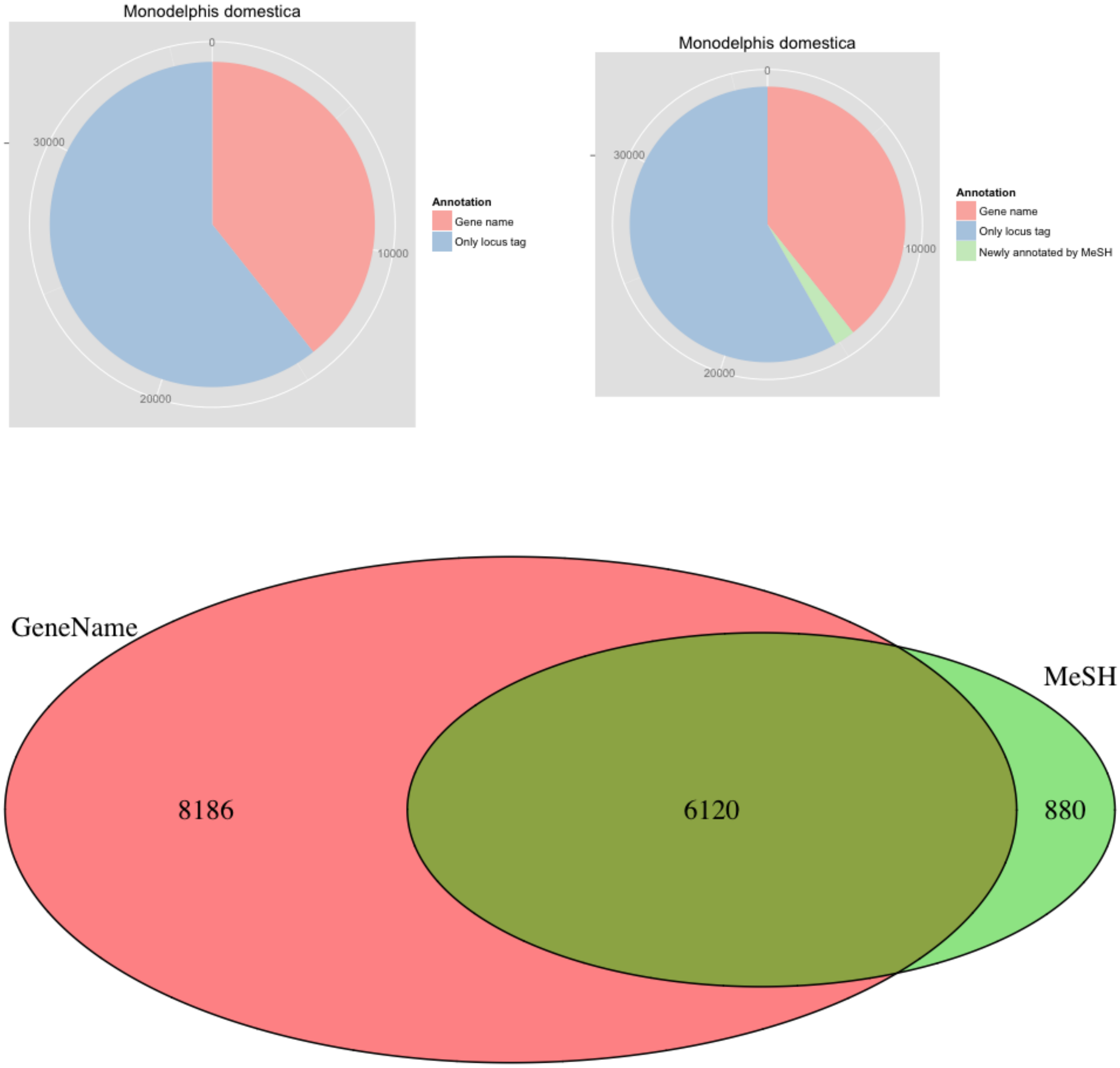

Figure 71: org.MeSH.Mdo.db

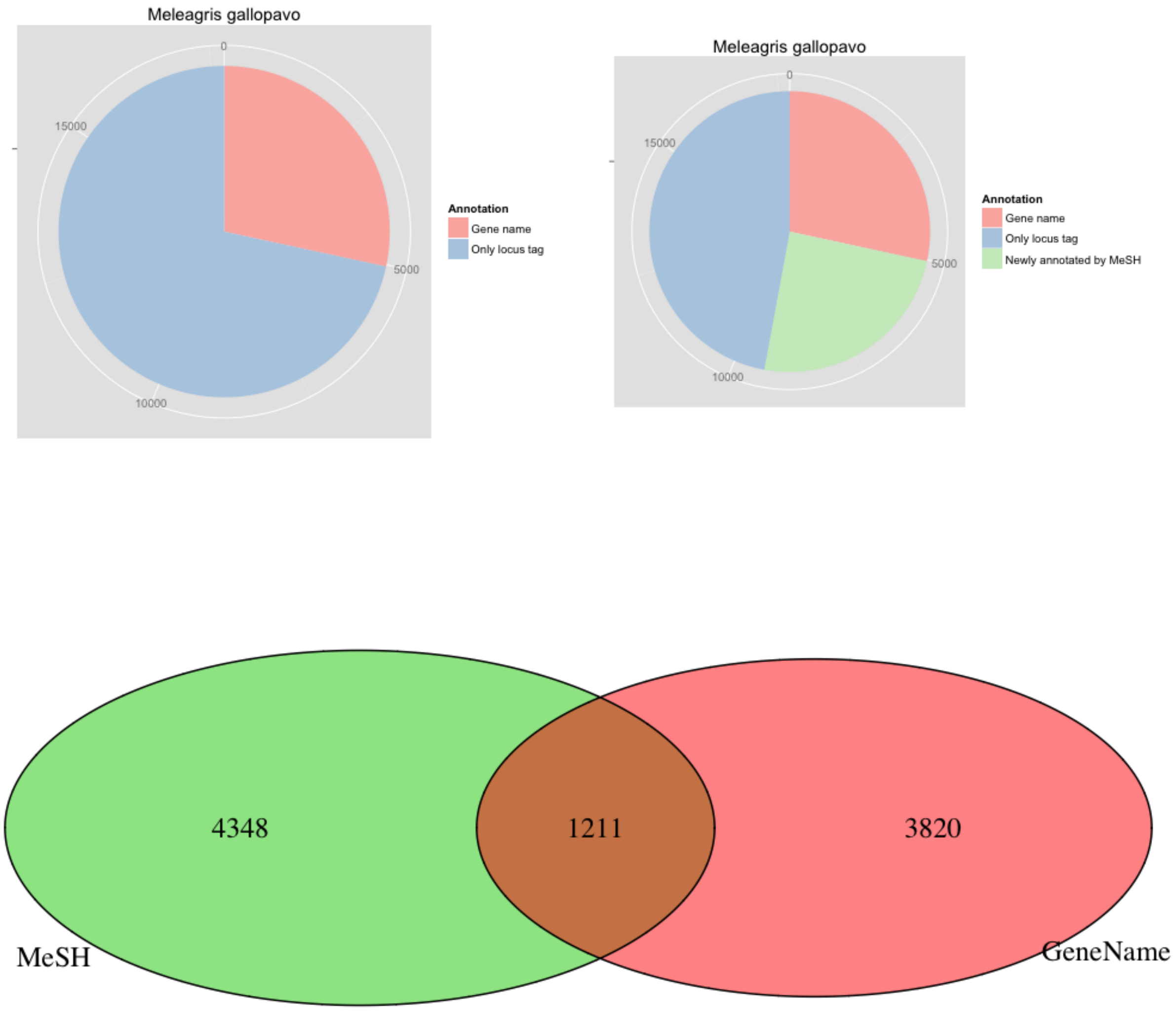

Figure 72: org.MeSH.Mga.db

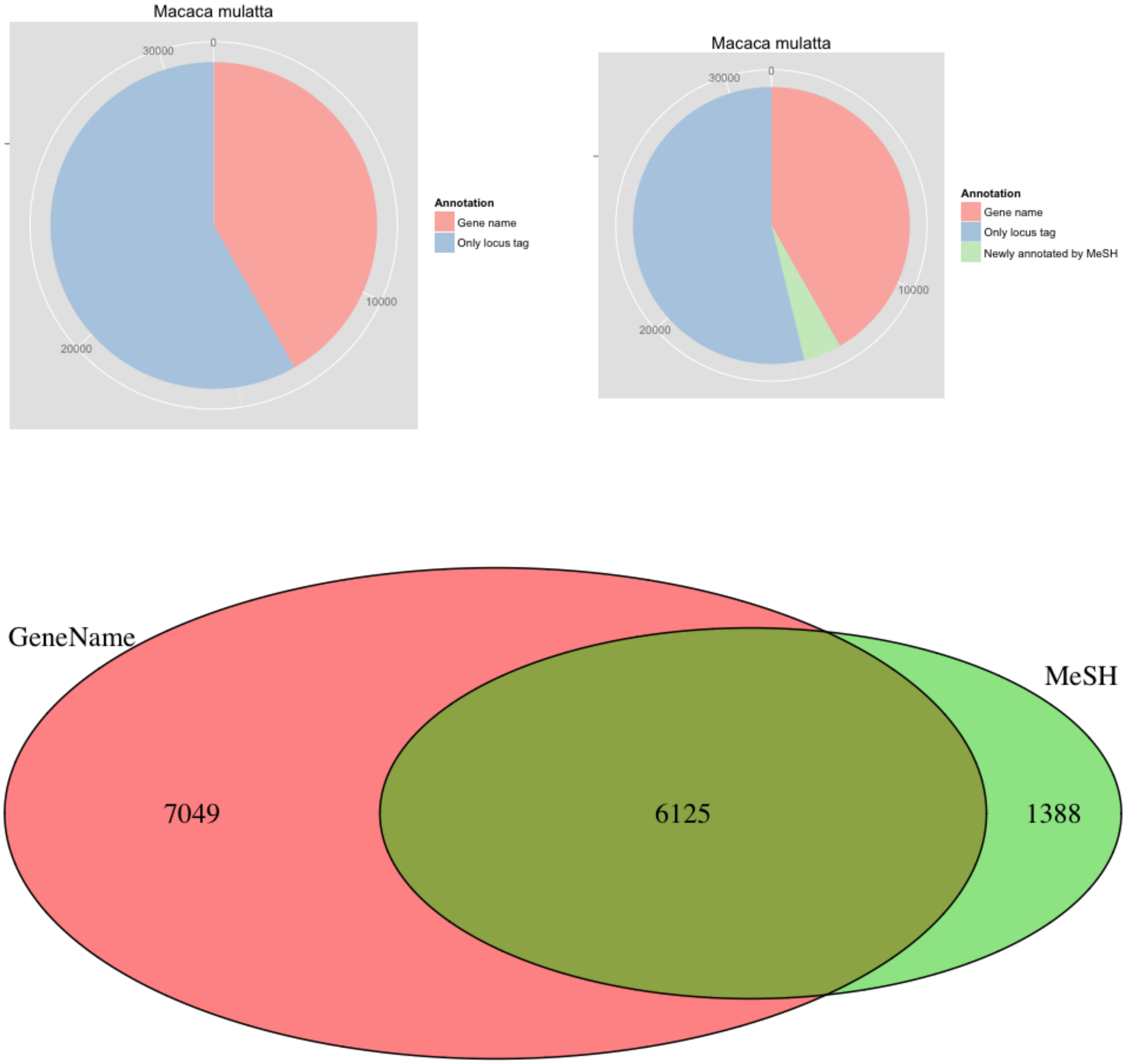

Figure 73: org.MeSH.Mml.db

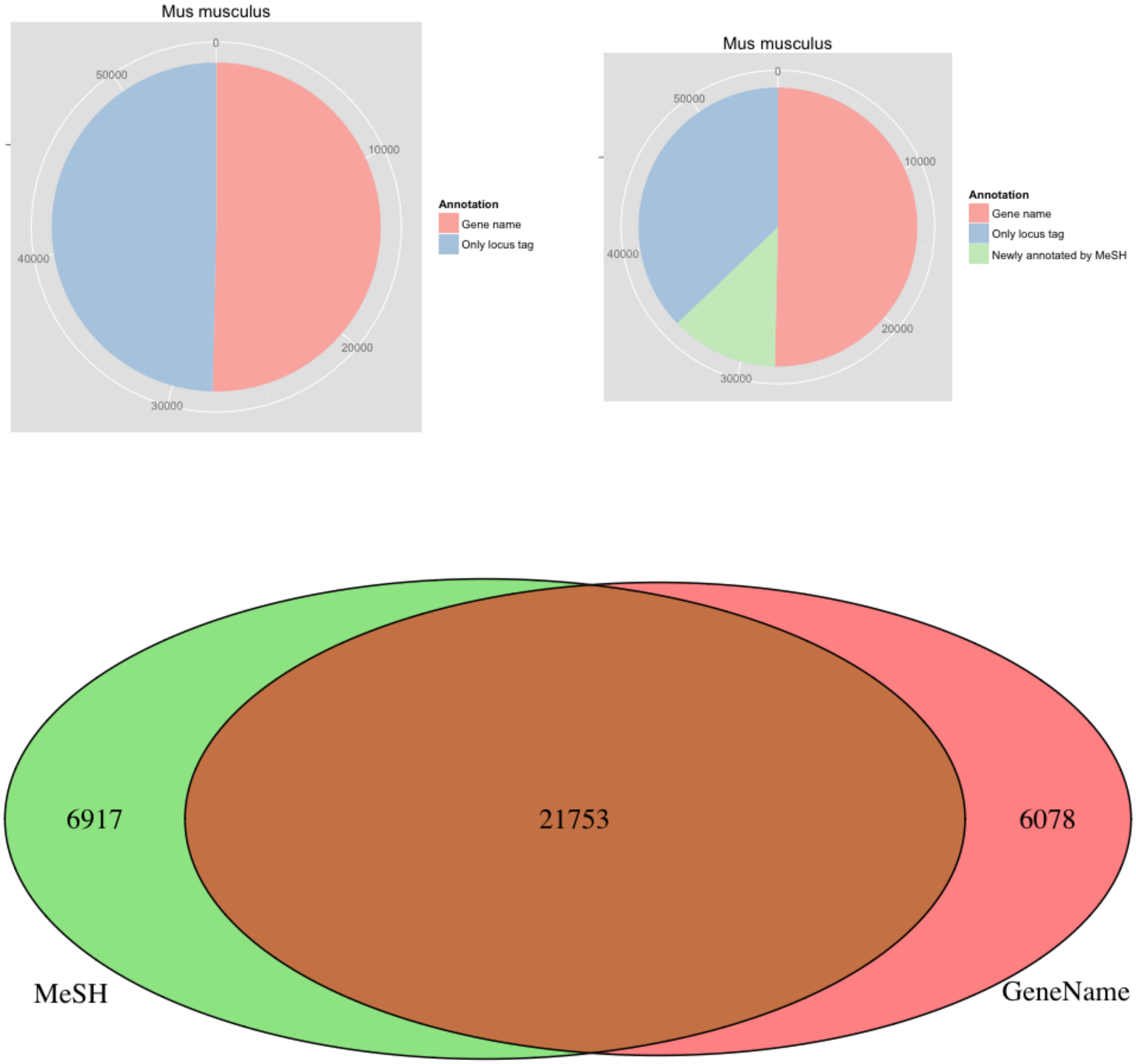

Figure 74: org.MeSH.Mmu.db

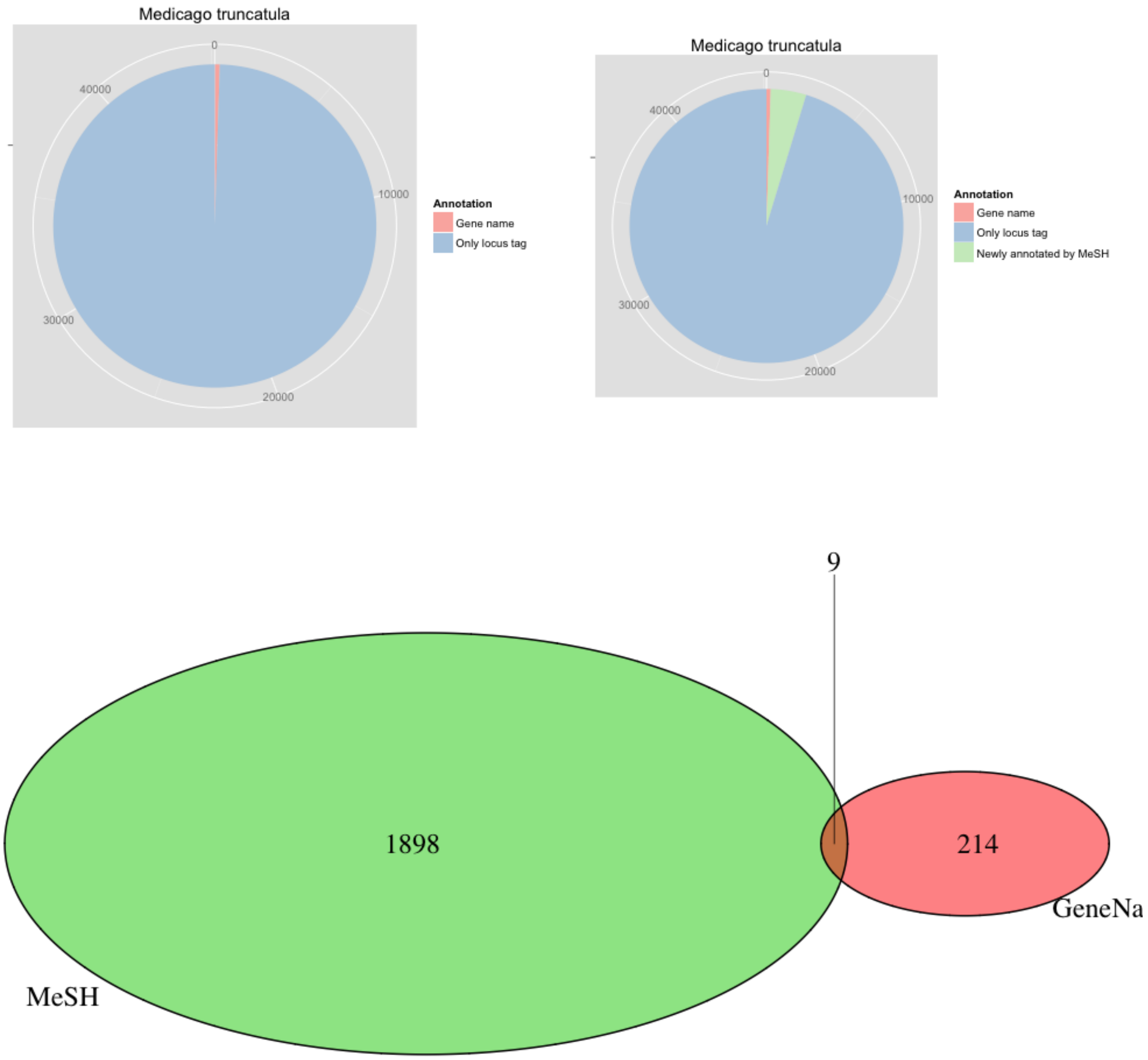

Figure 75: org.MeSH.Mtr.db

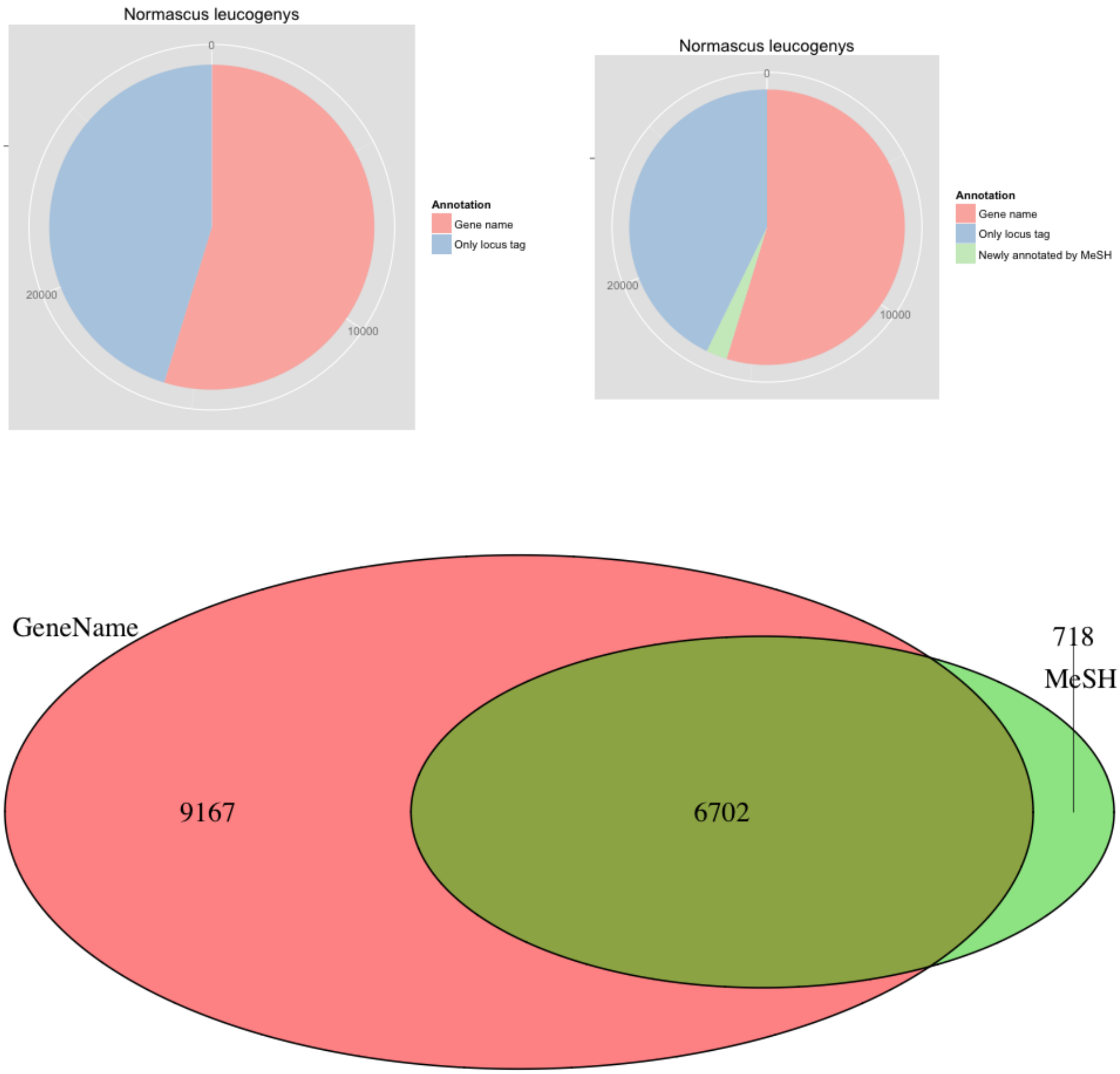

Figure 76: org.MeSH.Nle.db

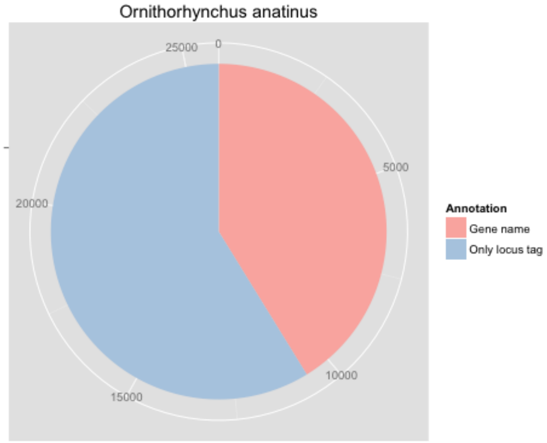

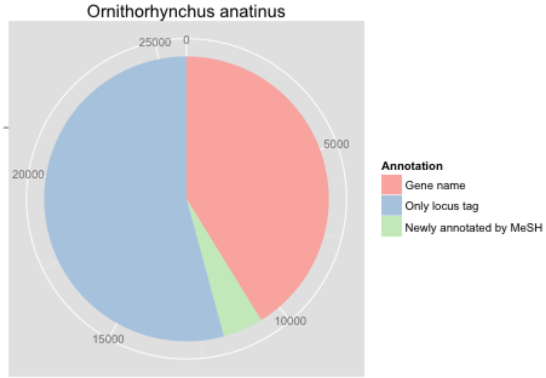

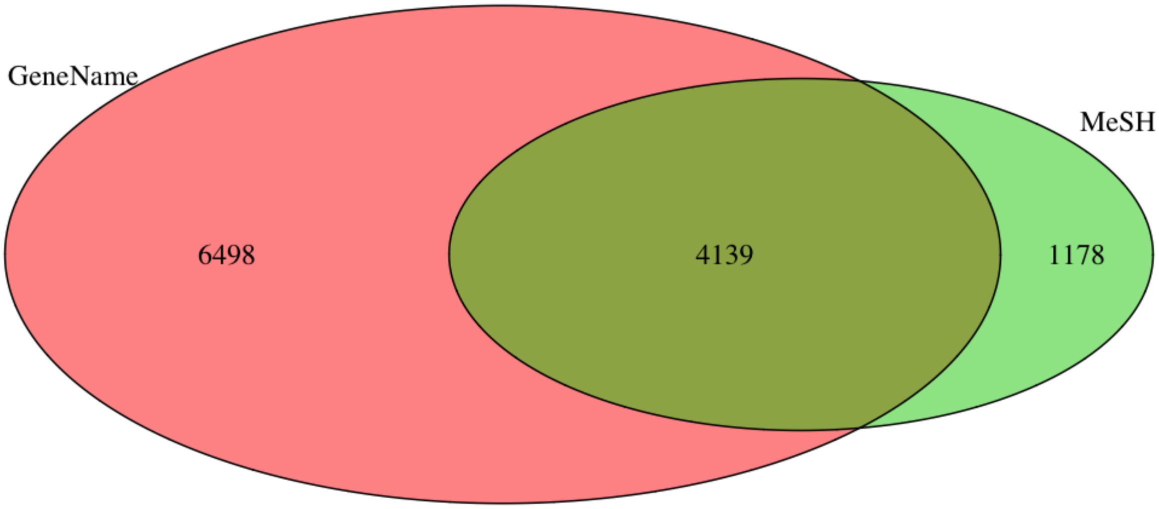

Figure 77: org.MeSH.Oan.db

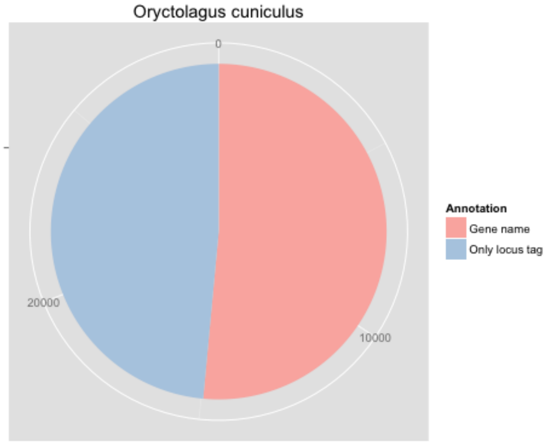

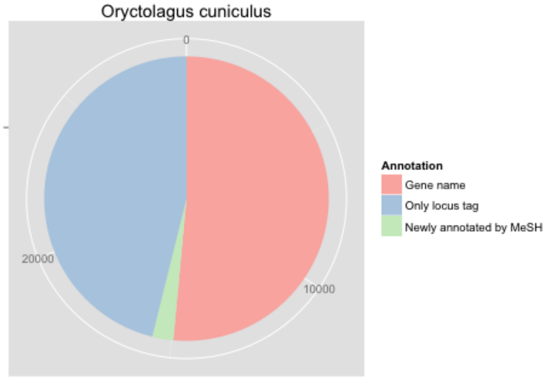

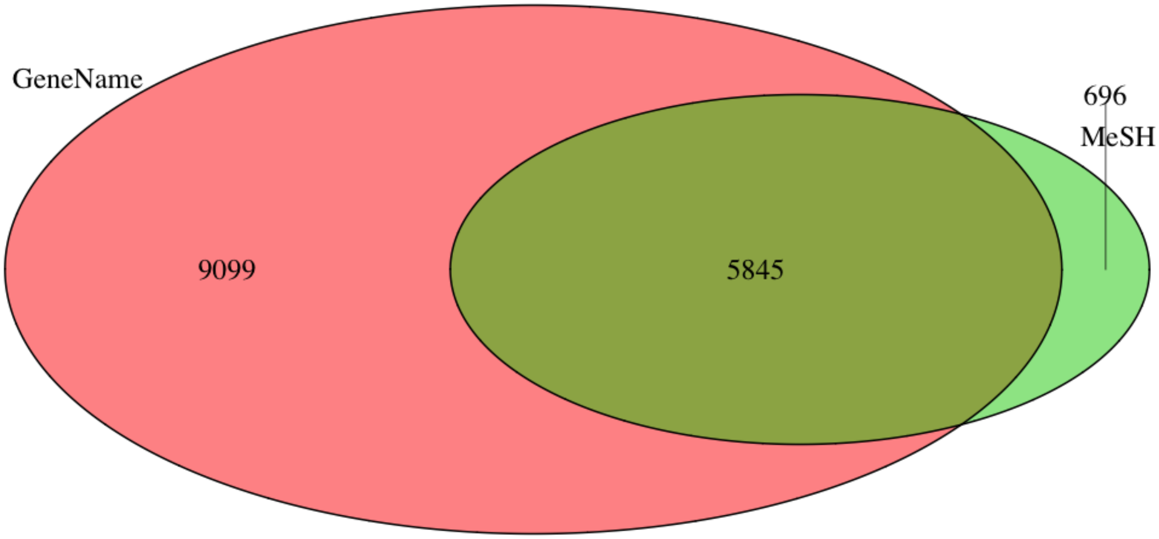

Figure 78: org.MeSH.Ocu.db

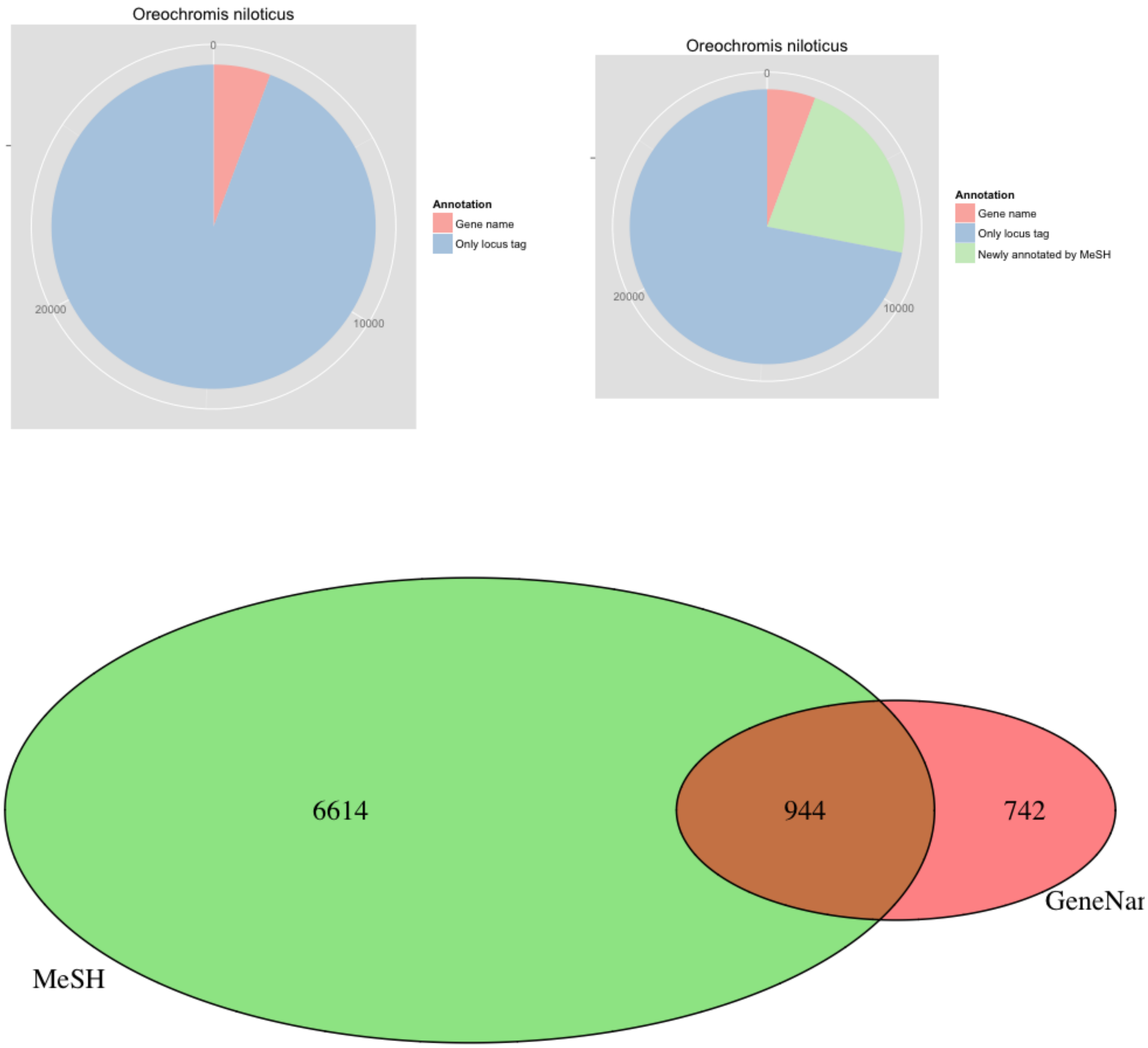

Figure 79: org.MeSH.Oni.db

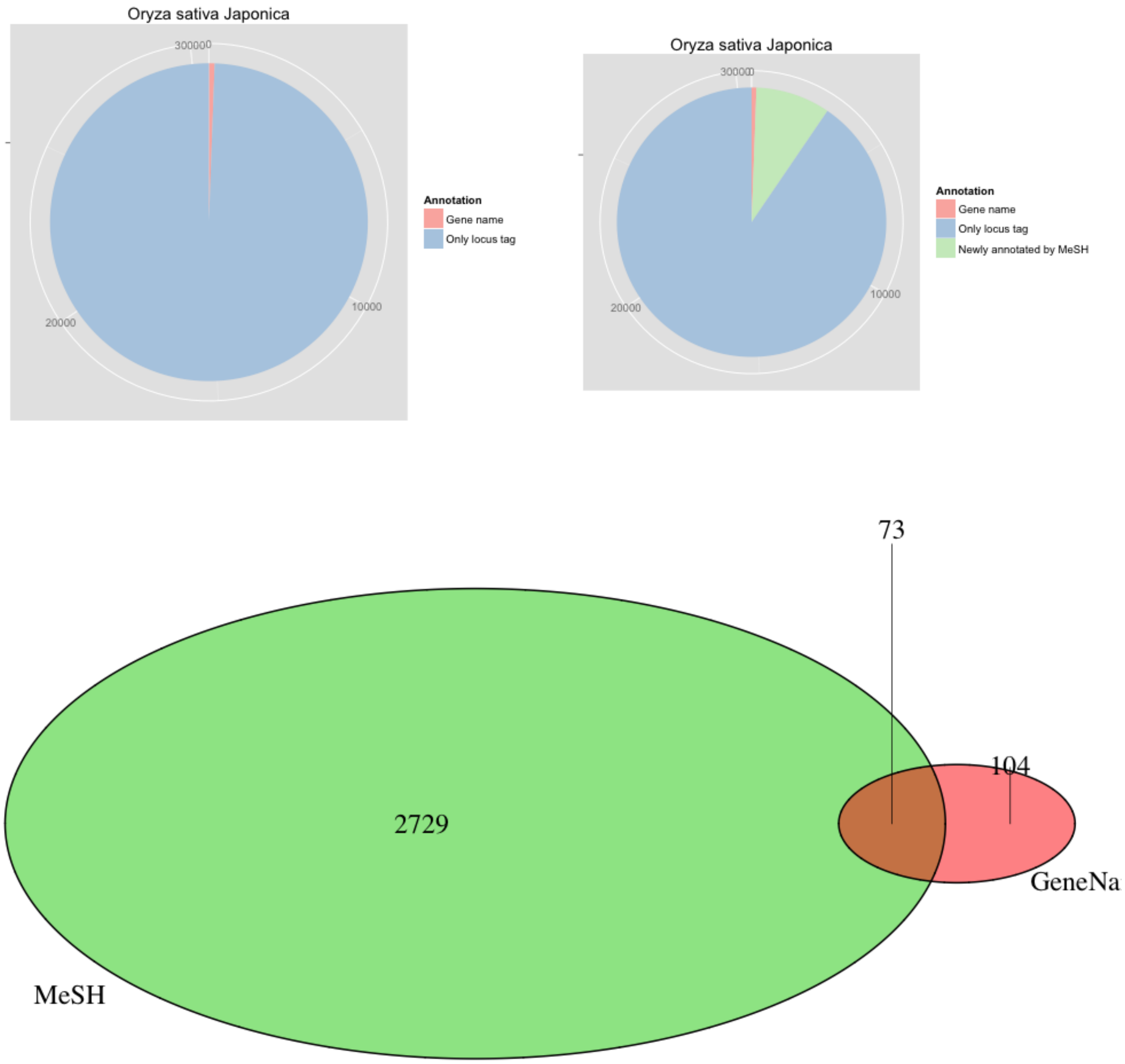

Figure 80: org.MeSH.Osa.db

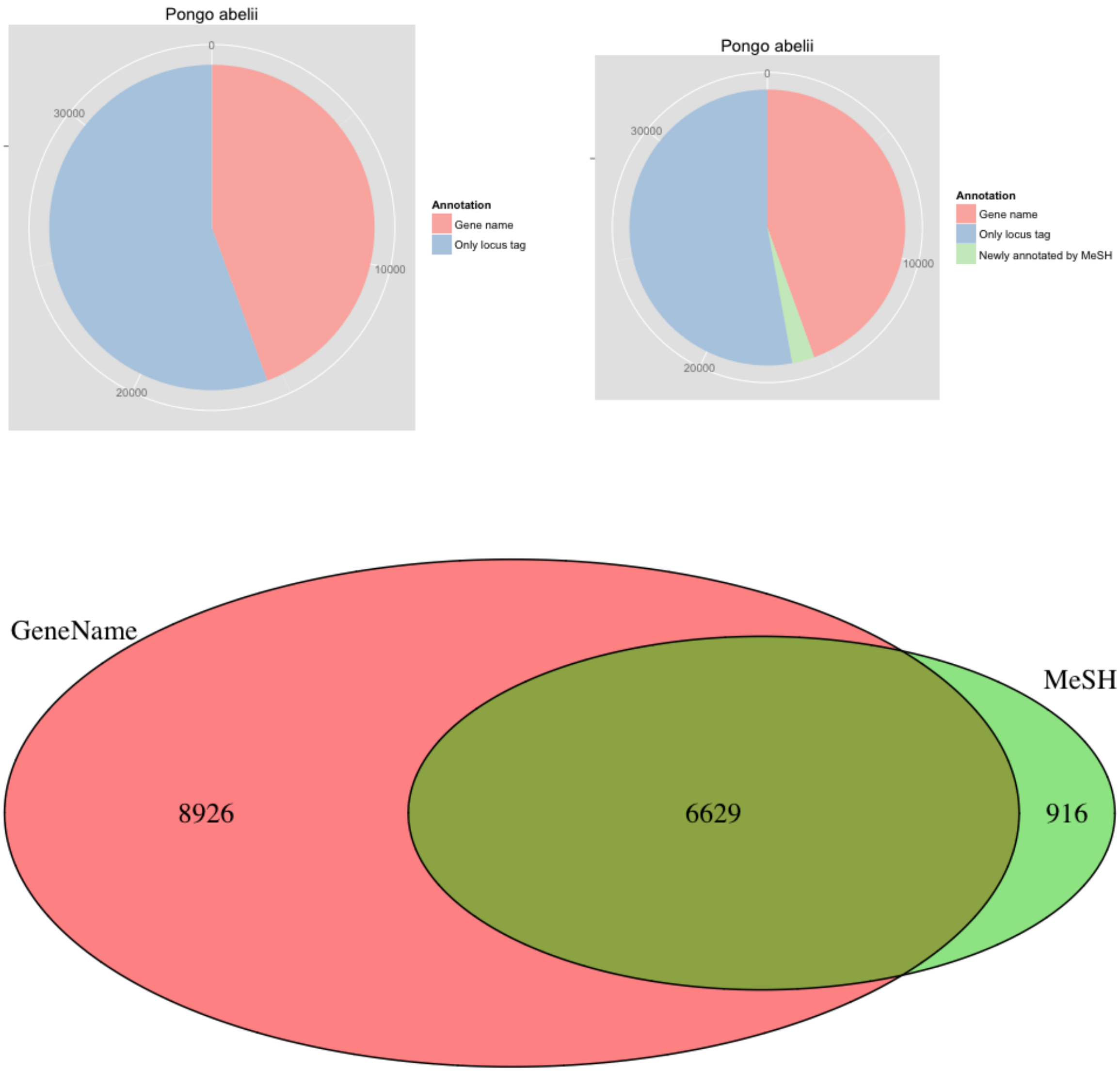

Figure 81: org.MeSH.Pab.db

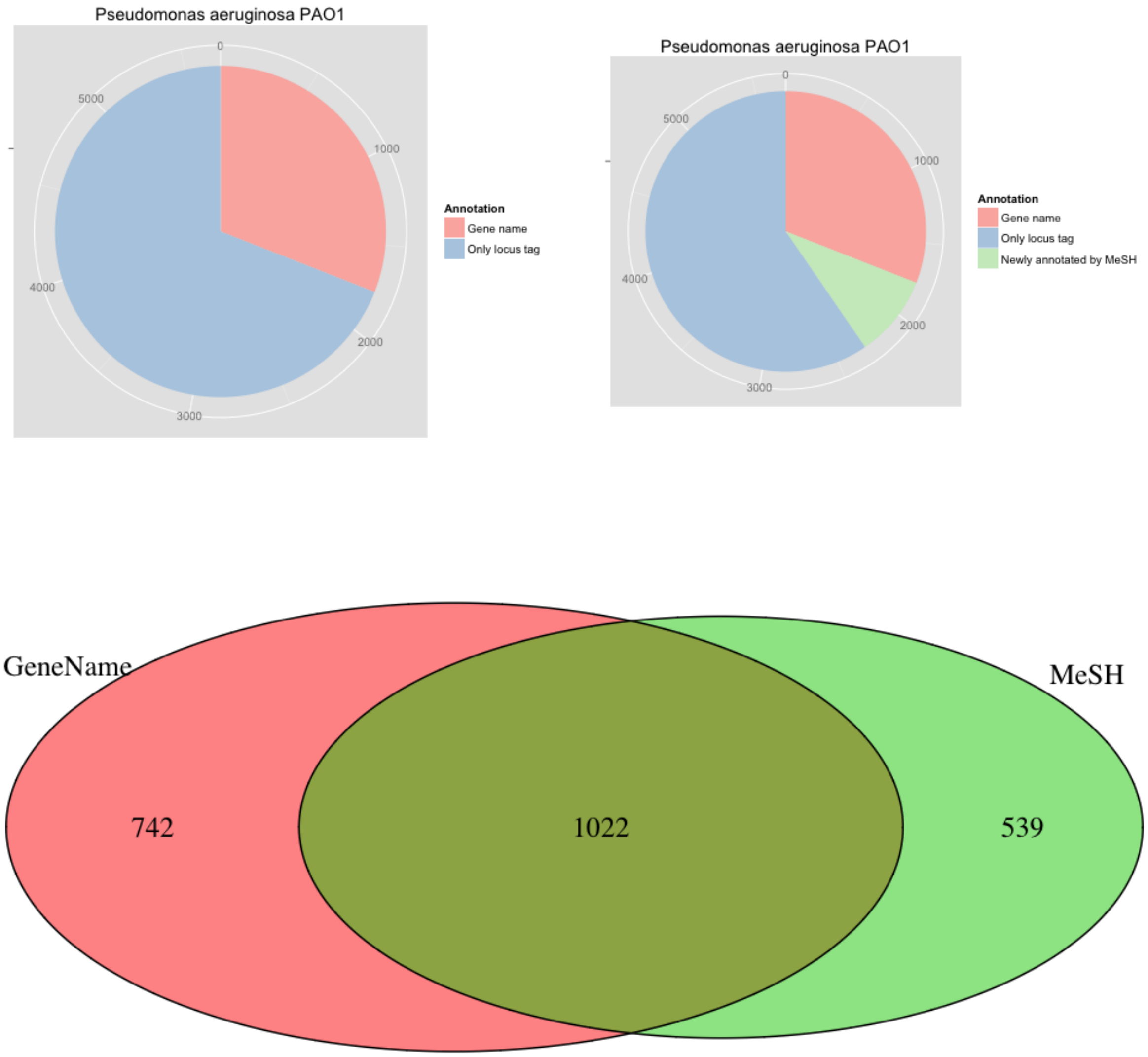

Figure 82: org.MeSH.Pae.PAO1.db

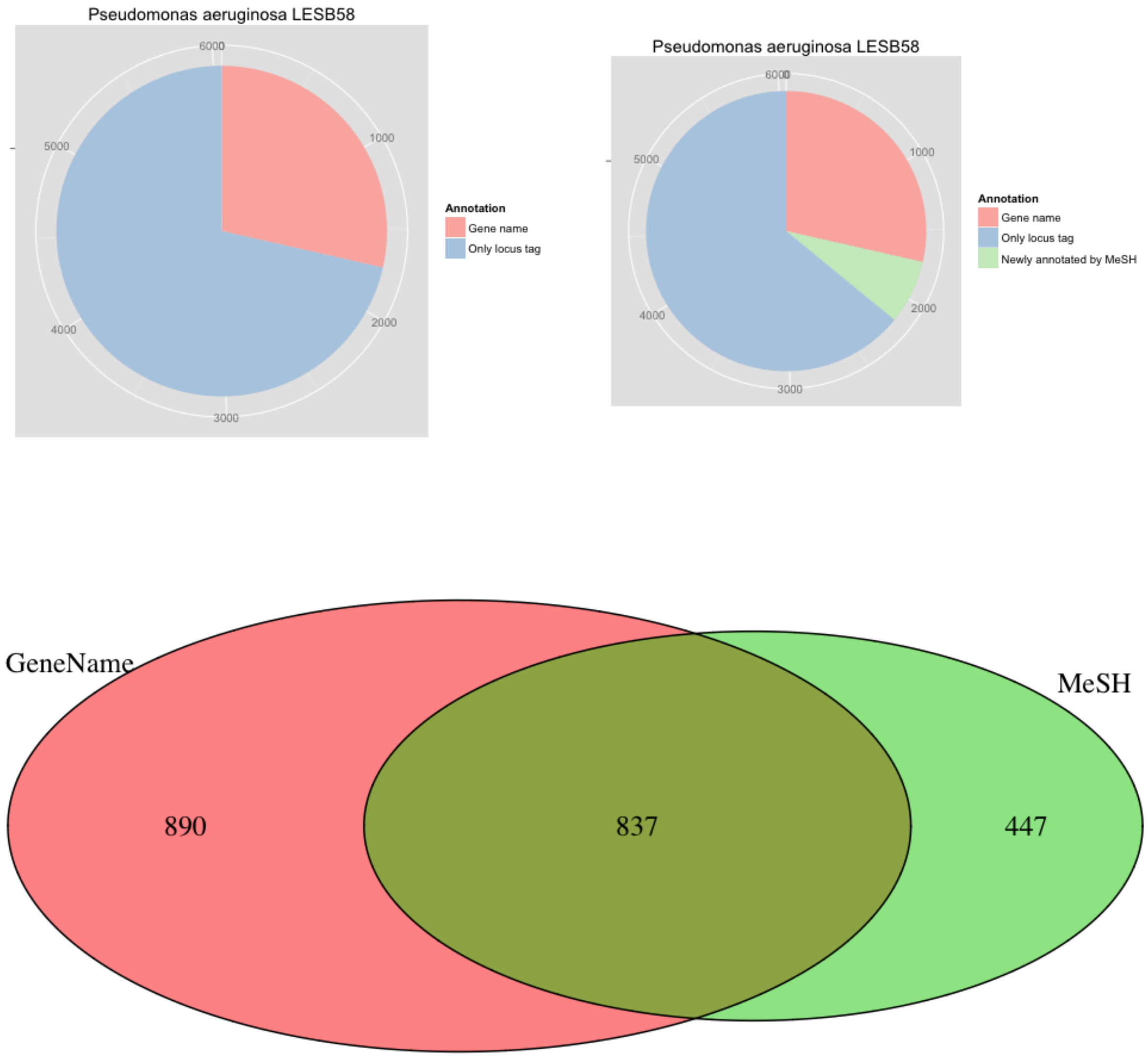

Figure 83: org.MeSH.Pae.LESB58.db

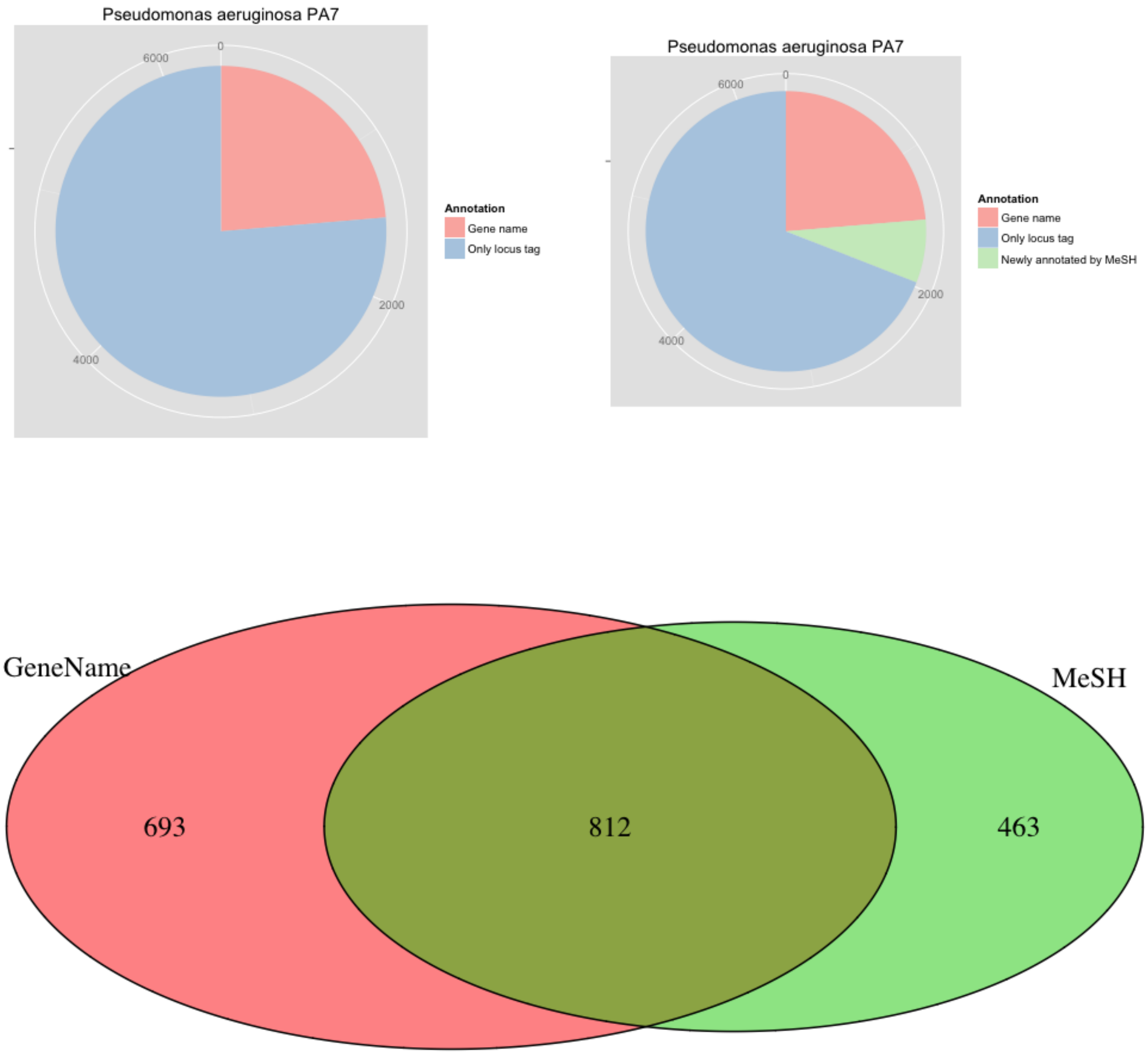

Figure 84: org.MeSH.Pae.PA7.db

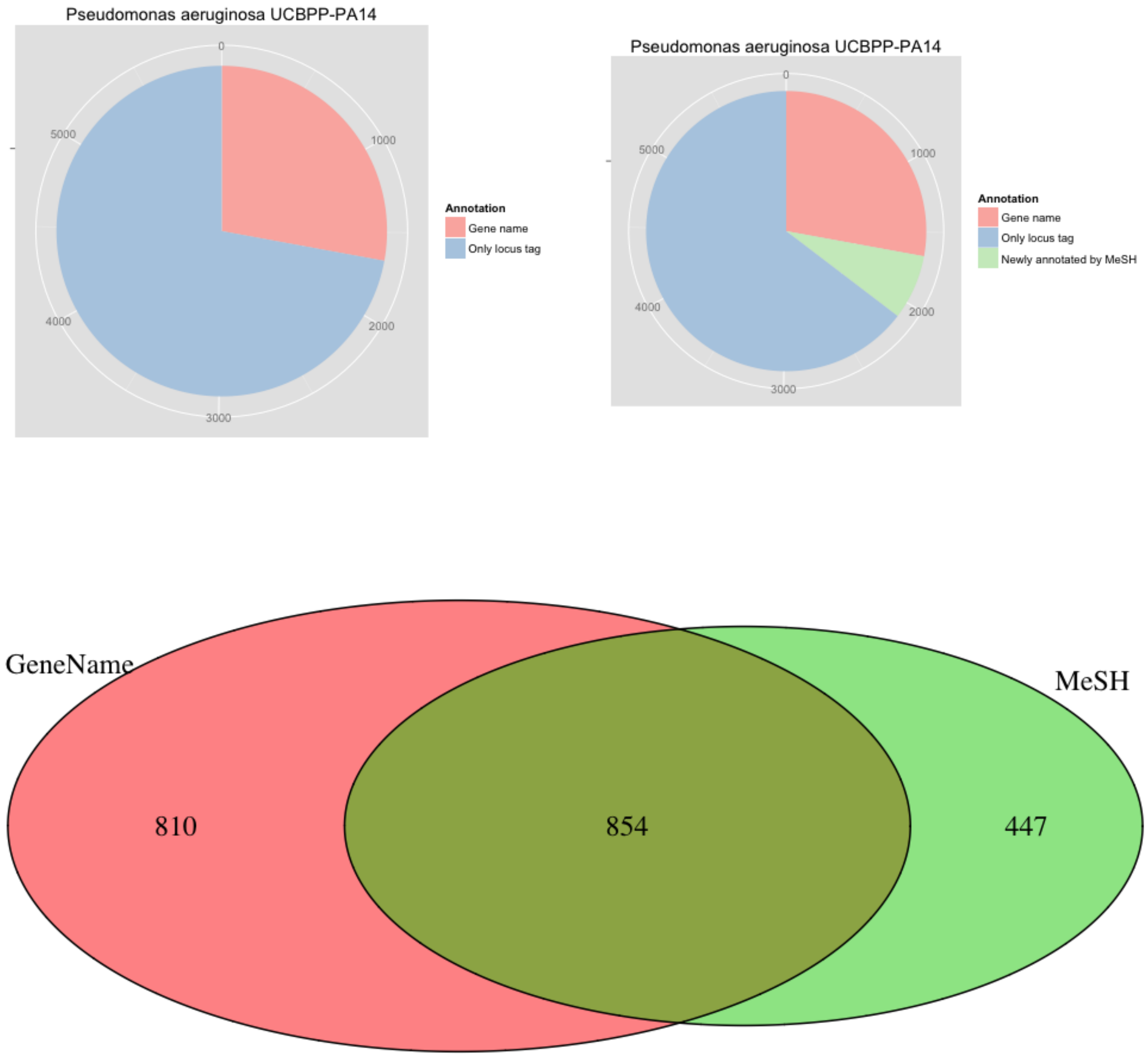

Figure 85: org.MeSH.Pae.PA14.db

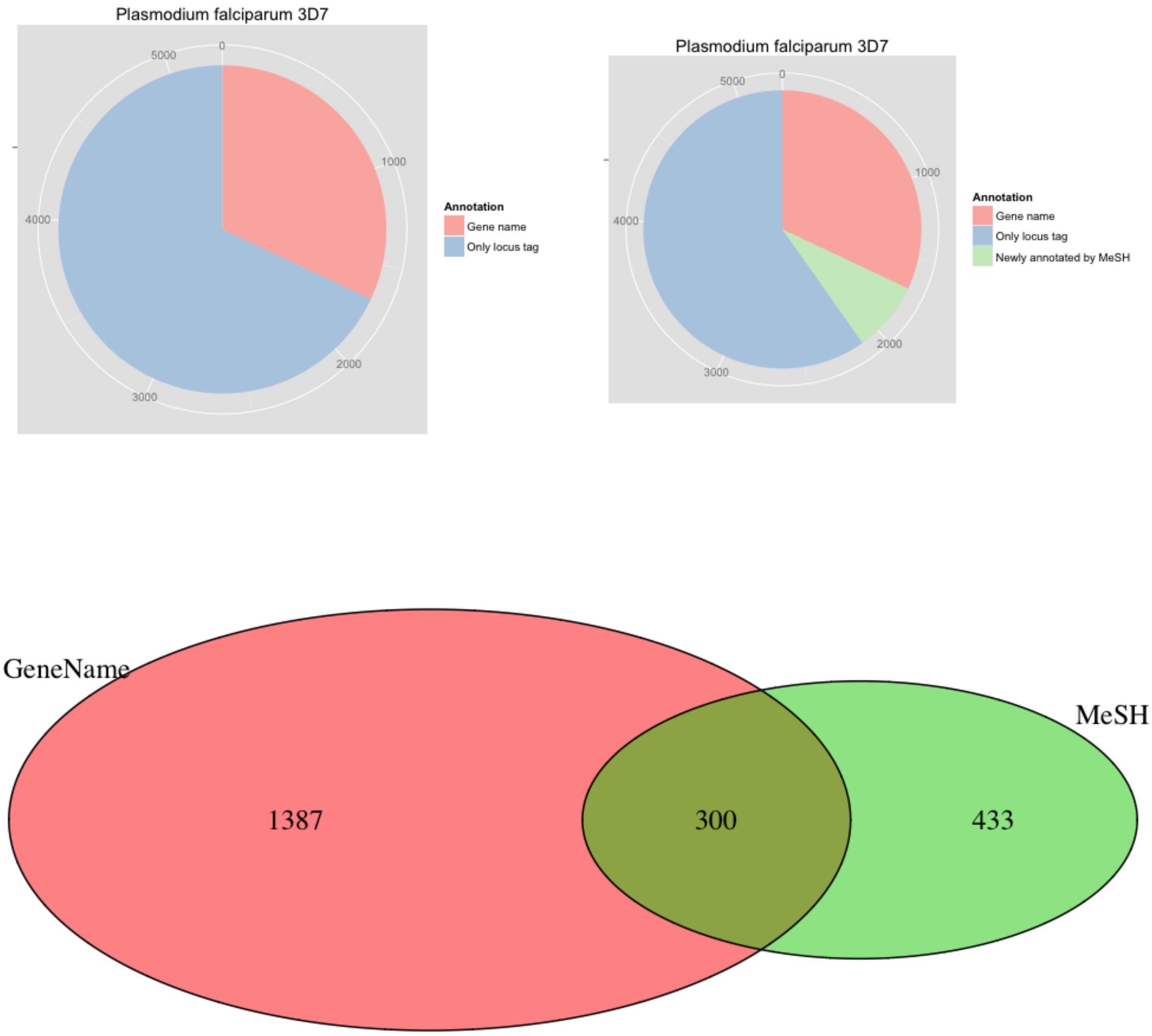

Figure 86: org.MeSH.Pfa.3D7.db

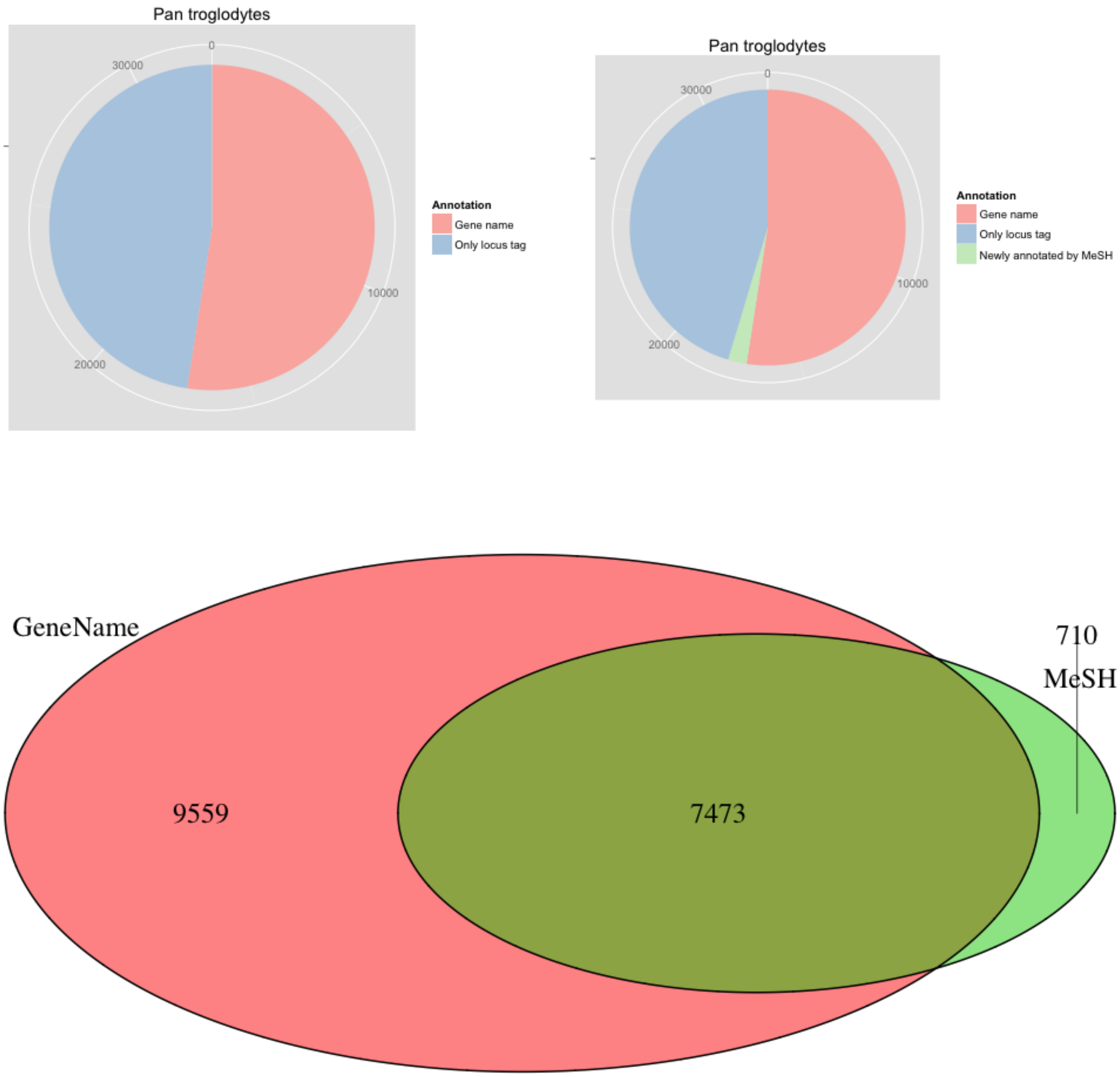

Figure 87: org.MeSH.Pto.db

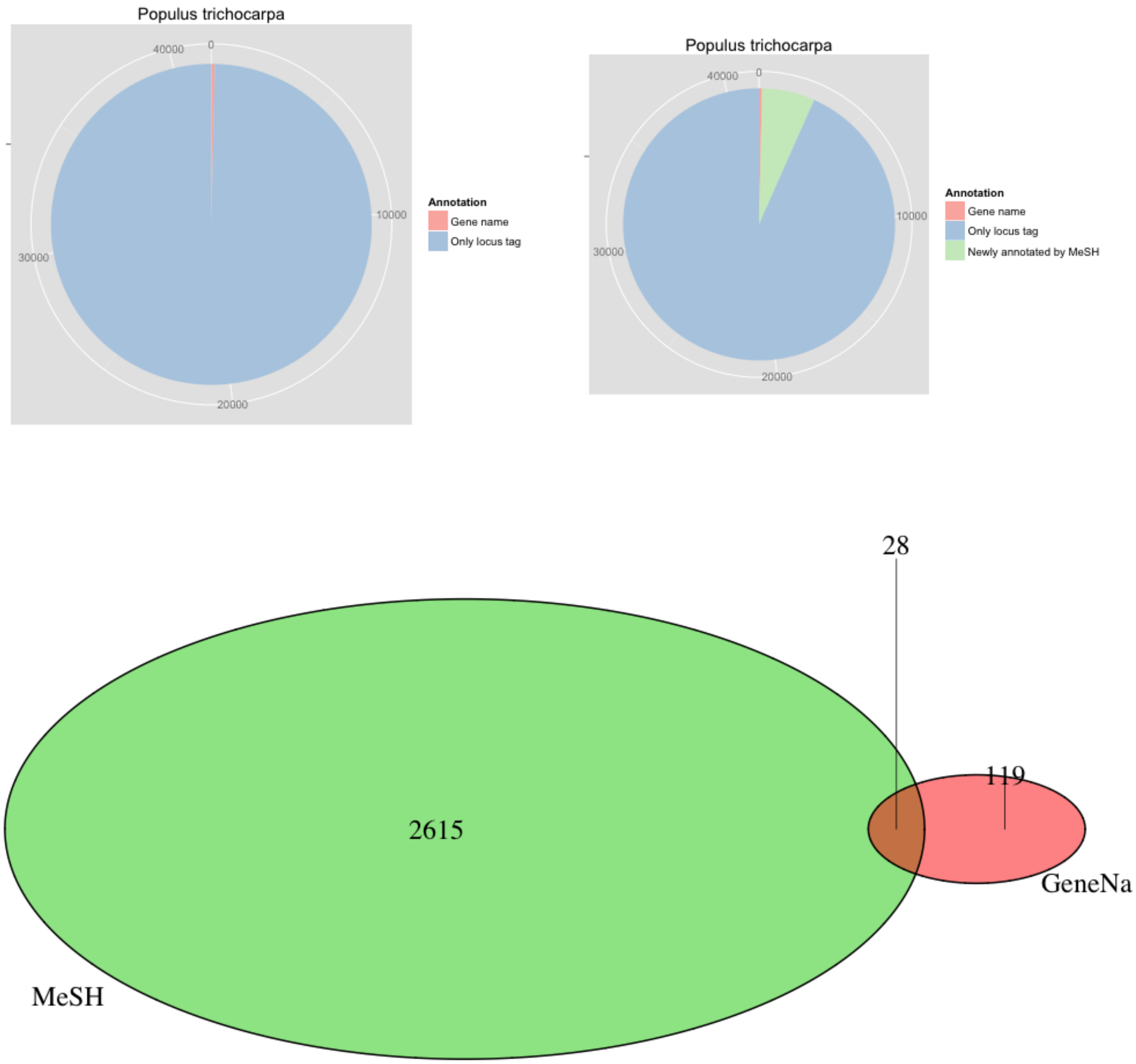

Figure 88: org.MeSH.Ptr.db

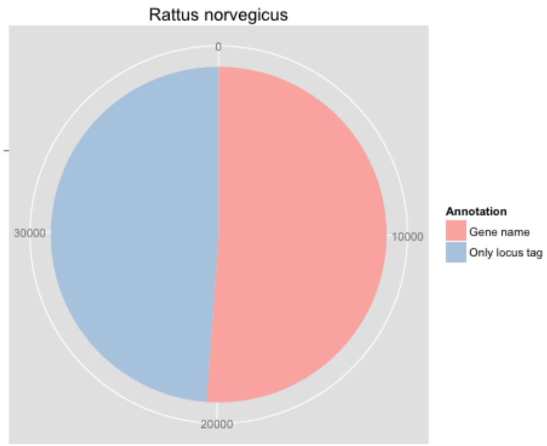

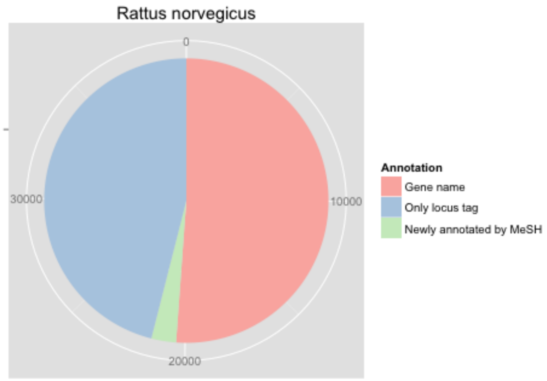

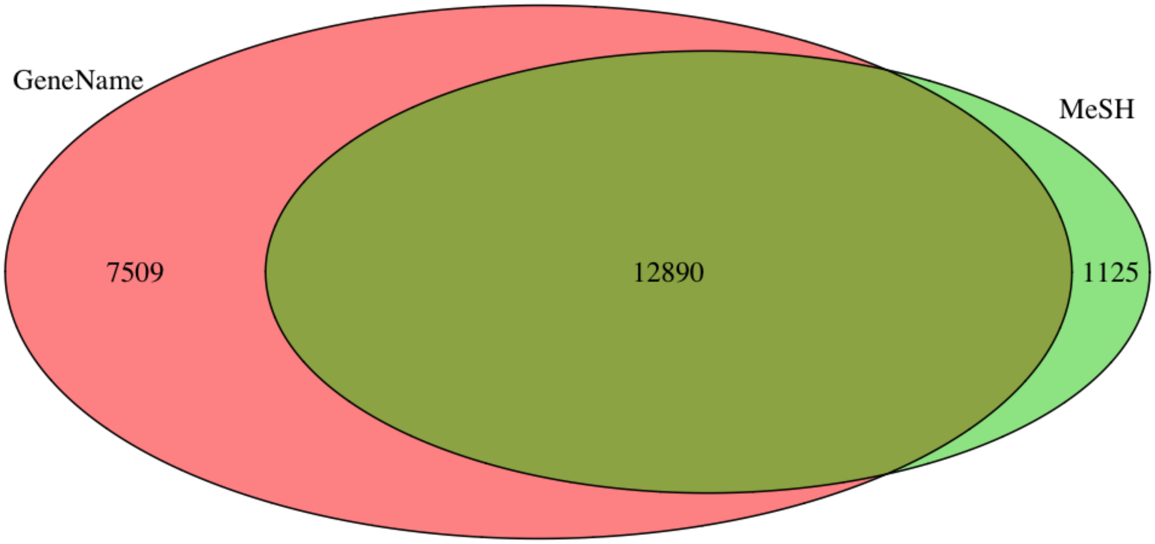

Figure 89: org.MeSH.Rno.db

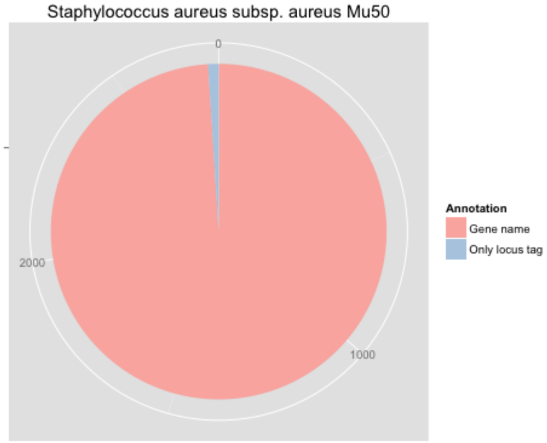

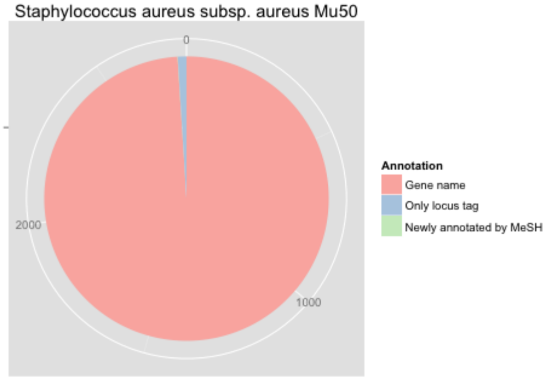

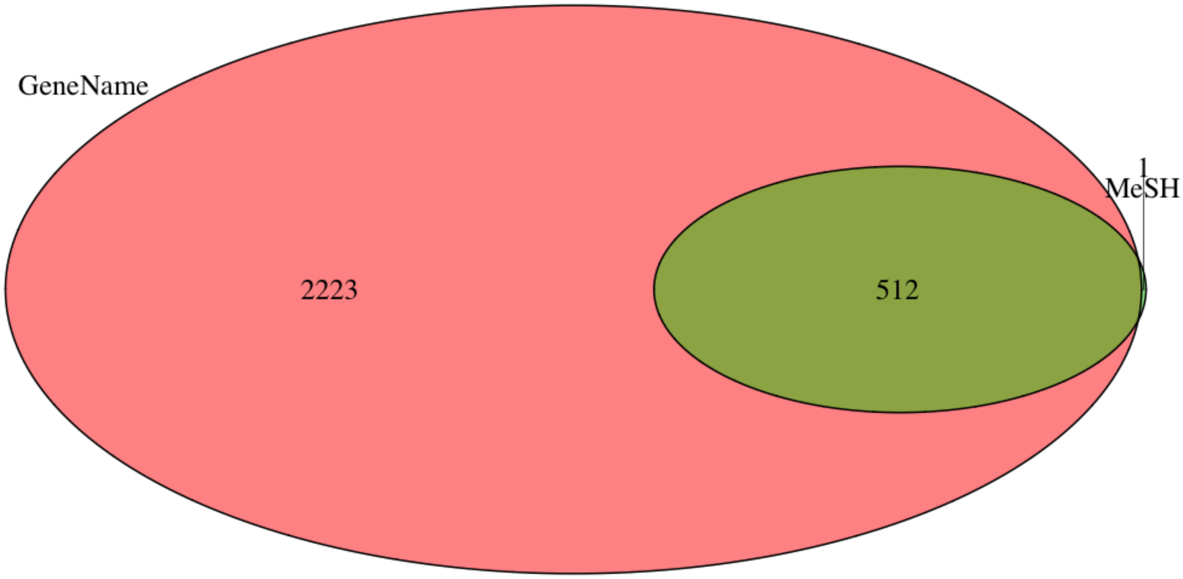

Figure 90: org.MeSH.Sau.Mu50.db

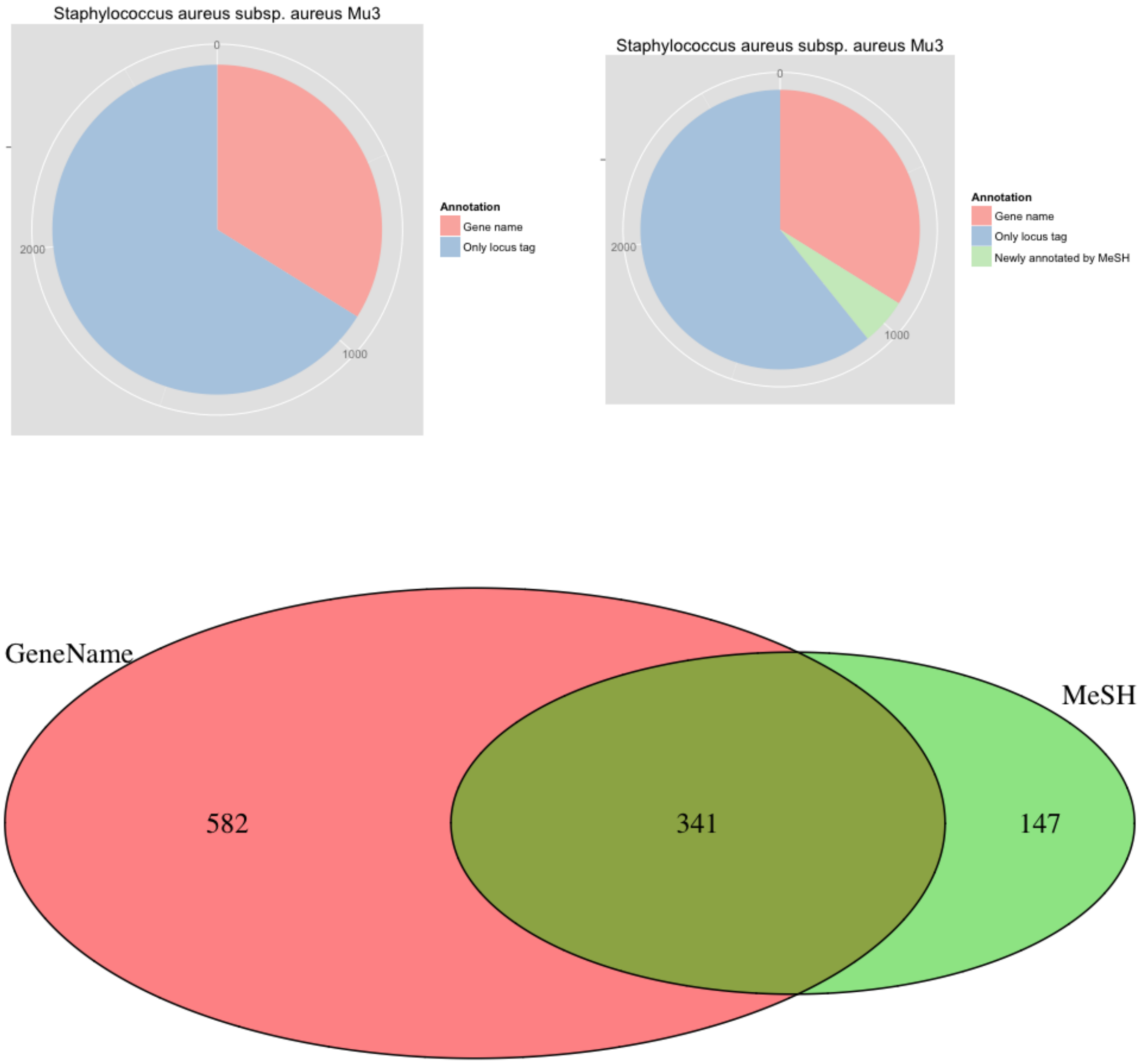

Figure 91: org.MeSH.Sau.Mu3.db

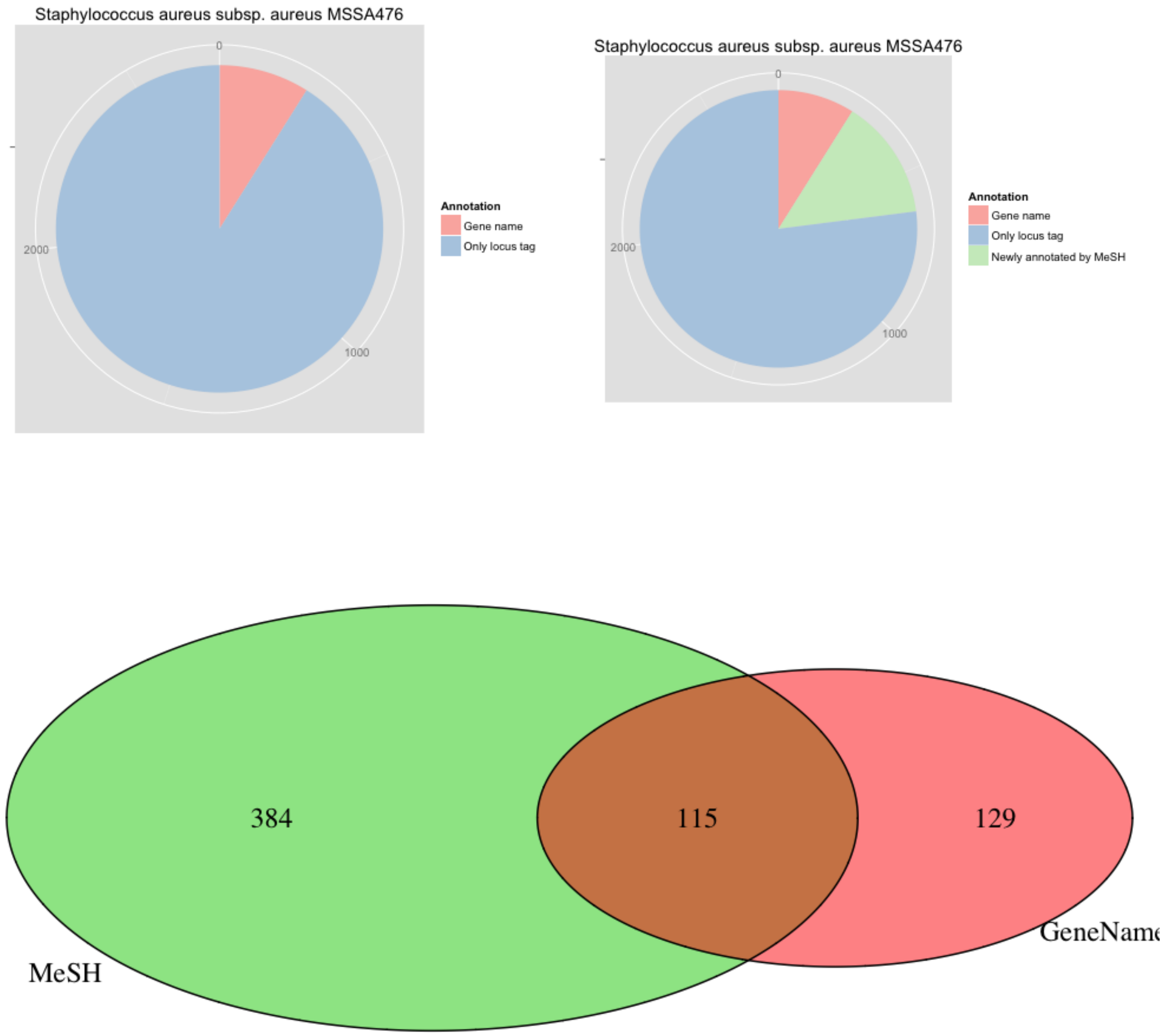

Figure 92: org.MeSH.Sau.MSSA476.db

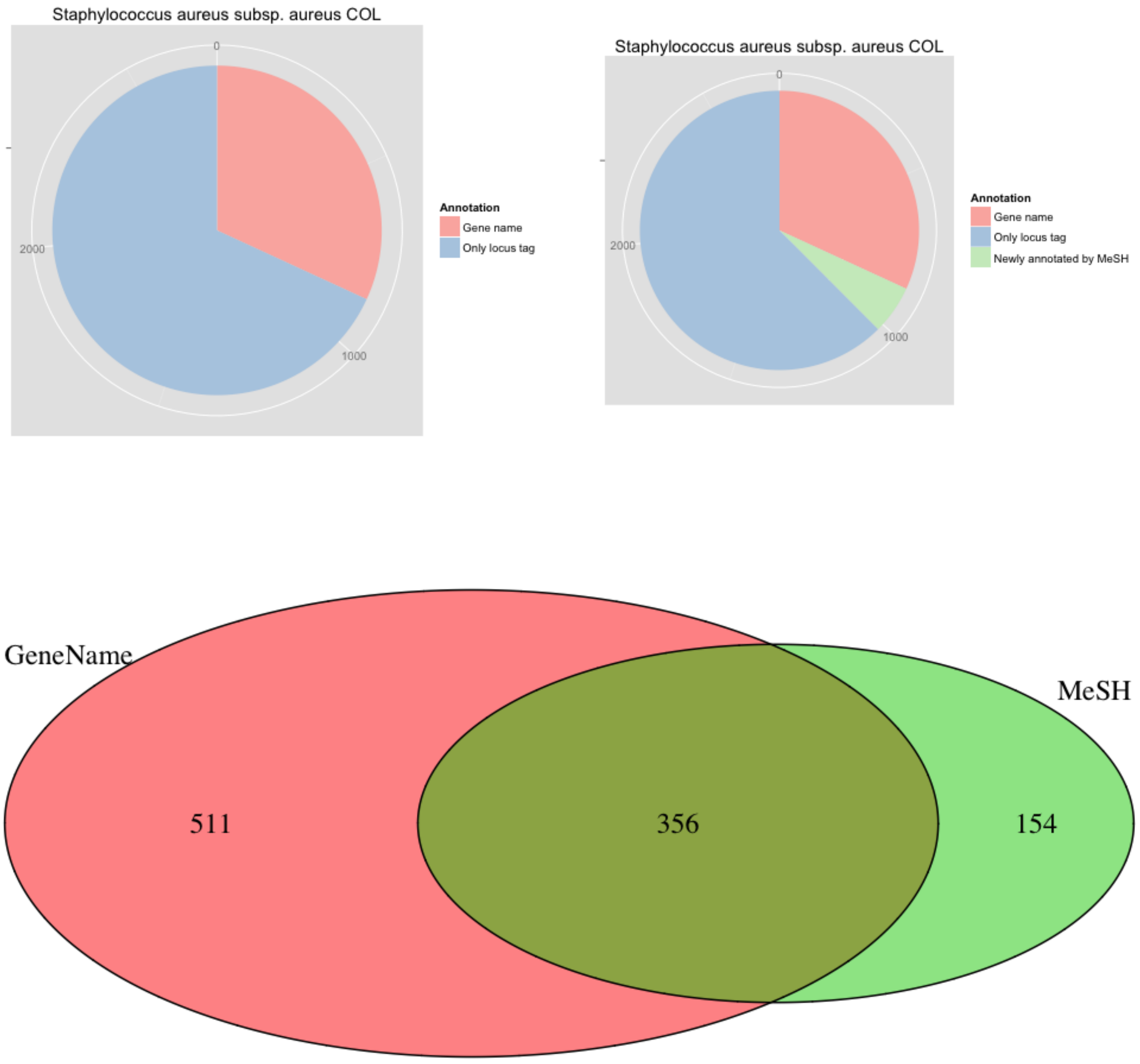

Figure 93: org.MeSH.Sau.COL.db

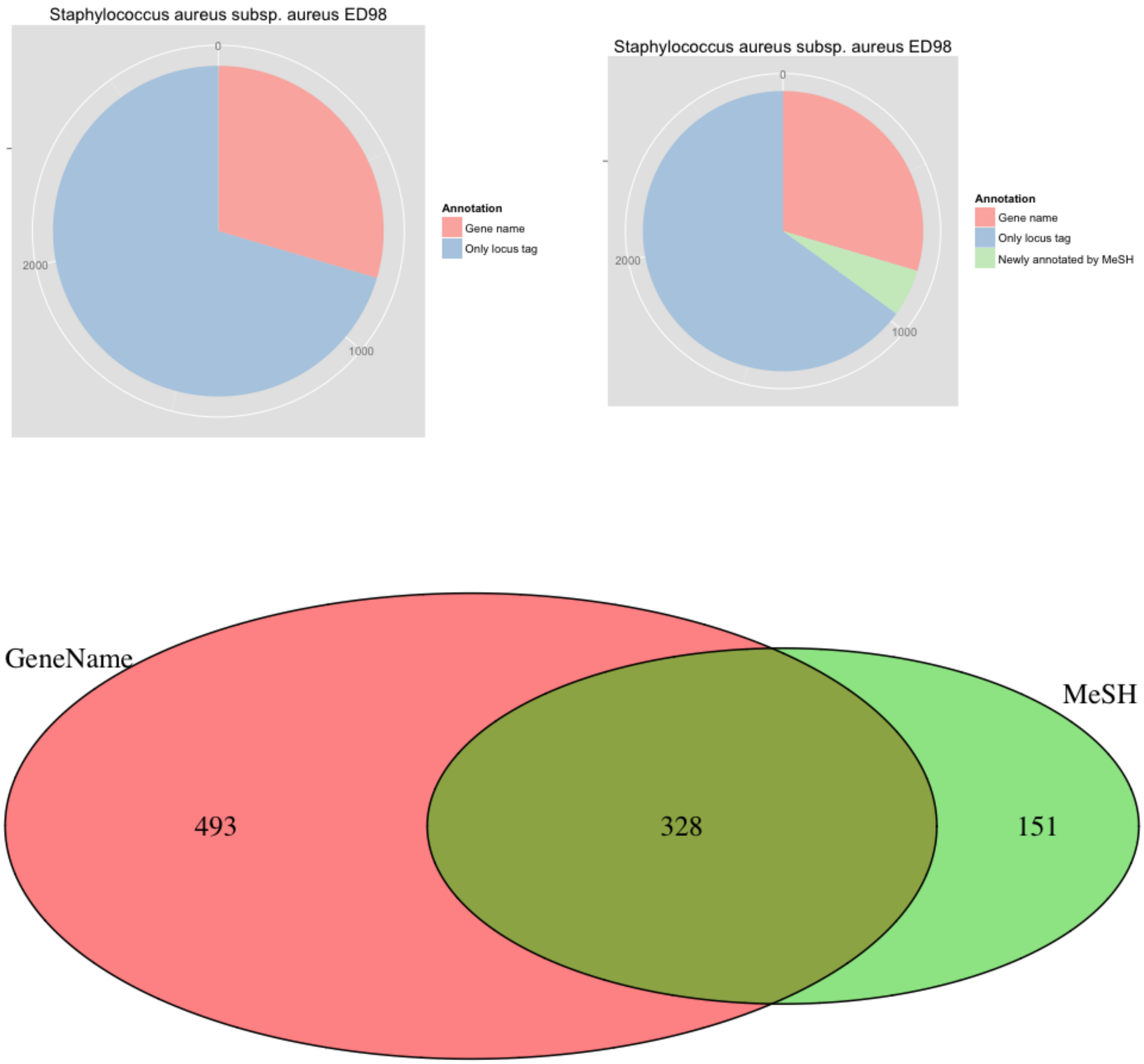

Figure 94: org.MeSH.Sau.ED98.db

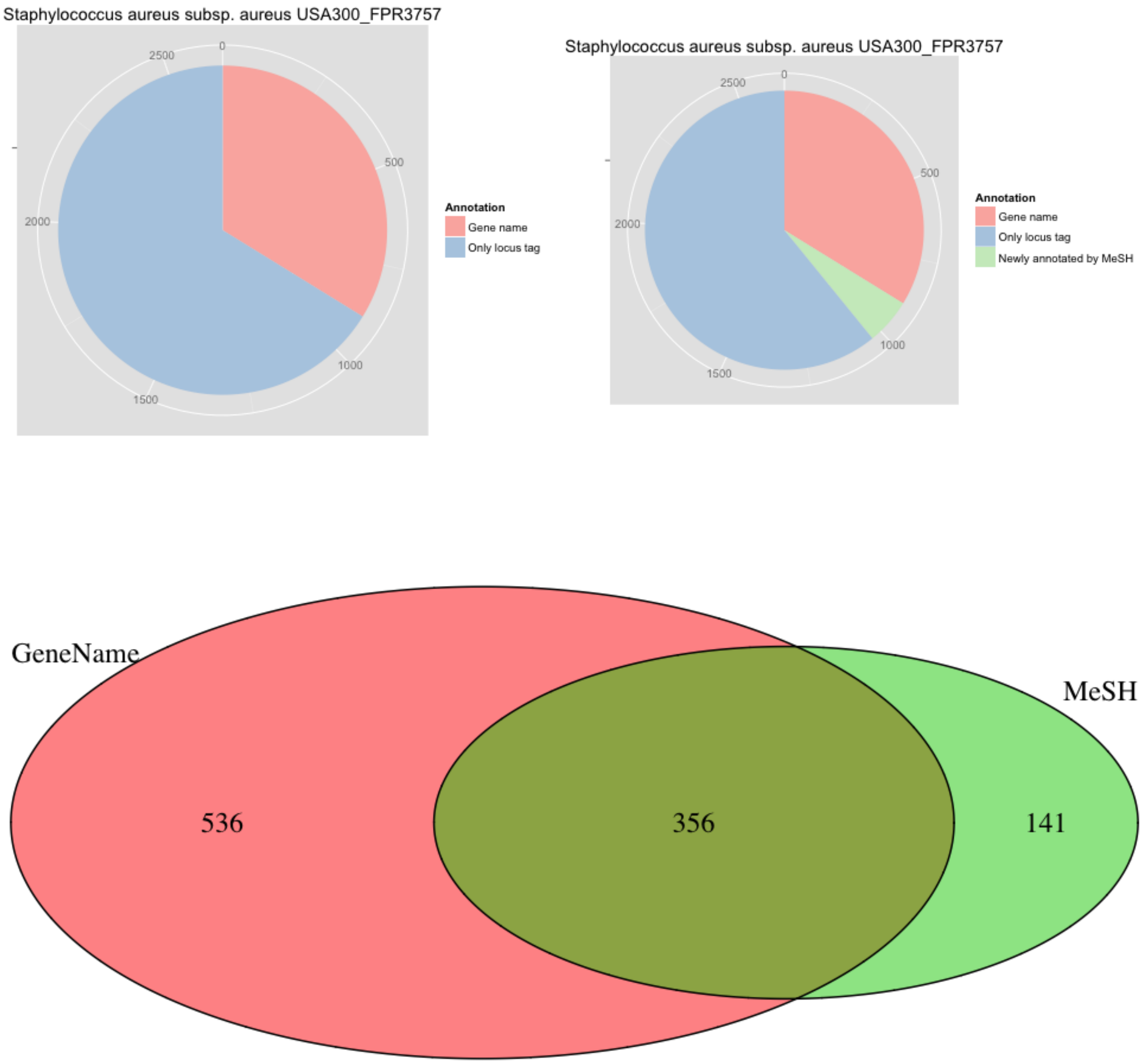

Figure 95: org.MeSH.Sau.USA300FPR3757.db

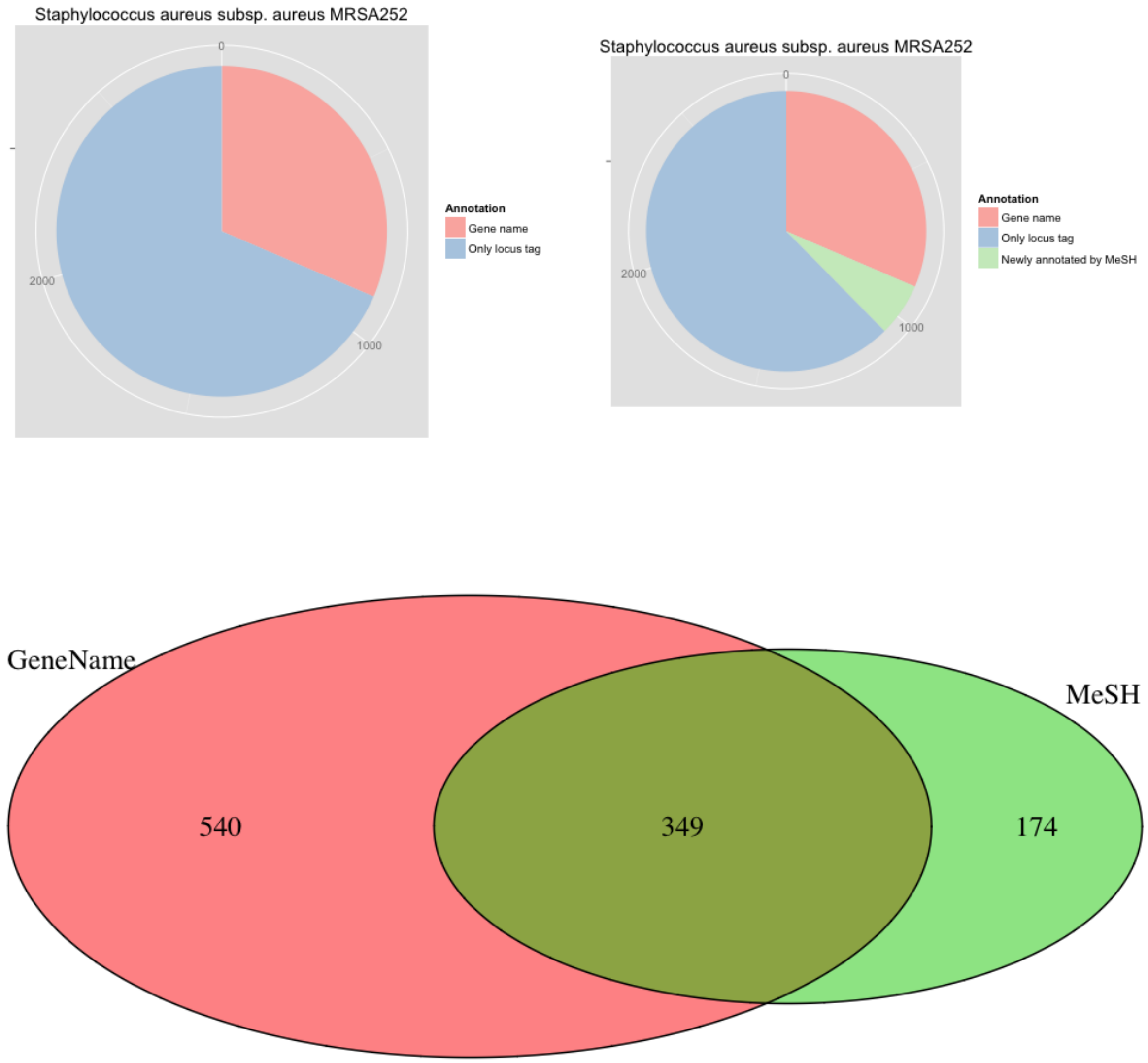

Figure 96: org.MeSH.Sau.MRSA252.db

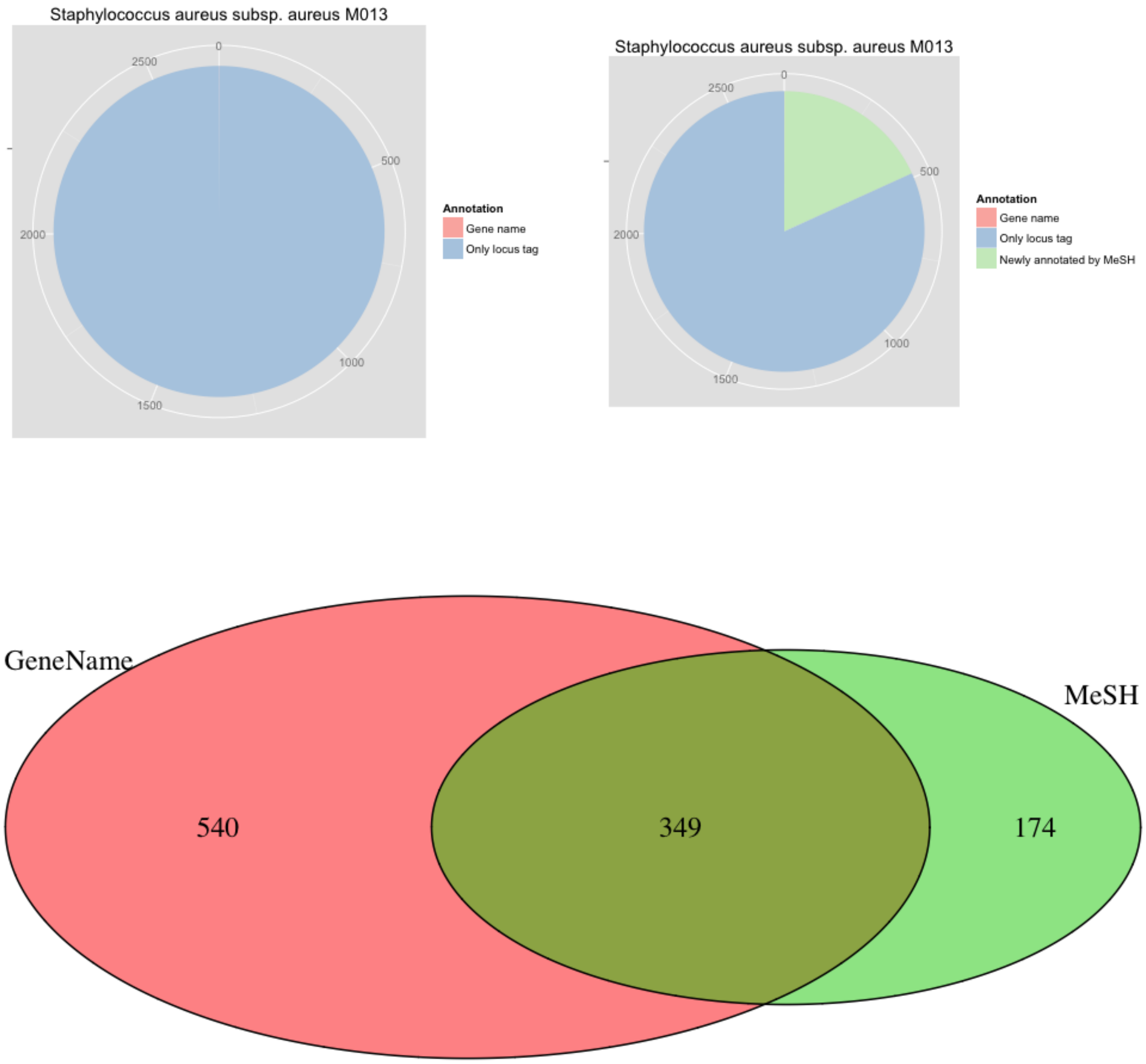

Figure 97: org.MeSH.Sau.M013.db

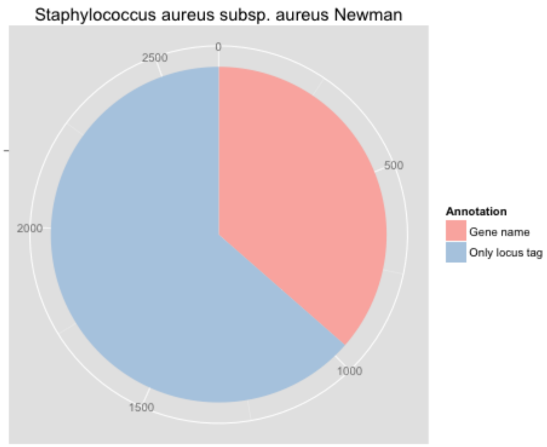

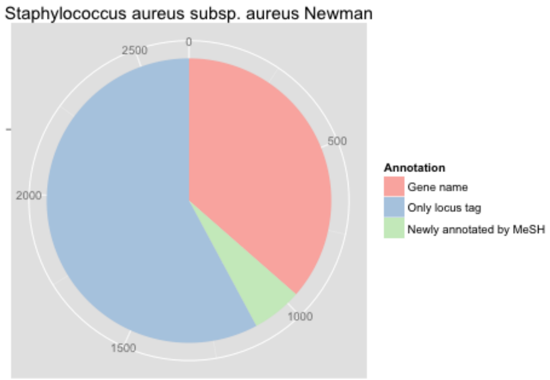

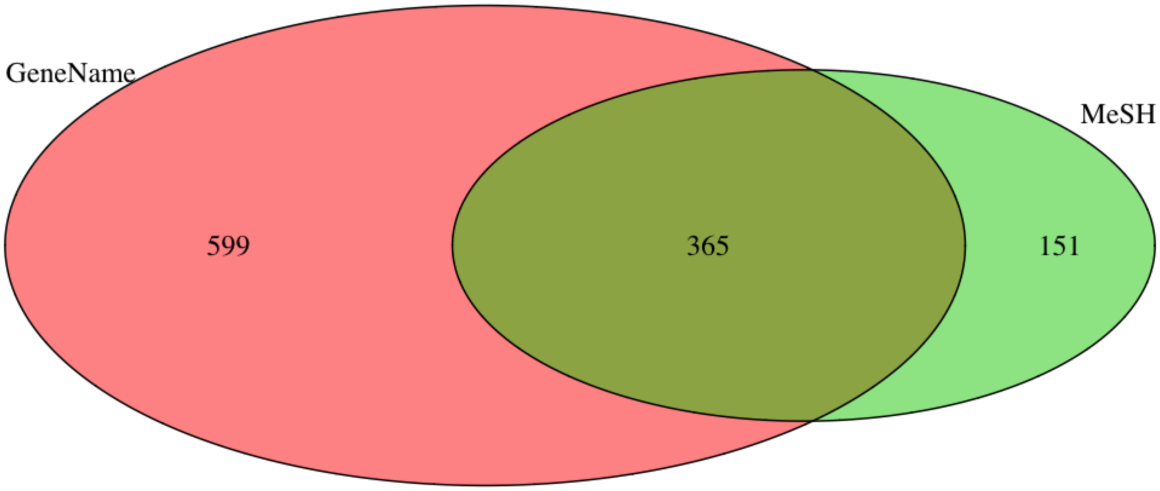

Figure 98: org.MeSH.Sau.Newman.db

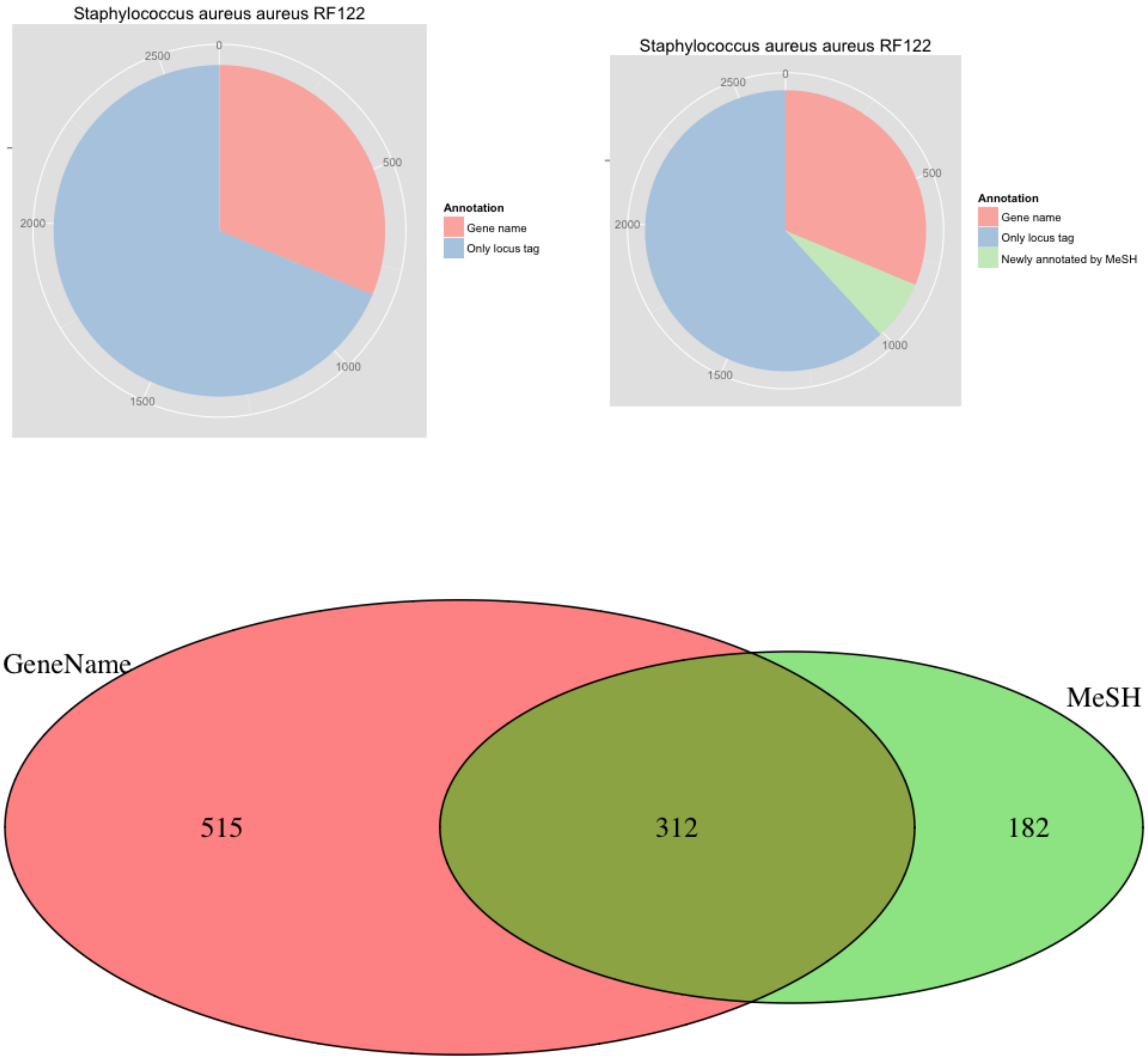

Figure 99: org.MeSH.Sau.RF122.db

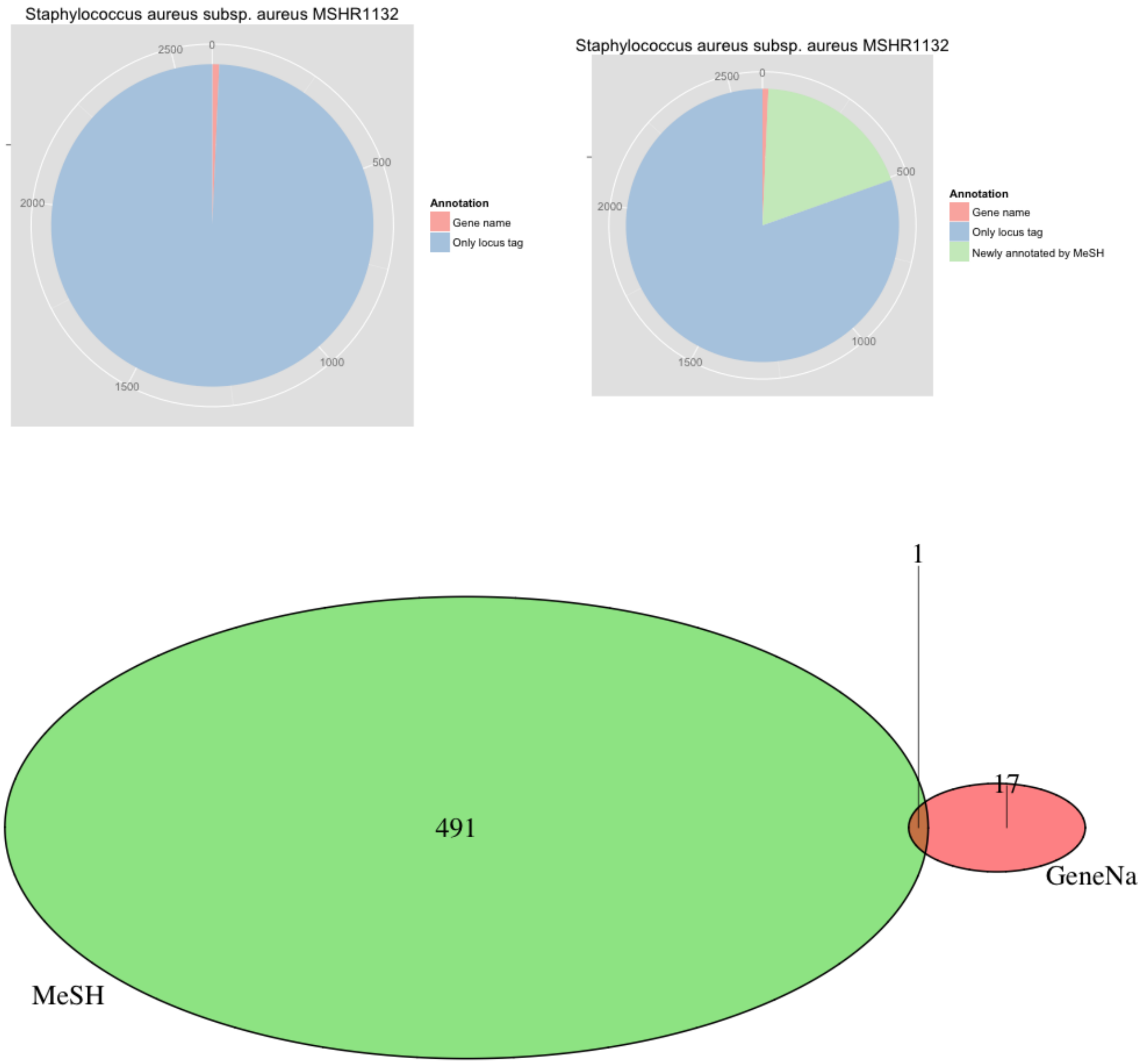

Figure 100: org.MeSH.Sau.MSHR1132.db

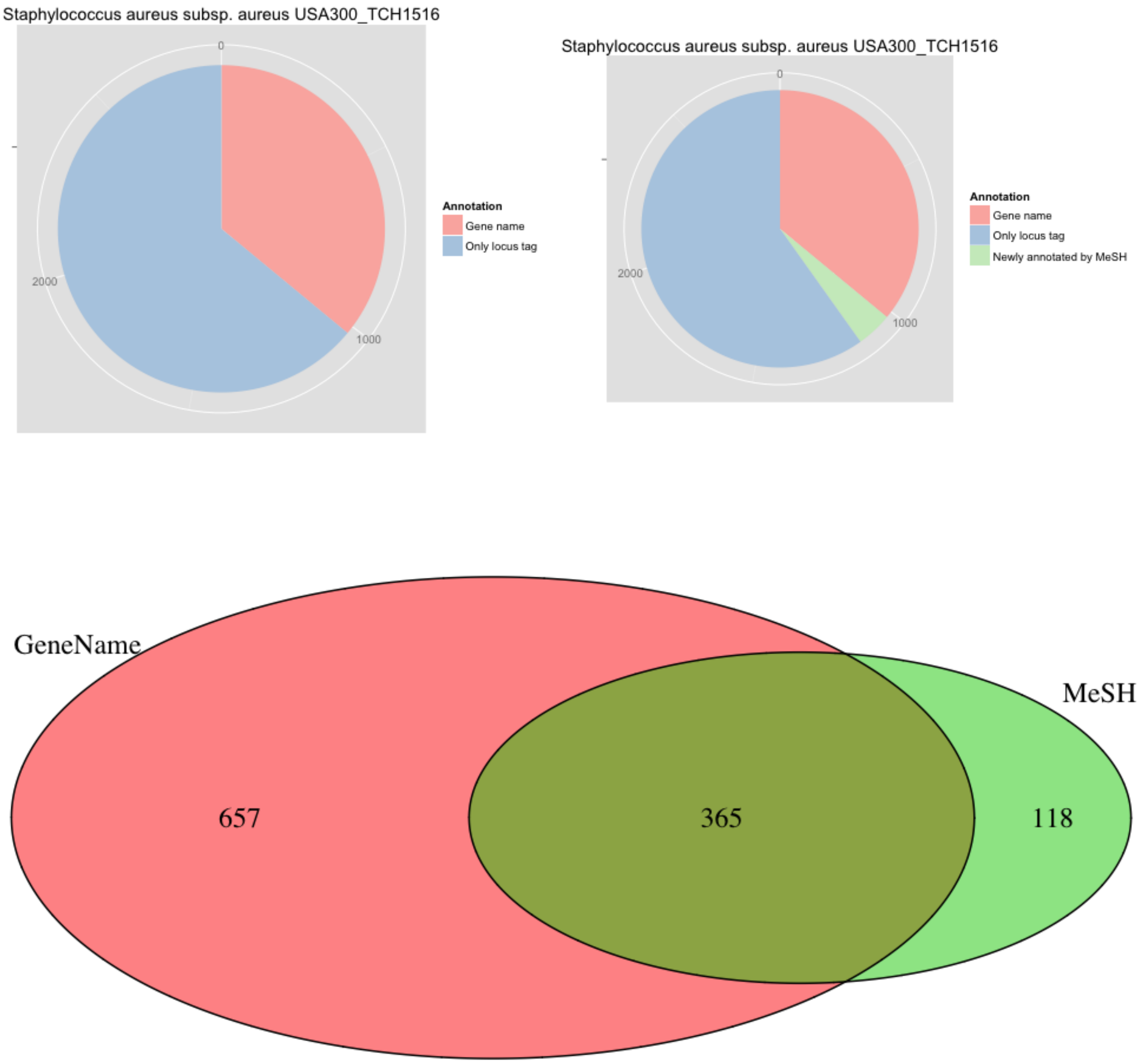

Figure 101: org.MeSH.Sau.USA300TCH1516.db

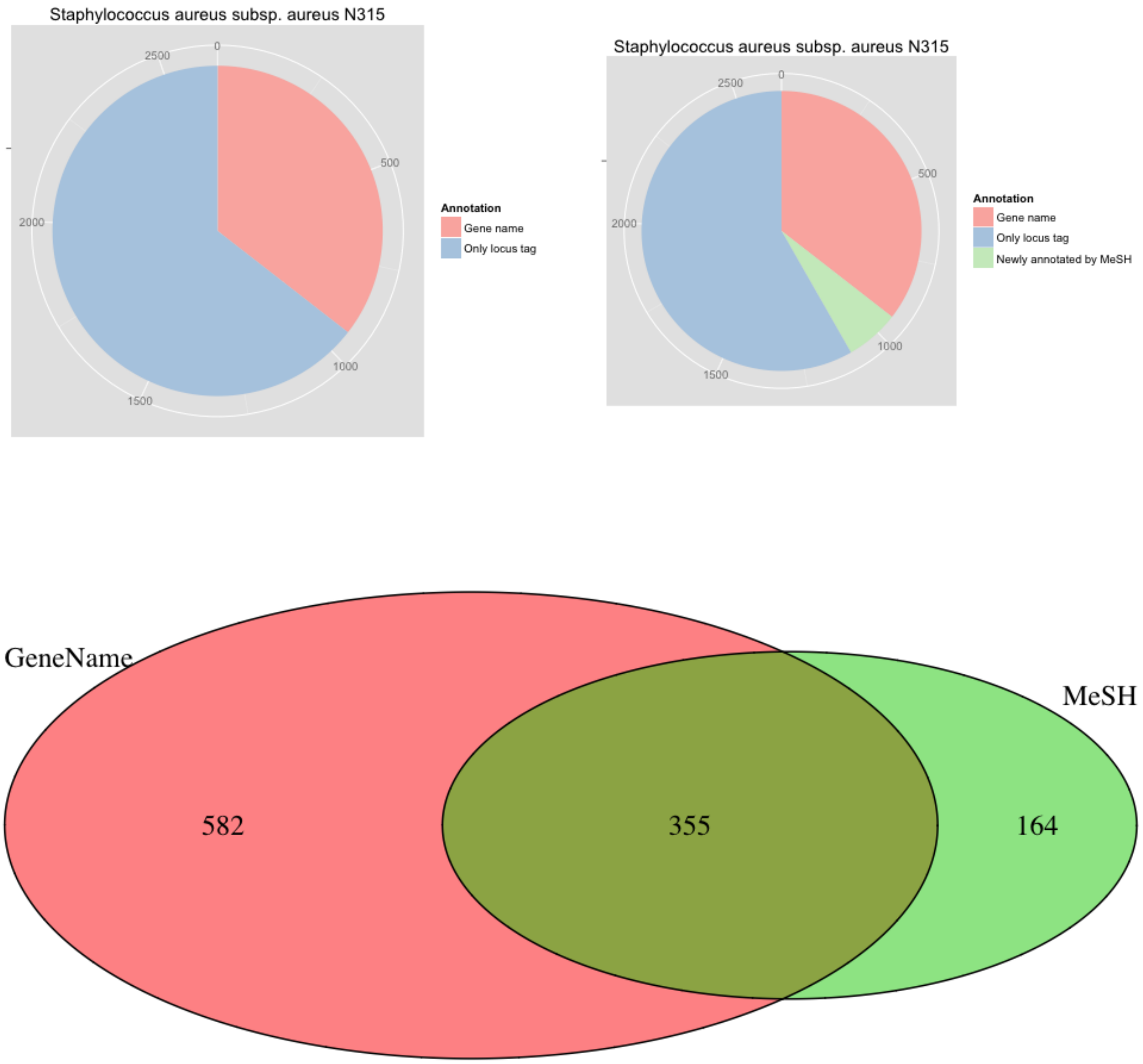

Figure 102: org.MeSH.Sau.N315.db

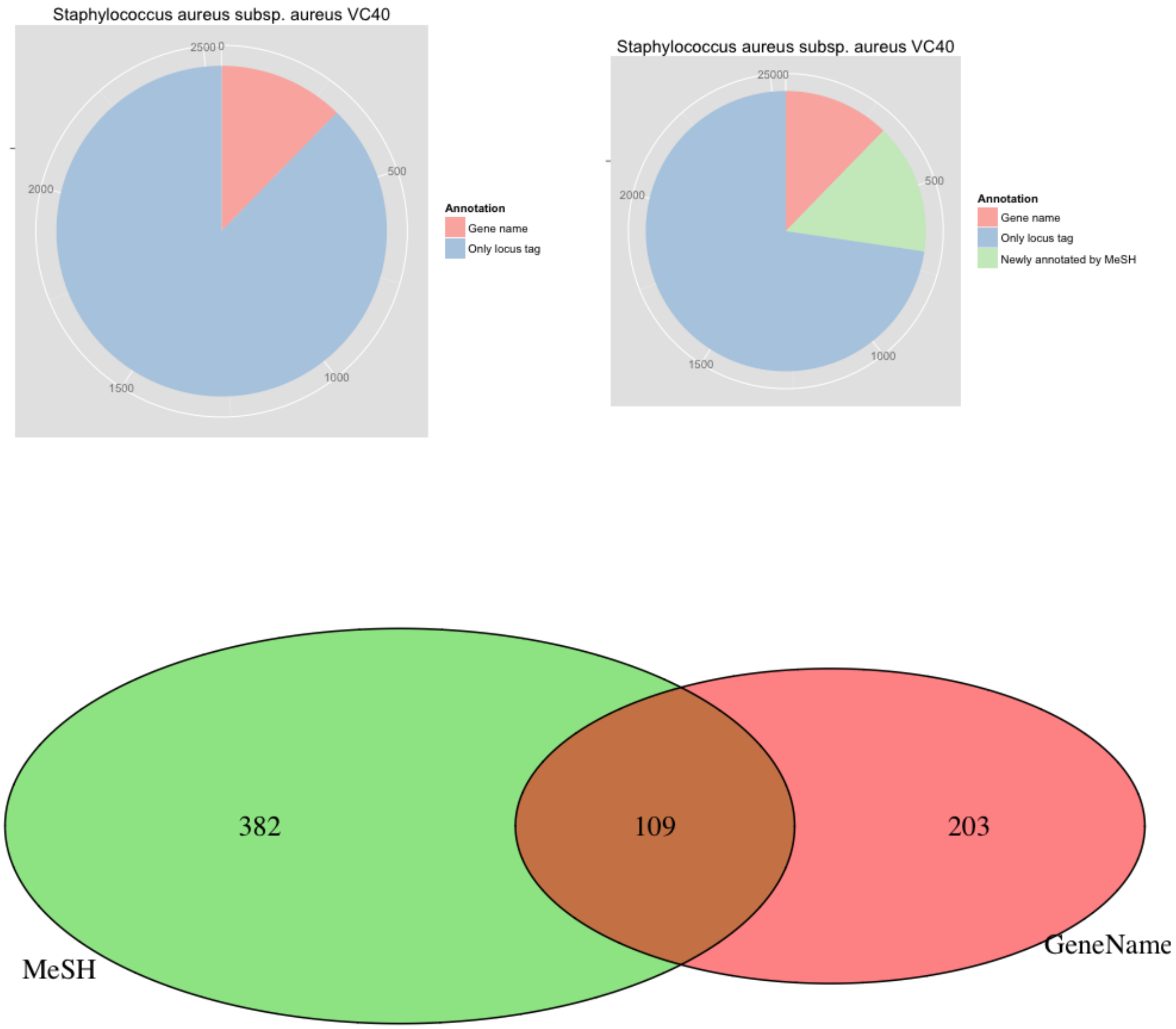

Figure 103: org.MeSH.Sau.VC40.db

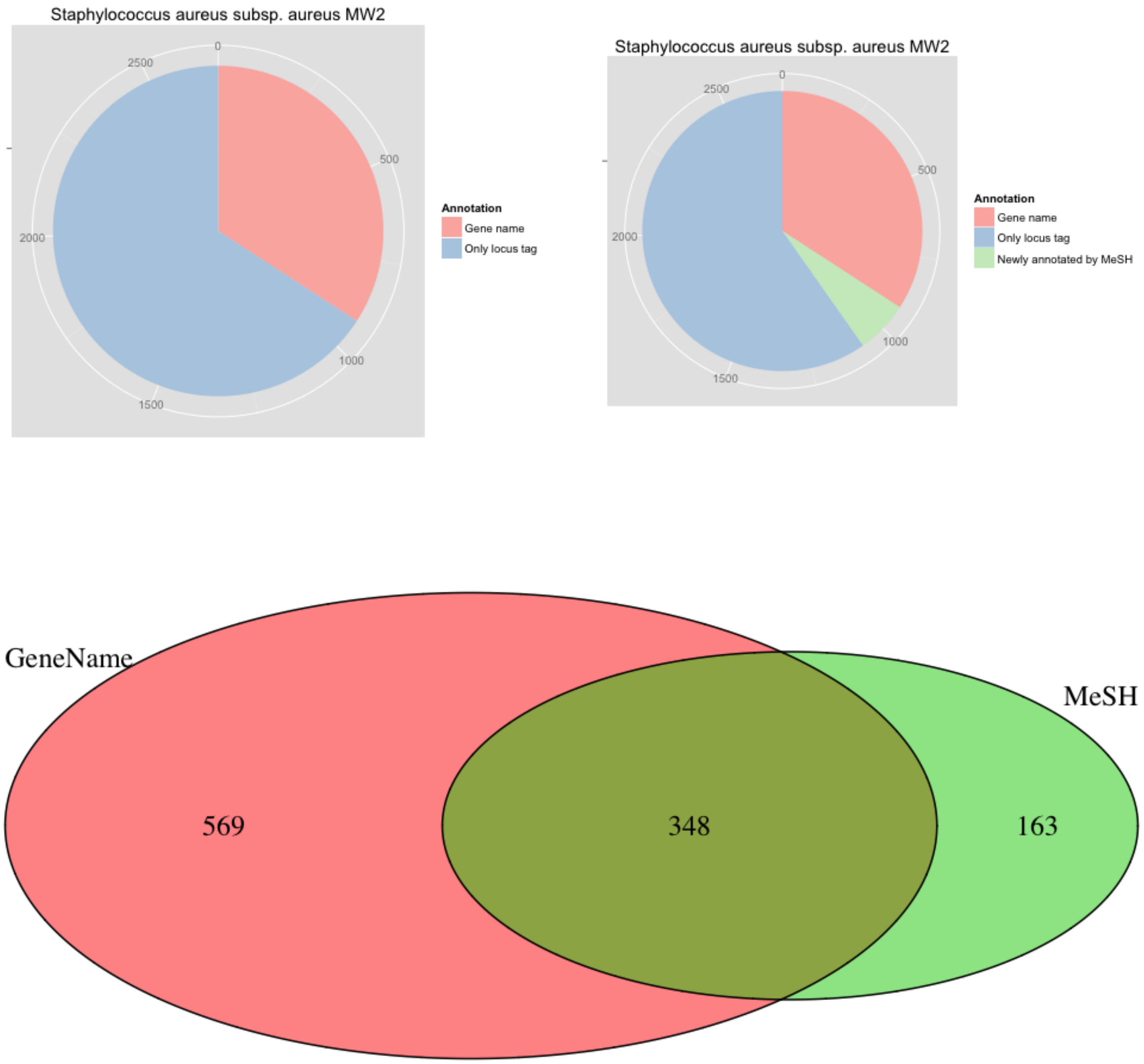

Figure 104: org.MeSH.Sau.MW2.db

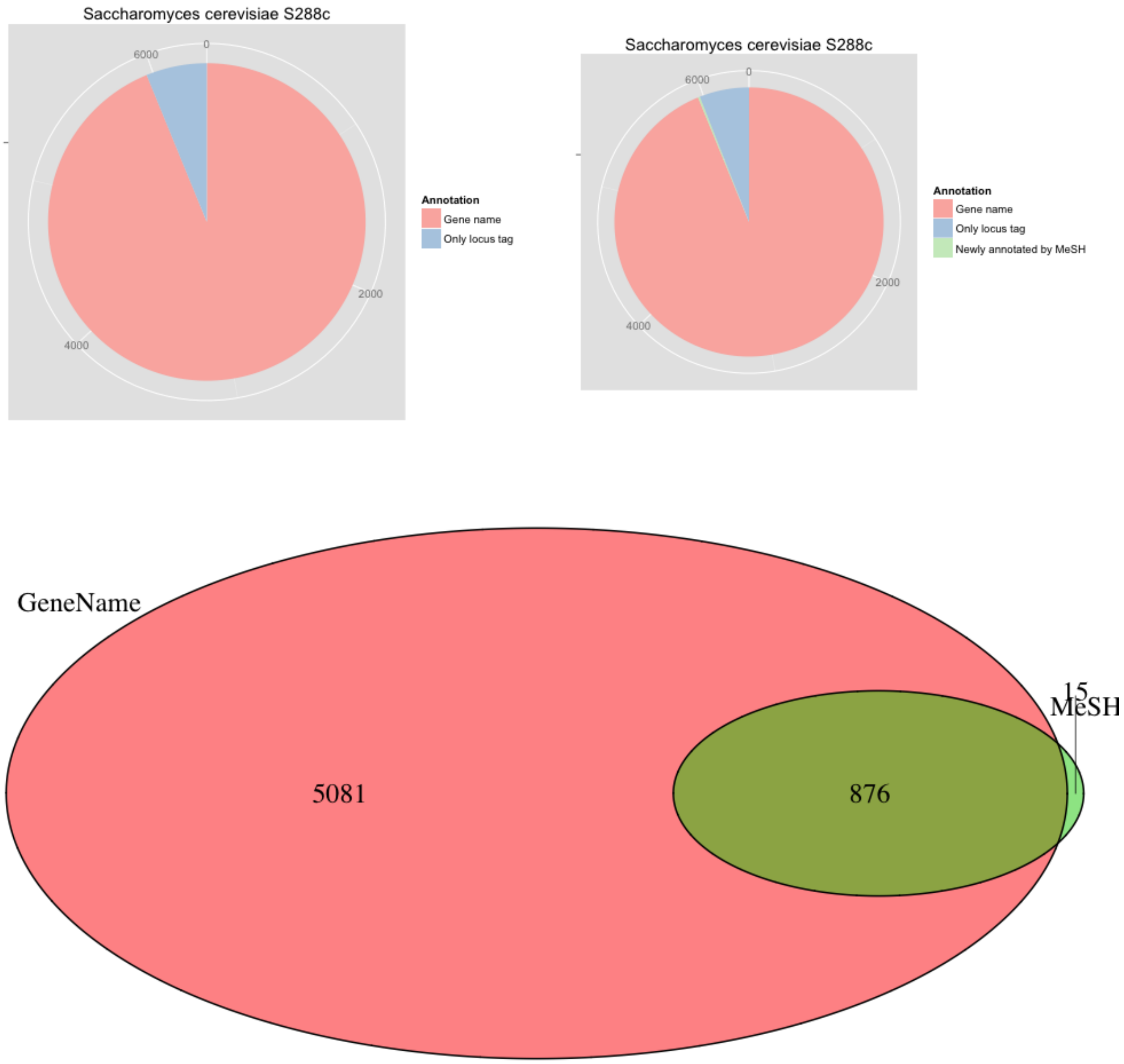

Figure 105: org.MeSH.Sce.S288c.db

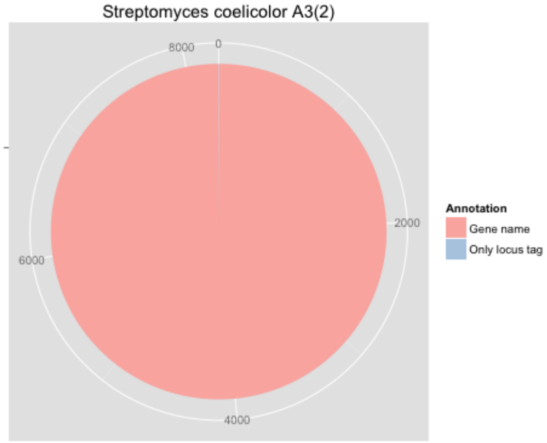

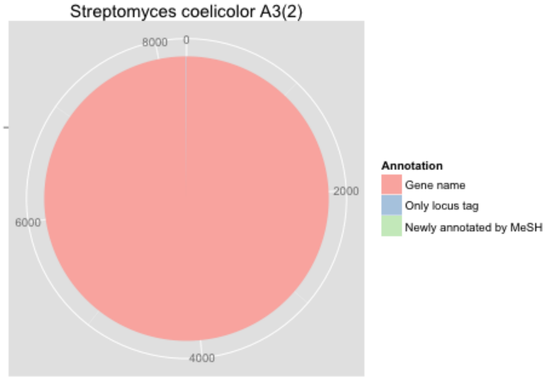

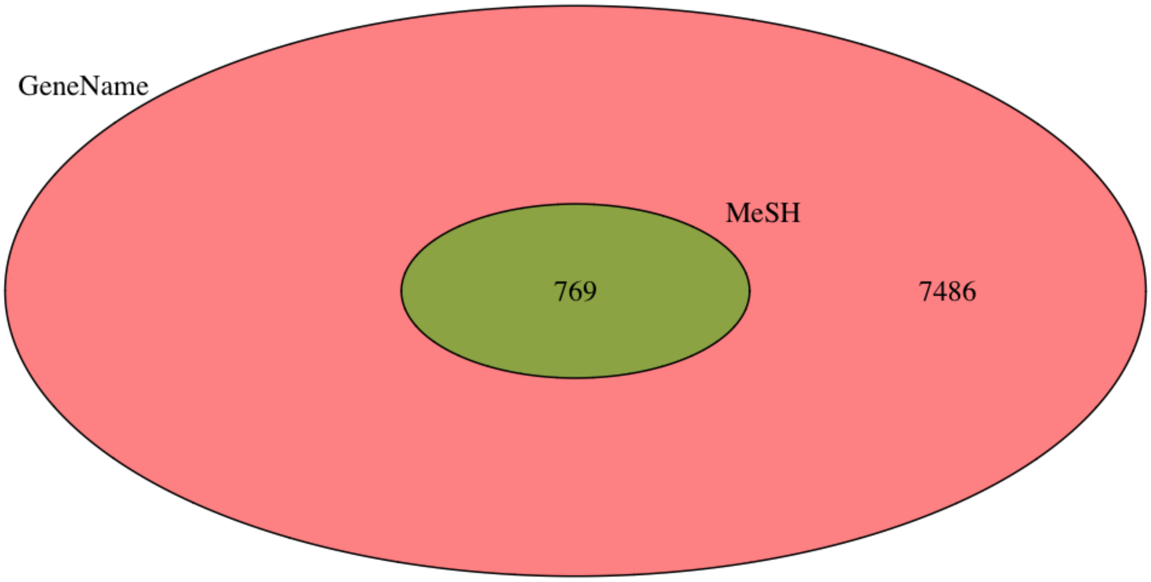

Figure 106: org.MeSH.Sco.A32.db

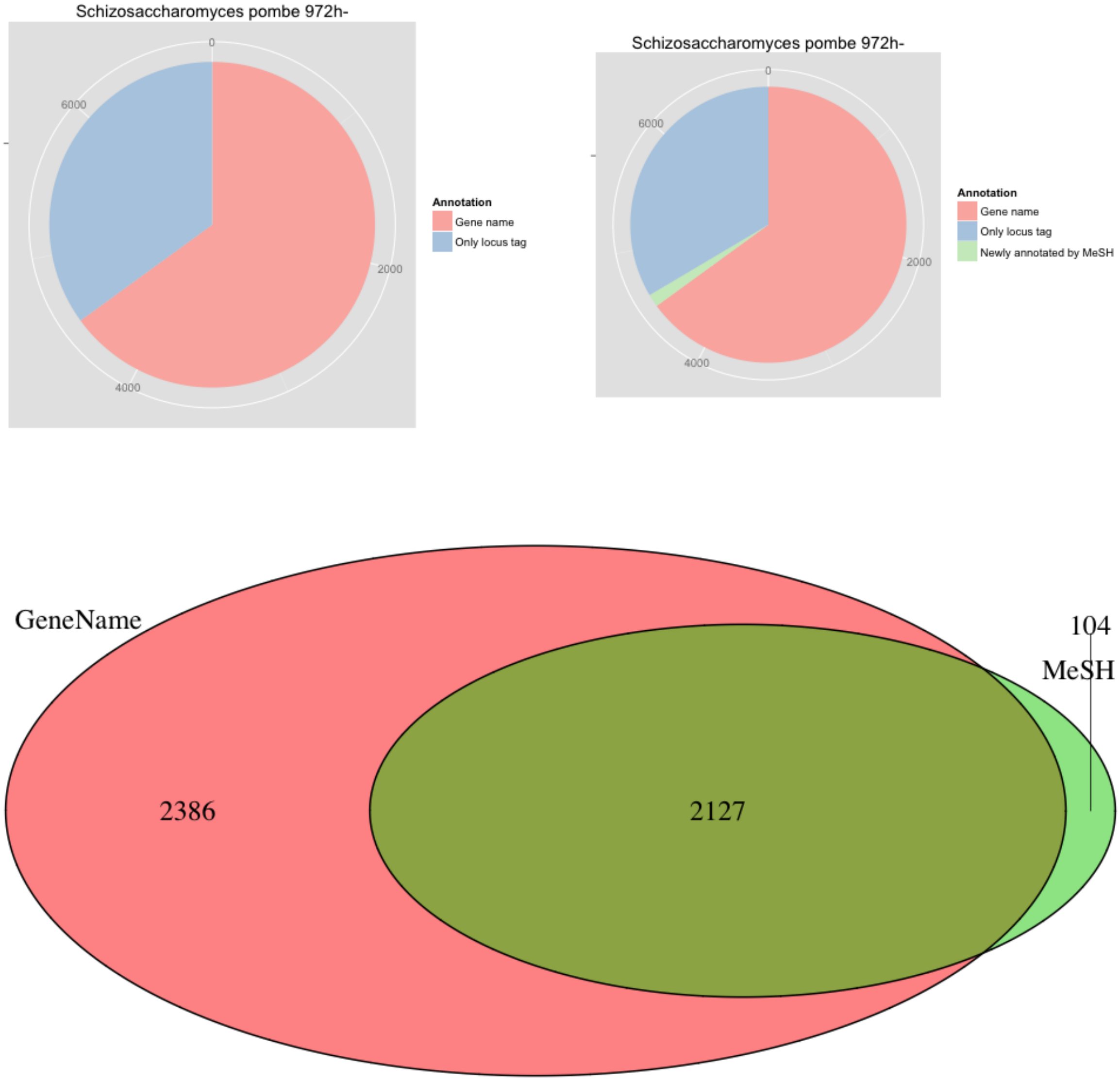

Figure 107: org.MeSH.Spo.972h.db

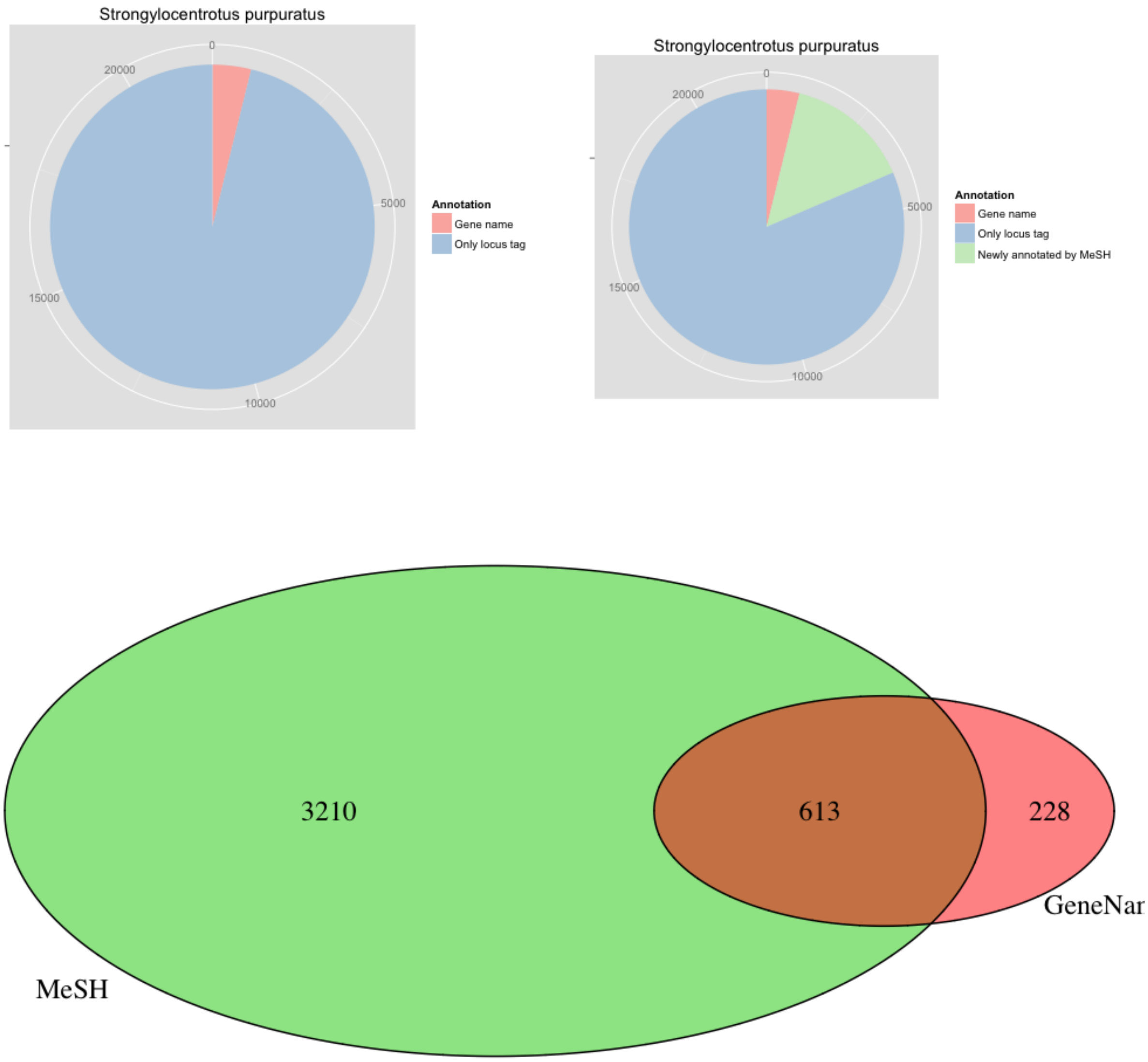

Figure 108: org.MeSH.Spu.db

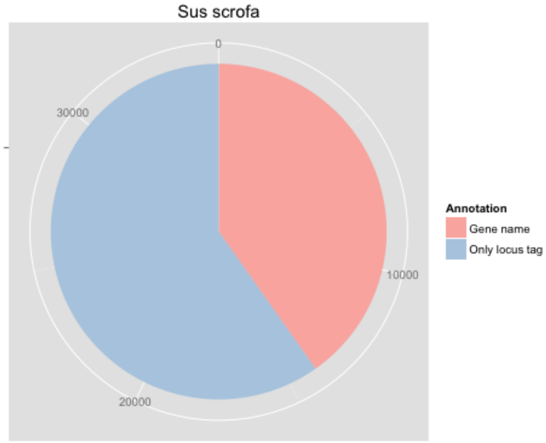

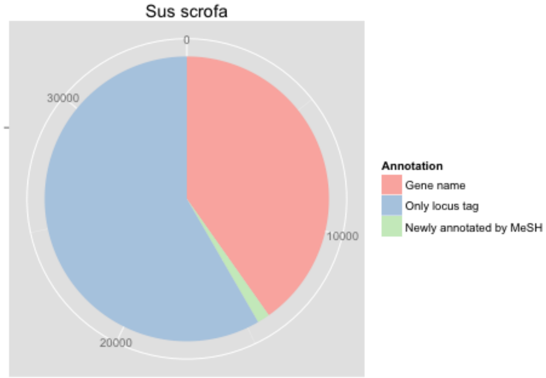

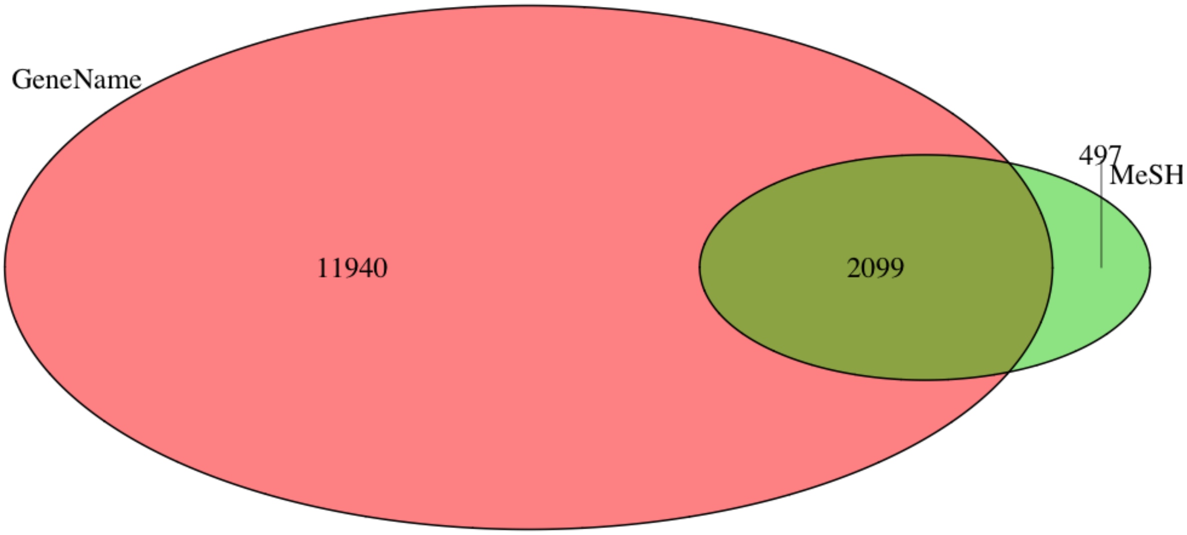

Figure 109: org.MeSH.Ssc.db

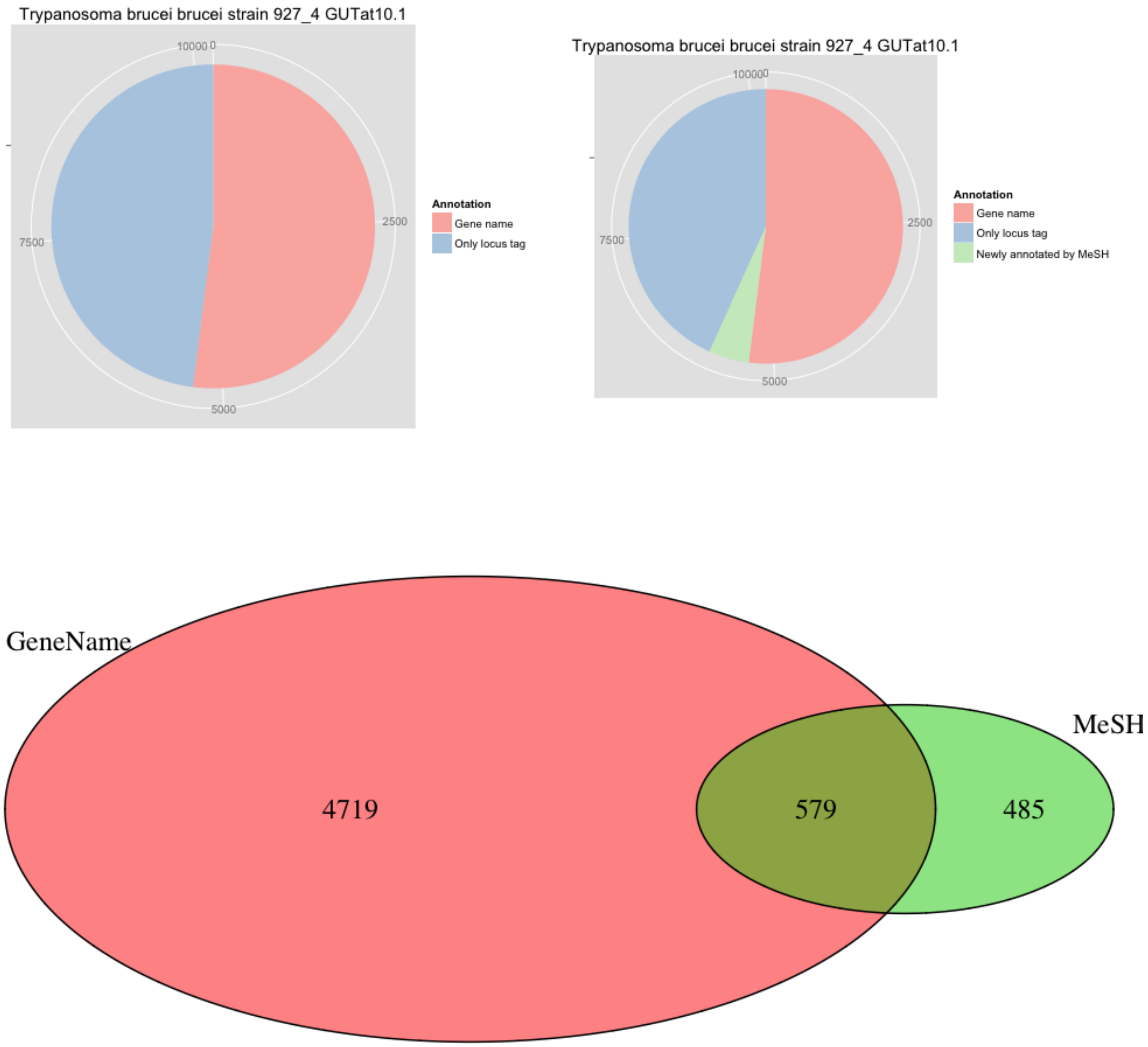

Figure 110: org.MeSH.Tbr.9274.db

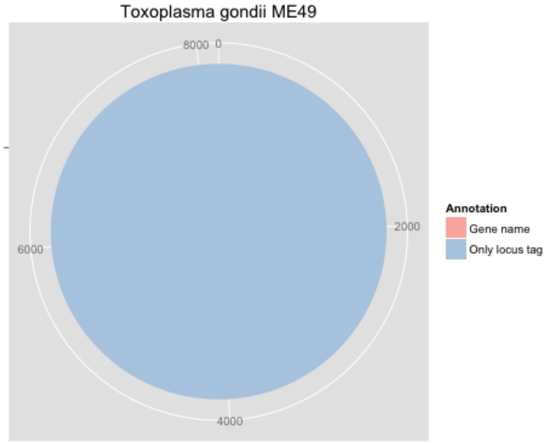

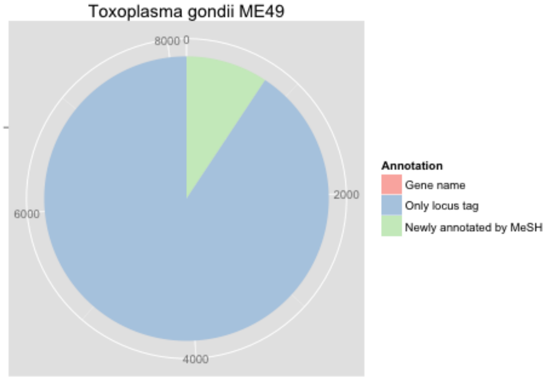

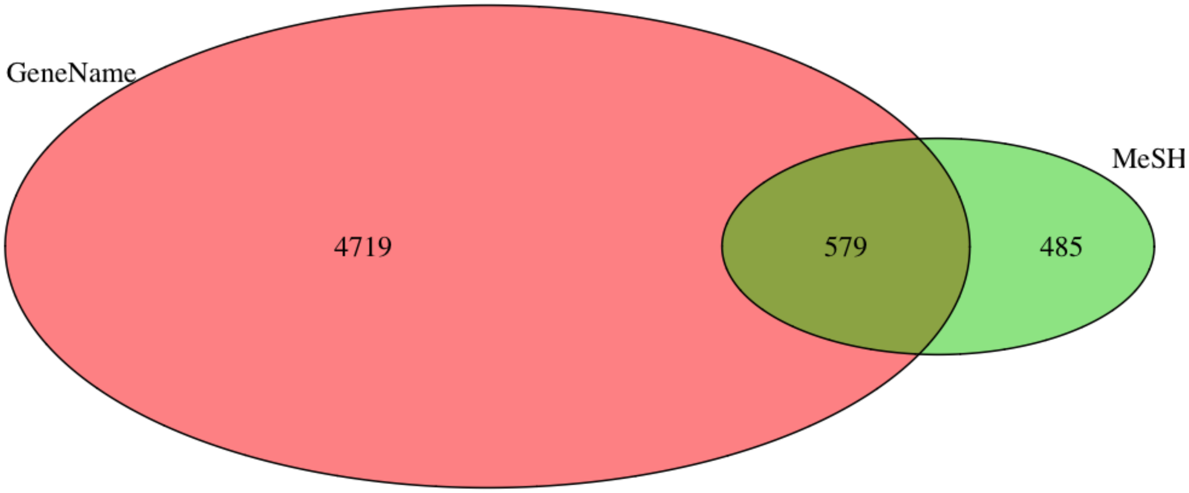

Figure 111: org.MeSH.Tgo.ME49.db

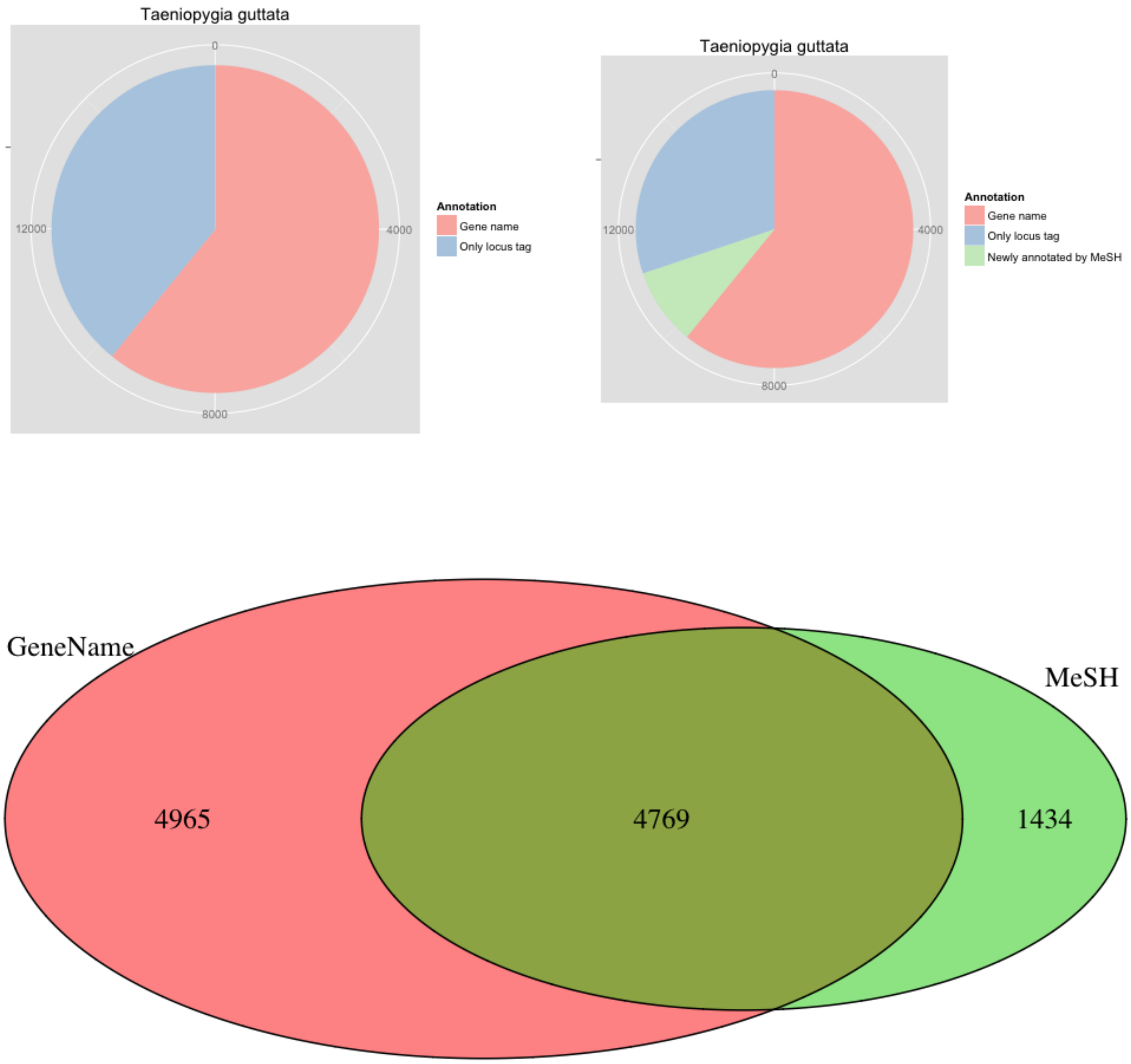

Figure 112: org.MeSH.Tgu.db

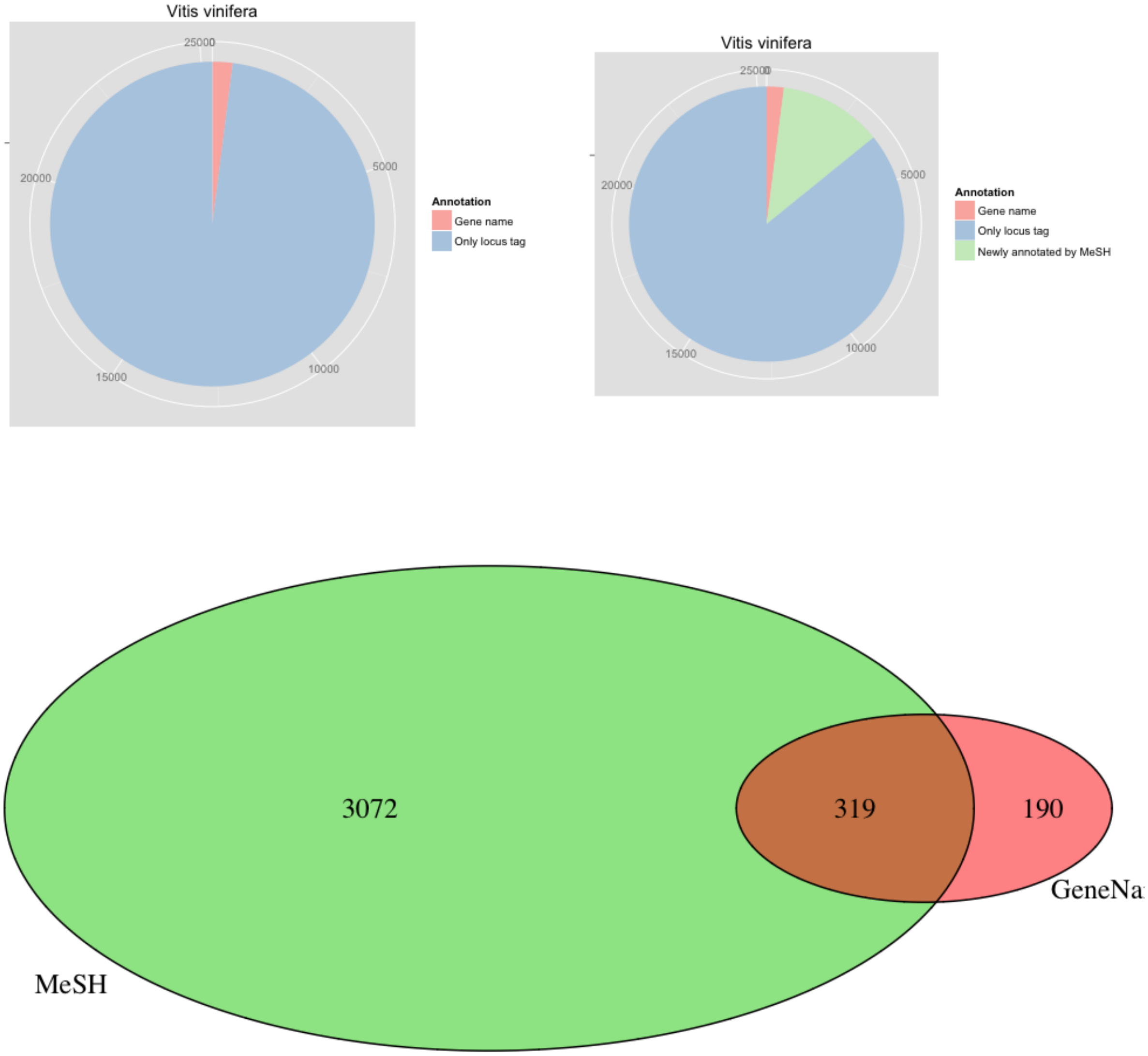

Figure 113: org.MeSH.Vvi.db

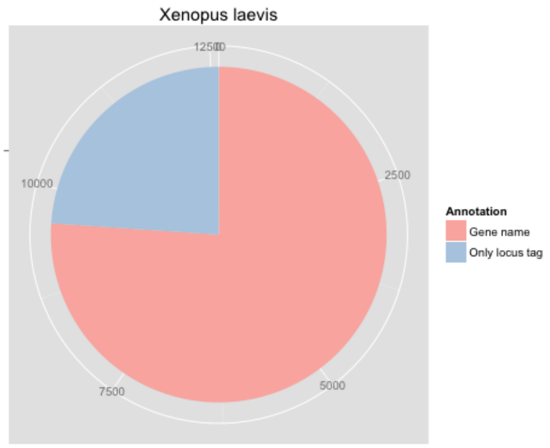

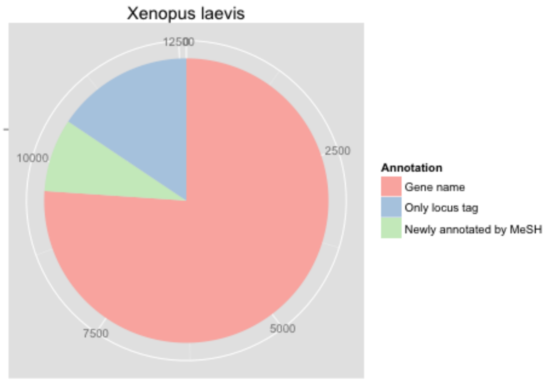

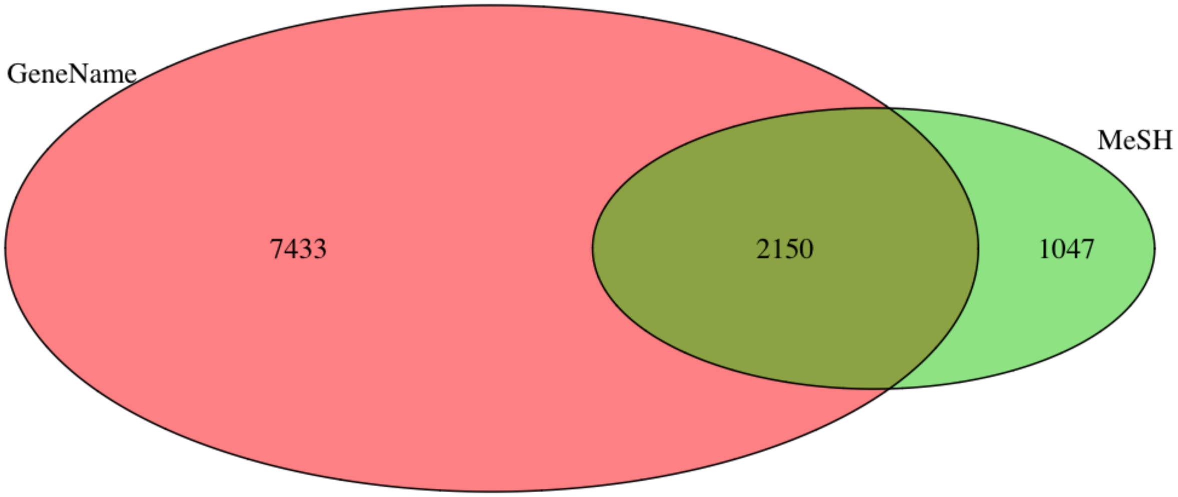

Figure 114: org.MeSH.Xla.db

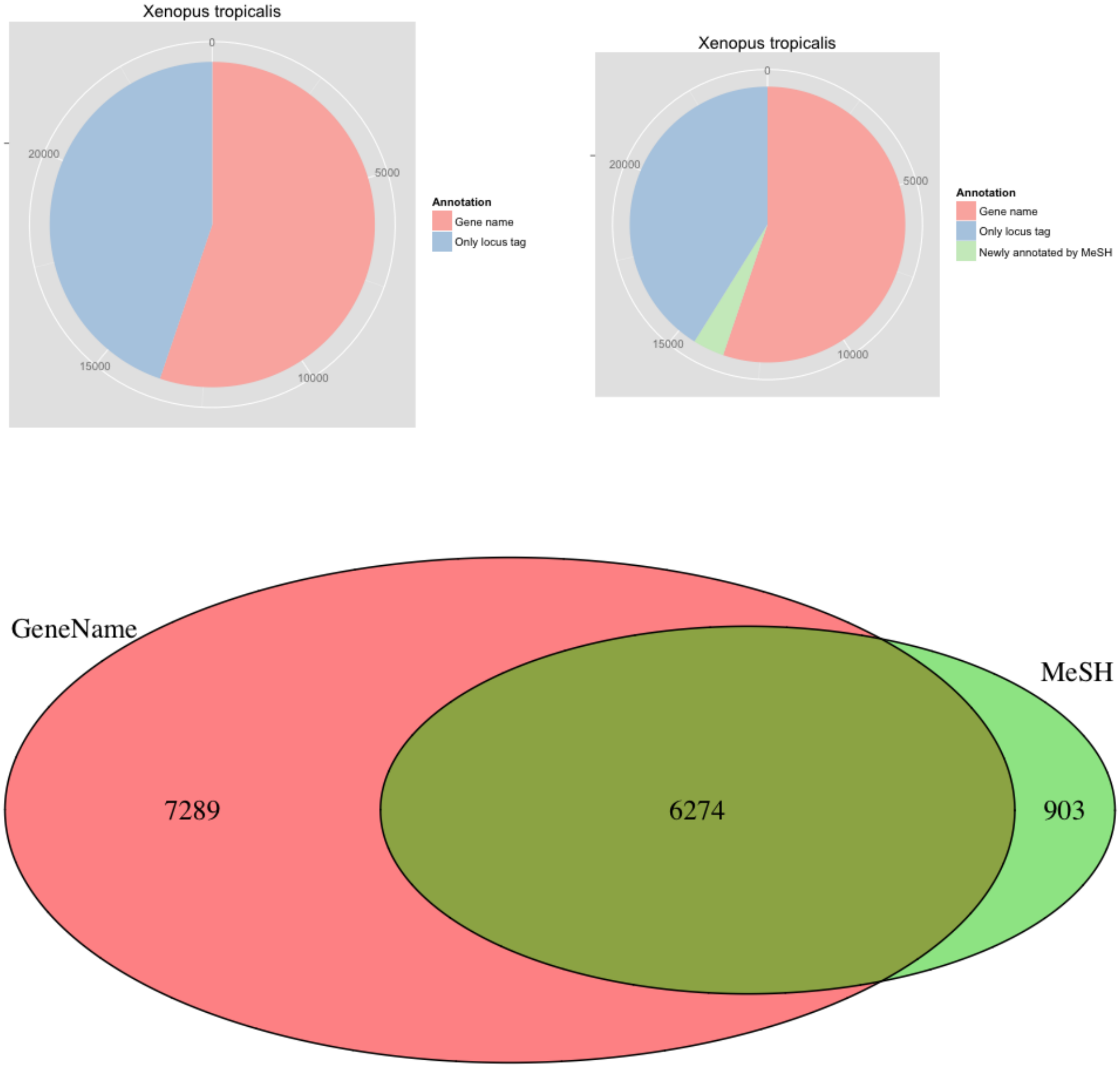

Figure 115: org.MeSH.Xtr.db

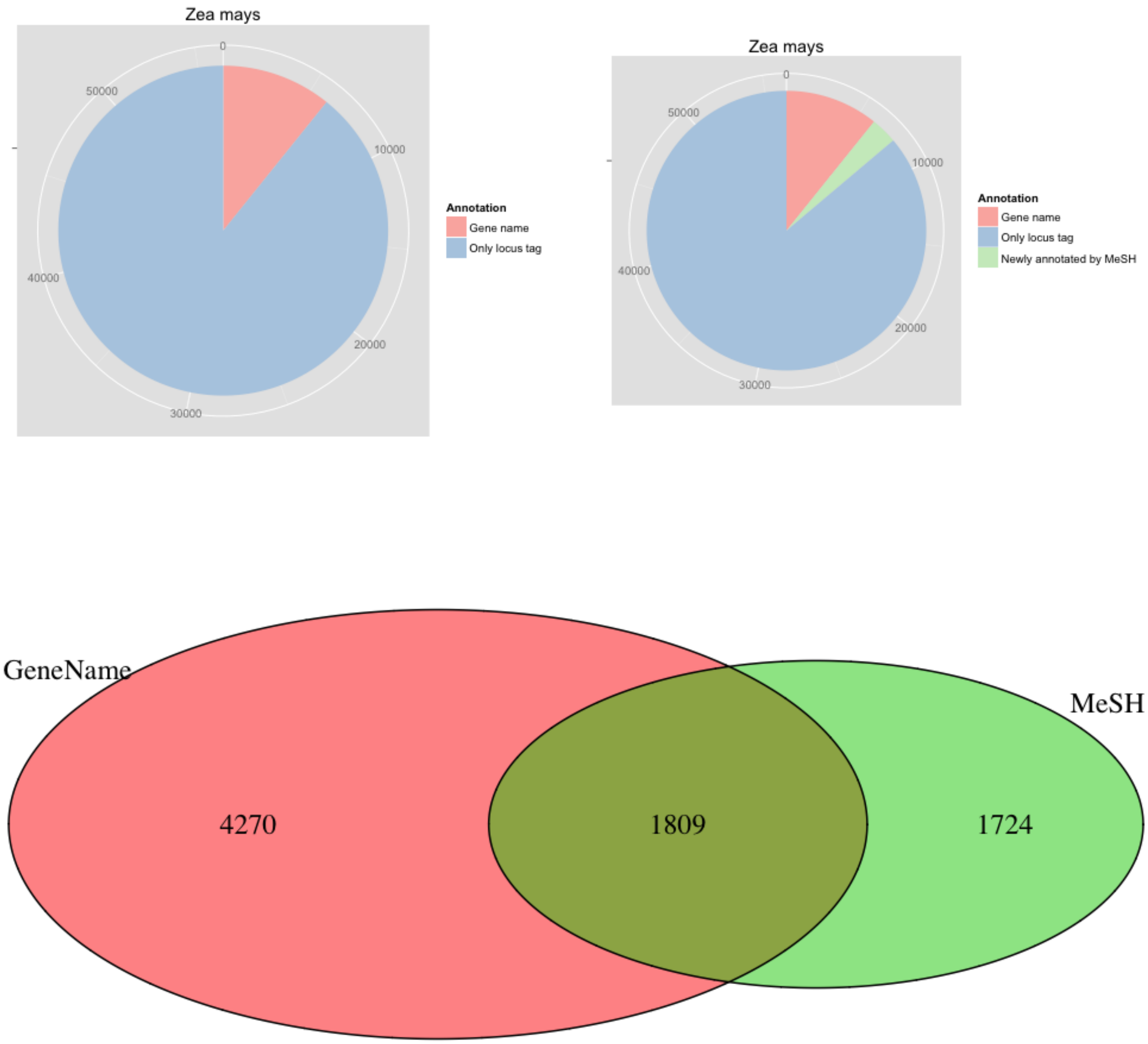

Figure 116: org.MeSH.Zma.db
